# A New Unusual Ice-induced Sedimentary Structure: the Silt Mushroom

**DOI:** 10.1038/srep36945

**Published:** 2016-11-11

**Authors:** Zhong Jianhua, Ni Liangtian, Sun Ningliang, Liu Chuang, Hao Bing, Cao Mengchun, Chen xin, Luo Ke, Liu Shengxin, Huang Leitong, Yang Guanqun, Wang Shaojie, Su Feifei, He Xuejing, Xue Yanqiu

**Affiliations:** 1 School of Geoscience, China University of Petroleum, Qingdao 266580, China

## Abstract

Upon channel bars or point bars within the lows of the Yellow River, a new sedimentary structure, named ‘silt mushroom’, has been observed. The process of their formation is interpreted to be via the ice process. The name, the silt mushroom comes from their figurative form. This is because they look somewhat similar to mushroom’s in size and shape; being in the range of 1 to 10 cm in diameter, with the medium 3–5 cm, and on average 10 cm in height, occuring generally in groups, and occasionally in isolation in relatively soft silt. They develop in the transition from winter to spring, and are convincingly related to ice processes. Ice-induced silt mushrooms are best examined in association with the many other newly discovered ice-induced sedimentary structures (over 20 kinds). Clearly, up to now, ice processes have been significantly underestimated. With the substantial discovery of the ice-induced silt mushroom, it opens up new questions. This is because its structure mirrors the same sedimentary structures found in rocks, questioning their genesis, and sedimentary environment analysis. This achievement is significant not only in sedimentology, but also in palaeogeography, palaeoclimate, geological engineering, hydraulics and fluviology.

The Yellow River, is the second largest river in China, and is well known for the highest levels of mud and silt in the world^1^,^2^,^3^. It has therefore attracted an increasing amount of attention since the late 1990s, because of its interruptions of flow. These interruptions cause a lot of unusual sedimentary environments and sedimentary structures to be formed. In the early 2000’s, many sedimentologists undertook a large degree of research upon the Yellow River, obtaining many accomplishments^4^,^5^,^6^,^7^,^8^,^9^,^10^,^11^,^12^,^13^,^14^,^15^,^16^, including the discovery some ice-induced sedimentary structures^17^,^18^,^19^,^20^,^21^,^22^,^23^,^24^. However since that period, because of low economic profits, few studies have been undertaken on the Yellow River. Nevertheless in the last two decades, following from a series of discoveries upon new sedimentary structures, we engaged a fresh study on the Yellow River. This study led us to discover a unique ice-induced sedimentary structure that has not been documented yet. As it looks the similar to a mushroom in its shape, this new sedimentary structure is termed an ice-induced silt mushroom. More remarkably, this exquisite and peculiar sedimentary structure, formed through natural processes, has hardly been documented in the major sedimentologic publications^25^,^26^,^27^,^28^,^29^,^30^,^31^,^32^ or papers^33^,^34^,^35^,^36^,^37^,^38^,^39^,^40^,^41^,^42^,^43^,^44^,^45^,^46^,^47^,^48^,^49^,^50^,^51^,^52^,^53^. Consequently, we believe that these newly discovered ice-induced silt mushrooms are a novel sedimentary structure, which is of great scientific significance and aesthetic value. This is especially so, at present, as the majority of sedimentologists’ attentions are attracted towards general sedimentary structures, with only few ice-induced sedimentary structures having been recognized. Hence, in this paper and to address this academic imbalance, we will intensively focus on ice-induced sedimentary structures. As the ice-induced sedimentary structures from the lower of the Yellow River may involve up to more than 20 new types, our discussion in this paper will be specific to the silt mushrooms, situated in the river’s lower, from Lijin to the estuary of the Yellow River.

Being the second largest river in China, and 1 of the 10 largest rivers in the world, the Yellow River has a drainage basin covering ~752,443 km^2^ (Fig. 1), a length of ~5464 km and a mean discharge of ~58.0 × 10^9^ m^3^/year. The Yellow River is especially known for its tremendous sediment load of ~16.0 × 10^9^ m^3^/year, which consists mainly of silt and clay, derived from the loess plateau^1^,^2^. A quarter of the sediment load is deposited in the channel, within the lower part of the drainage basin, whilst approximately half is deposited in the estuary region, and the final remainder deposited in the shallow waters of the Bohai Sea^3^. Due to the heavy silt deposits, the main channel is both narrow and shallow, commonly less than 10 m wide and 2 m deep when it is in low flow. This facilitates the overflow of flood water onto the point bars and channel bars. Small-scale flooding of approximately 1000 m^3^/s, can rapidly overflow the main channel and soak the point bar and channel bar. This sedimentary induced flooding, means the dry riverbed and bars can rapidly submerged within 1 hour.

In winter, the temperature in the section, from Binzhou bridge to the estuary, of lower of Yellow river starts frosting from November 11, the lowest minus 23.3 degree, and to end frosting to March 21 every year. During the transition from winter to spring every year, generally in March of every year, an unusual hydrologic situation occurs. This environmental change stimulated by increasing temperatures, causes ice flow, which commonly blocks the river channel (Fig. 2A) and some of which are washed onto the channel bars and/or point bars in the river’s lower (Fig. 2B). As a result, many ice blocks within the river current, overflow the point bar or the channel bar, creating a unique scene and forming interbedded silt layers and ice layers like a sandwich (Fig. 2C,D). Basing on the detailed observation, we find that the river water near the bank and on the point bar is readily frozen for the flow velocity is relatively slow and the river water in the middle of the river is relatively difficultly frozen for the flow velocity is relatively fast. So the thicken of ice layer near the bank and on the point bar is greater than that of the middle of the river. When entering the ice flow, a lot of ice layers are broken and washed to the lower or the estuary (Fig. 2A,B). Meanwhile, a lot of ice blocks are also washed onto the channel bars and point bars resulting in forming interlaced layers of ice and silt, a sandwiched formation (Figs 2C,D and 3). This provides basic favorable conditions for the formation of ice-induced sedimentary structures^17^,^18^,^19^,^20^,^21^,^22^,^23^,^24^. Otherwise, the banks of the lower of the Yellow river is frozen and thawed during the transition from winter to spring, but this does not directly influence the formation process of silt mushrooms and other ice induced sedimentary structures apart from ice frozen bubbles and ice frozen fissions. However, it is instrumental for the knowledge of how ice-induced sedimentary structures are formed, to know the features of the deposit in the study area.

The sediment load in the river is of an extremely fine grain, and because of it being easily moved by ice, or ice melt water, it is highly favorable to the formation of ice-induced sedimentary structures. The recording taken at the Lijin Hydrometric Station in 1987 shows that the sediments consisted of; less than 10% fine sand, 50–70% silt and 30–40% clay^14^. For our granulometric analysis, we collected six samples from our study areas in which ice-induced sedimentary structures have developed, all locations were on a bed material that consisted predominantly of fine silt, with a grain size distribution between 0.032 and 0.128 mm (Fig. 4)^14^. Generally, the constitution of ice-induced silt mushrooms is of poorly-sorted silt, sometimes with small muddy lamina. In a word, the sorting of the silt mushrooms is not as same good as the silt in the channel bar or point bar because of being stirred by ice flows.

## Features of the silt mushroom

The term ‘ice-induced silt mushroom’ (or ice induced silt-mushroom-like structure) is introduced herein to define a relatively small, circular, vertical, mushroom-like, ice-induced sedimentary structure (Fig. 5). It must be pointed out in the first place that the form is generally a silt-rich depositional body, sharing somewhat resembling small sand-boils (in size, shape and material composition), but differing from those forms in its internal texture, shape, formational environment, accompanying structures, and especially genesis. Currently, the silt mushroom occur in group and occasionally is also exposed individually. Ice induced silt mushrooms often develop on the surface of the channel bar and\or point bar during the transition from winter to spring.

To describe the form simply, it visually has a mushroom shape, mostly of a short and thick mushroom (Fig. 5A,C,H). The silt mushrooms structure can be divided into three parts: cap, stem and base (Fig. 6). The normal cap of a silt mushroom is circular (Fig. 5D) and nearly circular, mostly irregular shapes (Fig. 5B,D,G). So most silt mushrooms are actually irregular shaped (Fig. 5B), and this irregularity is defined by the variety in stem shape, internal textures and thickness of the silt layer on the stem. The cap is generally not flat (Fig. 5H), and ranges in thickness from 2–4 cm (Fig. 5C,D,G,H), the maximum thickness up to 10 cm. The cap is formed through the collapse of the thin silt layer on the stem top, this is caused by the ice layer melting because of temperature increase during the transition from winter to spring. So the surface develops ice crystals, frozen cracks and other ice induced sedimentary structures and the internal forms are ice-induced sedimentary structures (commonly small-scale vertical ice-induced fissions or ice frozen bubbles) with their normal beddings formed from, in general, the deposition of channel bar and\or point bar; horizontal beddings (Fig. 5C), small ripple lamination beddings, small climbing beddings and small tabular cross beddings. The cap is relatively small whilst the stem is relatively large (Fig. 5C) and this is closely related to be influenced by dense frozen fissures according to freezing and thawing, for dense frozen fissures make the cover on the stem being easy to be broken and make the cover on the stem to collapse closely near the stem. However, when the stems occur densely and laterally, one by one, the layer on the stem may not collapse and thus a single cap does not form (Fig. 5A,G,H). This leads to the formation of a connected large mushroom (Fig. 5H). As a result, contrasting to this situation, only the stem of a silt mushroom may form (Fig. 5E). This situation may be very common in the field. The cap of these interconnected mushrooms is commonly 5–6 cm in thickness, with the thin silt layer covering the stems having commonly as same small ripples on the surface as the surroundings, which has not been covered by ice block and has not sandwich textures, and small sand rippled cross beddings in the internal, of about 1 cm in height with fissures formed by collapse. This phenomenon suggests that the cap is formed at the same time and in the same way as the surroundings.

The stem is the main and most important element of a silt mushroom. In general, the stem has an uneven height, a nearly columnar shape with somewhat peculiar curve surface like a mustard stem (Fig. 5H), and its surface is smooth (Figs 5B,C,G and 6), resulting from the smooth wall of the holes in the ice blocks or has anti-steps and vertical shallow grooves (Fig. 5E,F) that are formed by traction of collapsed covering layer near the stems. This has been proven by the experiment (see the following). Sometimes, the stem has small odd warty heave (Fig. 5C), maybe coming from the secondary holes within the ice blocks. The cross section of the stem is circular, elliptical or irregular shape, varying in sizes from 1 to 15 cm diameter, with the average mostly 3–5 cm, and a height of around 10 cm. Occasionally, a few completely collapse heavily.

The base is not an important part of a silt mushroom, for it is not often observed. The base is smaller than the stems and its internal structures are not as well developed as cap and stem. Actually, the base does not often develop individually and connects altogether with surrounding sediments. We have carefully observed the base and its surrounding sediments by clearing up and cutting a few important areas at which the silt mushrooms develop very well and have not found any traces formed by freezing-thawing, and nor have found any needle stick phenomenon. A large number of observation shows that the bases and their surroundings sediments are relatively homogeneous (Fig. 7). But on the contrary, the caps and the covering layer over the stems have been frozen and thawed, which forms a great deal micro-ice induced product, such as frozen fissions (Figs 5D,H and 6) and frozen bubbles.

## Results and Discussion

### The cross beddings genesis

The most important sedimentary structures are the beddings developed in the stem. The bedding could reveal the genesis of the silt mushroom to some extent. The observation shows that the vertical variations within an ice-induced silt mushroom are typically attributable to the beddings by changing grain size, composition, color, texture or internal structure. An ice-induced silt mushroom may be heterogeneous, rhythmic, gradational or deformed. Most of silt mushrooms are heterolithic (composed of different sediments) as a result of sorting into repeated interlaminations of sediment of contrasting composition or grain size carried by ice flow (e.g. fine silt, mud silt, silty mud and mud, or organic material.). A point worth emphasizing is that some silt mushrooms have well-developed cross beddings in their stems (Fig. 5D) (but most silt mushrooms have irregular beddings and well-developed deformed beddings in their stems) as similar as those formed by usual river flows. Certainly most, these cross beddings in the stems are formed by the small flows of ice flow and not by usual river flows. But, the formation way is not as same as the common cross beddings. The main evidence in any of the following points: **(1)** Their sizes are commonly larger than that of the cross beddings formed by river flows, which are generally smaller than 2–3 cm in height. Nevertheless, the heights of the cross beddings in the stems are commonly larger than 3 cm (Fig. 5D), generally 7–8 cm, mostly up to 10 cm; Up to now, we have never observed the cross beddings formed by current larger than 4 cm in height in the lower of Yellow river. **(2)** The dips of the cross beddings developed in the stems are not as same as that of the cross beddings formed by usual river flows and even have opposite directions like a funnel in the vertical sections of a stem (Fig. 5E) and commonly have deformation beddings (Fig. 5F). Otherwise, the dip direction of the lamina is opposite and the direction of the river flow is from left to right. Obviously, the dip direction of the cross beddings in the stem is not principally the same as that of the usual cross beddings formed by river flow. **(3)** We can observe two silt mushrooms with clear inclined beddings in Fig. 5D. But the lowest beddings in the left is oversteepened cross-bedding, accounting to 60°, greatly larger than that of the usual cross beddings formed by river flow, which is not more than 40° generally. Another feature is also important that the dip angles of the lamina in the stem gradually become smaller from the bottom to the top (Fig. 5D). We think that the lamina of in the stem forms by leaking of the sediment along the edge of the hole in the ice block. So as the hole is filled, the dip angle of the lamina gradually becomes smaller. **(4)** Especially, the cross beddings are circular or arc-like shapes in the horizontal sections of a stem (Fig. 8), which have been never seen in the cross beddings formed by usual river flows. This kind of bedding is the most common. Concave shape suggests that the irregular silt lamina is also formed by leaking from the edge of the hole in the ice block. Nevertheless, it can’t be ruled out that it is overlapped by slight deformation; **(5)** The continuity of the lamina of the cross beddings in the stem is mostly not as good as that of the cross beddings formed by river flows.

### Associated ice induced sedimentary structures

The most common sedimentary structures which can be associated tightly with ice-induced silt mushrooms, are found in similar locations. These structures include; ice crystal imprints, frozen fissions, ice tool marks, ice bubble marks, ice freezing cracks, ice-induced silt volcanoes^8^,^9^,^10^,^11^ and so on (more than 20 kinds; see the appendix). The boundary of the formation of these sedimentary structures is strictly related to the ice block melt boundary (Fig. 5A). The ice-induced silt mushroom only occurs during the transition from winter to spring. As with the associated structures, the required time period for formation, and the severity of the conditions needed for this formation, means the shaping of silt mushrooms must be in relation to the ice process. Ice-induced silt mushrooms is exposed in groups or individually on the channel bar and\or point bar. Their probability of emergence is commonly higher at the top and front of the channel and\or point bar, where the sandwich texture may be easy formed, because the ice block can be easy to be washed onto there. Their formation is strictly restricted within the ice block, formed by ice flows. They may be truncated by an ice-induced, laterally depressed, collapsed wall (Fig. 5A).

### Results of the experiment

Based on field observation and experiments (Fig. 9), it was proved that the melting methods of the ice block are very interesting. When an ice block has been polluted by silt and mud, especially on its surface, its melting rate is not the same in different places: the place polluted by silt and mud will melt relatively fast and the pure areas will melt relatively slowly, so this results in the ice block changing to have a different rate in a different position. The place polluted by silt and mud melts fast to form a hole within the ice block. This is discussed in the experiment and theory analysis section. The research team believes there are some individually occurring silt mushrooms, but there is just one type of silt mushrooms in the group.

### Preservation of the silt moshroom

If the ice induced silt mushroom has scientific significance or not, it is the key that can it be preserved. Undoubtedly, some of the silt mushroom structure may be eroded by late geological processes as other sedimentary structures as beddings, but some of the silt mushroom-like structure may be preserved. Based on observation the silt mushroom-like structure has the same consolidation as other sediments or other sedimentary structures, so they can be preserved in the sedimentary successions as same as other sedimentary structures. In any river environment, some of sediments or some of sedimentary structures are eroded and the other can be preserved. This rule can be fitted to any river. In any river sedimentary successions reaction surface is easy to be observed and this tells us that erosion processes have happened and some sediments or some sedimentary structures has been positively eroded. The important feature of river deposition is that part of sediments and sedimentary structures preserve and part of sediments and sedimentary structures is eroded. Otherwise, because the lower of Yellow river locates in the relatively arid district the wind geology is intensive in transition of winter to spring the some surface of the point bar and channel bar is often overlain abruptly by aelian sand. This are favorable for being preservation of them for the silt mushroom-like structure is buried by aelian sand. In 1997, we observed a stretch of silt mushrooms exposed by wind erosion in a point bar, which was formed in 1996. This suggests that the silt mushrooms may be preserved as same as other usual sedimentary structures formed by river flow.

We have carefully observed the base or stem basement by clearing up and cutting a few important study areas at which the silt mushrooms develop very well and have not found any traces formed by freezing-thawing, and nor have found the needle stick. A large number of observation shows that the stem bases and their surroundings are relatively homogeneous (Fig. 8). But on the contrary, the caps and the covering layer over the stems have freezing-thawing action, which forms a great deal micro-ice induced product, such as frozen fissions (Fig. 6) and frozen bubbles. Nevertheless, freezing-thawing action or freezing-thawing cycle is a common geological process in the river bank in the regimes of the middle to high altitudes^47^,^48^,^49^,^50^,^51^,^52^,^53^.

## Mechanism and model of the silt mushroom formation

### Mechanism of the silt mushroom formation

In order to distinguish if the silt mushroom is formed by needle stick or by covering layer collapse, the key issue is that we should make sure that if the stem rise from the ground or the ground collapse. All the evidence suggests that the stem does not rise and the ground does collapse, so the silt mushroom does not form by needle stick and does form by the ground collapse, all the evidences are as follows: **(1)** We directly observe *in situ* that the covering layer on the stem collapse. **(2)** We can make the covering layer to collapse to form a silt mushroom in the field. **(3)** All the silt mushrooms develop within an ice melt depression; **(4)** The experiment provides strong evidence that the silt mushroom forms really by the ice block melting and the covering layer to collapse and not by freezing and thawing.

The repeated freeze-thaw cycle may really reform or erode the sediment surface textures^47^,^48^,^49^,^50^,^51^, especially the bank surface^52^,^53^, but it can’t form the silt mushroom documented in this paper. It may be that only very-strong and elaborate ice process form the silt mushrooms because in these cases greater amounts of energy are released and the freeze-thaw cycle does not have so great energy and so elaborate action to form so numerous nearly round silt mushroom.

The formation mechanisms of ice-induced silt mushrooms is very important and worthy of study because this new phenomenon has many applications for the academic field of sedimentary environment analysis. In terms of the ice-induced silt mushroom’s genesis, one can infer this is due to the unusual flowing processes of the Yellow River; these occur from the end of the winter to the beginning of the spring. As mentioned above, the ice-induced silt mushrooms are associated with many other ice-induced sedimentary structures. Like these, the distribution boundary of silt mushrooms is strictly controlled by the ice block melt boundary (Fig. 5A). Therefore, the main cause of the silt mushroom formation can only be attributed to the natural ice process. However, the origin of ice-induced silt mushrooms on the Yellow River delta is completely unique. They result from the existing hydrological, climatic and hydrodynamic conditions.

As we know, the Yellow River is unique in the world. Firstly, because the Yellow River located in the relatively dry region of north China; the total water yield is insufficient and during 1990s the Yellow River was often in zero flow. Secondly, for its tremendous sediment load and a very small gradient in the lower, this is especially the case in the section from Binzhou Bridge to the estuary; the lower of the Yellow River becomes narrower and shallower, resulting in frequent flood. Thirdly, the lower of the Yellow river is in a warm temperate zone with four distinct seasons. Every year when it is getting cold in winter, the Yellow River freezes and the mains are obstructed by ice blocks, which form ice flow. So, during the transition from winter to spring, a lot of ice blocks are washed onto the channel bars and point bars to form an unusual sedimentary environment. This provides particularly favorable natural conditions for the formation of sedimentary structures. This includes silt mushrooms.

Additionally, the riverbed’s fine texture is both texturally and compositionally important to the formation of the silt mushroom. The uniqueness of the ice layers built upon the mud and silt, and the river’s fine-grained sediment load. These are generated by the Yellow River’s flow and make this a singular phenomenon. Of all the conditions observed, it is the unusual hydrological phenomenon generated by the Yellow River’s ice-run that can be attributed to formation of ice-induced silt mushrooms. These hydrological actions collectively play a key role in the formation of ice-induced silt mushrooms, as ice-melting creates the honeycomb-like holes; these holes are later filled with silt. This thesis, therefore, proposes that there is a formation model of the Yellow River delta silt mushroom that relates to the ice layer formation and melting process.

It is really that there are a lot of needle ice blocks on the channel bar or point bar in the lower of Yellow river. The needle ice in the lower of the Yellow river in thicken is extremely compatible with that of Outcalt (1971)^48^, actually being about 10 cm, and is a little compatible in the cross diameters of the ‘vertical filaments of ice, larger than 1 mm in cross section of ice filaments and being reaching to 2–3 mm. Another interesting phenomenon is that there are a great deal small tube-like holes between the ice filaments. A great deal of silt can leak along these small tube-like holes (marked by blue arrow). But we should note that there is often a space between the needle ice block and the sedimentary surface. The sedimentary surface has clear wave ripples and ice frozen fissures^51^ and this shows that it had not been badly influenced by freezing and thawing action. In a word, this ice block does not has “root” and is washed by ice flow from other place of the upper of Yellow river. The detailed observations reveals that the ice block with needle textures comes most possibly from the external edge where it is easy to develop needle texture (Fig. 10) in the ice block for constant water seepage from the levee. This is suggested by the stacked ice blocks and silt layers like a sandwich and the lots of large scale ice tool marks, about 1 meter in width and more than 500 meters in length.

It is important to ensure that, if the stem rises from the ground or the ground collapses, the silt mushroom is formed. This can either be through the needle stick or the collapse of the covering layer. All the evidences suggest that the stem does not rise from the underground due to freezing and thawing. Instead, the ground does collapse and this forms the silt mushroom, rather than the needle stick. The evidence is as follows: **(1)** Direct observation *in situ* that the covering layer on the stem collapses. **(2)** One could make the covering layer collapse to form a manmade silt mushroom that is the same as a natural one **(3)** All the silt mushrooms, whether in groups or individual, develop within an ice melt depression (Fig. 5A). Therefore, the genesis of the silt mushroom is closely related to the ice block thawing. **(4)** The experiment has been repeated to show that the silt mushroom can form through the thawing of an ice block and the collapse of a covering layer on the stem (Fig. 9).

### Model of silt mushroom formation

A tentative model for the formation of the ice-induced silt mushroom is proposed here. There are four stages of the formation process for the ice-induced silt mushroom that can be clarified: **(1)** The ice block is washed onto the channel bar and/or point bar; this takes place during the process of ice flow in the transition between winter and spring (Fig. 11A); the mushroom’s surface or internal has been polluted by silt and mud that is distributed unevenly to form clouds of silt and mud; **(2)** when the sun shines on the ice block, the sections that have silt and mud thaw easily and so the ice block only partially melts; therein, a lot of holes appear, resulting in the formation of a honeycomb-shaped holes, best observed in some of the small ice blocks (Fig. 11B) or simple holes within an ice block. It is highly possible that these relatively bigger holes may be enlarged later by cool water that is near freezing temperature. **(3)** When another ice flow happens, the channel bar and\or point bar will be submerged again. Those honeycomb-shaped holes or simple holes will be covered by the mud and silt, sometimes including the ice block, brought on by floods (Fig. 11C). **(4)** With the temperature increasing during the transition between winter and spring, the ice block, with its honeycomb-like holes, melts completely; the subsequent silt layer that cover the ice block collapses. This finally results in a complete silt mushroom formation (Fig. 11D). The formation mechanism described above has been satisfactorily explained by the team’s model. The above formation process of silt mushrooms has already been confirmed to some extent by our simple experiments.

## Conclution

Further academic significance from our discovery of ice-induced silt mushrooms, may be as follows:

1. Ice-induced silt mushroom is a new type of sedimentary structure; its discovery may be significant to complete basic theories upon sedimentology. This achievement is significant not only in sedimentology, but also in geography, geological engineering, hydraulics and fluviology.
2. Ice-induced silt mushrooms could be of value to environment analysis; the phenomenon requires an exposed environment, with the ice flows happening frequently. The frequent ice flows in combination with a great deal of mud and silt, may be responsible for the ice-induced silt mushrooms visible.
3. Extremely strict conditions are needed for the formation of ice-induced silt mushrooms, such as specific circumstances within an areas; hydrology, climate, sedimentary environment and fine material quality. The ice block washed onto the channel and point bar plays a fundamental role in the formation of ice-induced silt mushrooms.
4. The silt mushrooms present in the lower of Yellow river are extremely similar to those produced experimentally by simulated ice block melt. The apparent success of the study of the lower of Yellow river led to a search for similar structures in other similar modern sediments or ancient rocks in the world. The discovery of ice-induced silt mushrooms, may contribute to furthering our understandings of similar sedimentary structures in rocks, questioning their genesis and furthering similar structures sedimentary environment analyses.

## Methods

This investigation is mainly restricted to field observations and indoor experiments as follows:

### (1) Field observations

Ice-induced sedimentary structures, including silt mushrooms, are discussed in terms of their geological features; field observations are therefore highly important. The concept of a silt mushroom is introduced and the conditions of their formation is also presented. A basic aim of fieldwork on silt mushrooms should be to observe them in detail and describe them accurately and concisely.

Since the lower of the Yellow River is often seriously flooded by ice–flow, field observation can be dangerous and it is imperative that participants select a safe and suitable time and location to conduct their work. Where possible, fieldwork should aim to measure and record some typical silt mushrooms on the channel bar and/or point bar and to select and to document typical ones judged from preliminary field observation. This enables local variability to be distinguished from any regional variability and ensures that common features are seen from a proper perspective. Participants should make sure to take measurements, statistics and photographs of the silt mushrooms where this is possible. Just because of this, the field observation may be too rather arbitrary to select. Experience over twenty years informs us that the ideal location to investigate silt mushrooms is the section of the river from Binzhou Bridge in Lijin to the estuary. The distance covered is approximately sixty kilometers long. The section near Shengli Yellow River bridge is especially convenient to reach and has a lot of channel bars and point bars that is easy to approach, especial the ice blocks are easy to be washed onto the channel bars and point bars to form a special texture like a sandwich for having a lot of curves that is favorable for ice block to be washed onto the channel bars and point bars to form sandwiches that is of central importance in formation of silt mushrooms.

**Figure 2:**
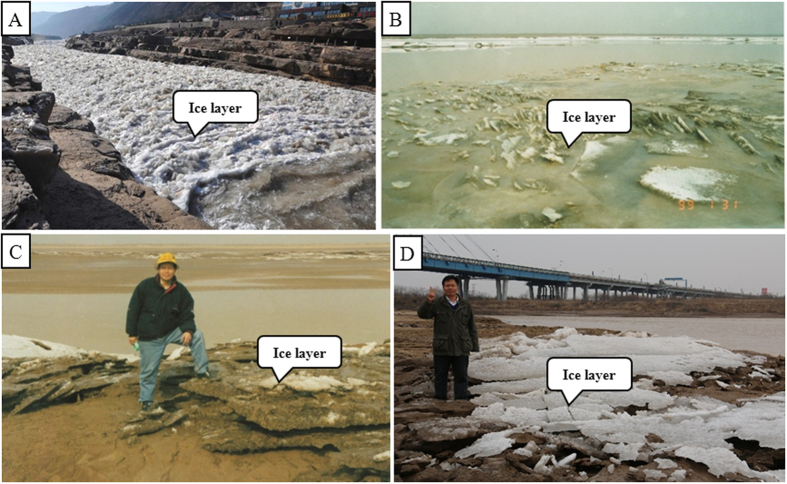
Ice flows in Yellow river. (**A**) Ice flow near Hukou waterfall in the upper of Yellow River, Shanxi Province, from the internet; (**B**) Ice flow near Dongying in the lower of the Yellow River; The ice block had been washed onto the point bar and this made the ice blocks being inclined sharply. (**C**,**D**) Ice blocks interlaminated with muds and silts are washed onto the channel bar to form sandwich-like texture, near Dongying, Shandong, during a few ice flow surges. (The person appearing in this figure is the co-first author Professor Zhong Jianhua. Photos taken by Dr. Ni Liangtian).

**Figure 5:**
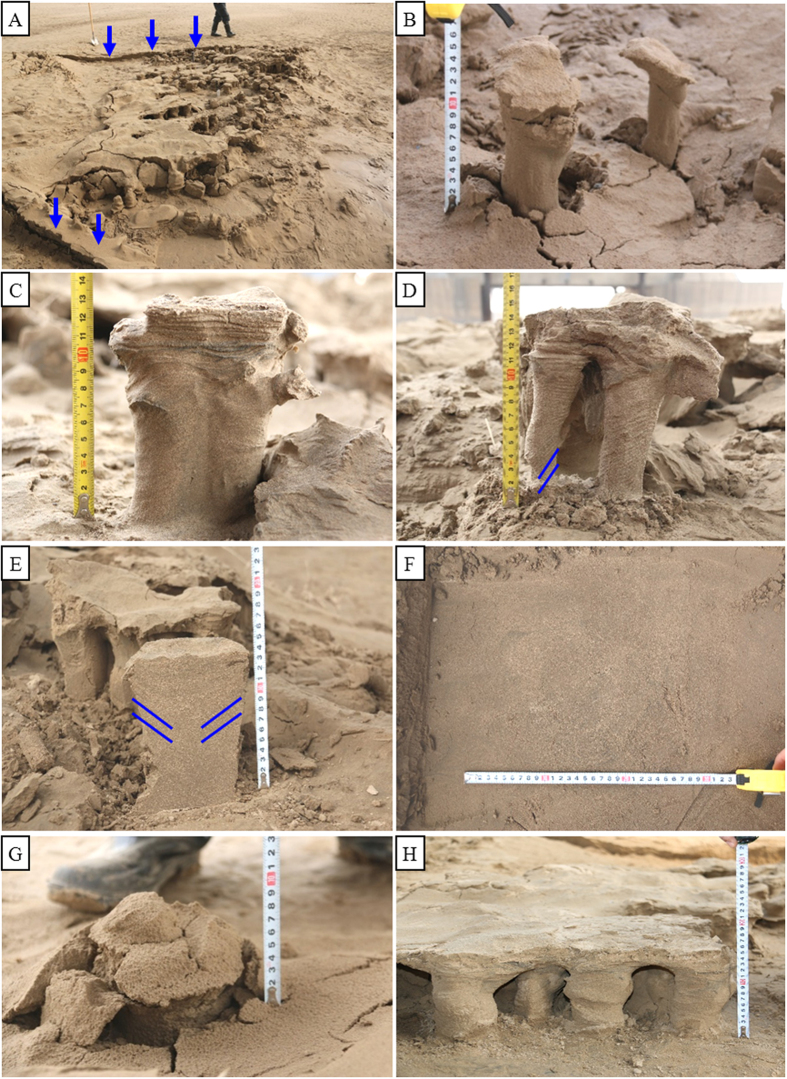
Appearances of typical ice-induced silt mushrooms. (**A**) A group of silt mushrooms with a single cap and unified caps, around 50–60 examples (a), developed within a melted ice block. Note there are a few ice melt ridges (indicated by blue arrows) around the silt mushrooms. (**B**) Two irregular silt mushrooms with gently dipping caps surrounded by a collapsed silt layer. (**C**) A typical silt mushroom seems to be compounded by two organizational forms. The top have clear horizontal beddings and is not perfect. There is some small deformation beddings between the top and the stem. (**D**) Two silt mushrooms compounded with a common cap, obviously having a well-developed cross bedding. Their caps is not well developed due to collapsing. The dip angles of beddings (sighed by blue lines) in the lower stem is relatively sharp, reaching about 60°. (**E**) A vertical cross section of a mushroom with special cross beddings of opposite dip directions (sighed by blue lines), like a imperfect funnel, suggesting that the silt’s filling is from two directions. (**F**) An example of relatively regular, circular beddings at the bottom center of the Photo (**E**). (**G**) Tow silt mushrooms in the forming process. The ice layer has melted partly and the silt mushroom has broken through the silt layer. (**H**) The ice block has melted completely and the mushrooms stems stand closely one by one. The silt layer upon the stems has not collapsed, making the caps connected and resulting in formation of some chambers. (All photos taken by Dr. Ni Liangtian in the lower of the Yellow River).

**Figure 6:**
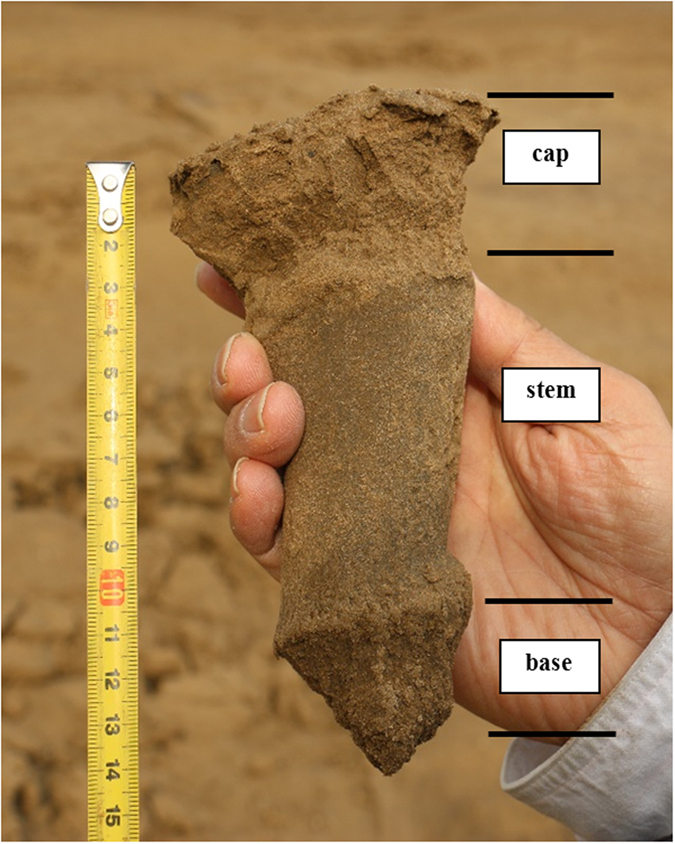
An Ice-induced silt mushroom: its three constituent parts: cap, stem and base. (Photos taken by Dr. Ni Liangtian in the lower of the Yellow River).

**Figure 7:**
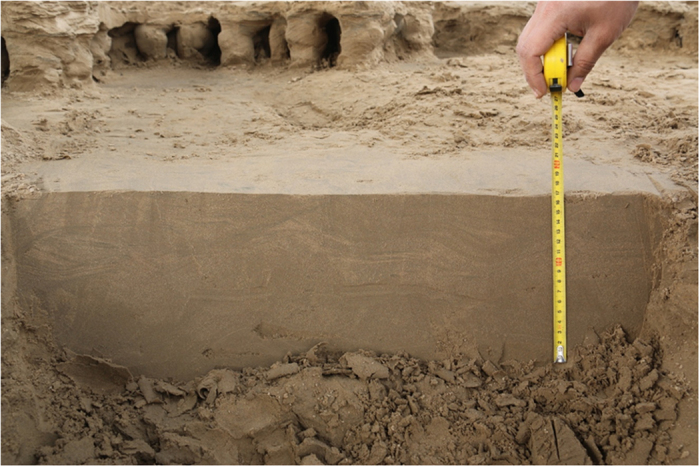
The relatively homogeneous and continuous beddings. It is difficult to observe to having been reformed by freezing and thawing. This phenomenon suggests the silt mushroom is not formed by freezing and thawing.

**Figure 8:**
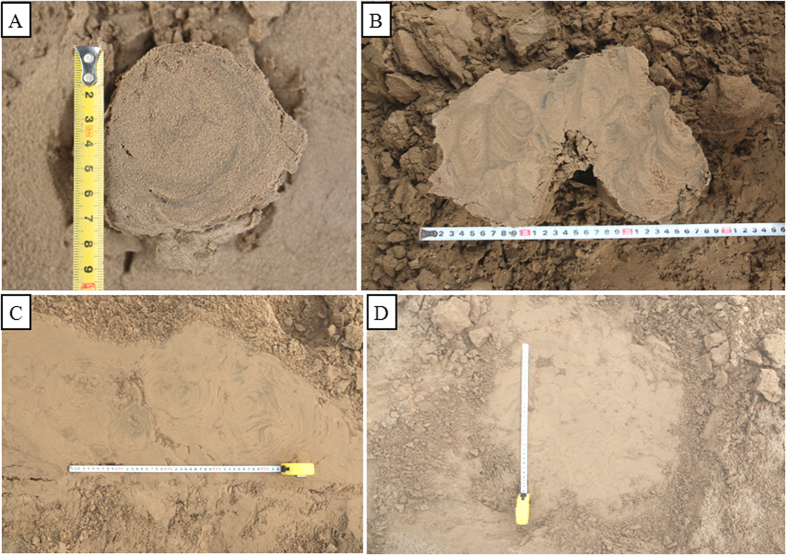
Closed-up top view of a cross section in the stems and bases. (**A**) A closed-up top view of a cross section in the middle of a stem. The discontinuity circular beddings are apparent and this reveals the lamina is arc-shaped. (**B**) A close-up top view of a compound silt mushroom. On the plane section of a stem, we can observe the arc-like lamina, which is displayed by arc-lamina and other complicated lamina. (**C**) A close-up top view near the basement of the stems. On its plane section, we can observe 5–6 bases with well-developed circular lamina and other curve lamina, which is formed by the funnel-like bedding in vertical section. (**D**) A close-up top view near the basements of the stems. On its plane section, we can observe more than 20 bases with well-developed circular lamina (marked by blue arrows), which is formed by the funnel-like bedding in vertical section.

Field investigation may not go to schedule as a result of not being exposed of the channel bars and point bars for ice flowing or overlain by ice layer for too cold. An effort should always therefore be made to wait for the proper opportunity and to secure the proper location. Once the proper location has been selected, participants should first clear the site carefully and then make a detailed observation including careful description, strict measurements and photographs. The author hereby refers to a working method that is three-dimensional and carefully thought out: **(1)** clearing up of a field site and getting a serial of vertical sections by cutting some silt mushrooms and their bases (Figs 7 and 8). It is important to dissect the silt mushrooms and their bases extremely carefully with a long sharp knife, generally from top to bottom and in two perpendicular directions (Fig. 5E,F); **(2)** it is also extremely helpful to photograph as many silt mushrooms and their bases as possible, from a series of positions and at different distances. Fortunately, this is very easy to do in the study area.

### (2) Indoor experiments

In order to re-create the formation process of a silt mushroom, it can be helpful to design a simple laboratory experiment. The experiment has two aspects: **(1)** the formation process of the ice-hole within an ice block; **(2)** the formation process of the ice-induced silt mushroom.

**Figure 9:**
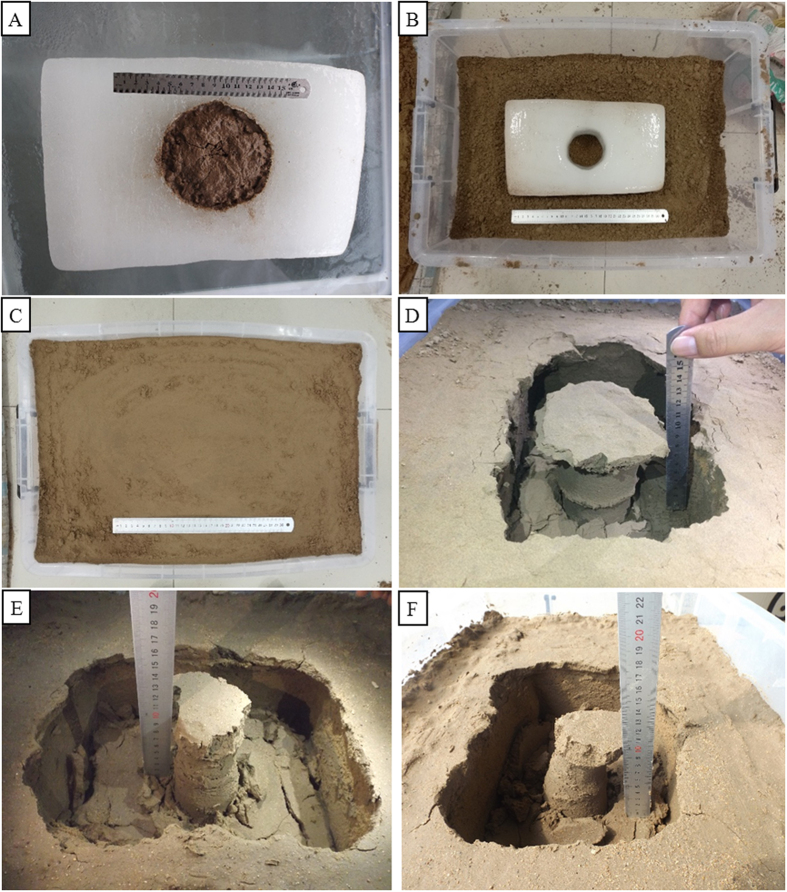
Needle ice blocks. (**A**) A needle ice block overlain by a thin silt layer. Note the ice block is overhanging and the sediment surface under the ice block is not of being frozen and thawed. (**B**) The typical needle texture and there are small tubes in the ice block, along which a great deal silt is leaking.

**Figure 10:**
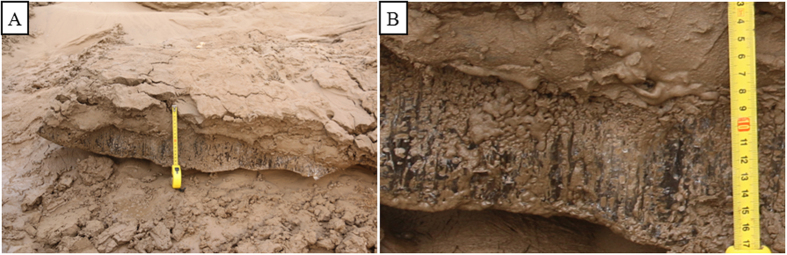
A simple physical experiment for the formation of ice-induced silt mushrooms. (**A**) A ice block polluted by a lump of silt and mud where the ice melts fast. (**B**) A ice block with a hole that is bigger in the upper and smaller in the lower. (**C**) The ice block has been covered by silt. (**D**) The ice block has been melt completely and the silt mushroom with smooth stem has formed. (**E**,**F**) A silt mushroom with some anti-steps or flat fissures in its stem due to the friction and traction of the collapsed cap because of the ice hole shape that is smaller in the upper and bigger in the lower. (**E**) Front view and (**F**) side view.

**Figure 11:**
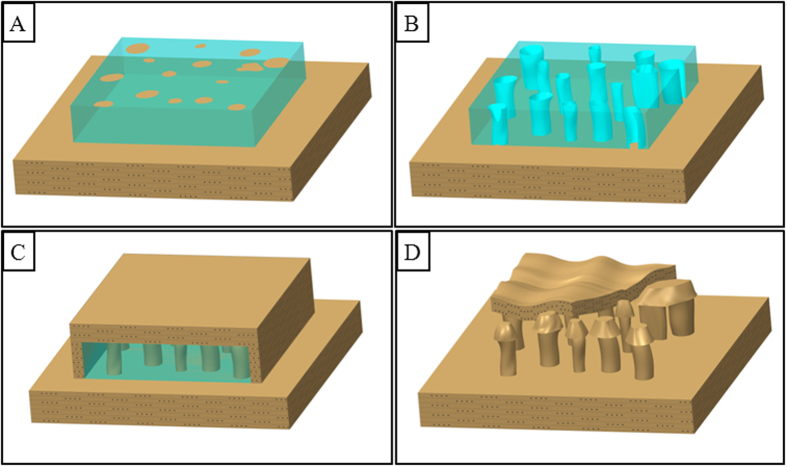
A tentative 3D Model for the formation mechanism of ice-induced silt mushrooms. (**A**) A ice block with some lumps of silt and mud is washed onto the bar. (**B**) The ice block has been melt partially to form numerous holes in the places polluted by lumps of silt and mud. (**C**) The ice block is covered by a layer of silt and mud washed by next flood. (**D**) The ice block has been melt completely which resulting the formation of silt mushrooms.

Simple physical experiments can be devised to recreate the formation process of a silt mushroom. The first thing to do is to prepare a freezer. Firstly, the authors purchased a small ice tank (400 cm width, 780 cm length and 830 cm in height) and then a smaller plastic tank (57 cm length, 37 cm width and 28 cm height). The authors then gathered around 100 kilograms of loose silt from the Yellow River. The experiment followed a number of steps: **(1)** Preparing an ice tank and plastic box. Switch on the ice-tank and put the plastic tank into it. Fill the tank with water to the level of 10 cm. After all the water is frozen, take the plastic tank out of the ice tank and cut the ice block to be 30 length, 20 cm width and 10 cm thicken and put a lump of silts on the surface of the ice block (Fig. 9A); **(2)** Wrapping its bottom and sides with a cotton quilt to keep warm in order to prevent the ice-layer from melting too fast, expose the ice layer to the sun to the complete formation of a hole (Fig. 10B). **(3)** Put the ice layer into the ice tank after the holes have formed (Fig. 9B), filling it with moderate ice water to about 20 cm high; **(4)** Pour moderate silt into a plastic box and stir. Slowly add the mixture of water and silt every minute with a spool into the plastic tank. Repeat the process until the silt levels 10 cm in height. Meanwhile, pat the water surface with your hands in order to make the silt closely packed. **(5)** Leave the sediments to freeze for one night; **(6)** Take the plastic tank out of the ice tank and wait until the ice layer has completely melted to observe the silt mushroom that has formed (Fig. 9C–F). The process may be repeated by changing the experimental conditions such as changing the added amount of silt, changing interval times each time and so on. In a word, in order to better reappear the formation process of silt mushrooms. The good experimental result has been obtained. Since our experiment was about 1–2 days long, whereas the actual formation process might be one week to two weeks long in the lower of Yellow river, from the Binzhou Bridge to estuary, the time ratio t’ is reduced to about 1/7 and the experimental size is also reduced largely. So there are some differences between the both and the experimental result is only provided to refer to.

## Additional Information

**How to cite this article**: Jianhua, Z. *et al*. A New Unusual Ice-induced Sedimentary Structure: the Silt Mushroom. *Sci. Rep.* **6**, 36945; doi: 10.1038/srep36945 (2016).

**Publisher’s note:** Springer Nature remains neutral with regard to jurisdictional claims in published maps and institutional affiliations.

## Supplementary Material

Supplementary Information

Supplementary Information

## Figures and Tables

**Figure 1:**
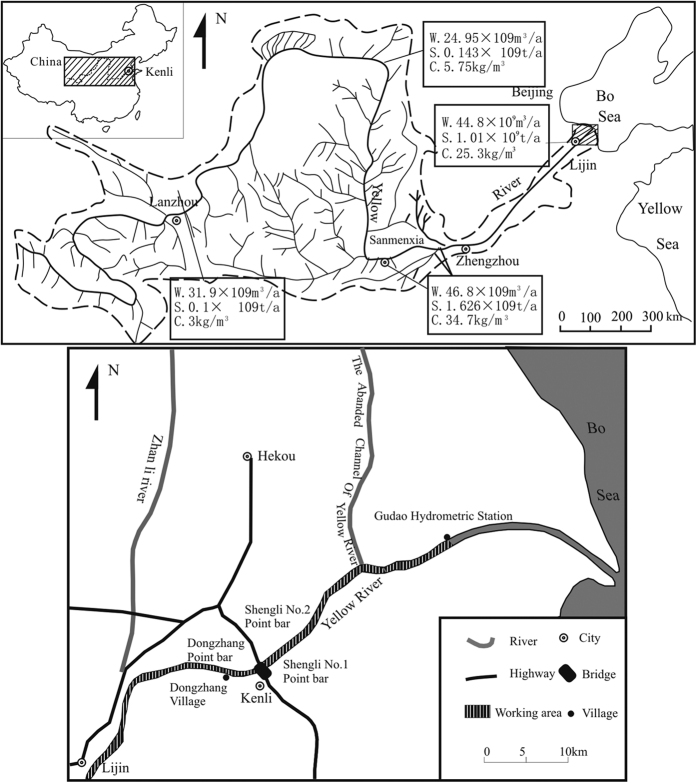
Map of the Yellow River valley and the location of the study area (after Zhong J. H. *et al*.^14^, with permission from Elsevier). Legend information; W: discharge, S: sediment yield, C: sediment load.

**Figure 3:**
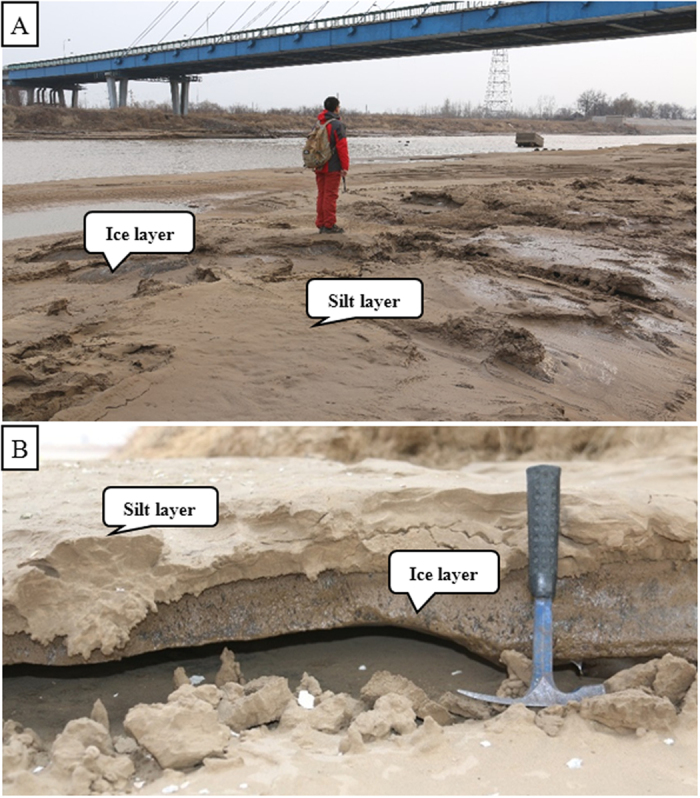
Ice-silt sandwichs. (**A**) Note that the channel bar is covered with thin ice layer and thin silt layer. All the ice blocks occur in the form of fragments; The background is Shengli Bridge and its channel. (**B**) An ice-silt inter-bed, the layers are shown sandwiched together and the ice block has needle textures. Note there is a relatively large space between the needle ice block and the sedimentary surface, this reveals that the needle ice block is not formed *in situ* and comes from the upper flow (Photos taken by Dr. Ni Liangtian in the lower of the Yellow River).

**Figure 4:**
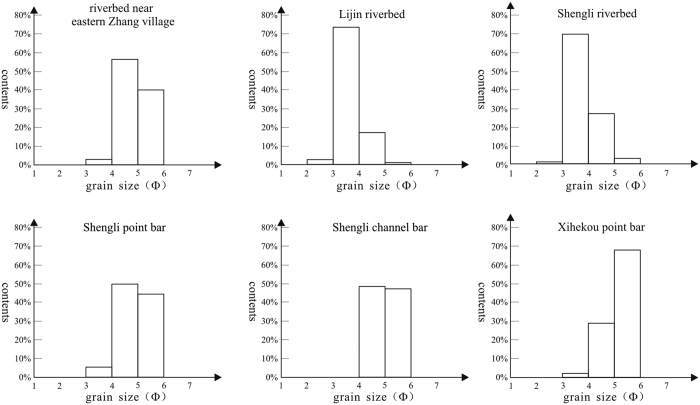
Grain size distributions of river sediments. (After Zhong J. H. *et al*.^14^, with permission from Elsevier).
