# Supplementary material for: A New Unusual Ice-induced Sedimentary Structure: the Silt Mushroom
Source: Sci Rep. 2016 Nov 11;6:36945. doi: 10.1038/srep36945 (PMC5105128; doi:10.1038/srep36945)
Supplement: Supplementary Information [file srep36945-s1.pdf]

A New Unusual Ice-induced Sedimentary Structure:

the Silt Mushroom

Zhong Jianhua, Ni Liangtian*, Sun Ningliang, Liu Chuang,
Hao Bing, Cao Mengchun, Chen xin, Luo Ke, Liu Shengxin, Huang Leitong, Yang Guanqun,
Wang Shaojie, Su Feifei, He Xuejing, Xue Yanqiu

School of Geoscience, China University of Petroleum, Qingdao 266580, China

A New Unusual Ice-induced Sedimentary Structure:

Chinese flower-bun-like structures

Appendix Ⅰ

Supporting information

Accompanied or associated ice-induced and ice water sedimentary structures with silt mushroom

(All photos were taken by Ph. D. Ni Liangtian and all the photos were taken in the lower course of Yellow river; The numbers on the tape n all the photos indicate decimeters.)

In order to help the readers to more easily and exactly understand the silt mushroom and its genesis, it is necessary to provide some photos that have the important information of the formation background of silt mushrooms and accompanied or associated ice-induced and ice water sedimentary structures with silt mushrooms. Certainly most, most of them (at least over 18 kinds from the second to the nineteenth) are of new sedimentary structures that have never been documented up to now in any books or magazines. All the photos herein were taken from 1996 to the present.

* These authors contributed equally to this work. Correspondence for materials should be addressed to Ph. D. Ni Liangtian.
E-mail:382938098@qq.com

1. Background: Ice and silt layer sandwiches

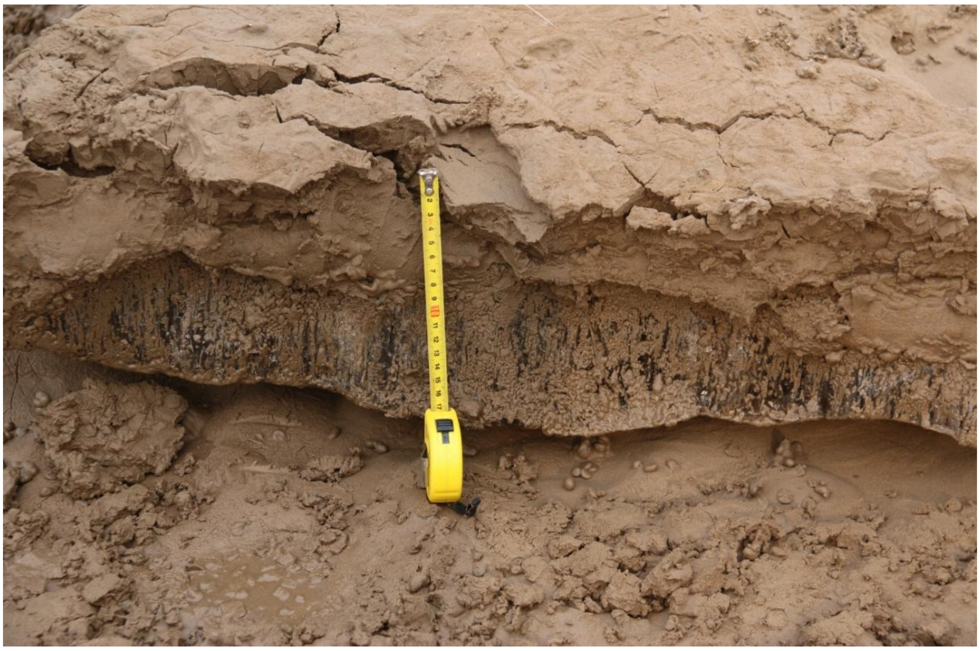

An interbedded silt and needle ice layers. The two components are very clearly different: the transparent ice layer has vertical striations and the silt layer is an earthy yellow color. A thin ice block was swashed onto the channel bar and then covered by a thin layer of silt, resulting in the formation of a sandwich-like texture. Without question, these needle ice textures is formed by freezing and thawing, but it is not formed in situ and is swashed by ice flow from the upper course of the Yellow river.

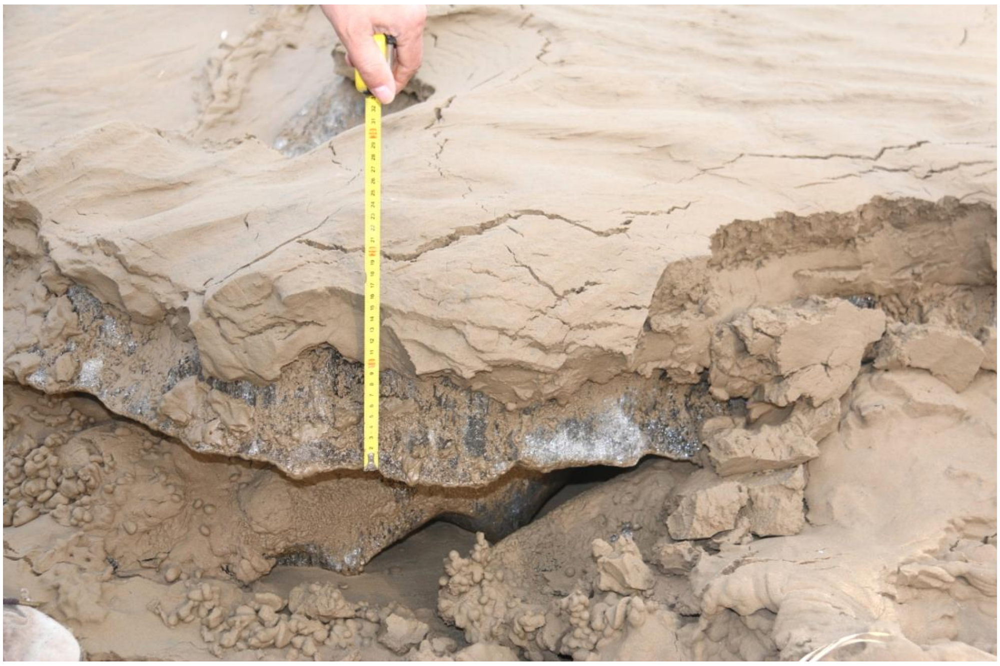

An interbedded silt and needle ice layer: The two components are very clearly different: the ice layer is white or transparent and the silt layer is an earthy yellow with lateral fissures. Two thin ice blocks were washed onto the channel bar and then covered by a thin silt layer, resulting in the formation a sandwich.

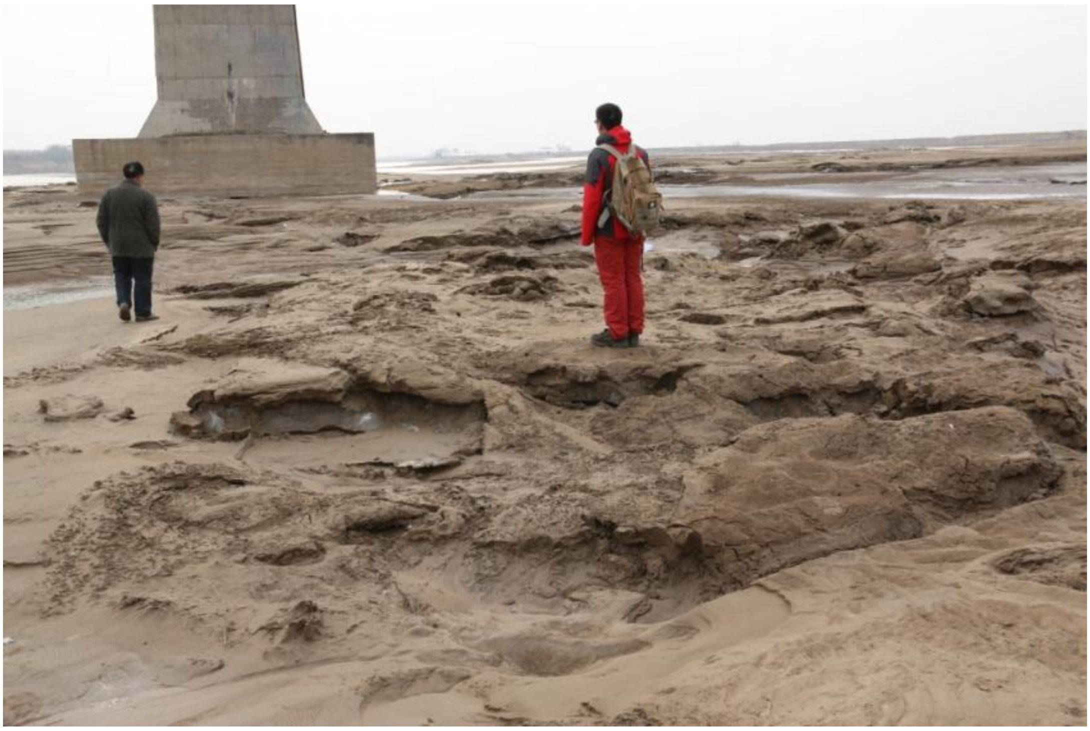

An interbedded silt and ice layer: The two components are very clearly different: Many thin fragments of ice were washed onto the channel bar and then covered by a thin layer of silt, resulting in the formation of a large, discontinuous sandwich.

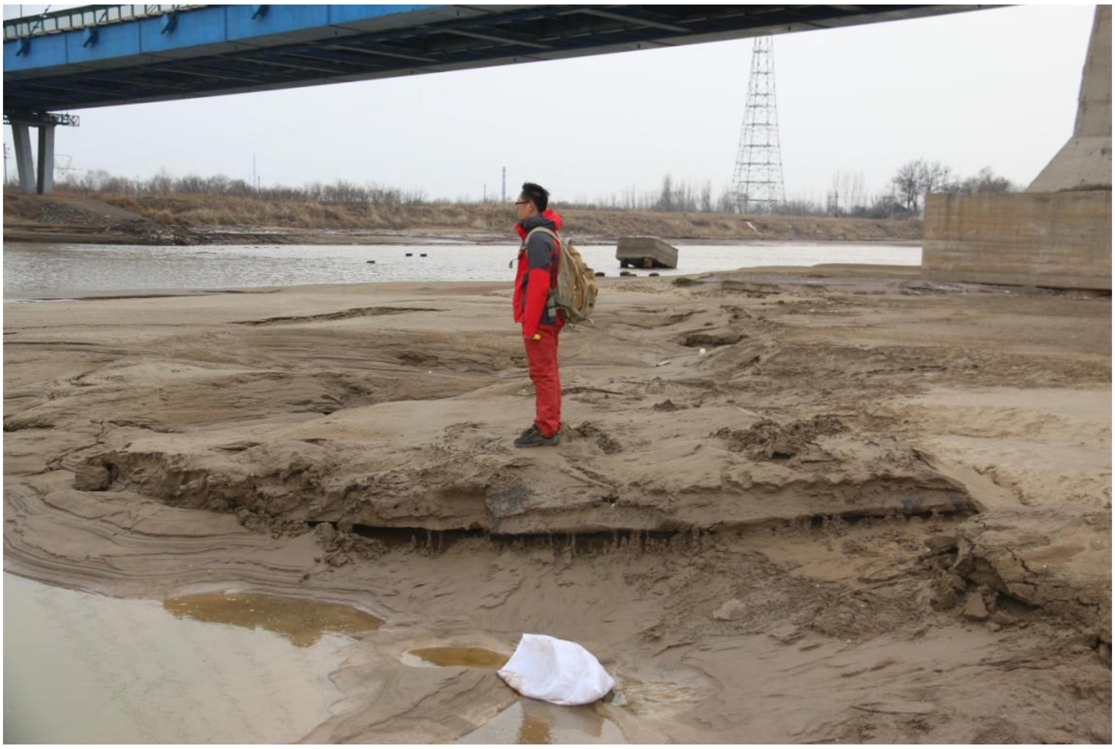

An interbedded silt and ice layer: The two components are very clearly different: Many thin fragments of ice were washed onto the channel bar and then covered by a thin layer of silt, resulting in the formation of a large, discontinuous sandwich.

The person appearing in the figure(left one) is the co-first author Professor Zhong Jianhua.

The person appearing in the figure(right one) is the co-author Sun Ningliang.

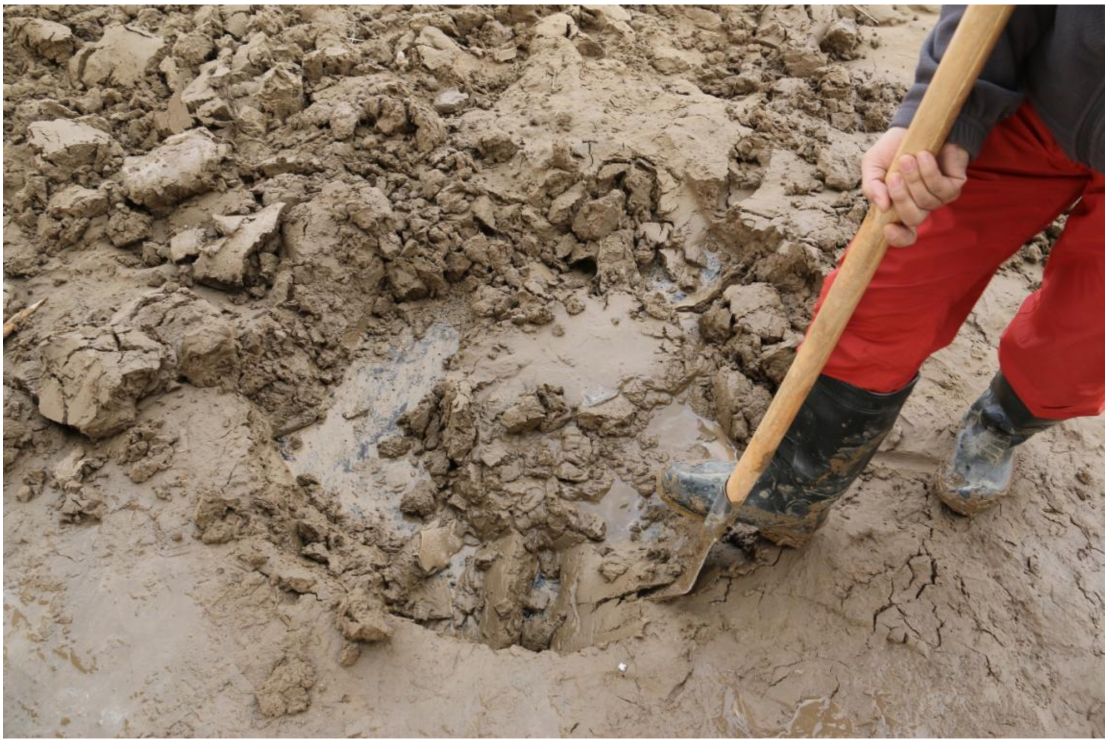

The buried ice layer was dug out by a Ph. D. student.

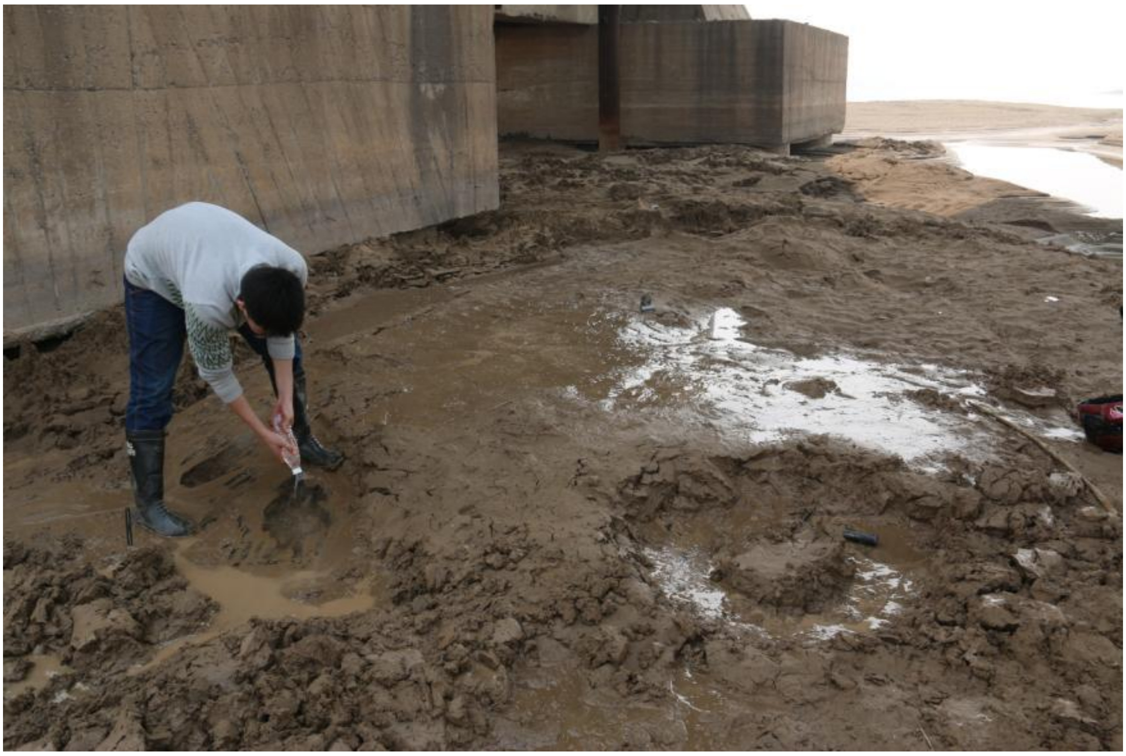

The dug out ice layer was cleaned.

The person appearing in the figure is the co-author Liu Chuang.

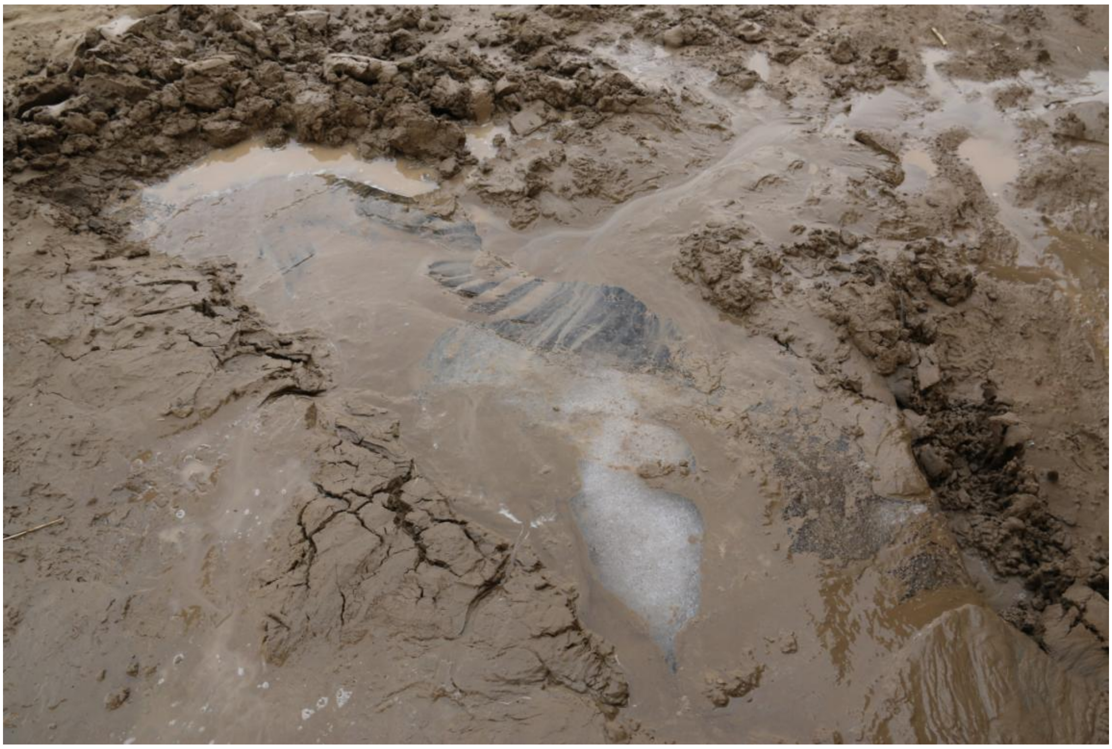

A dug out needle ice layer, which was melting and had a lot of small holes within the ice block.

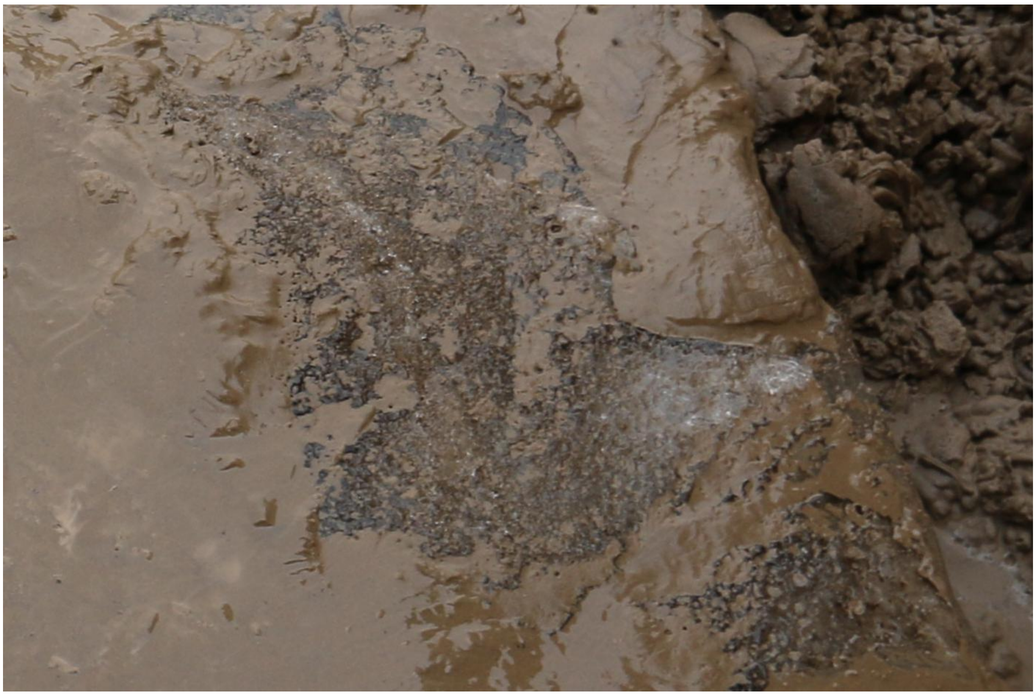

A lot of small holes are clearly observed. The water and the mud and silt paste has leaked from these holes.

2. Chinese flower-like-bun structures

This kind of ice induced sedimentary structure has the same genesis with ice induced silt mushroom. The Chinese flower-bun-like structures are formed by the filling of mud and silt in the holes within the ice block and then the ice block melt and the covers overlain the ice block collapse.

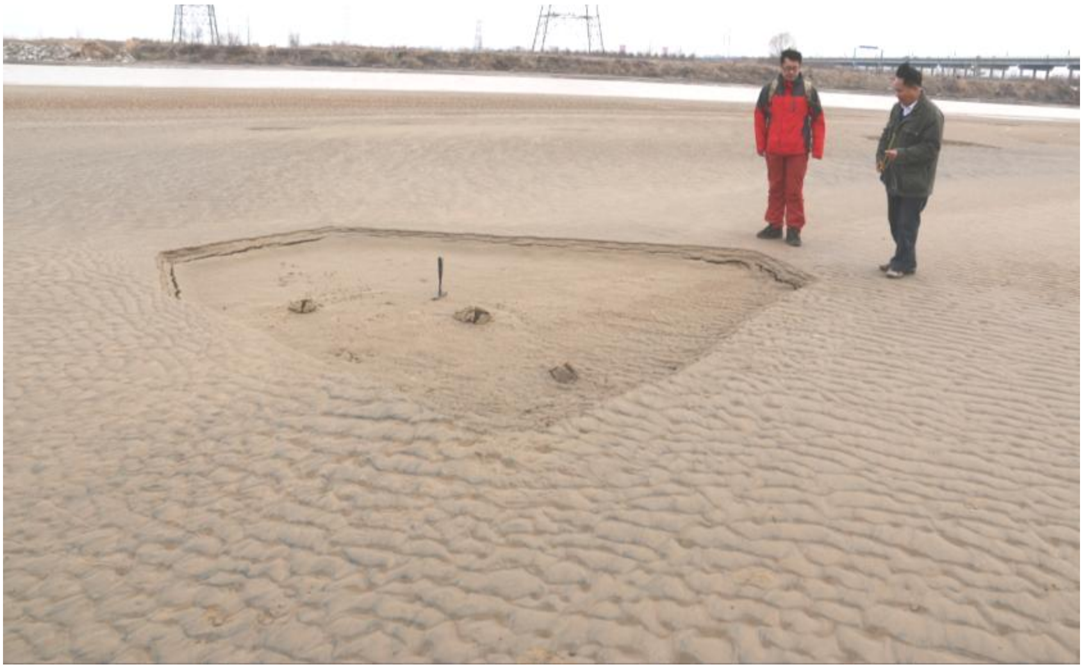

Three Chinese flower-bun-like structures developed in an hexagonal ice melt depression.
The person appearing in the figure(right one) is the co-first author Professor Zhong Jianhua.
The person appearing in the figure(left one) is the co-author Sun Ningliang.

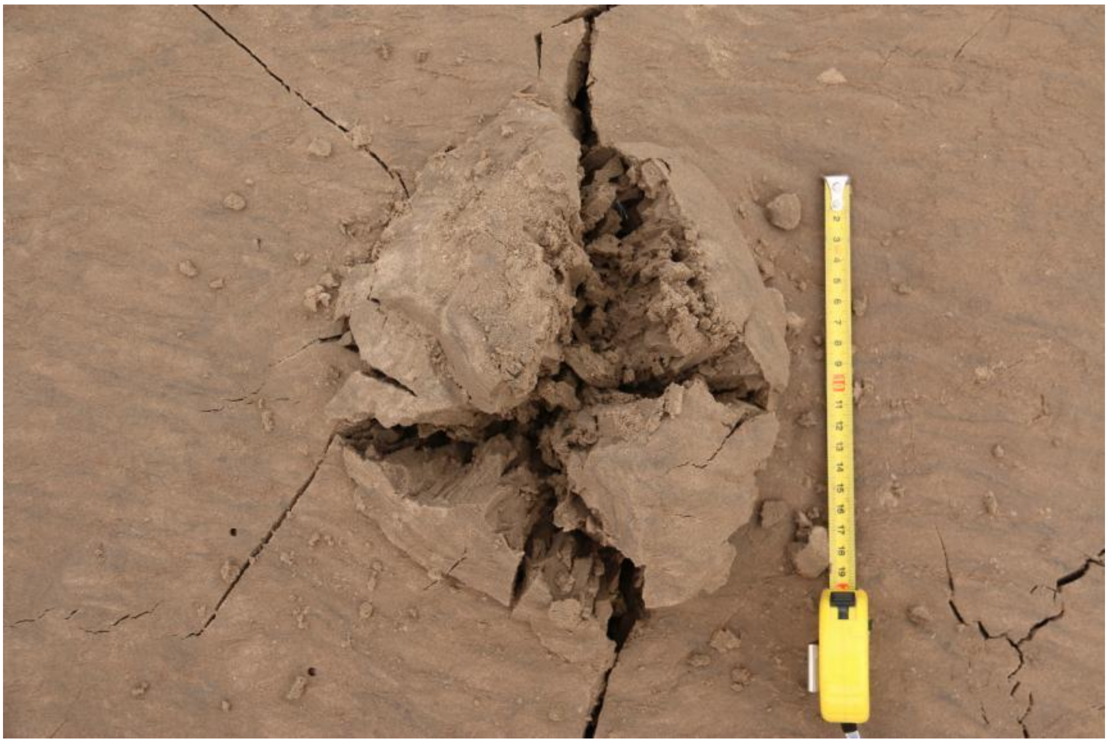

A small typical Chinese flower-bun-like structure in the above depression. There are four lenient fissures that cut it into fore parts and a great deal of frozen fissions within it.

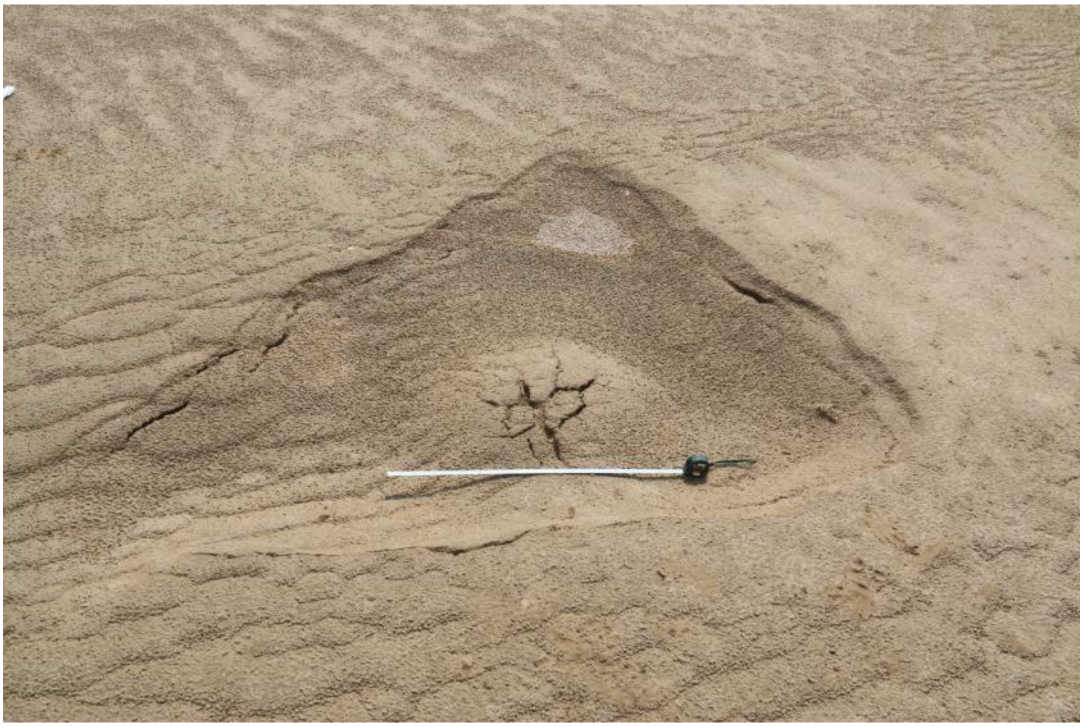

A view of a large, slightly upheaval Chinese flower-bun-like structure, it measures 65cm in diameter and 9cm in height. It developed within an ice melt depression.

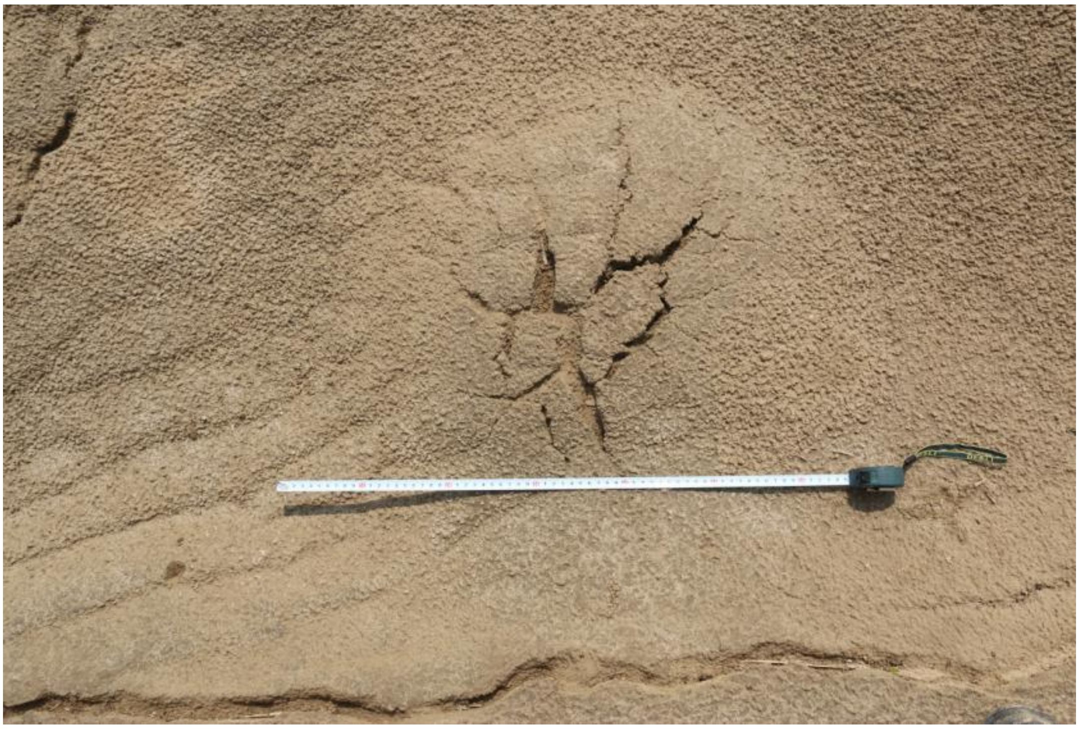

A close-up, top-down view of the above.

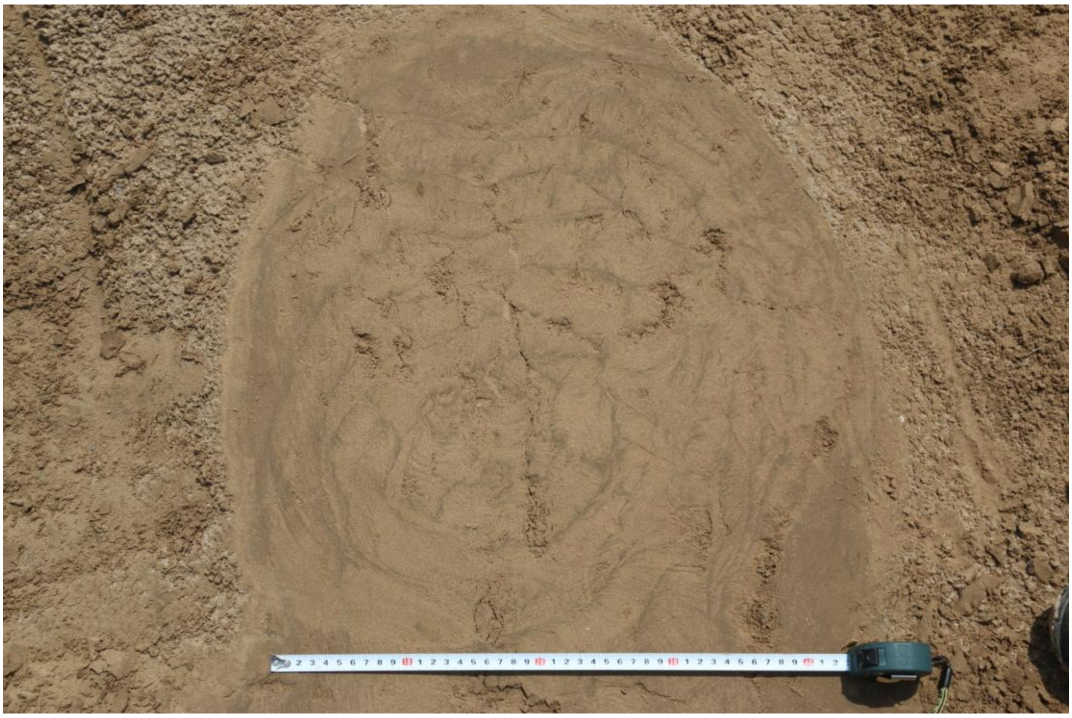

A close-up, top-down view of a cross plane section near the basement of the above structure, with clearly normal circular ripples beddings due to up-lifting of it and a cross fissure. The section is from the horizon about 5cm above the surrounding sediment surface. Plan view of same specimen as above.

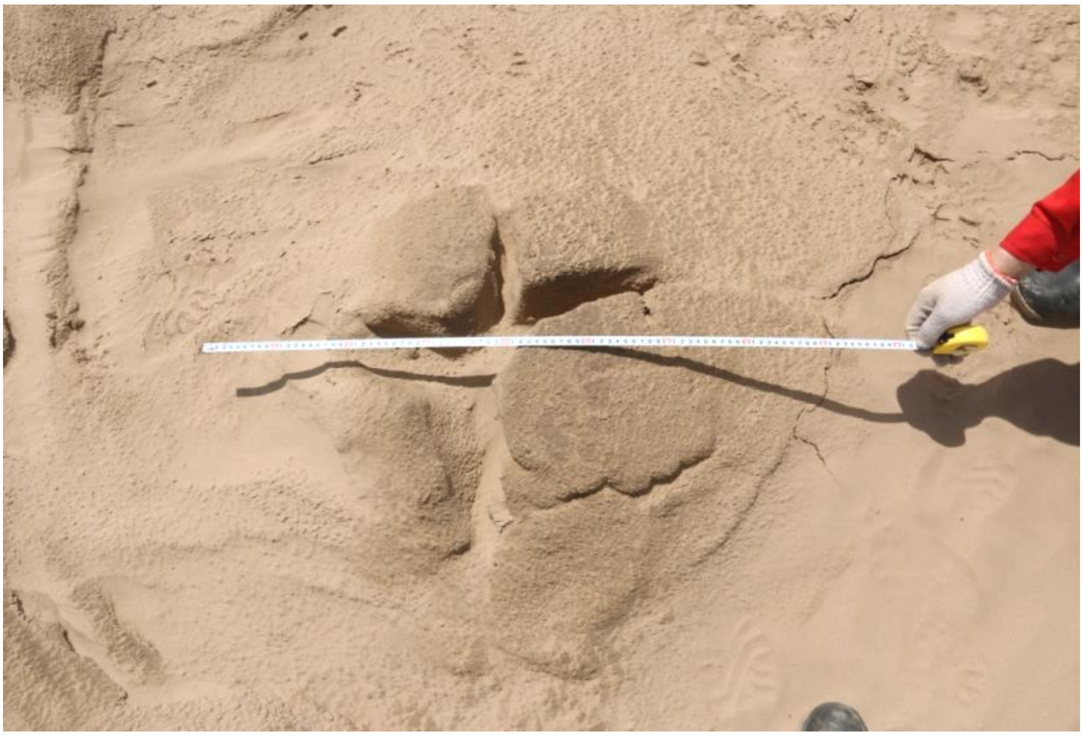

The cross-shape fissions cutting the Chinese flower-bun-like structure remarkably divide this structure into four parts.

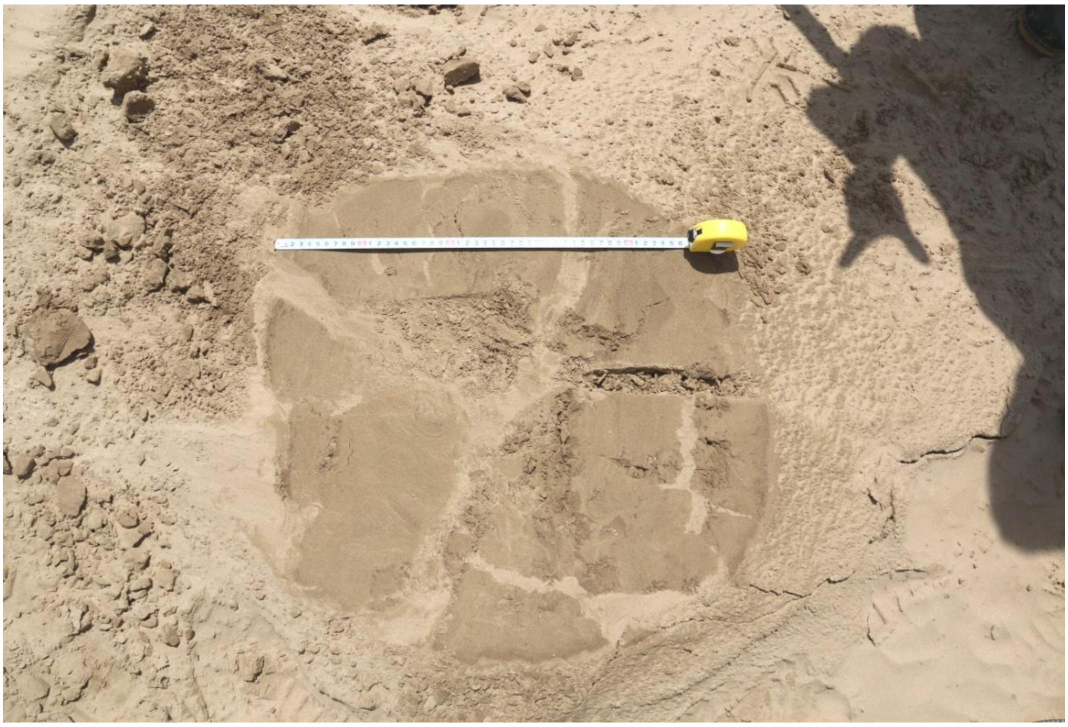

A close-up, top-down view of a cross plane section near the basement of the above structure with clearly cross-shape fissions filled by aeolian silt. The section is from the horizon about 5cm above the surrounding sediment surface. Plan view of same specimen as above.

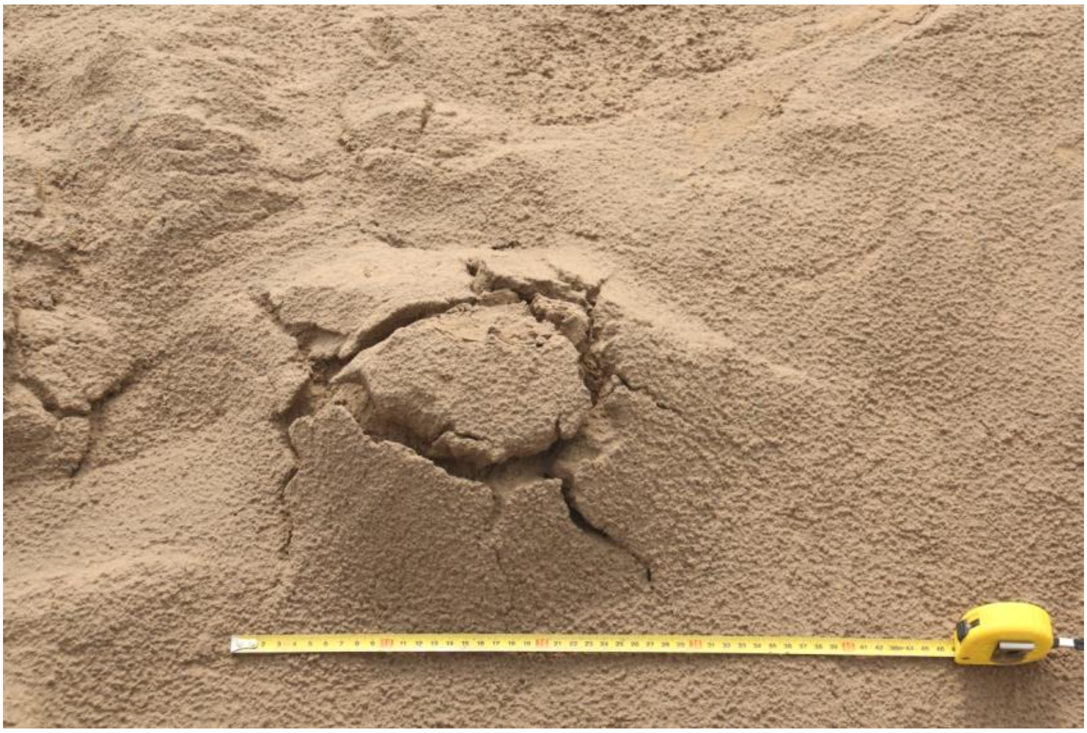

A close-up view of a typical Chinese flower-bun-like structure, with a clearly circular fission and a few radial fissions. Its is nearly circular in shape and has gently dipping and symmetrical walls.

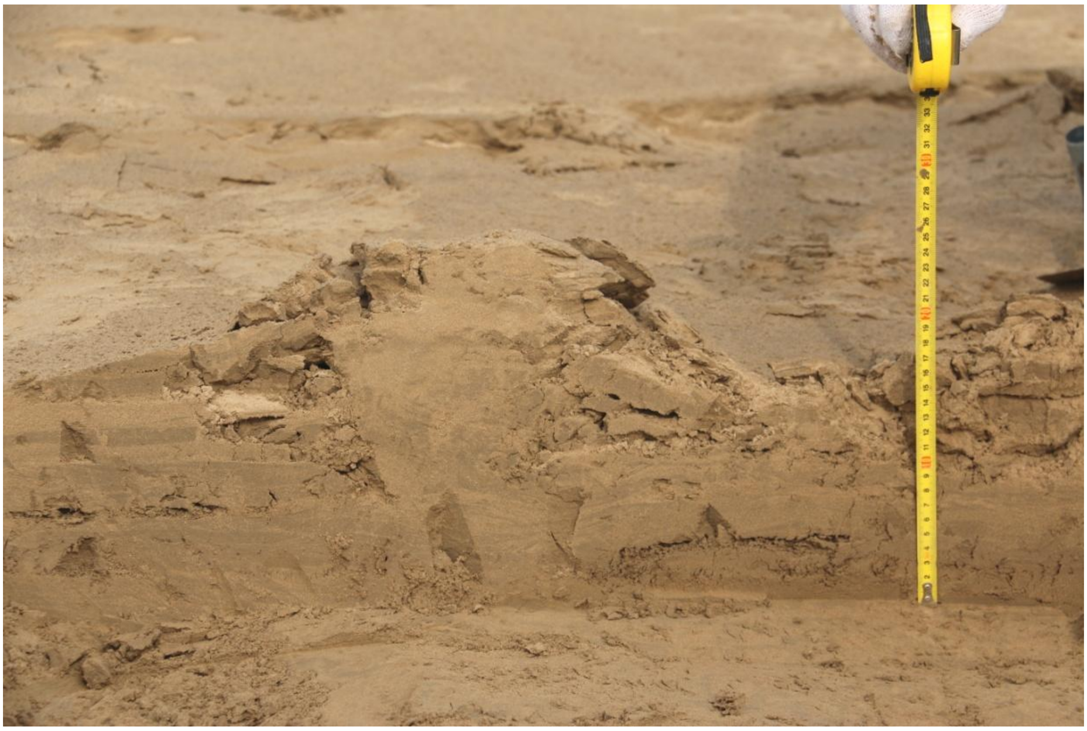

A close-up view of the vertical section of the above Chinese flower-bun-like structure. Note there are some slump fragments and two fissions on its two sides. Vertical view of same specimen as above.

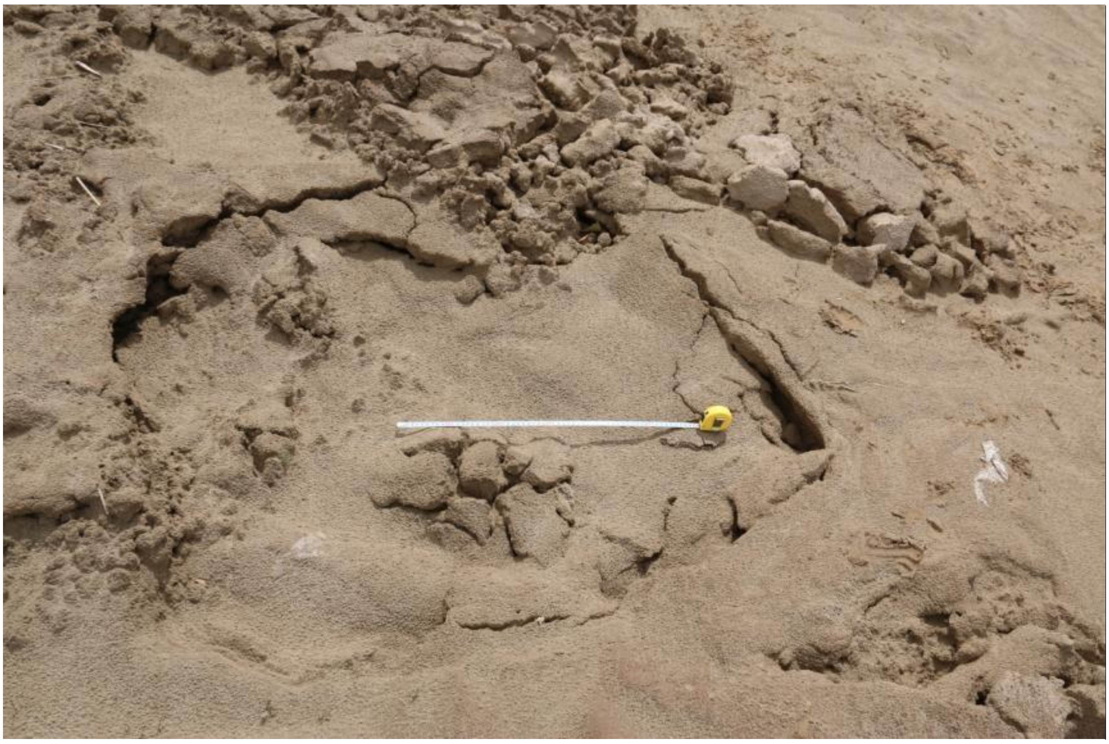

A close-up view of a Chinese flower-bun-like structure with lenient fissions, which develops within an small and irregular ice melt depression.

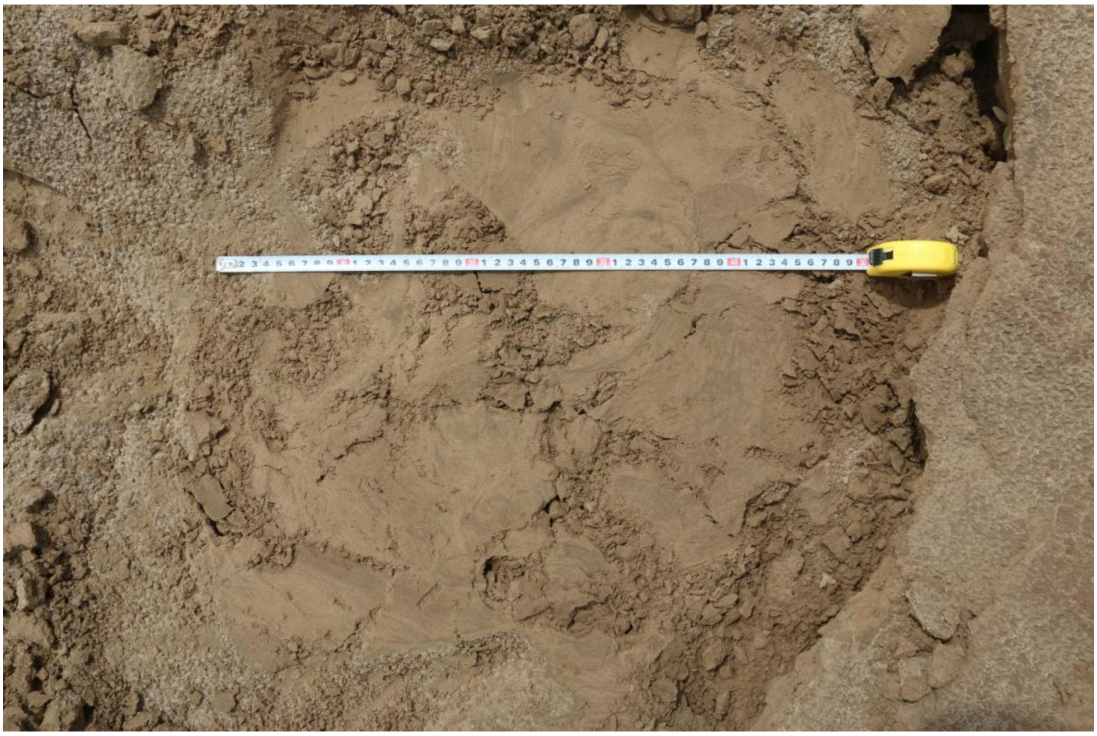

A close-up, top-down view of a cross plane section near the basement of the above structure with irregular fissures and some faint beddings. The section is from the horizon about 1-2cm above the surrounding sediment surface. Plan view of same specimen as above.

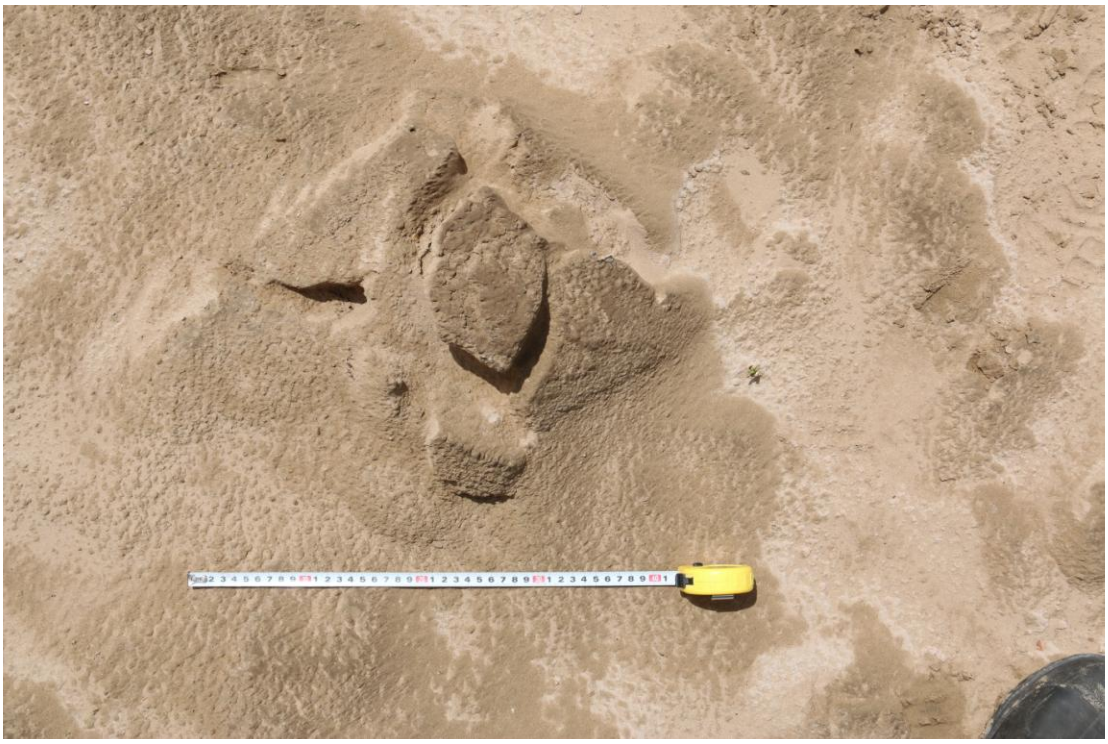

A medium-size Chinese flower-bun-like structure with clear oval fission and 4-5 radial fissions

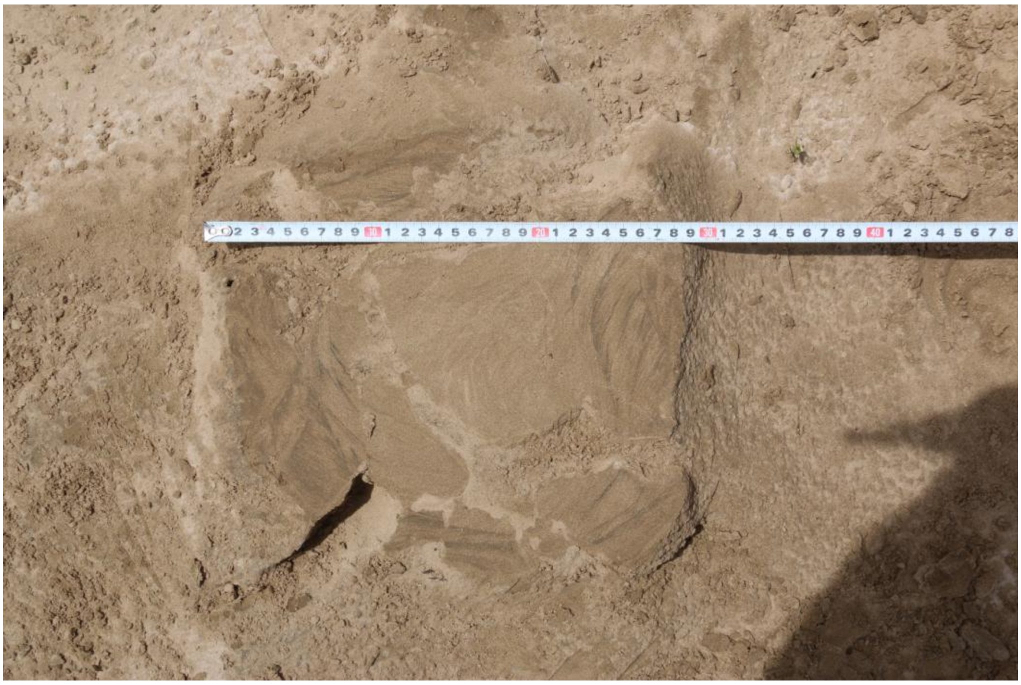

A close-up, top-down view of a cross plane section near the basement of the above structure with extremely clear and irregular fissures filled by aeolian silt and some faint beddings. The section is from the horizon about 3-4cm above the surrounding sediment surface. Plan view of same specimen as above.

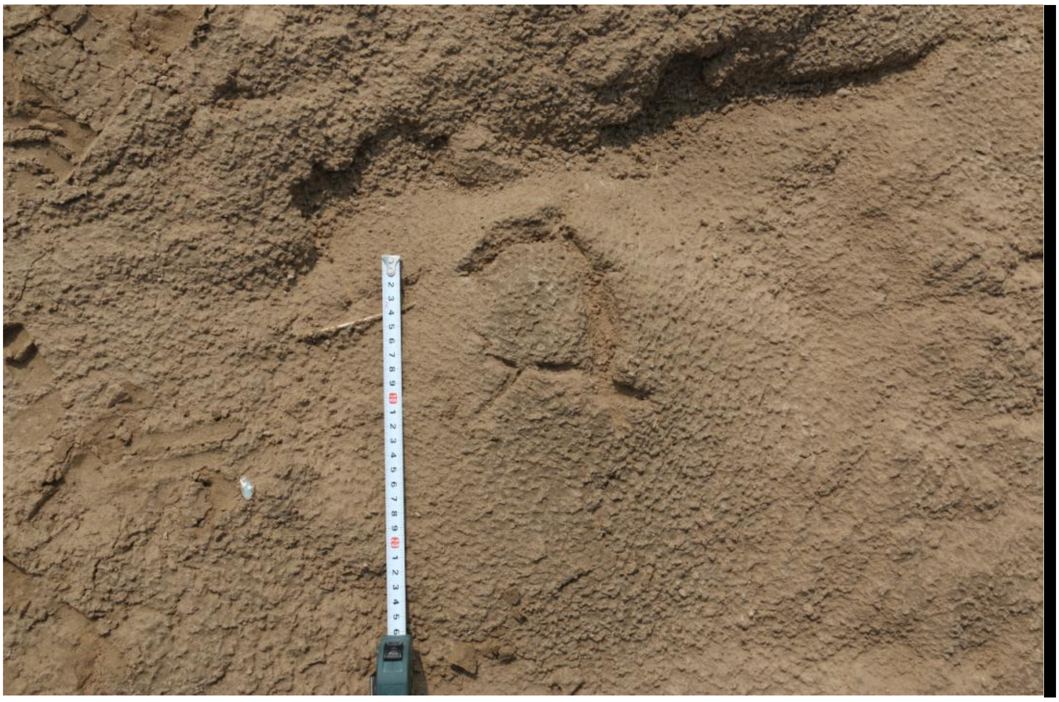

A close-up, top-down view of a Chinese flower-bun-like structure, which has a imperfect circular fission.

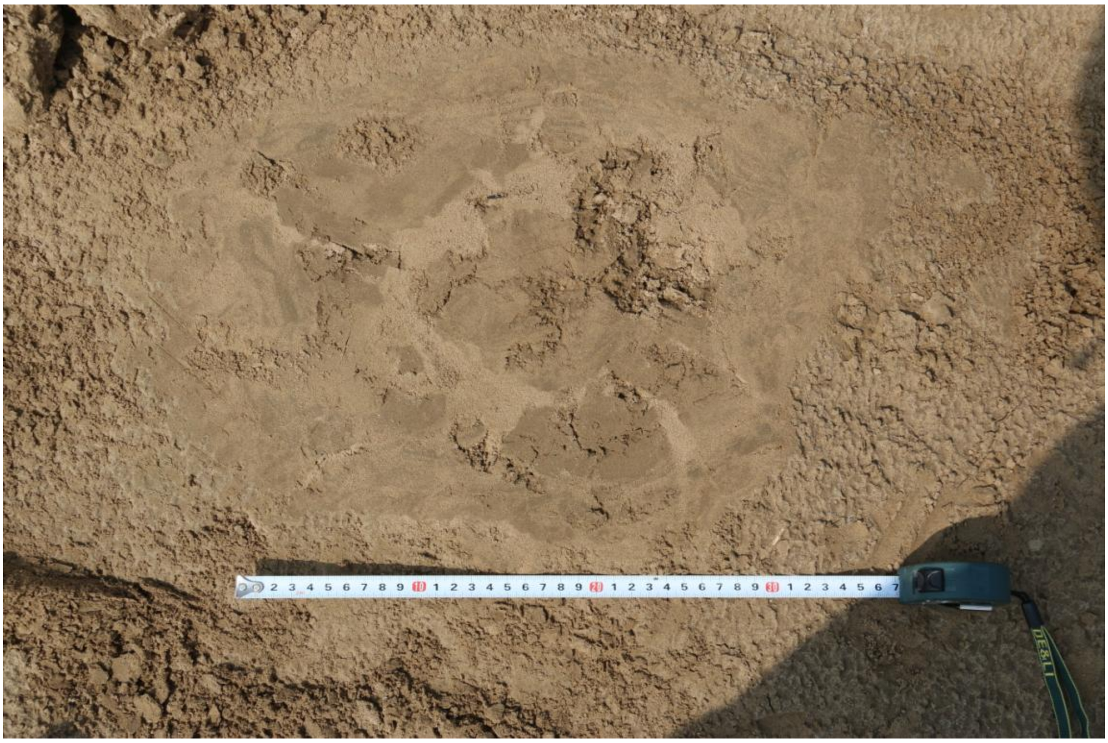

A close-up, top-down view of a cross plane section near the basement of the above structure, which looks also like a turtle shell . The section is from the horizon about 1-2cm above the surrounding sediment surface. Plan view of same specimen as above.

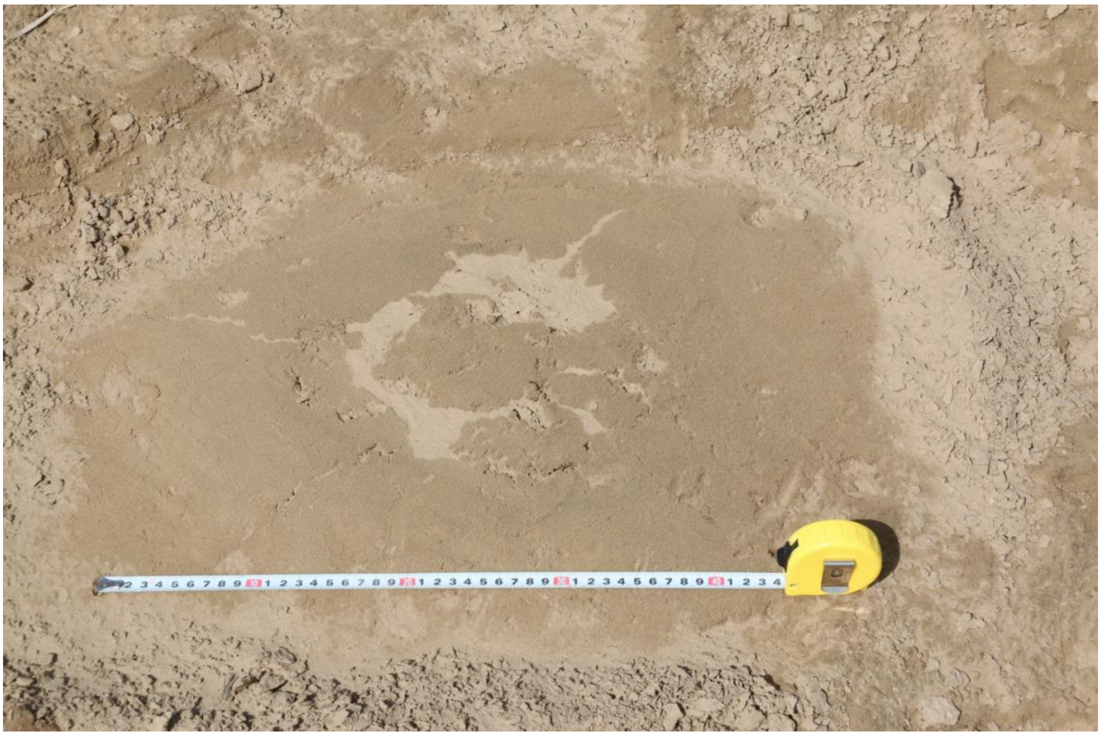

A close-up, top-down view of a cross plane section near the basement of another Chinese flower-bun-like structure, which looks also like a turtle shell . The section is from the horizon about 5cm above the surrounding sediment surface.

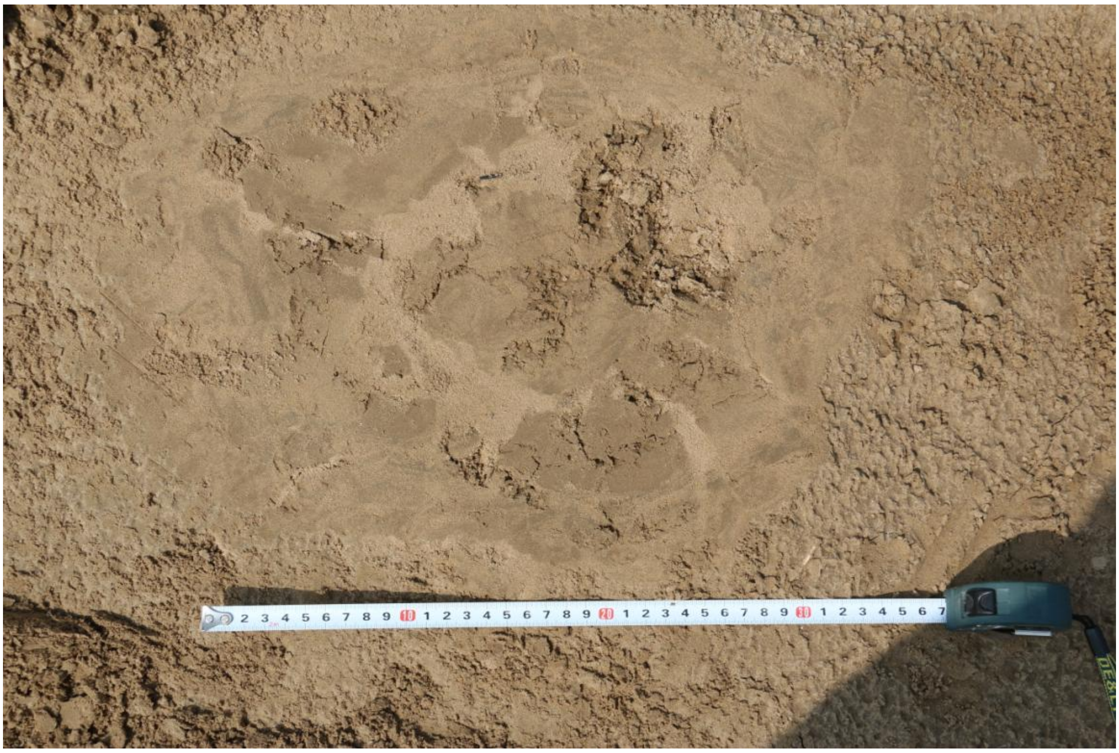

A close-up, top-down view of a cross plane section near the basement of another Chinese flower-bun-like structure, which looks also like a turtle shell . The section is from the horizon about 5cm above the surrounding sediment surface.

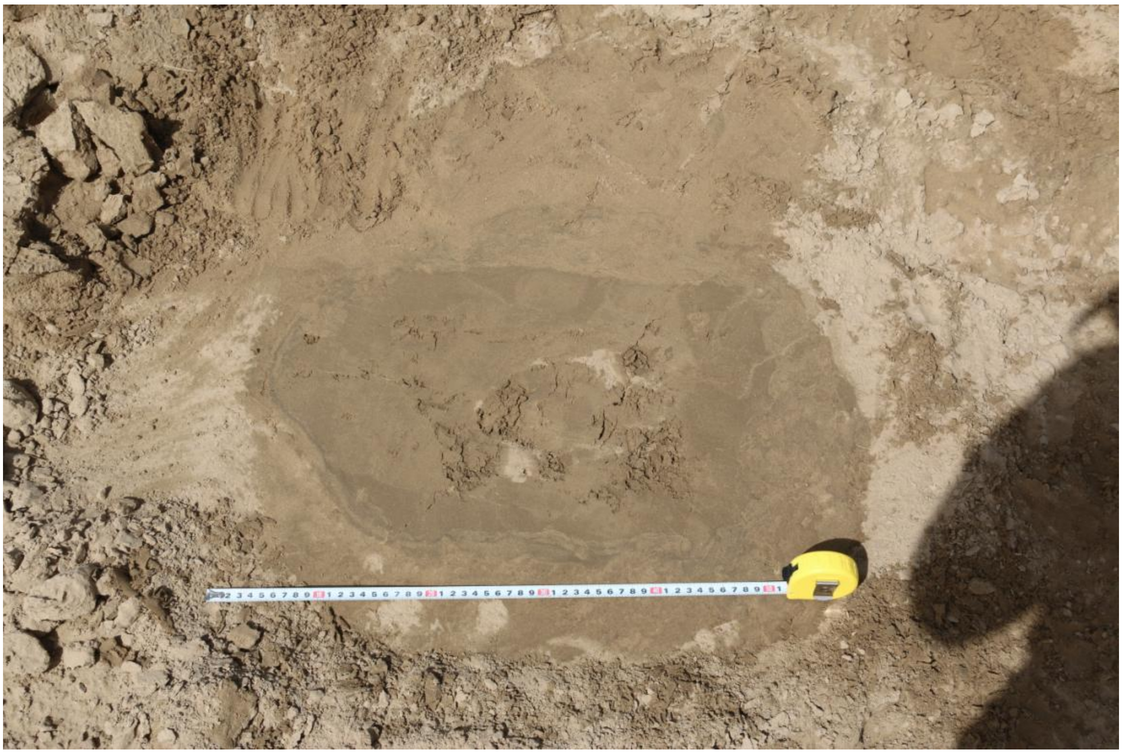

A close-up, top-down view of a cross plane section near the basement of another Chinese flower-bun-like structure, which looks somewhat like a turtle shell . The section is from the horizon about 5cm above the surrounding sediment surface.

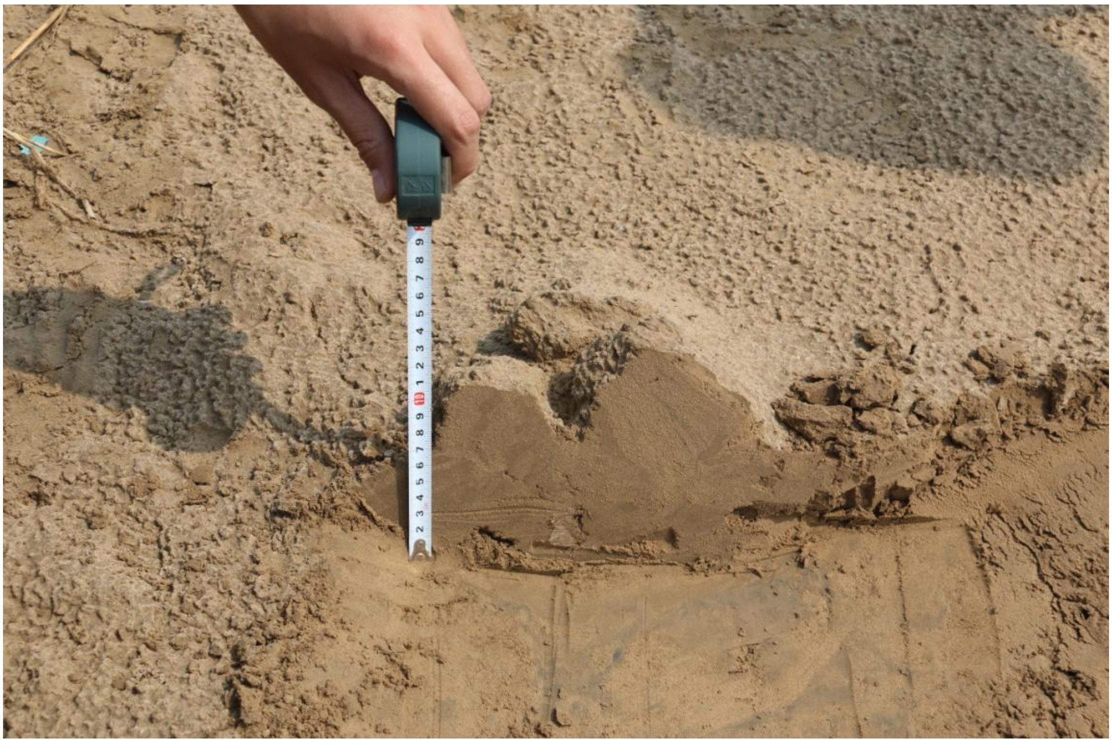

A close up view of vertical section of a small Chinese flower-bun-like structure, with some deformation beddings and a few fissures within it.

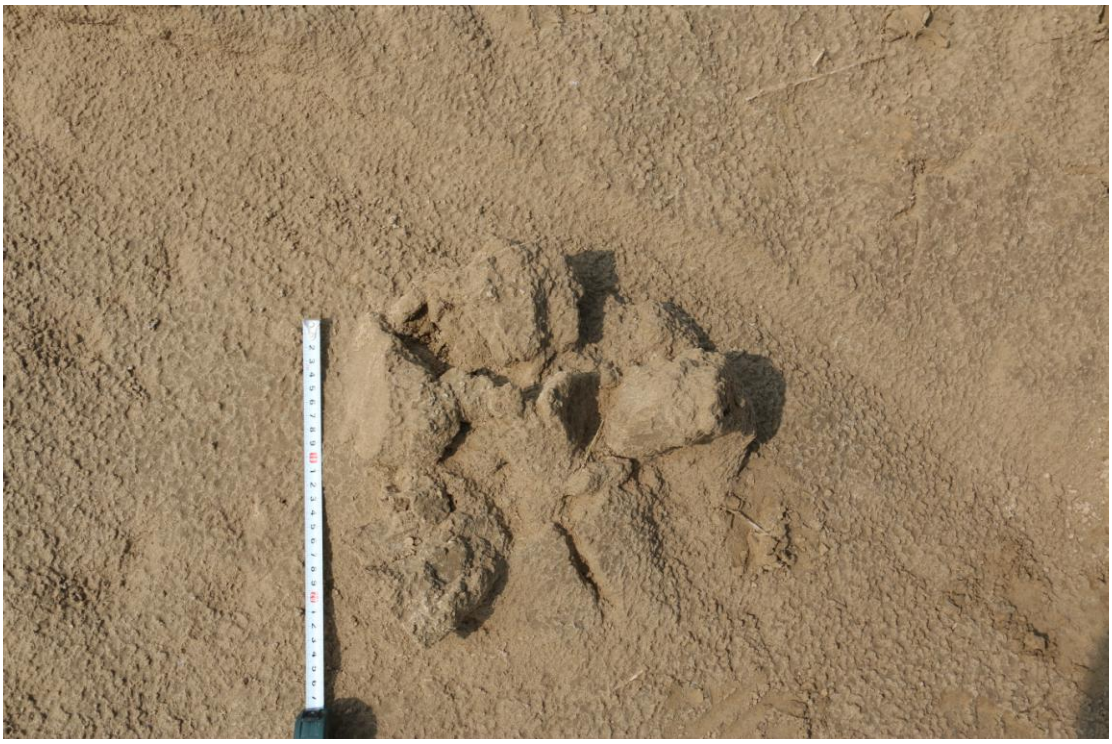

A small and extremely irregular Chinese flower-bun-like structure.

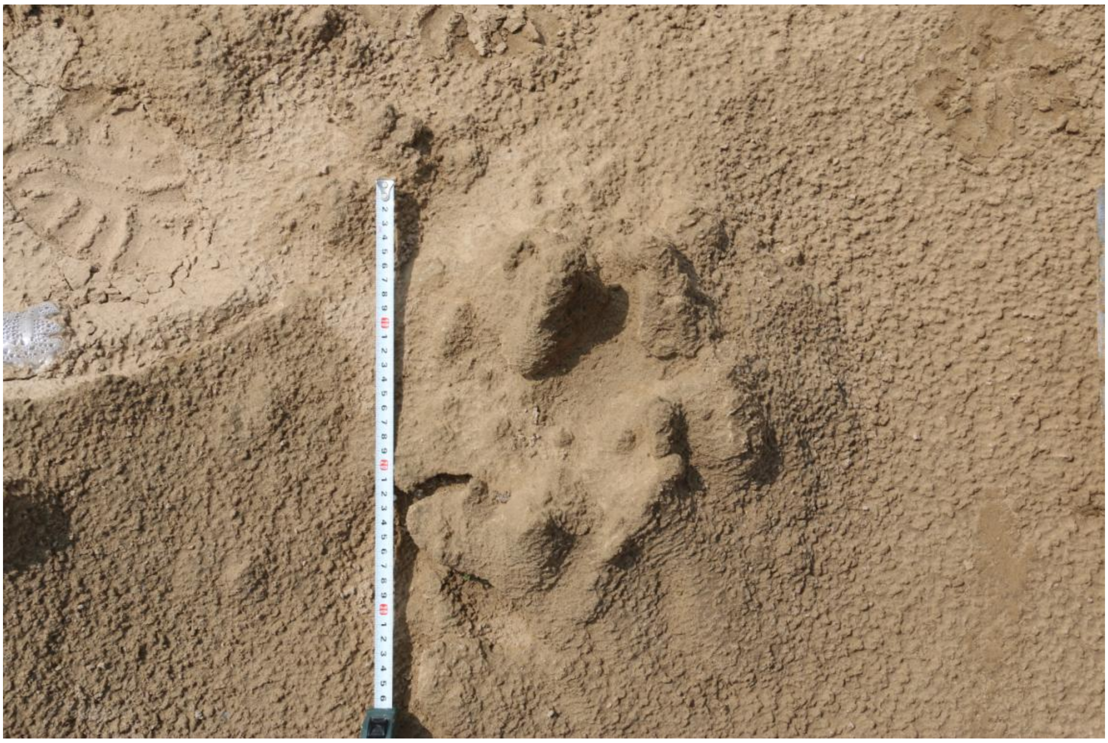

A extremely irregular Chinese flower-bun-like structure, which is hunched up intensively and reformed by late wind blow and rain wash.

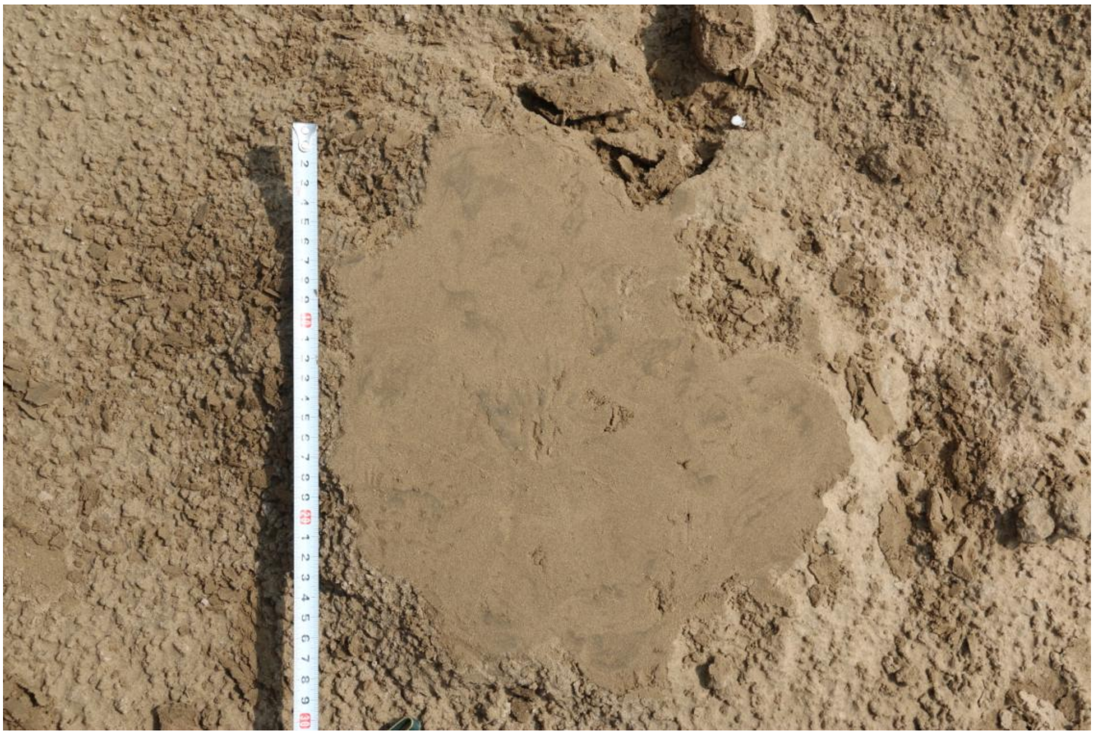

A close-up, top-down view of a cross plane section of the above, which has a lot of irregular deformation beddings. The section is from the horizon about 5cm above the surrounding sediment surface. Plan view of same specimen as above.

3. Ice-induced silt mushrooms

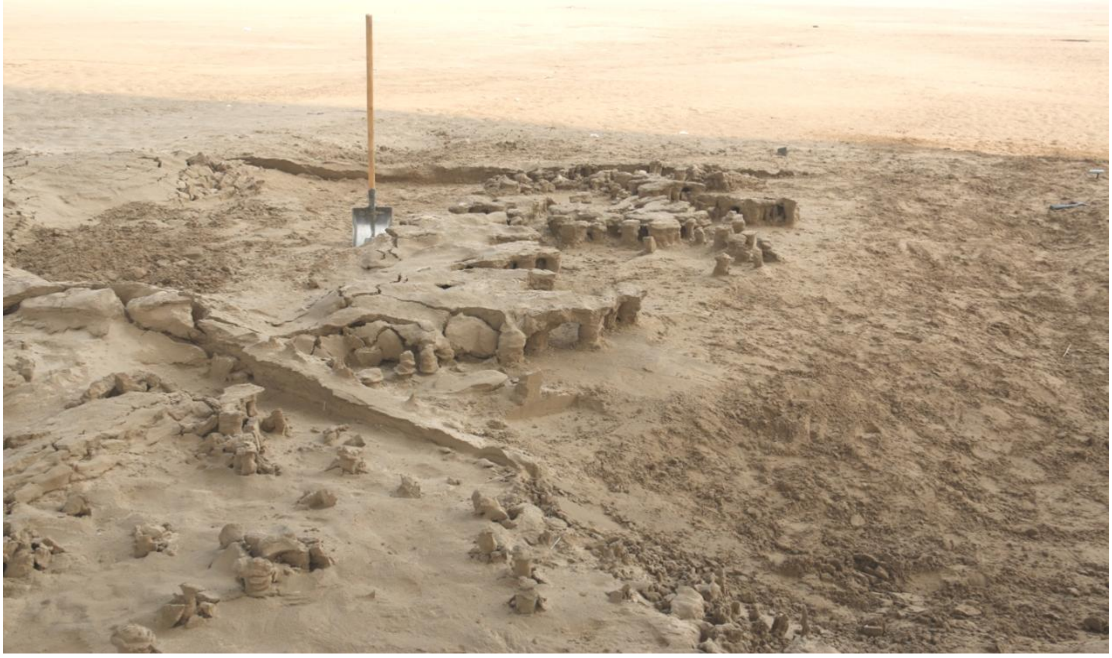

Two groups of silt mushrooms have developed within the two areas delineated by two ice blocks. The standing shovel is about 140cm in length.

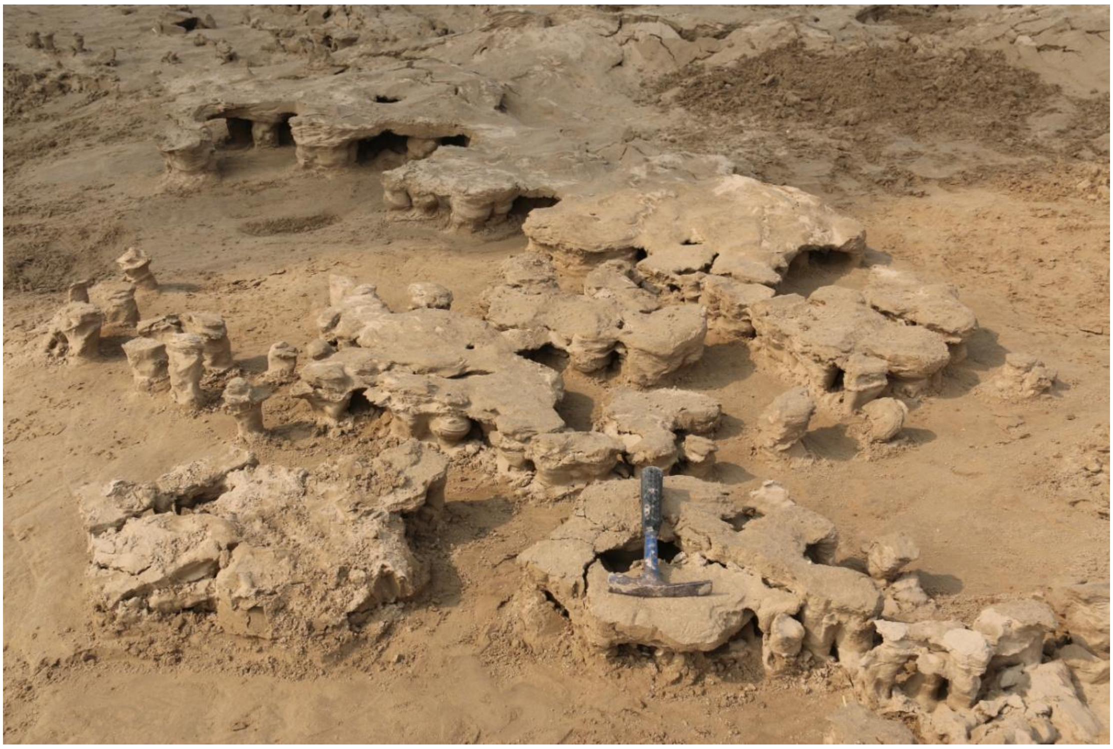

A group of well-developed silt mushrooms with a individual and unified caps. Also, developing within areas delineated by an ice block. The hammer is 28cm long.

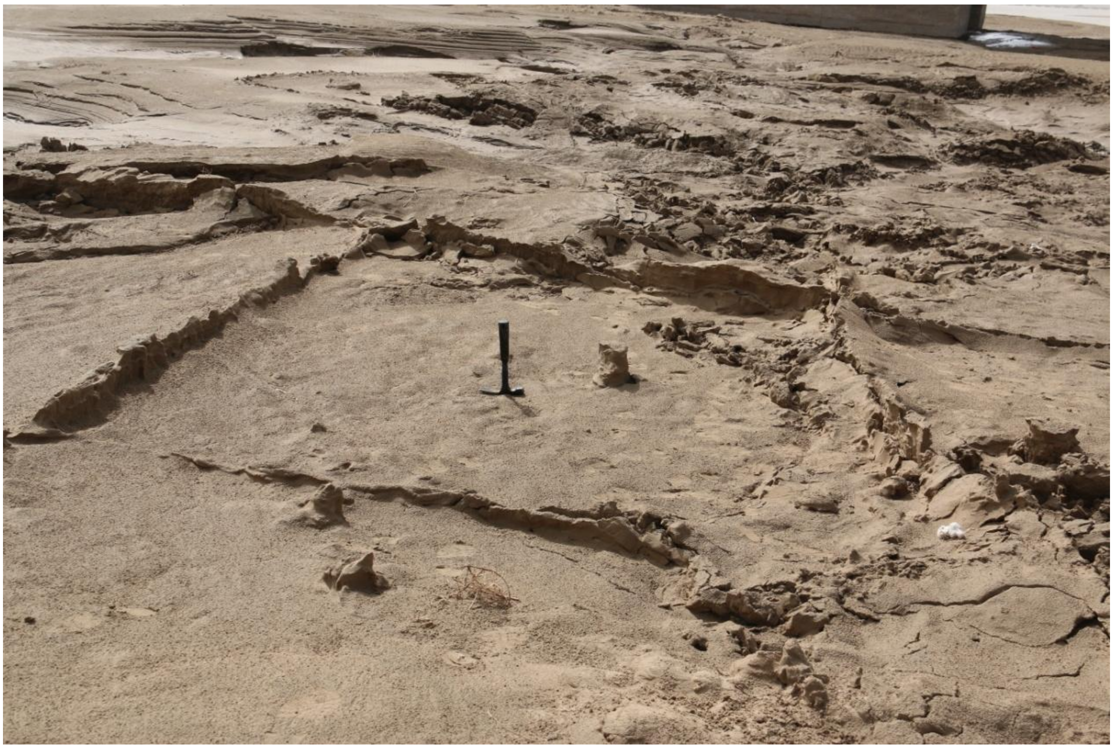

A few silt mushrooms developing in isolation. Also, developing within areas delineated by an ice block. The hammer is 28cm long.

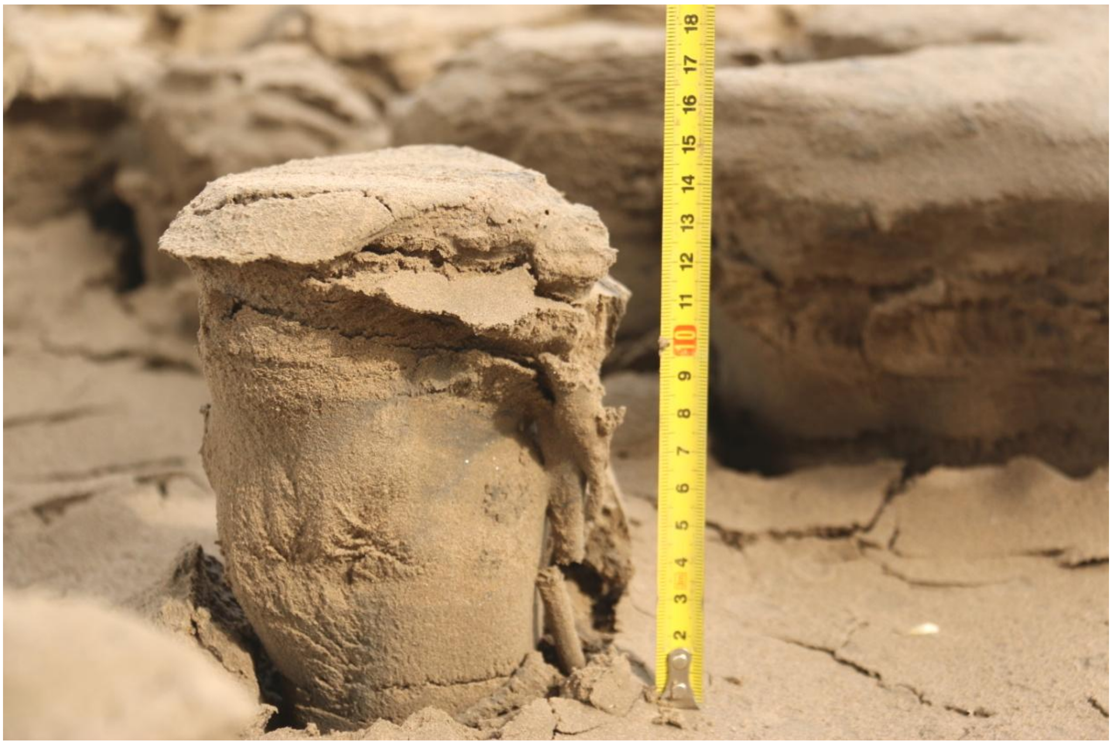

A well-developed and sturdy silt mushroom. Its cap is relatively small and the stem is relatively large. On right side of the stem there is an extremely irregular small circular column that has the same origin as the silt mushroom.

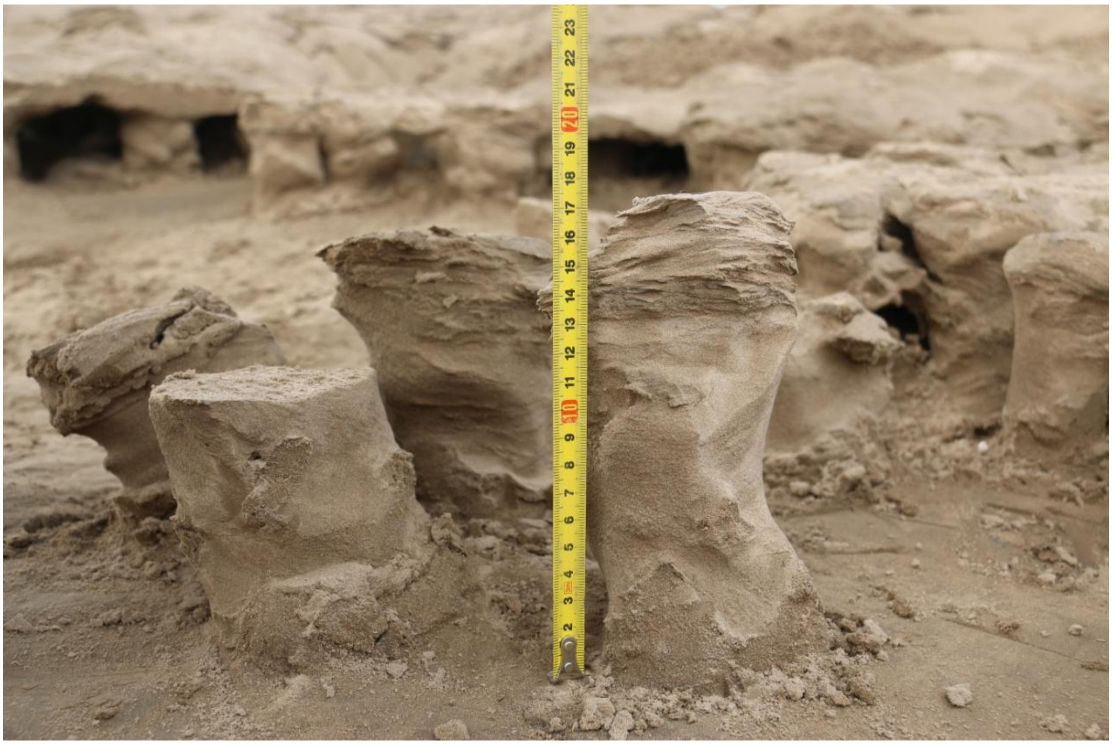

A close-up view of a group of silt mushrooms. In the foreground is a group of single silt mushrooms with independent caps and to the rear are compounded silt mushrooms with common caps. The stems have special, curved surfaces. The cap of the mushroom in the lower left of the image has been cut away, and so the top is very flat and the stem is shorter than that of the other silt mushrooms.

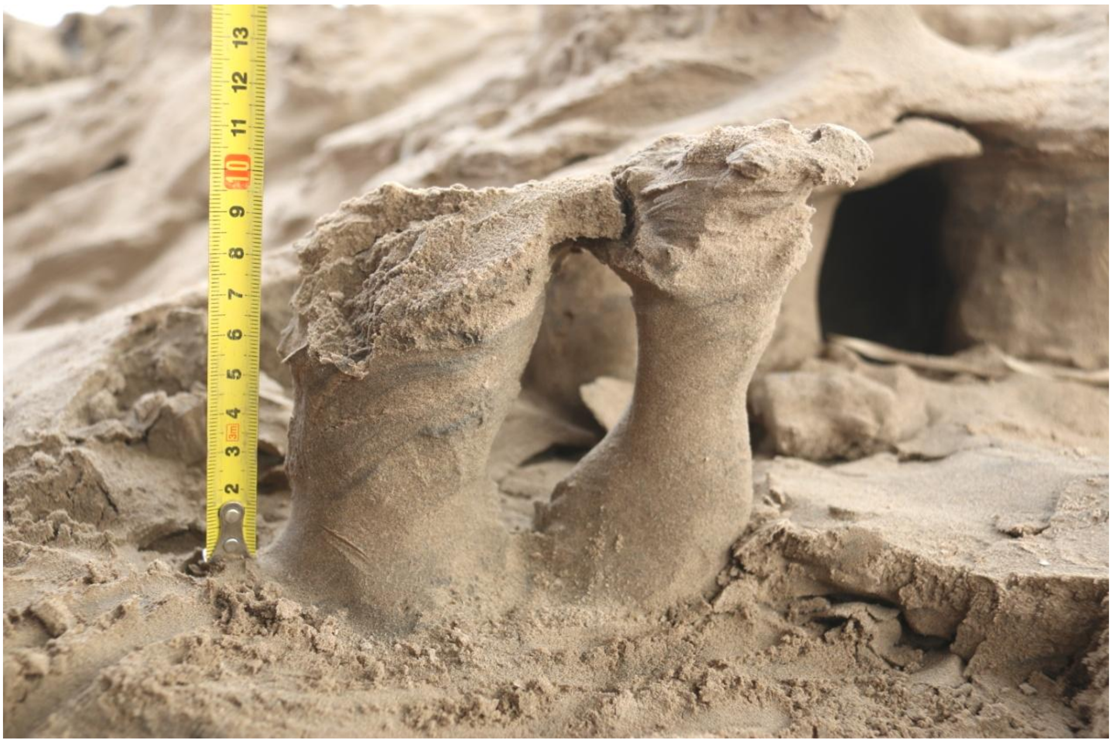

Two silt mushrooms with slanted bedding planes, atop their stems are imperfect, but connected caps, caused by their caps collapsing. They also have smooth stems.

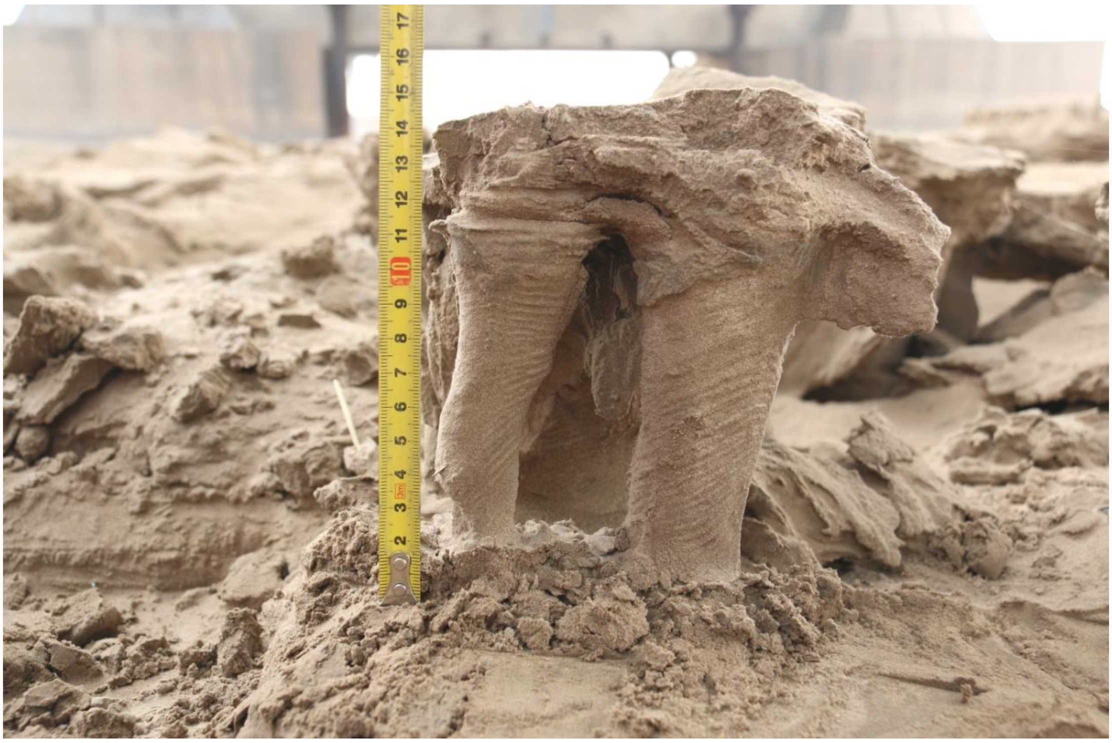

Two silt mushrooms with clear inclined beddings. Atop their stems are extremely irregular and connected caps, again caused by their caps collapsing.

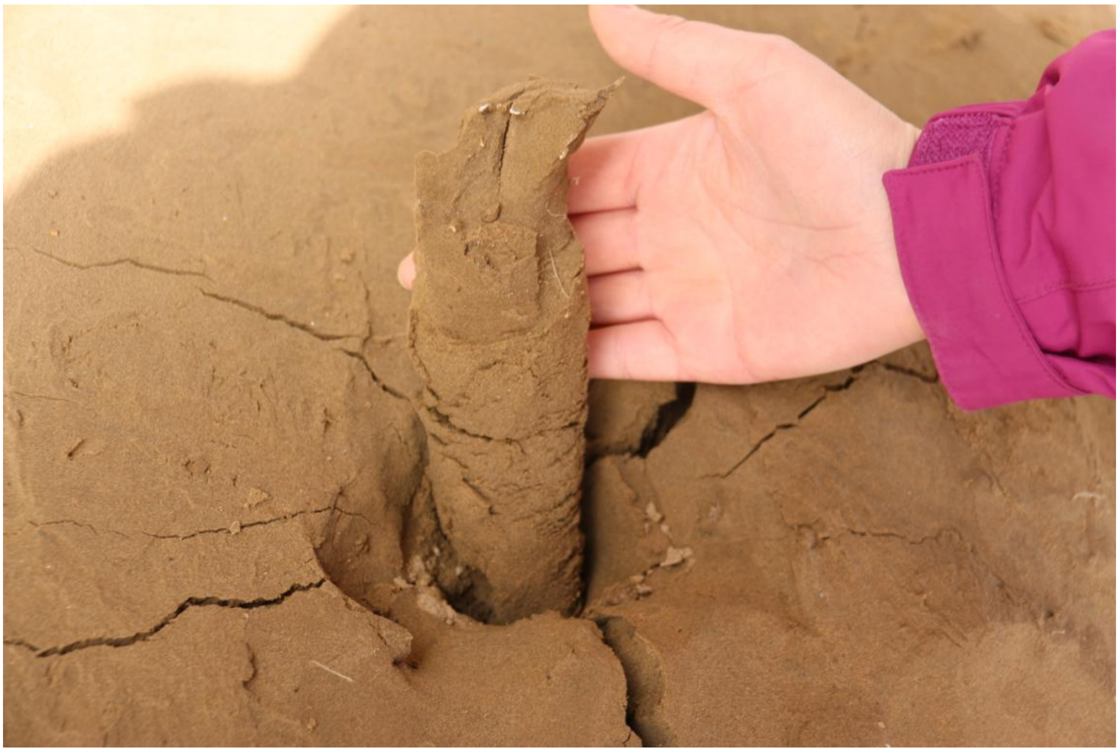

A perfect mushroom stem with a circular column shape.

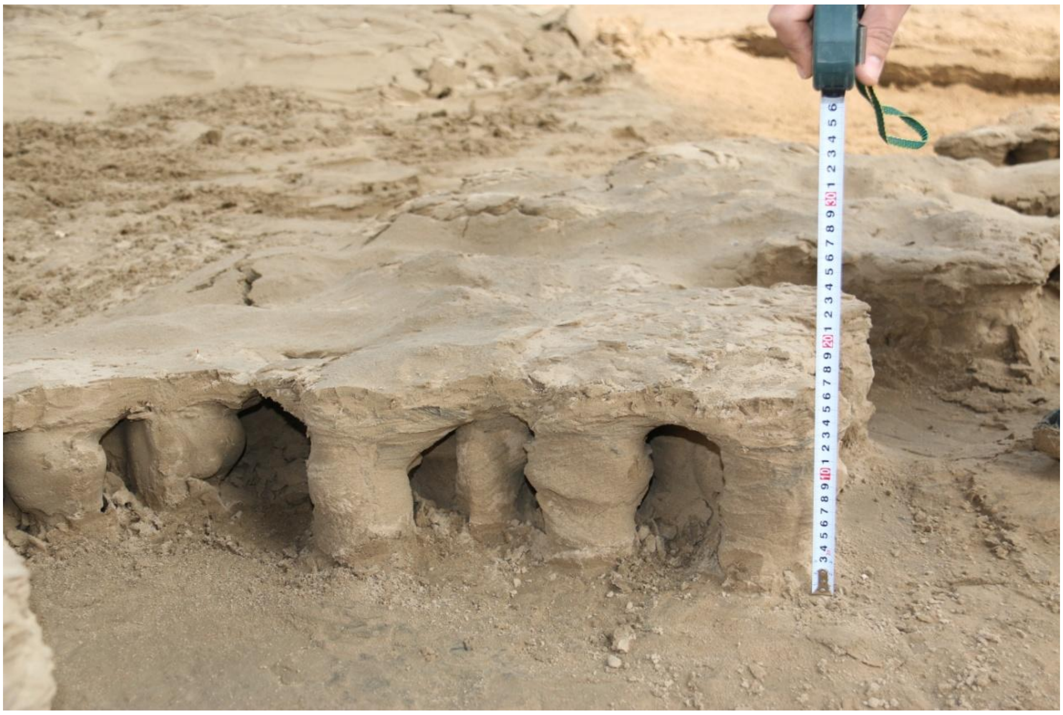

A group of silt mushrooms with a unified cap. They share a large unified cap interspersed with fissures and convex shapes in the stems. This is caused by the development of many silt mushrooms with sturdy stems all in close proximity. The stems have especially curved surfaces resulting from the special shapes of the holes in the ice block. Consequently, this has led to the formation of many chambers between the stems.

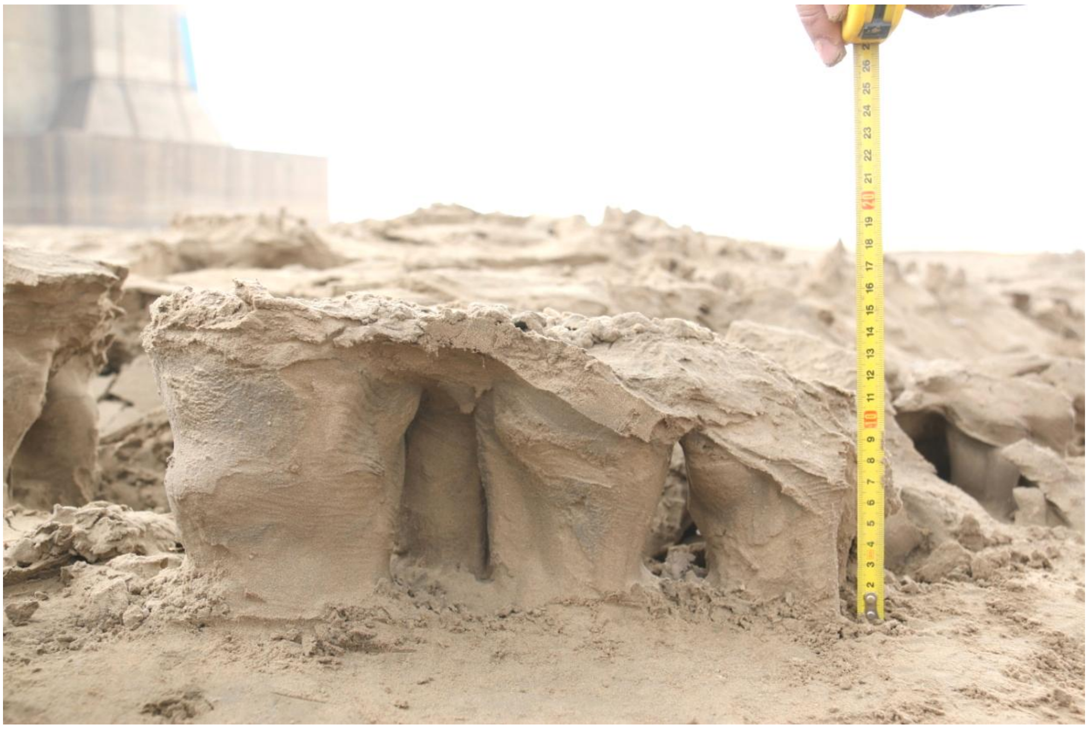

A small group of silt mushrooms. They share a thin connected cap, caused by the formation of many mushrooms with thick stems, some bedding planes and curved surfaces, all in close proximity. This also resulted in the formation of several chambers. The cap is relatively thin and is level on the left but dips sharply to the right due to the ice thinning in the margin of the ice block.

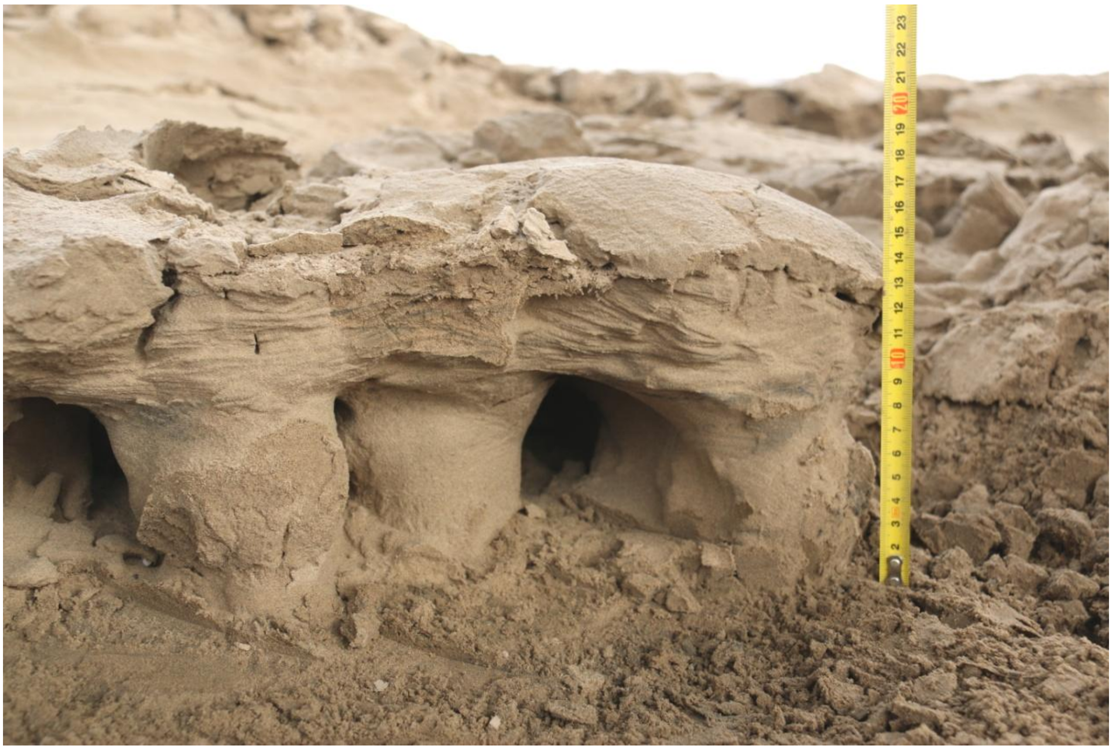

A small group of silt mushrooms. They share an extremely thick and nearly flat connected cap, measuring almost 10cm thick. They have clear bedding planes due to their close proximity. They also have thick and close stems with some bedding planes, resulting in the formation of chambers.

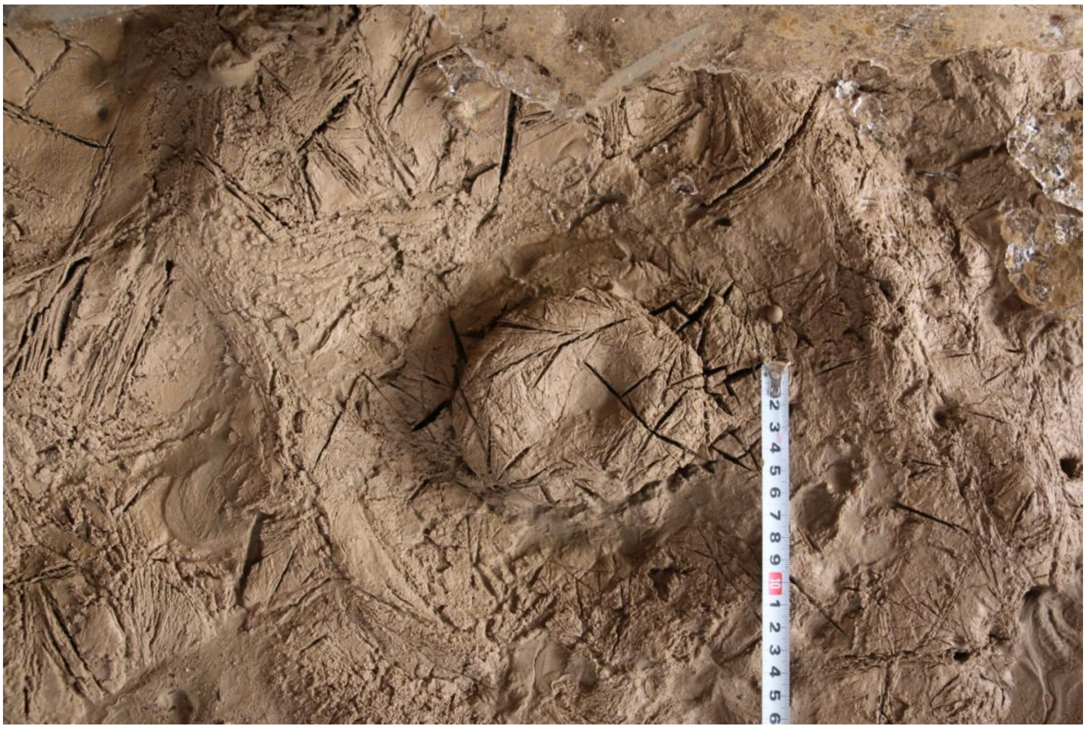

A close-up, top-down view of a mushroom top, with a whole top and accompanied by a great deal of ice crystal marks and ice frozen cracks. view from above.

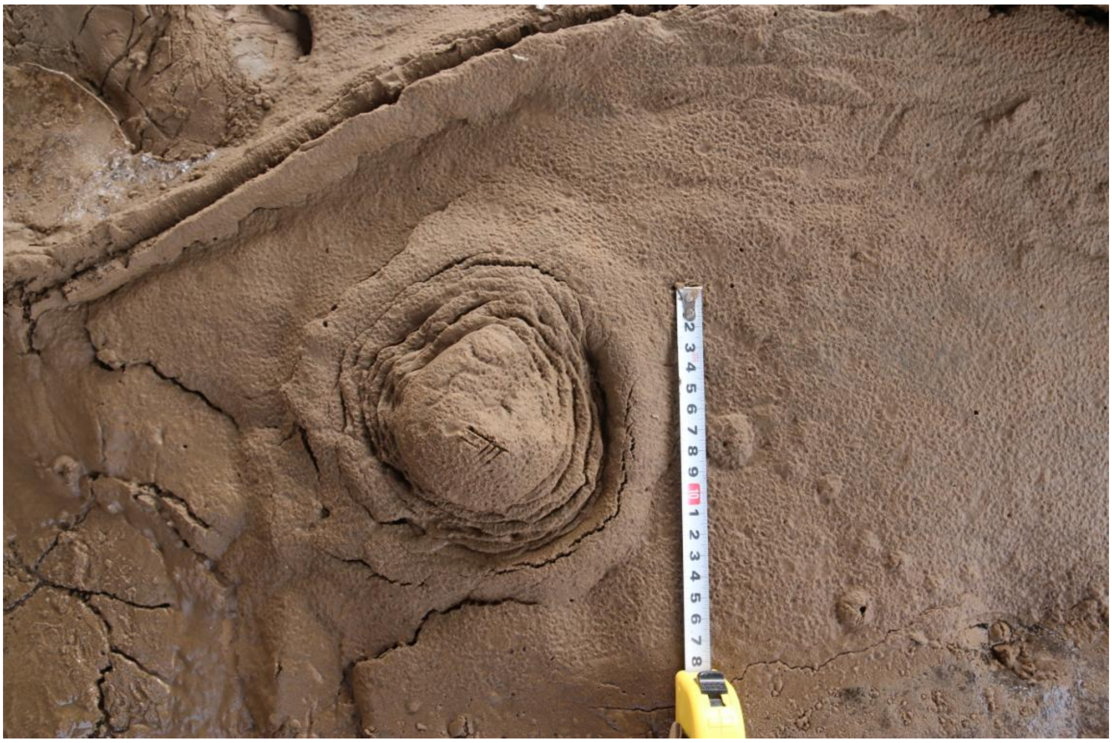

A close-up, top-down view of a mushroom top, likes a cabbage. view from above.

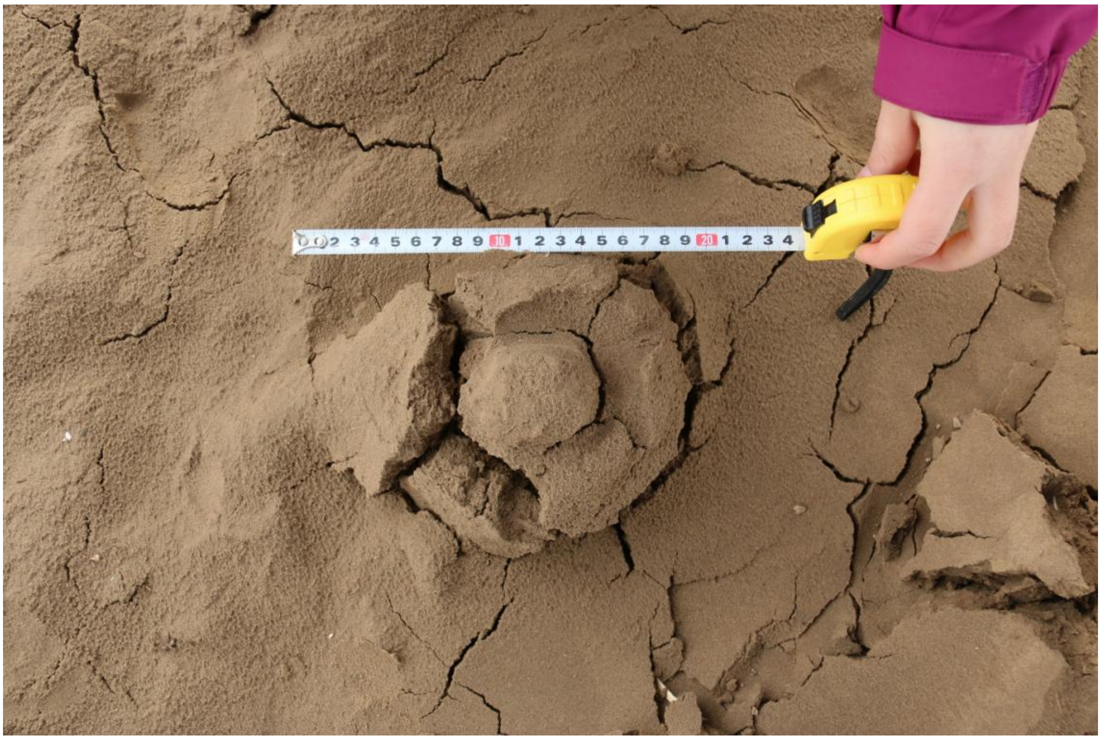

A close-up view of a mushroom top, with a circular fission and a few radial fissions. view from above.

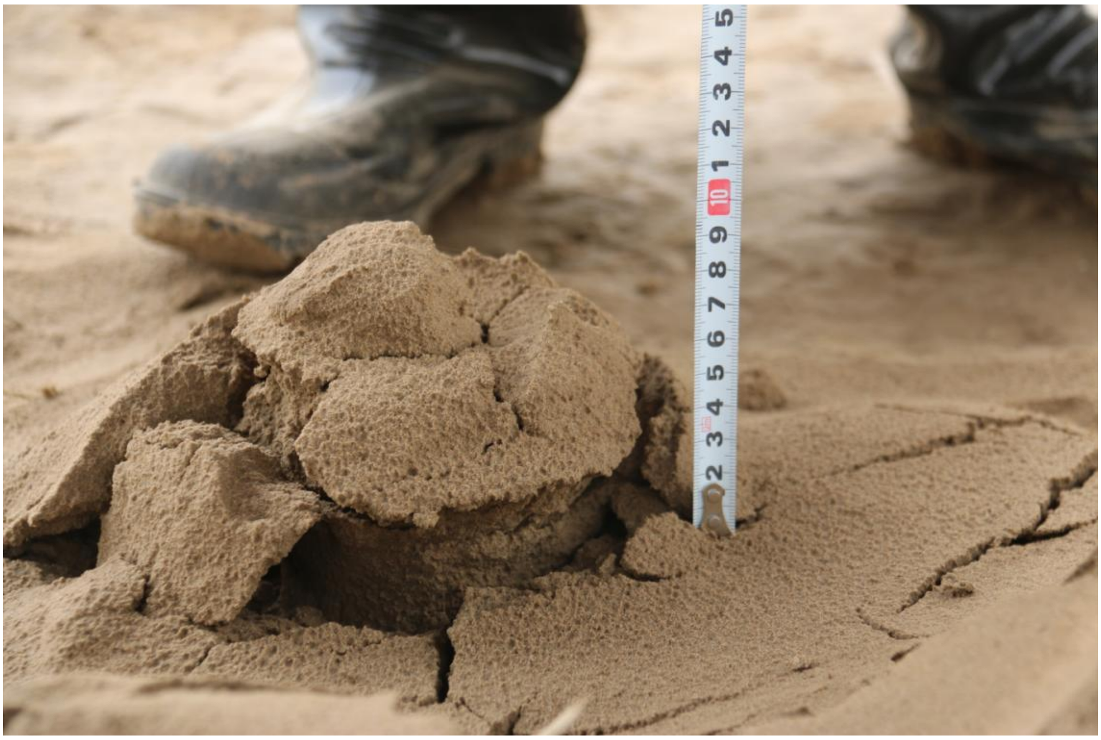

A close-up view of a silt mushroom under construction.

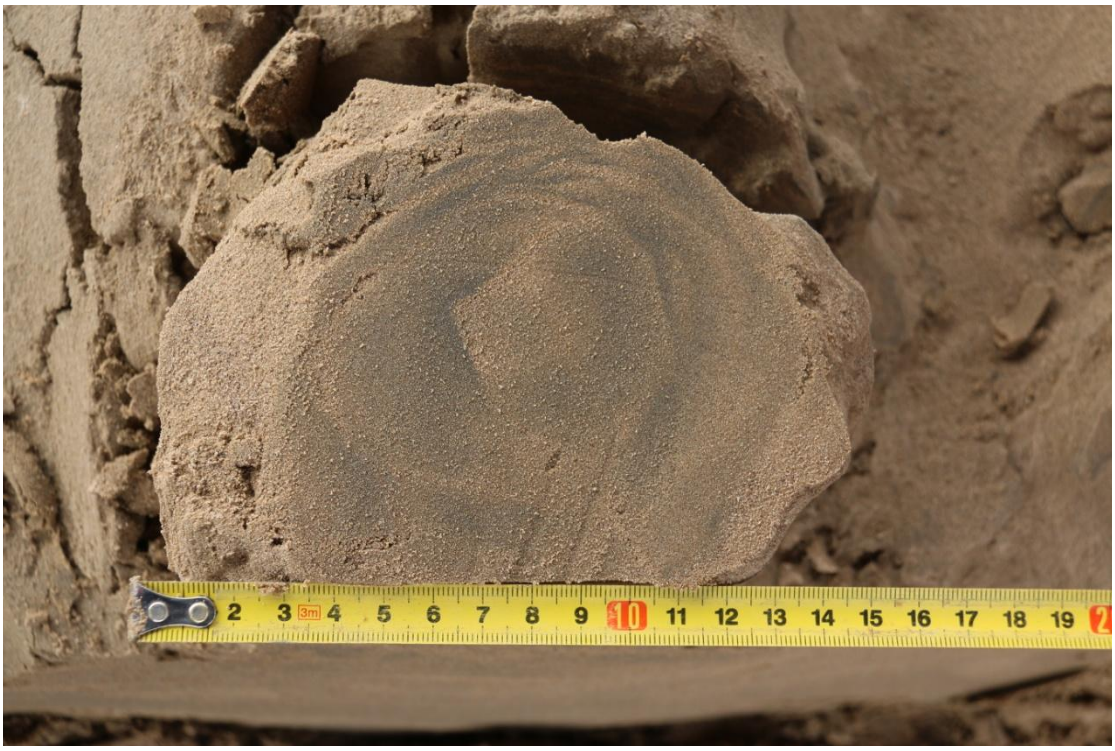

A close-up, top-down view of a planed cross section of a large stem. The irregular circular bedding planes can be clearly observed. view from above. The section is from the horizon about 8cm above the surrounding sediment surface of above.

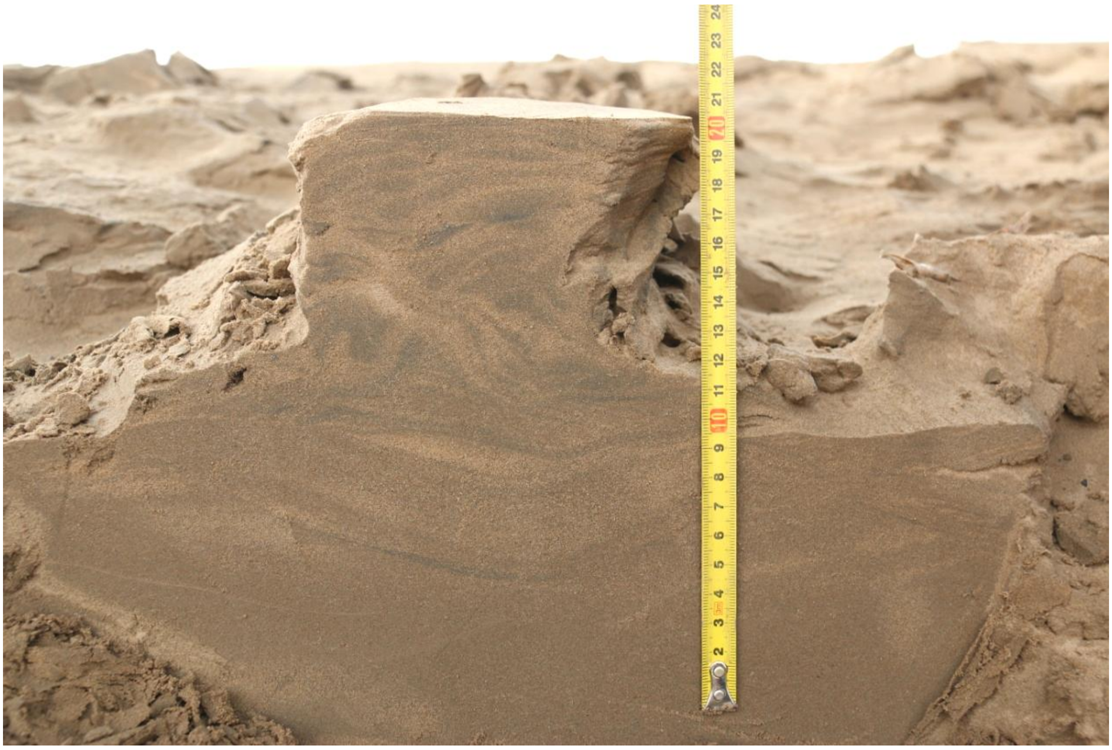

A vertical cross section of a stem. The cap has been cut off and the stem has small, irregular, deformed bedding planes and, concave bedding planes at the base.

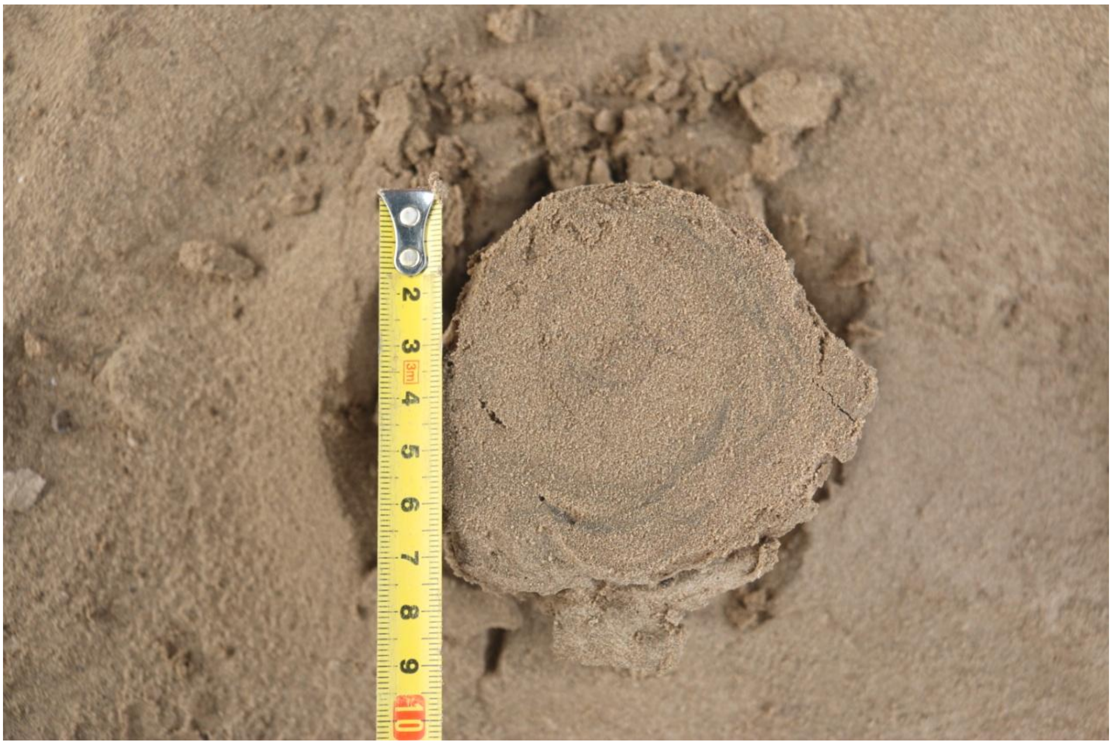

A close-up, top-down view of a plane cross section of a medium-sized stem. Irregular circular beddings s can be clearly observed. view from above. The section is from the horizon about 5cm above the surrounding sediment surface of above.

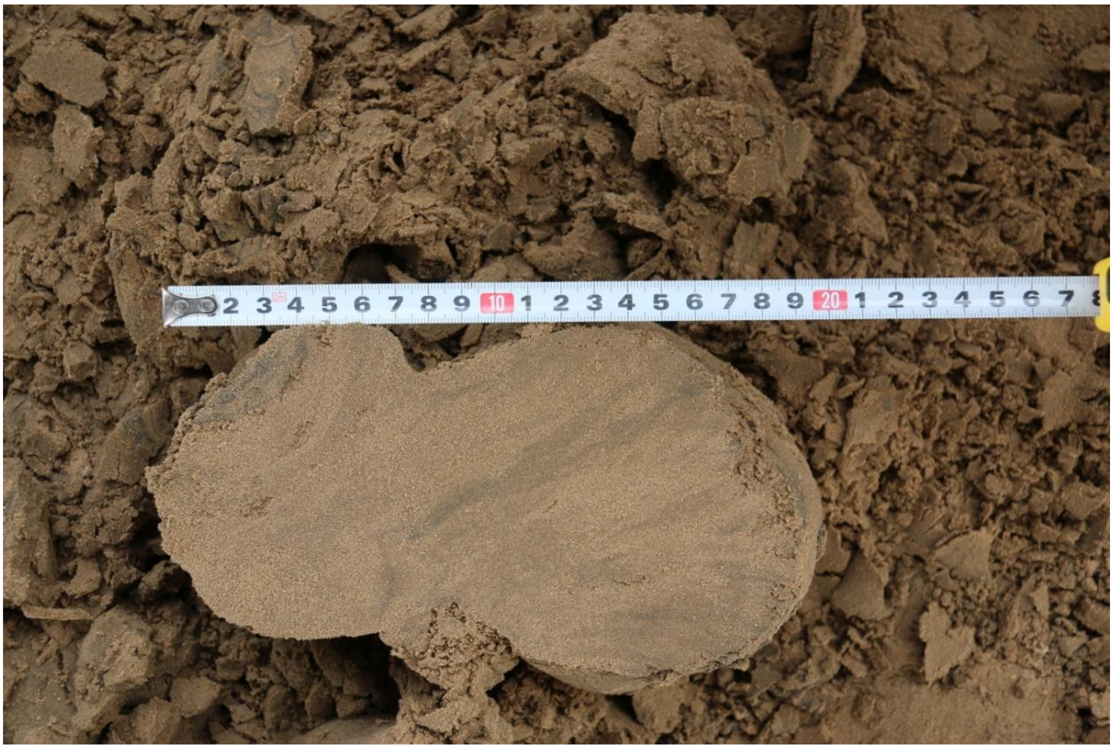

A close-up, top-down view of the cross section of two compound stems shaped like a figure eight. The section is from the horizon about 6cm above the surrounding sediment surface.

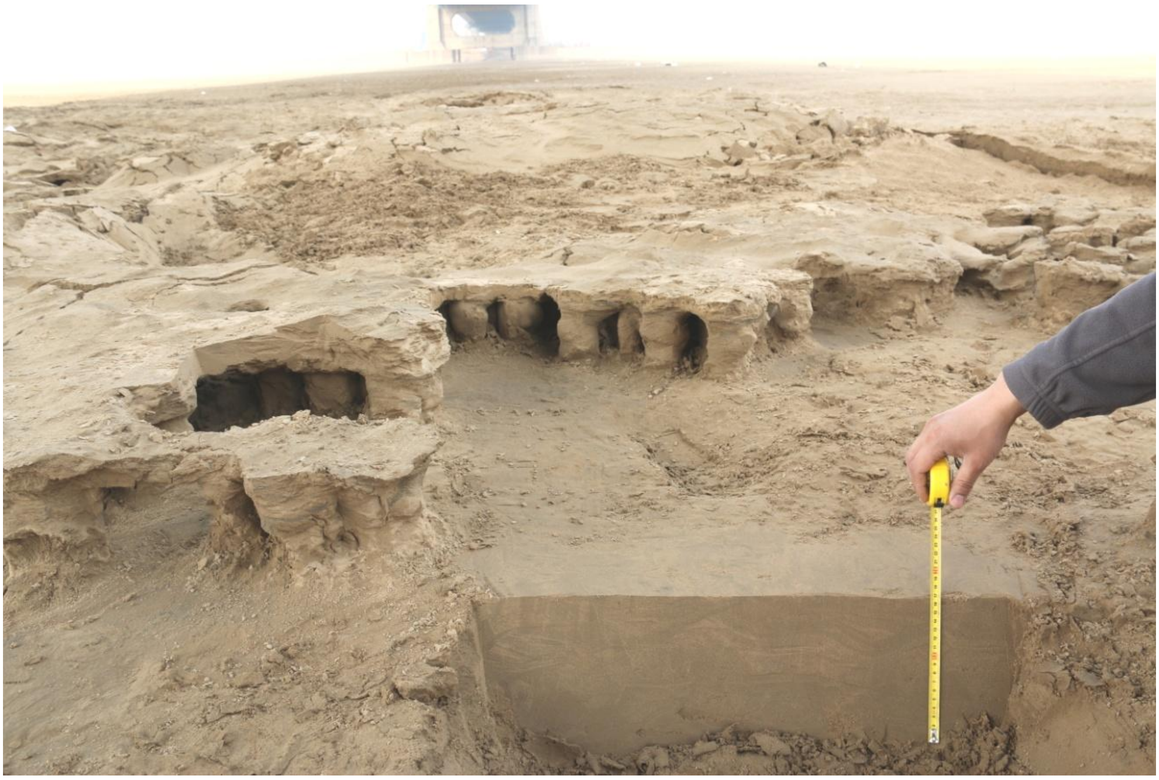

A group of silt mushrooms with a unified cap. The stems stand in a close formation, which prevents the cap from collapsing. Hence a single, unified cap, with a great deal of chambers has formed. Their base has some slight deformation beddings (the lower right).

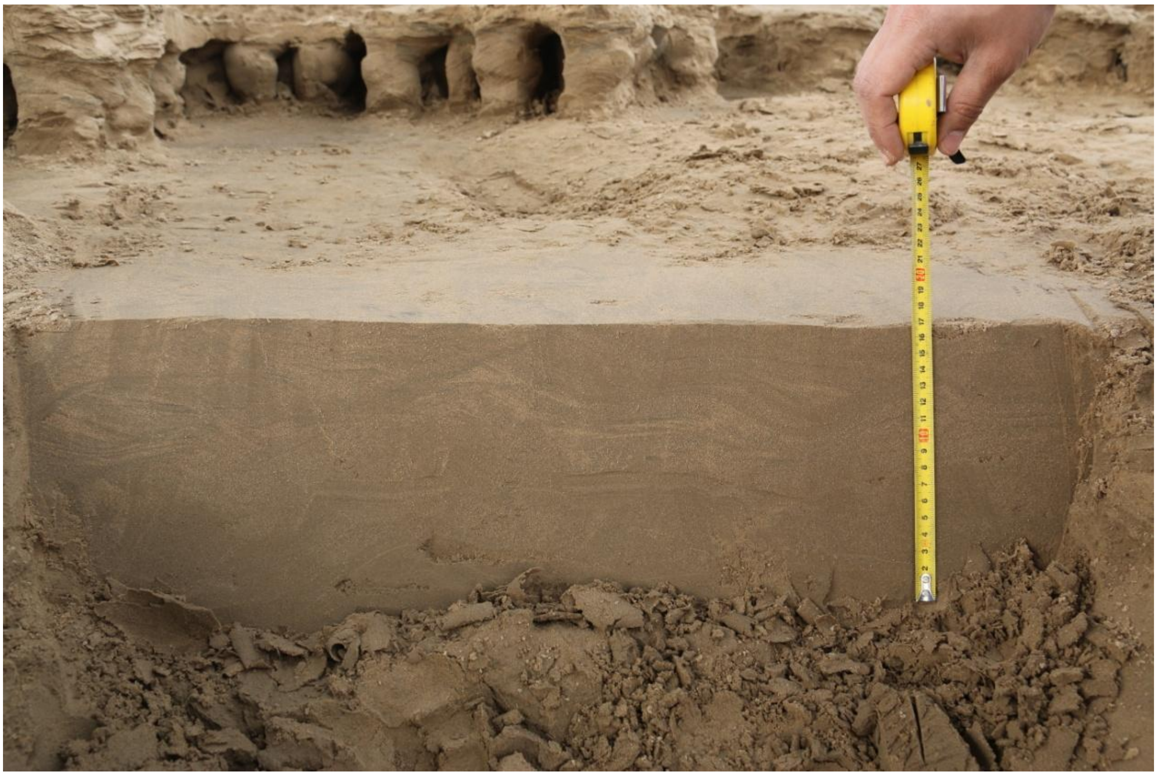

A close-up view of a vertical section (in the lower right of the above photo). Here, their base is slightly deformed because of the disturbing influence of ice blocks. Close view of same specimen as above.

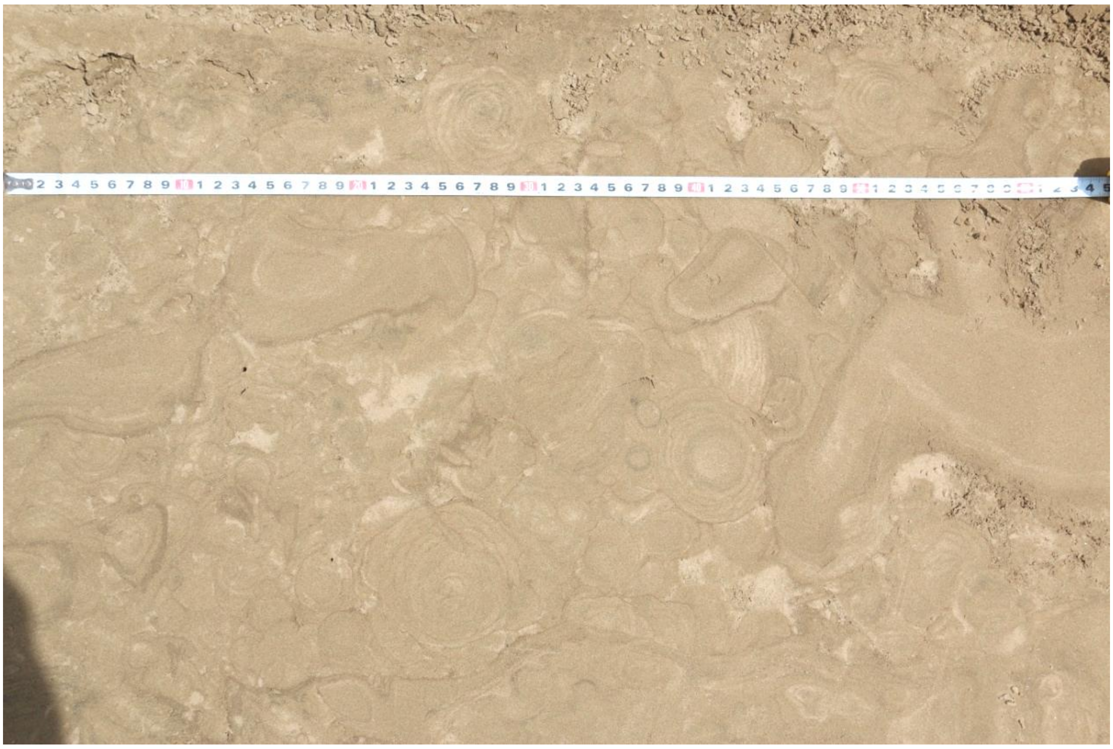

A close-up view of the plane cross section of the base of a group of silt mushrooms. The most striking feature is the circular beddings formed by the base of silt mushrooms. The section is from the horizon about 0cm above the surrounding sediment surface of above.

4. Ice cast marks

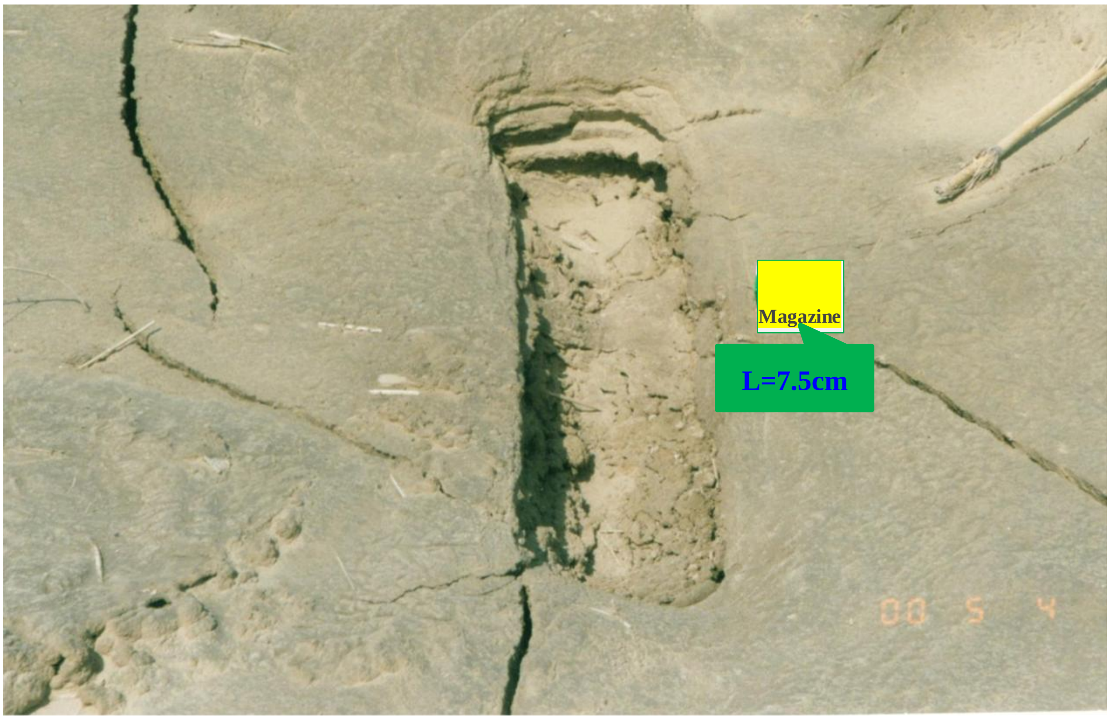

An long rectangle ice cast mark formed by an upright ice block. It is 38cm long, 9cm wide and 6-7cm deep. On the left lower corner there are some irregular bean-like structures formed by ice water deposition.

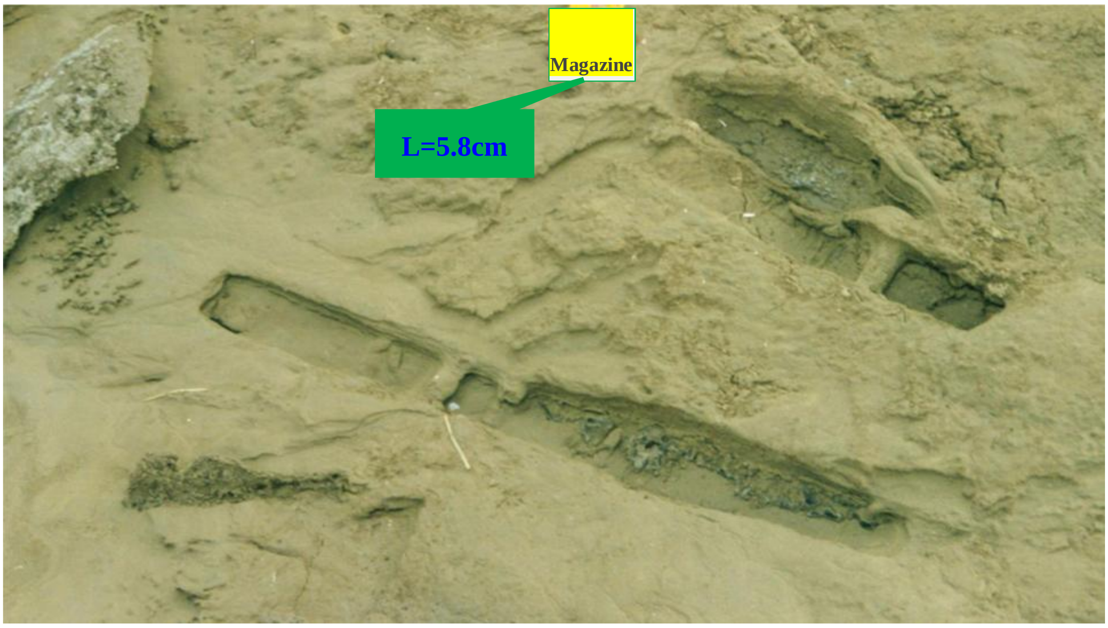

Three ice cast marks formed by an upright ice block. The largest is 56cm in long, 7-9cm wide and 1-3cm deep. There is a small ice fragment in the upper left of the photo.

5. Ice imprint marks

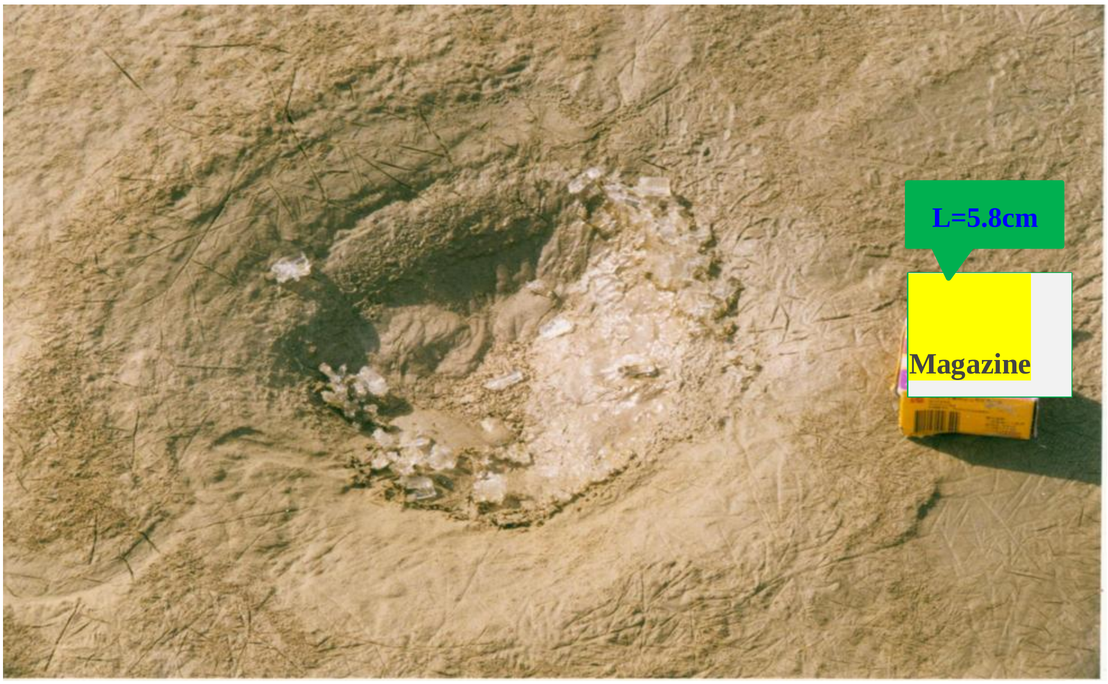

A circular ice imprint mark, it has a pot-like appearance with many small ice fragments on its bottom and ice crystals in its margin. It was formed by being compacted by a circular protrusion on the back side of an ice block.

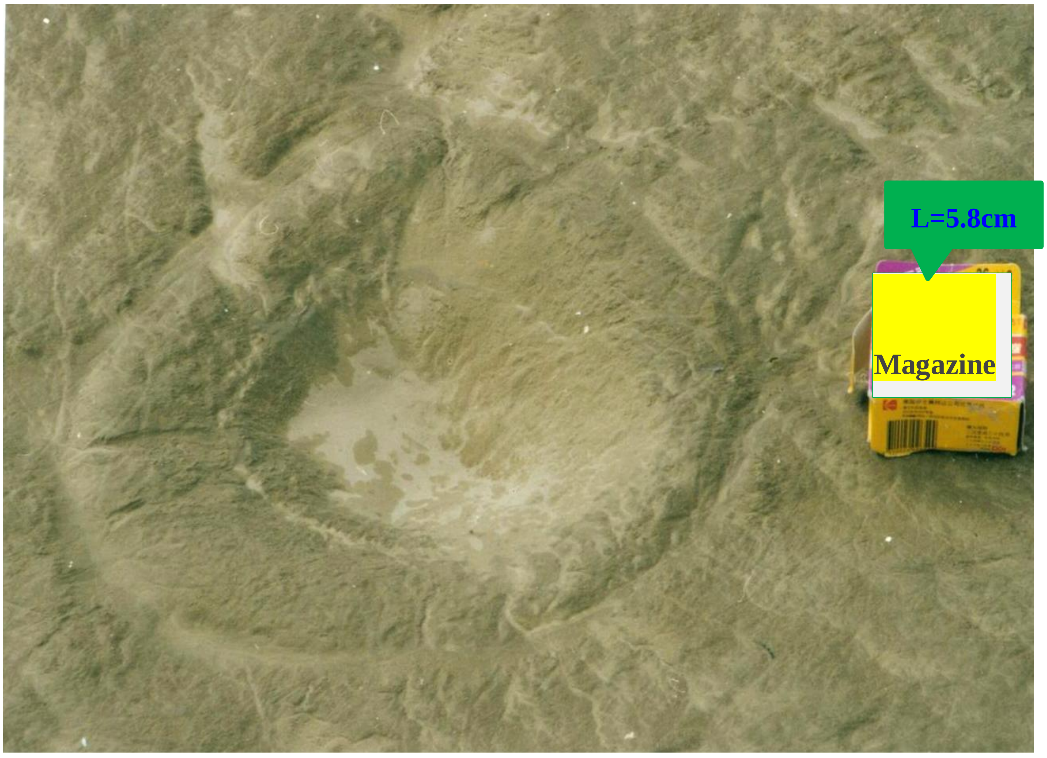

A nearly circular ice imprint mark, it has the appearance of a shallow dish with low and wide circular ridges. It was formed by the compacting action of a circular bulge on the back side of an ice block, and accompanied by some opaque ice crystals.

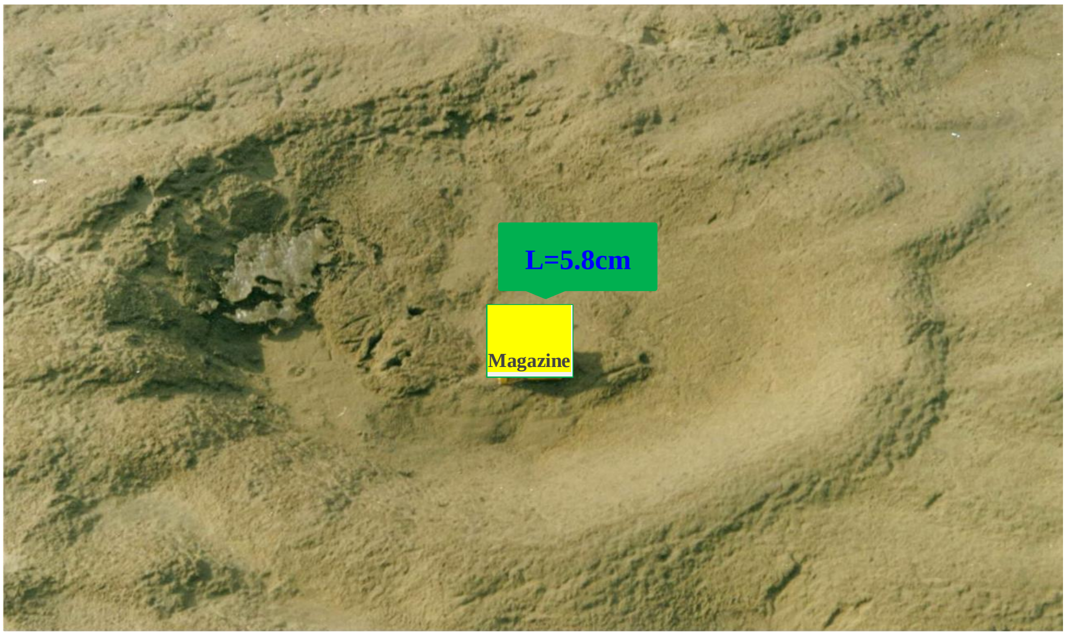

A large circular ice imprint mark, with a shallow, flat dish-like appearance. Small ice fragments can be seen towards the bottom of the upper right corner, it is accompanied by some opaque ice crystals. It is shallow and nearly rounded.

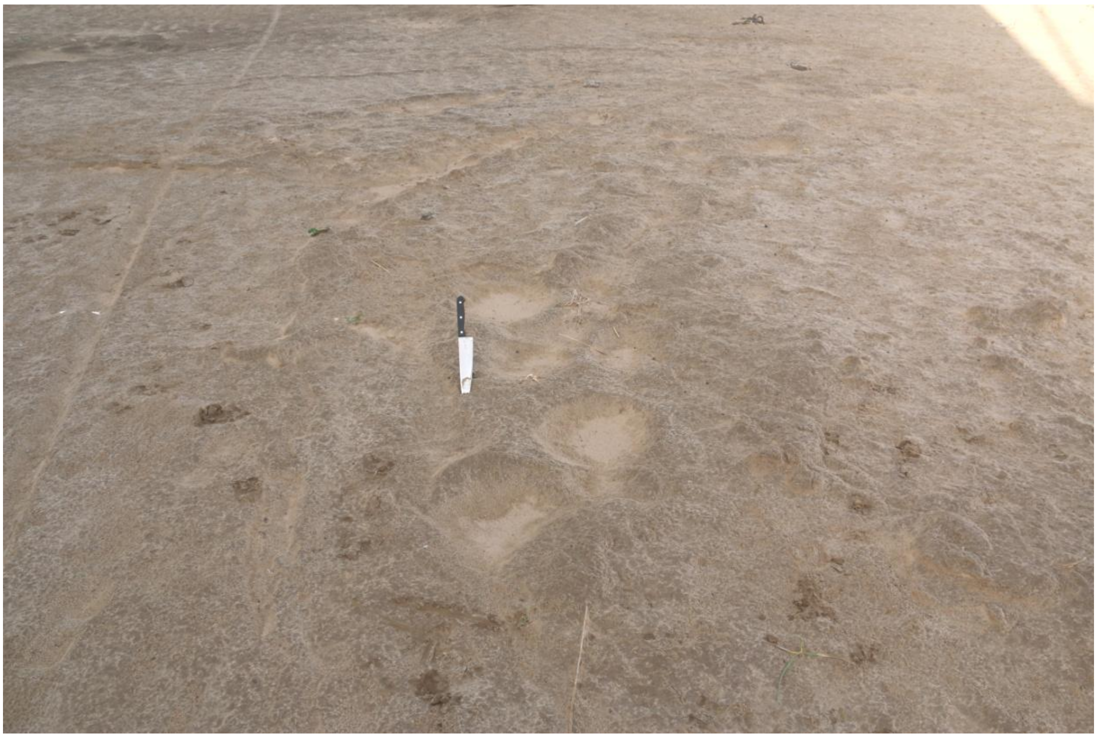

Three relatively perfect ice imprint marks, with a pot-like appearance. They have diameters from 25 to 30cm and depths of between 3 and 6cm. All of the imprints have imperfect ridges around the depression. There are also other, smaller imperfect ice imprint marks around the three. The knife is 14cm long.

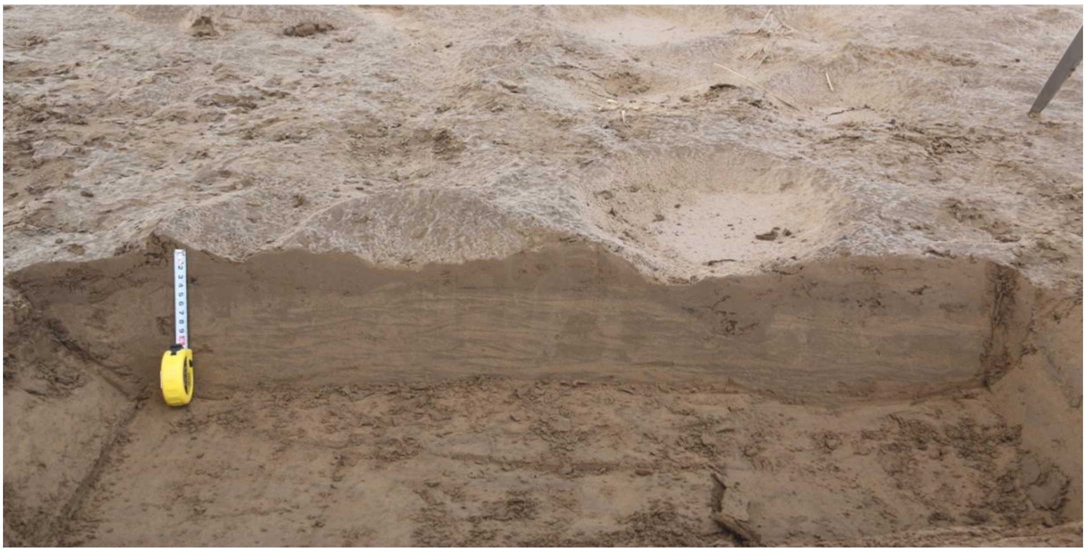

A vertical cross section of the middle and edge of two pot-like ice imprints. The ridges around the depression can be clearly observed. Two sections can be observed: the upper is uniform and massive, lacking any bedding planes, from the result of being heavily disturbed by ice blocks, and the lower section has some small, slightly deformed bedding planes in middle and lower areas. Vertical view of same specimen as above.

6. Ice tool marks

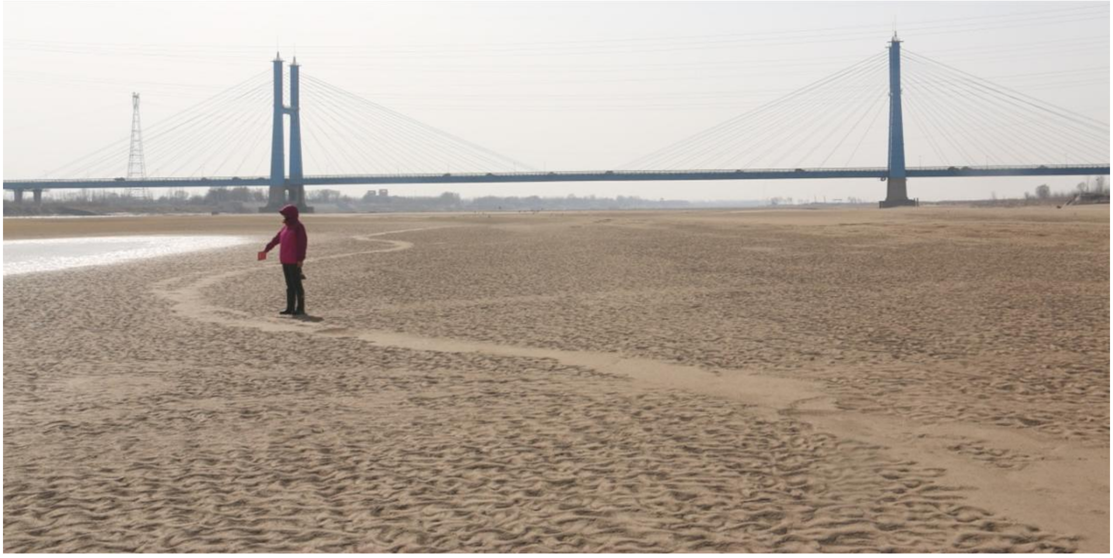

A large scale curved ice tool mark produced by an ice block moving, measuring more than 300 meters in length and between 50cm and 1m in width. Its surface is extremely flat, even and smooth and it has developed on the channel bar and is surrounded by ripples. Shengli Bridge can be seen in the background.

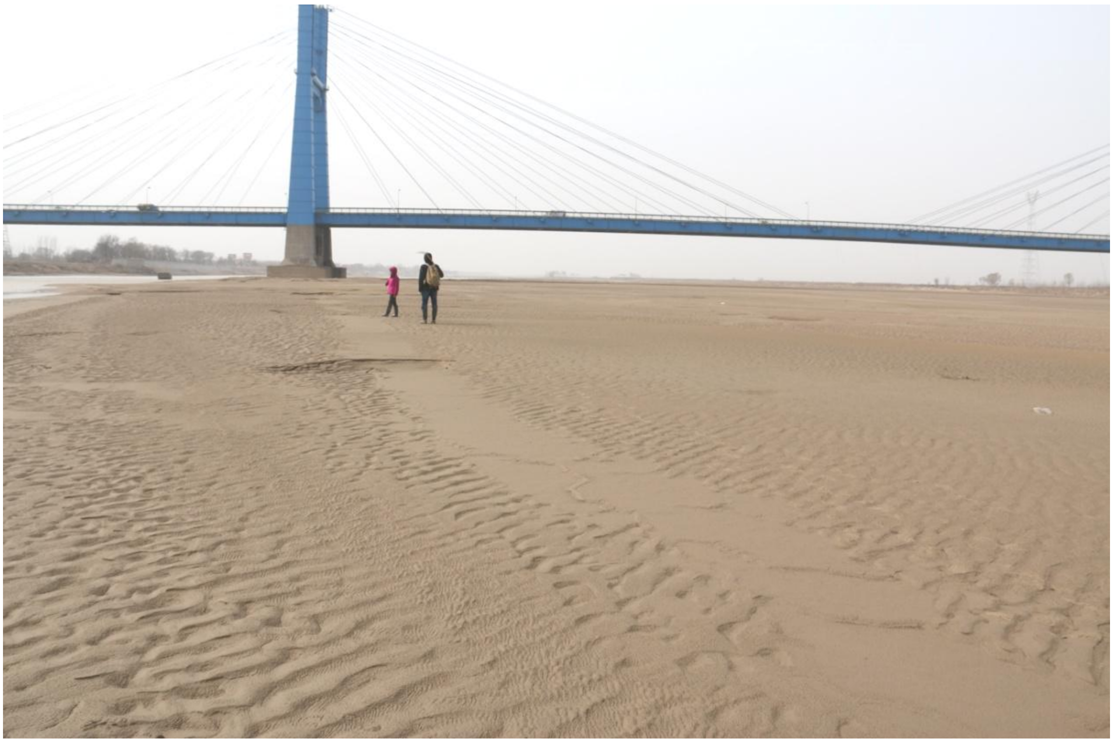

A large scale curved ice tool mark produced by an ice block moving tightly upon the soft deposition surface, measuring more than 100m in length and about 1m in width. It is surrounded by small current ripples. It has developed on the channel bar under Shengli Bridge.

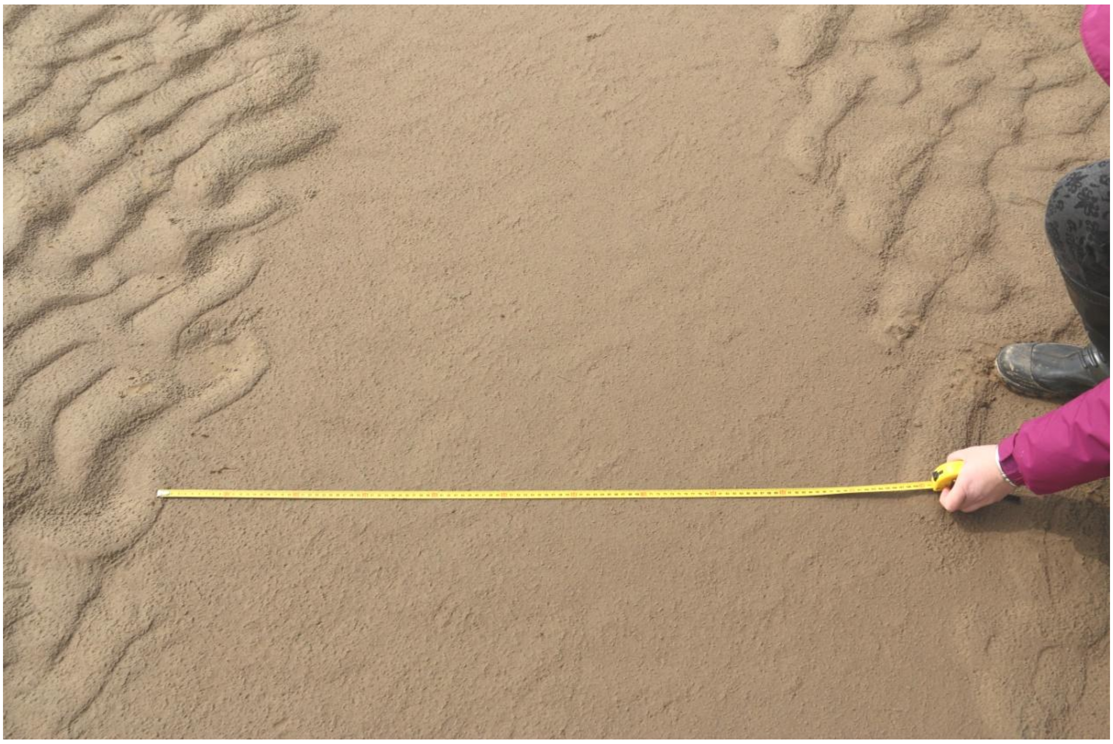

The extremely smooth surface of an ice tool mark surrounded by small current ripples.

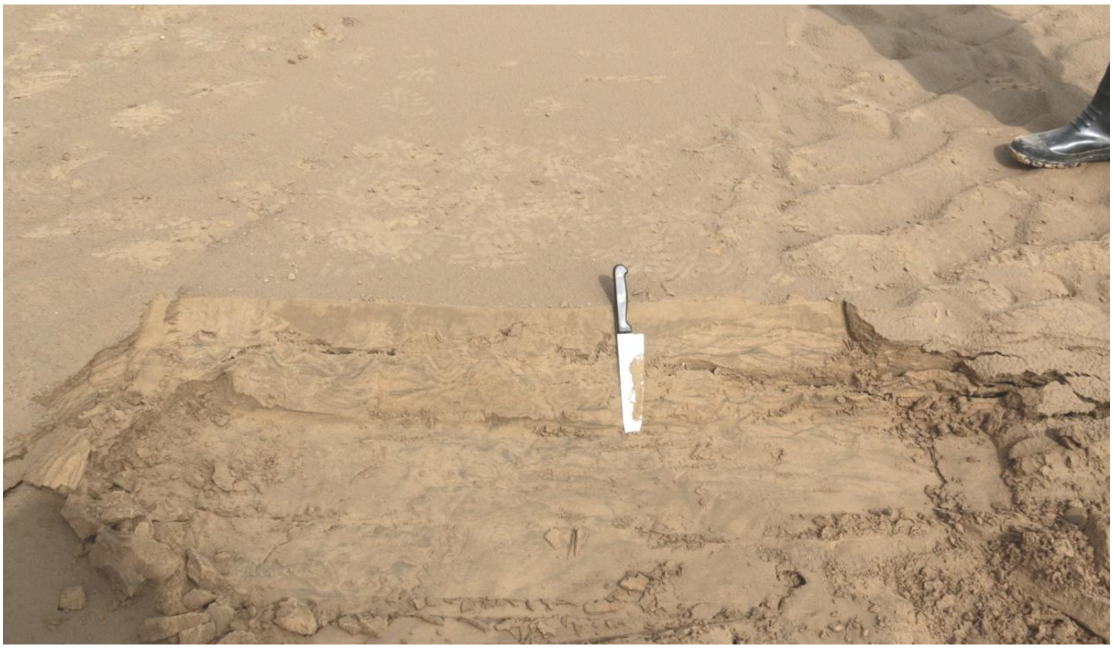

A cross section of an ice tool mark. Some complicated deformed beddings developed within a layer which was disturbed by sliding or moving of an ice block on soft silt sediments. They are surrounded by small current ripples. The knife is 14cm long. Vertical view of same specimen as above.

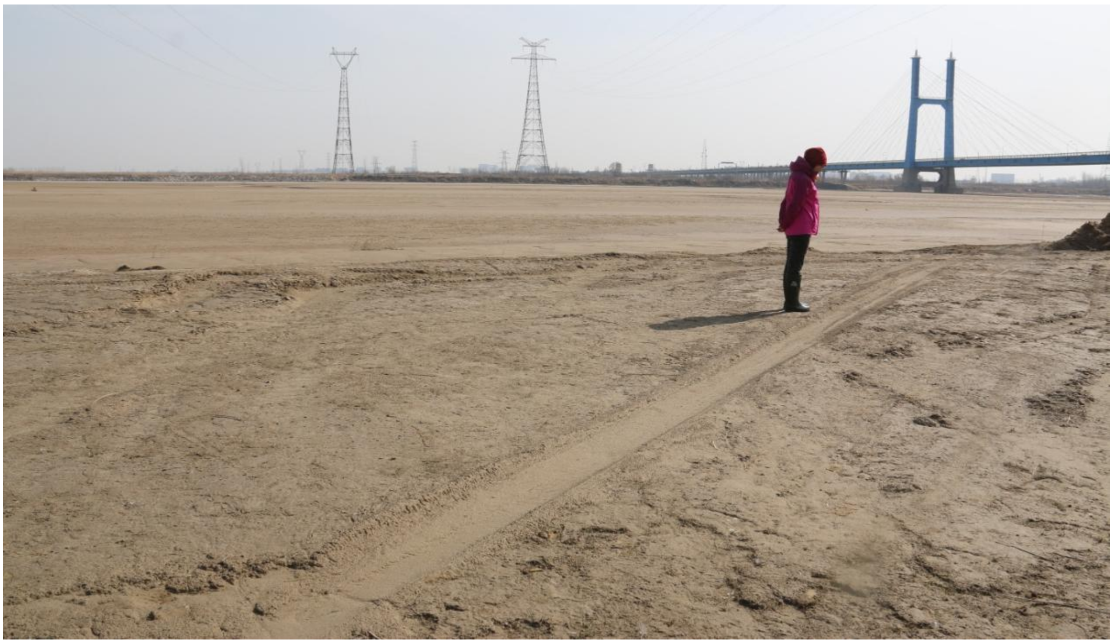

A well-developed ice tool mark produced by the movement of an ice block. It has the appearance of a shallow, circular gully with small, slightly convex ridges on either side. It is 16.3m long, 40-50cm wide and 6-7cm deep, and has developed on the point bar north of Shengli bridge.

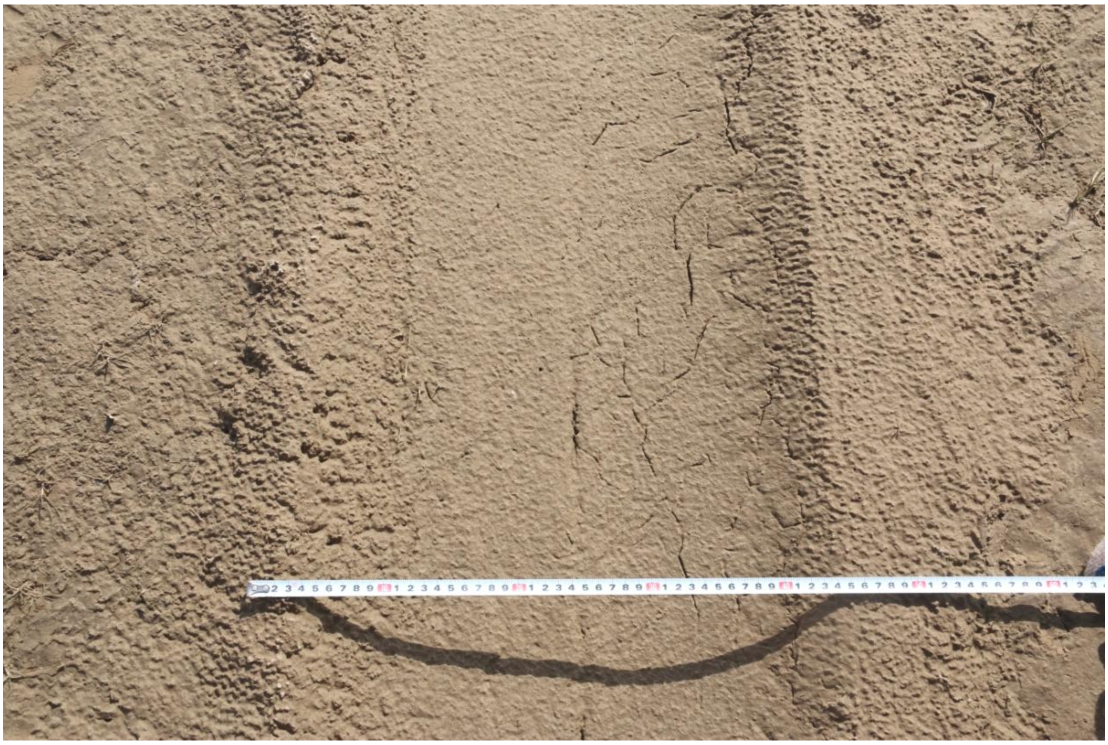

A close-up, top-down view of the aforementioned ice tool mark. Close plan view of same specimen as above.

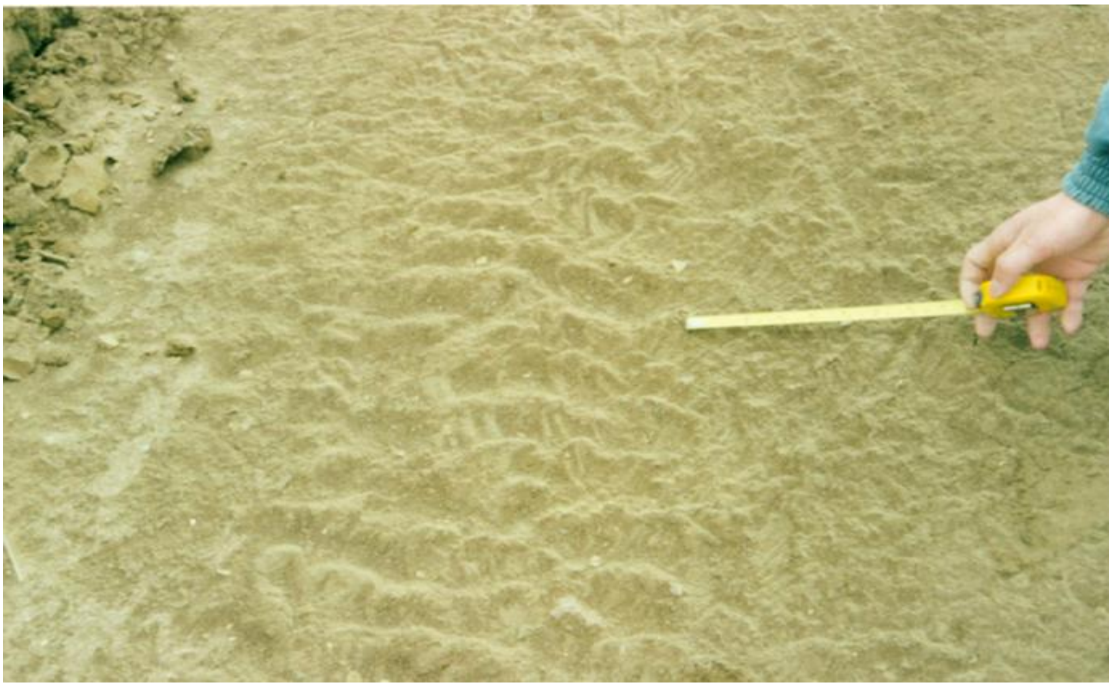

A close-up, top-down view of a small ice tool mark. It has many small folds and its axial surface dips in the direction of the lower course of the Yellow river.

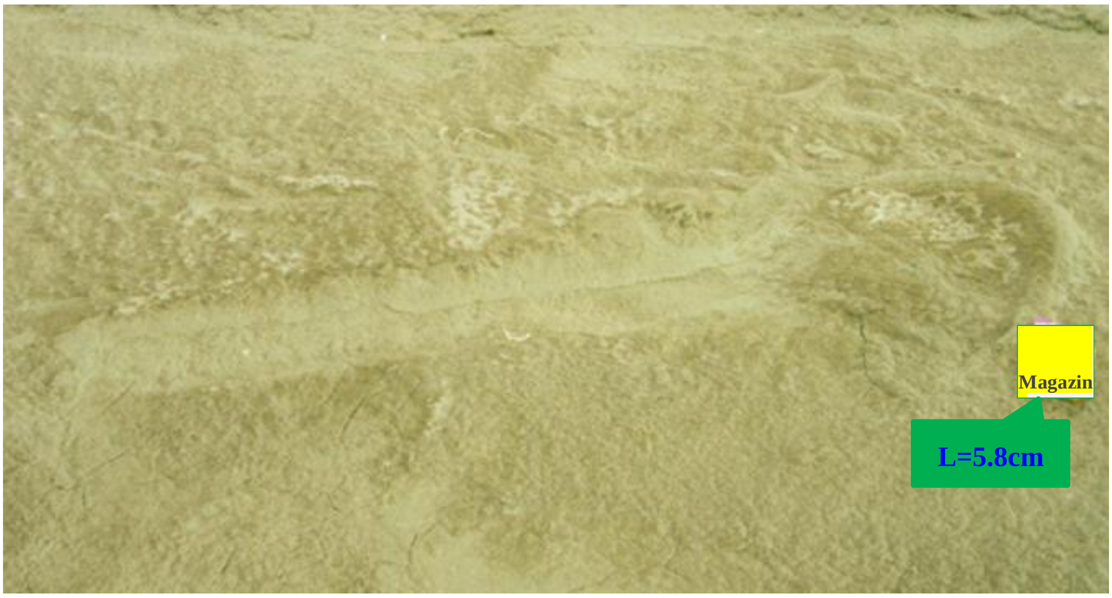

A close-up view of a spoon-like ice tool mark. The straight section has a gully-like shape with a small ridge in the middle and the end expands out into a crescent shape with sharply tubular transversal ridges. The ice block had moved from left to right.

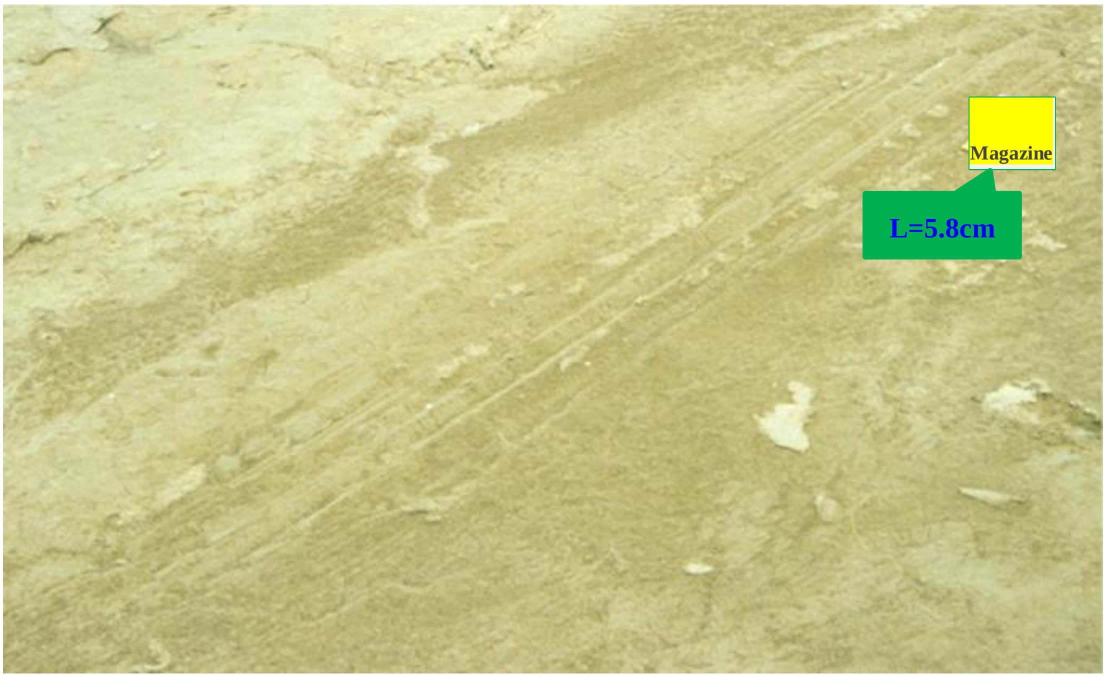

A close-up view of several groove-like ice tool marks produced by the movements of an ice block. It consists of 3-4 straight, triangular grooves formed by a jagged ice block. The ice block had moved from left to right.
