# Supplementary material for: A New Unusual Ice-induced Sedimentary Structure: the Silt Mushroom
Source: Sci Rep. 2016 Nov 11;6:36945. doi: 10.1038/srep36945 (PMC5105128; doi:10.1038/srep36945)
Supplement: Supplementary Information [file srep36945-s2.pdf]

A New Unusual Ice-induced Sedimentary Structure:

the Silt Mushroom

Zhong Jianhua, Ni Liangtian*, Sun Ningliang, Liu Chuang,
Hao Bing, Cao Mengchun, Chen xin, Luo Ke, Liu Shengxin, Huang Leitong, Yang Guanqun,
Wang Shaojie, Su Feifei, He Xuejing, Xue Yanqiu

School of Geoscience, China University of Petroleum, Qingdao 266580, China

A New Unusual Ice-induced Sedimentary Structure:

Chinese flower-bun-like structures

Appendix Ⅱ

Supporting information
Accompanied or associated ice-induced and
ice water sedimentary structures with silt mushroom

(All photos were taken by Ph. D. Ni Liangtian and all the photos were taken in the lower course of Yellow river; The numbers on the tape n all the photos indicate decimeters.)

In order to help the readers to more easily and exactly understand the silt mushroom and its genesis, it is necessary to provide some photos that have the important information of the formation background of silt mushrooms and accompanied or associated ice-induced and ice water sedimentary structures with silt mushrooms. Certainly most, most of them (at least over 18 kinds from the second to the nineteenth) are of new sedimentary structures that have never been documented up to now in any books or magazines. All the photos herein were taken from 1996 to the present.

* These authors contributed equally to this work. Correspondence for materials should be addressed to Ph. D. Ni Liangtian.
E-mail:382938098@qq.com

7. Ice-induced dome or silt mud mound

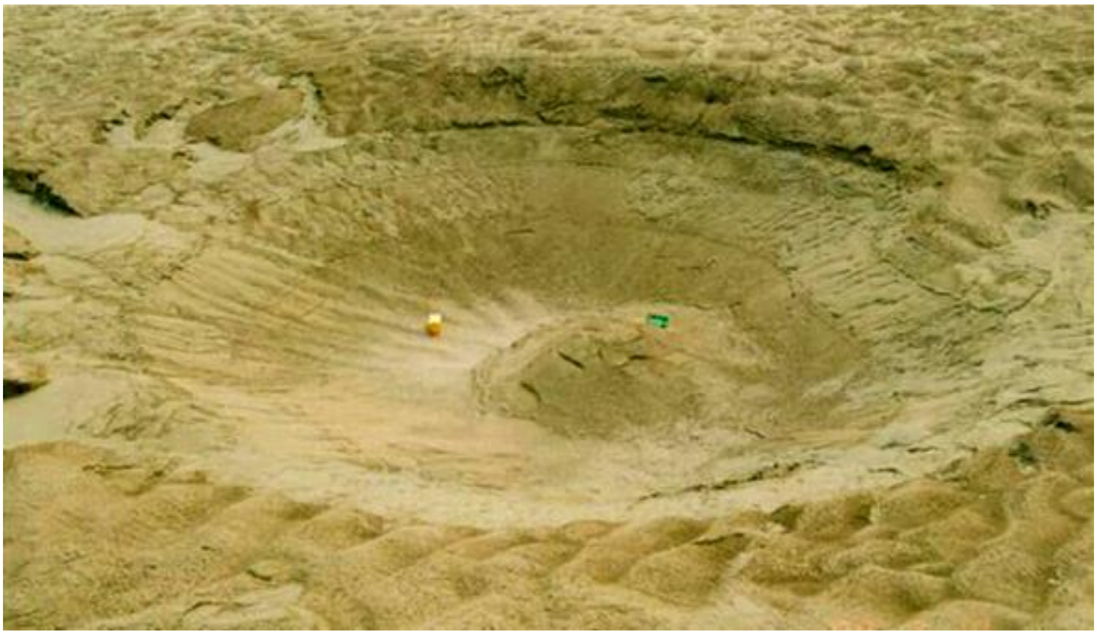

A large scale ice-induced dome or a large scale ice-induced silt mud mound. It is shaped like a dome with gently dipping walls and was formed by water leaking from a hole in the middle of the ice layer like a roof over the circular depression. It has clear ice line marks and a great deal of ice crystals around its walls.

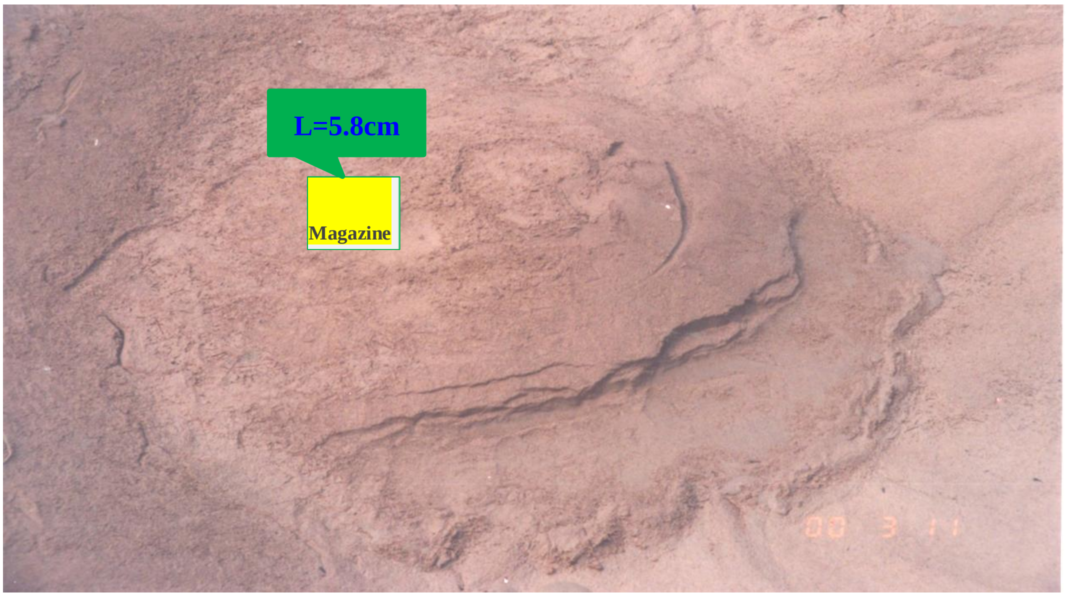

A close-up view of the aforementioned ice-induced dome/large scale ice-induced silt mud mound. The wall on the bottom-right hand side has collapsed due to liquefaction caused by wave action.

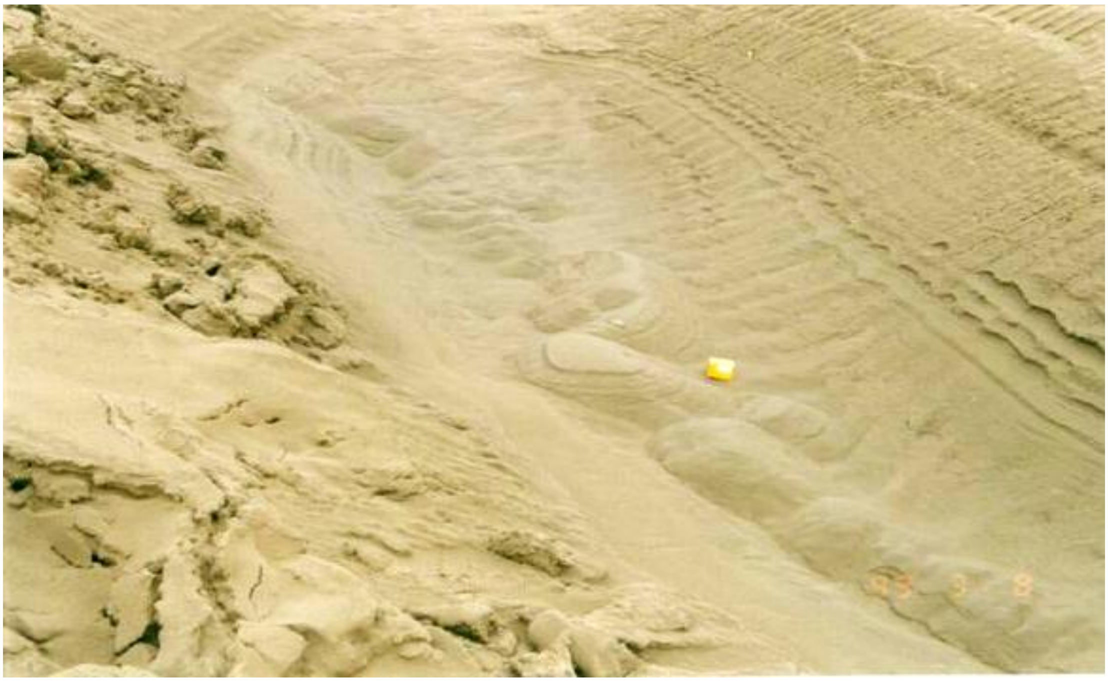

A group of ice-induced domes/ice-induced silt mud mounds. They vary in size from less than 10cm to about 50-60cm in diameter, and measure between 3-5cm and more than 10cm in height. One unusual feature is the 2-3 water lines marks on their walls. They developed in a long depression and were formed by water leaking from a series of holes in the ice layer over the long depression. The yellow box is about 5.8cm long.

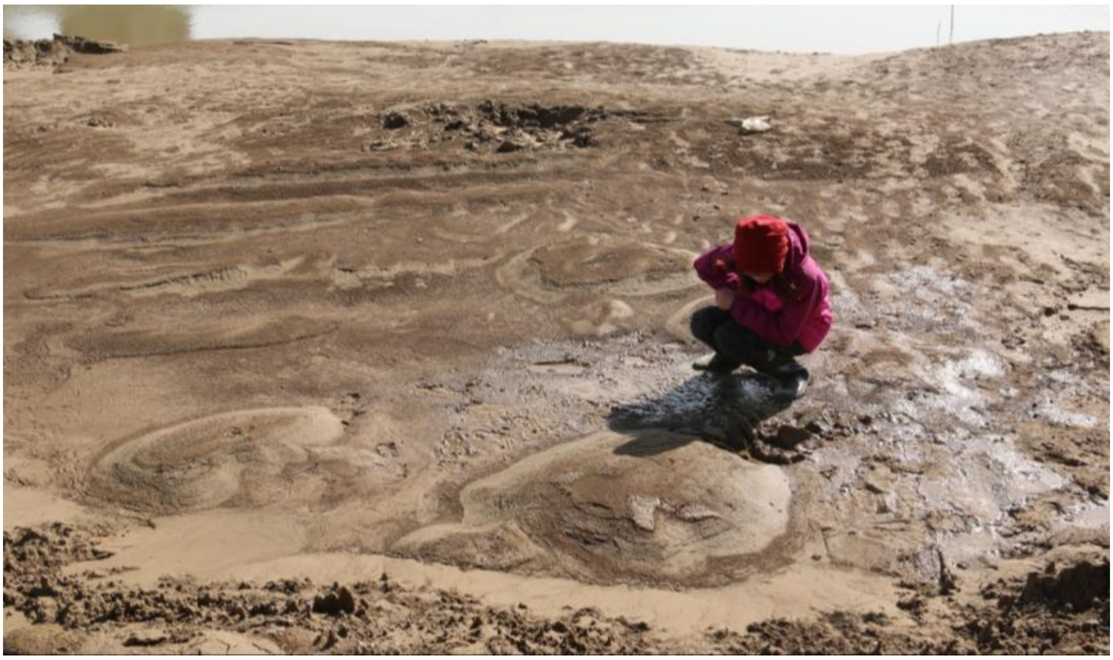

About 10 ice-induced domes/ice-induced silt mud mounds. Most of them have dome-like shapes, though one has a more elongated shape (the one on the upper left side). They developed in a shallow and flat depression, around them are a great deal if ice-induced sedimentary structures such as ice crystals, ice-induced bean-shaped structures and so on.

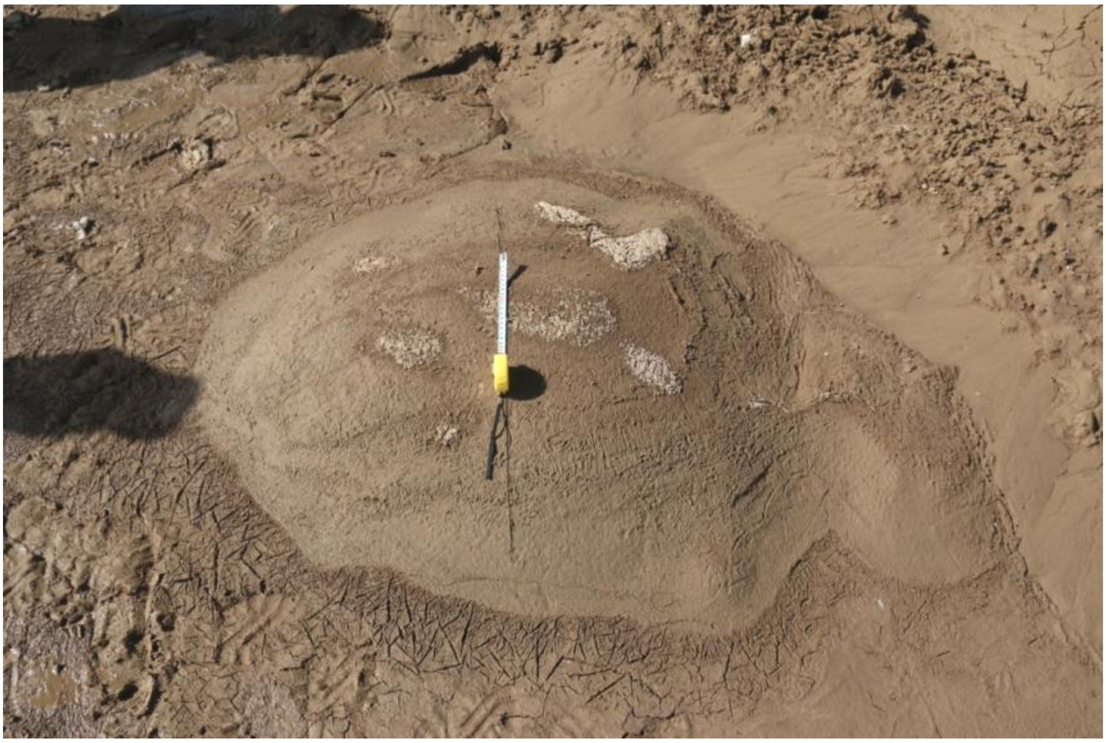

A close-up view of the largest ice-induced dome/large scale ice-induced silt mud mound, it measures 110cm in diameter and 19cm in height. It has an enlarged base on the nearer side resulting from slumping caused by water-saturated mud. On and around the surface of the left and lower sides, a lot of ice crystal marks can be seen, and many micro deltas can be seen on the upper right hand side, having been formed by ice water deposition.

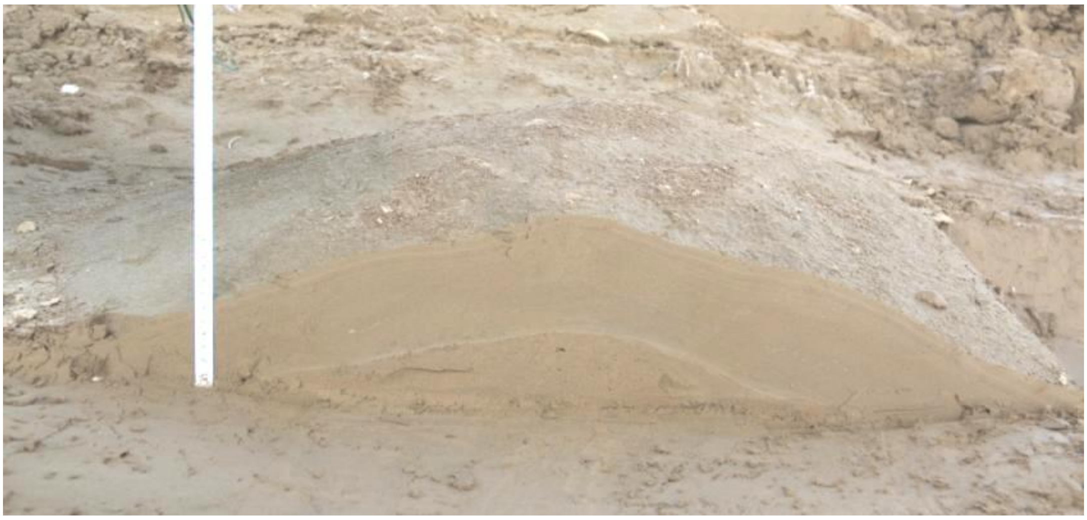

A close-up view of the vertical section of the aforementioned ice-induced dome. The similar shape of both the symmetrical convex bedding planes and the surface of the dome can be observed.
Vertical view of same specimen as above.

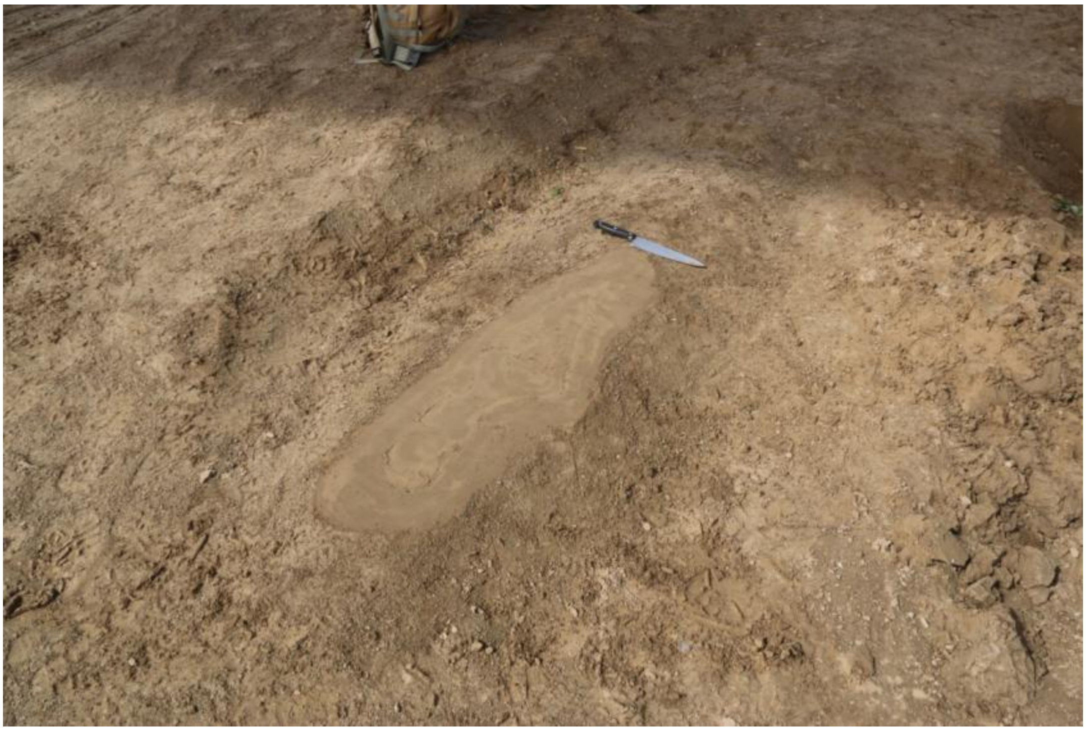

A plane cross section of a long ice-induced dome shaped like a loaf of bread. Within it there were some irregular long circular bedding planes. The section is from the horizon about 5cm above the surrounding sediment surface. The knife is about 14cm long.

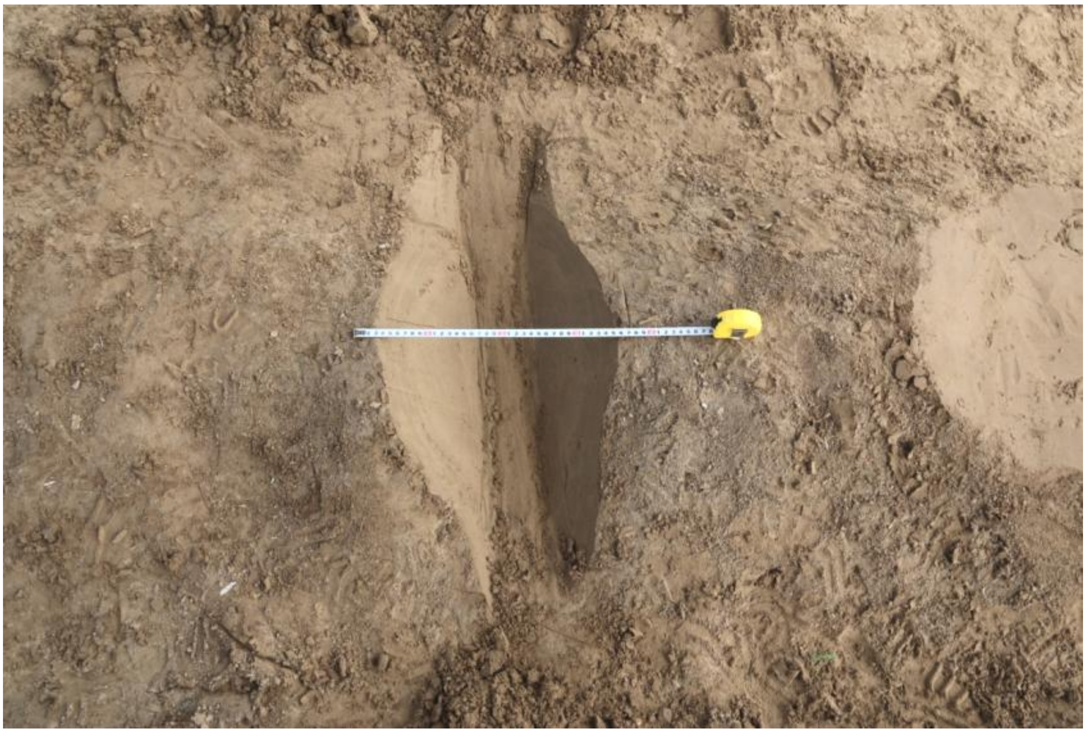

Two highly inclined cross sections of the long ice-induced dome.

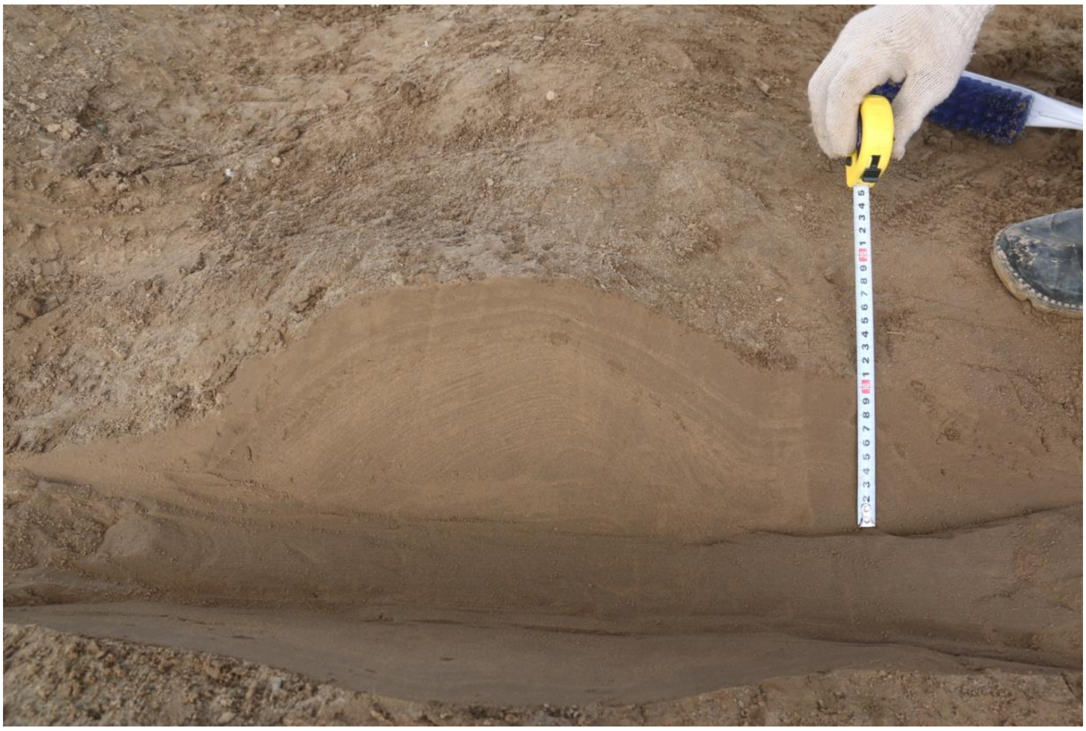

A nearly vertical cross section of the long ice-induced dome. The convex bedding planes may be divided into two sections: the outer part consists of convex bedding planes, which are the same as the shape as the mound. The internal Section consists of discontinuous convex bedding planes that have drifted from the lower right to the upper left, resulting from the movements of the ice block and its leaking deposits. Vertical view of same specimen as above.

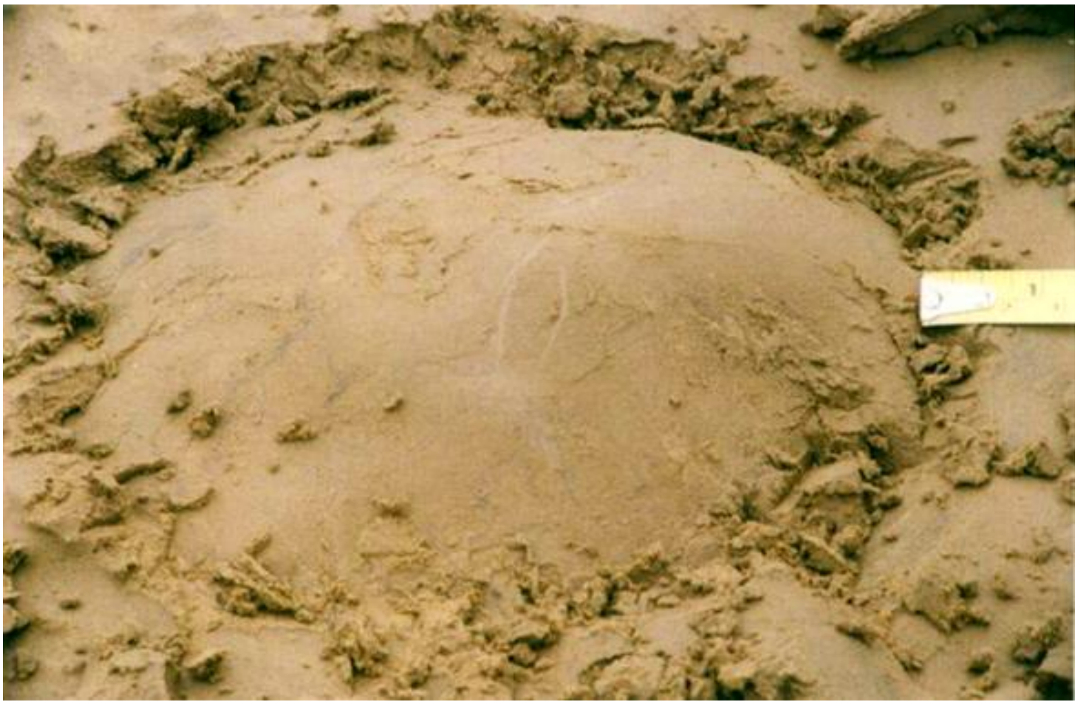

A close-up view of a large scale ice-induced dome/large scale ice-induced silt mud mound. It was frozen at the time and so had a smooth surface when I was dug out.

8. Ice-melt dome or ice-melt hollow dome

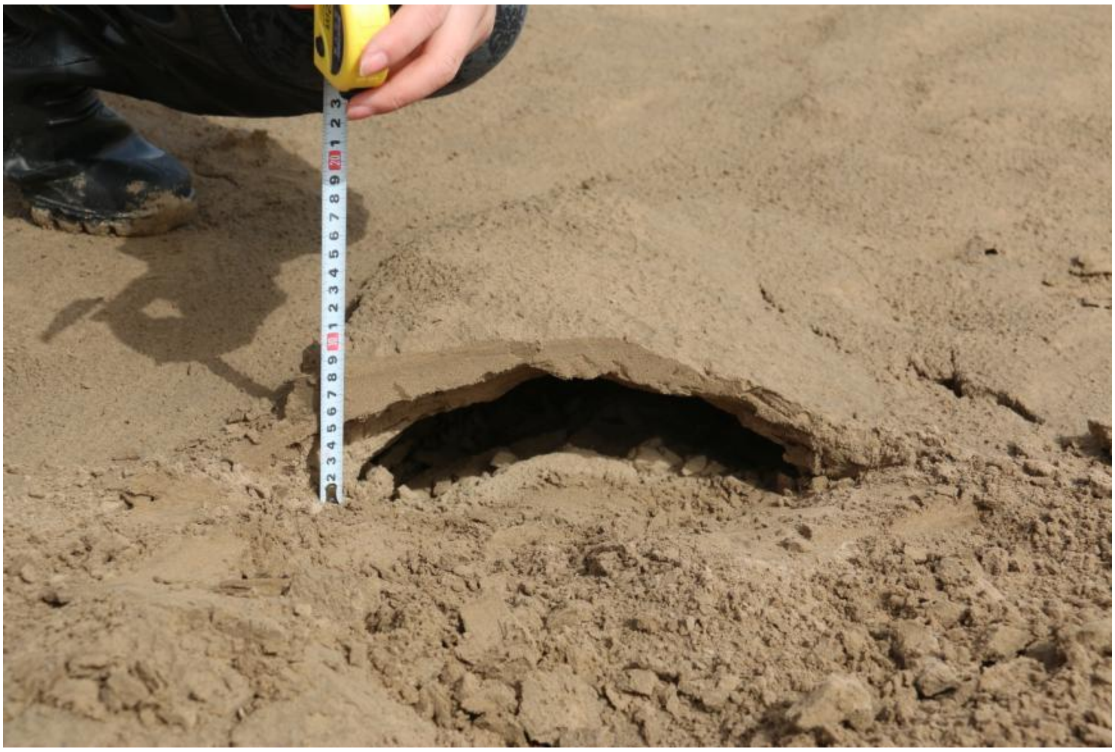

A ice-melt hollow dome. The hollow space is formed by ice block melt completely.

9. Frozen bubbles

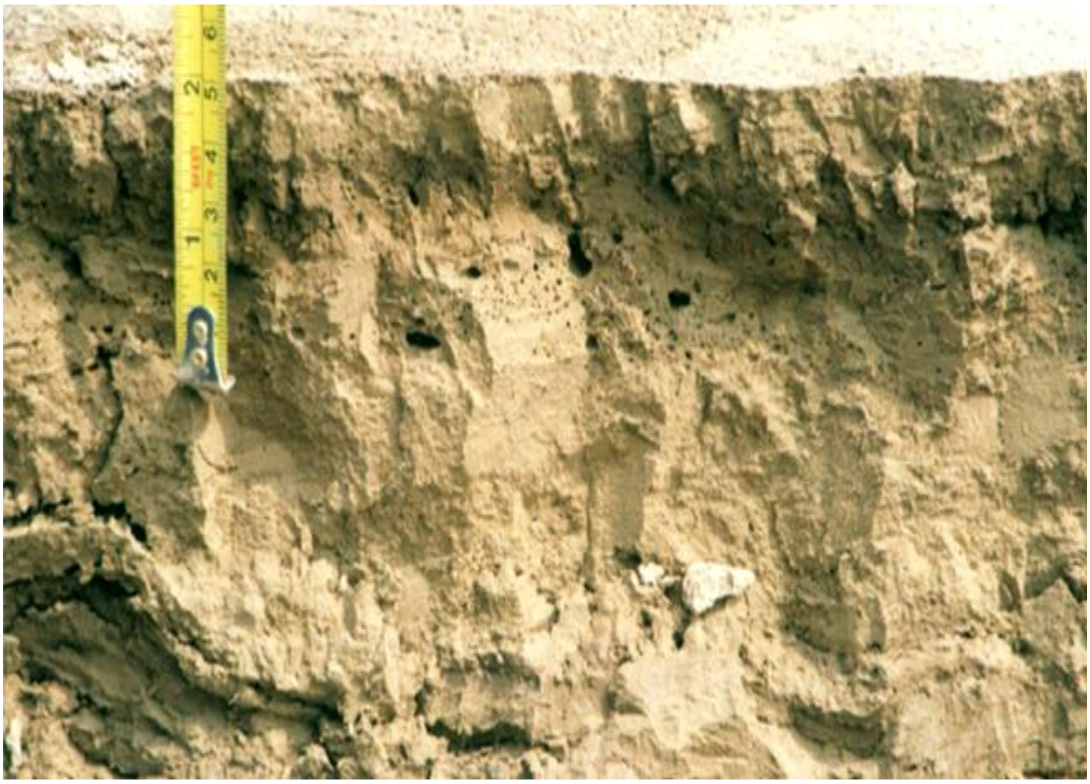

A series of frozen bubbles and frozen fissures accompanied by a great deal of nearly vertical frozen fissures. They developed in the wet muddy silt layer of a point bar and were formed by

freezing and thawing cycles.

10. Ice melt slump pots and depresses

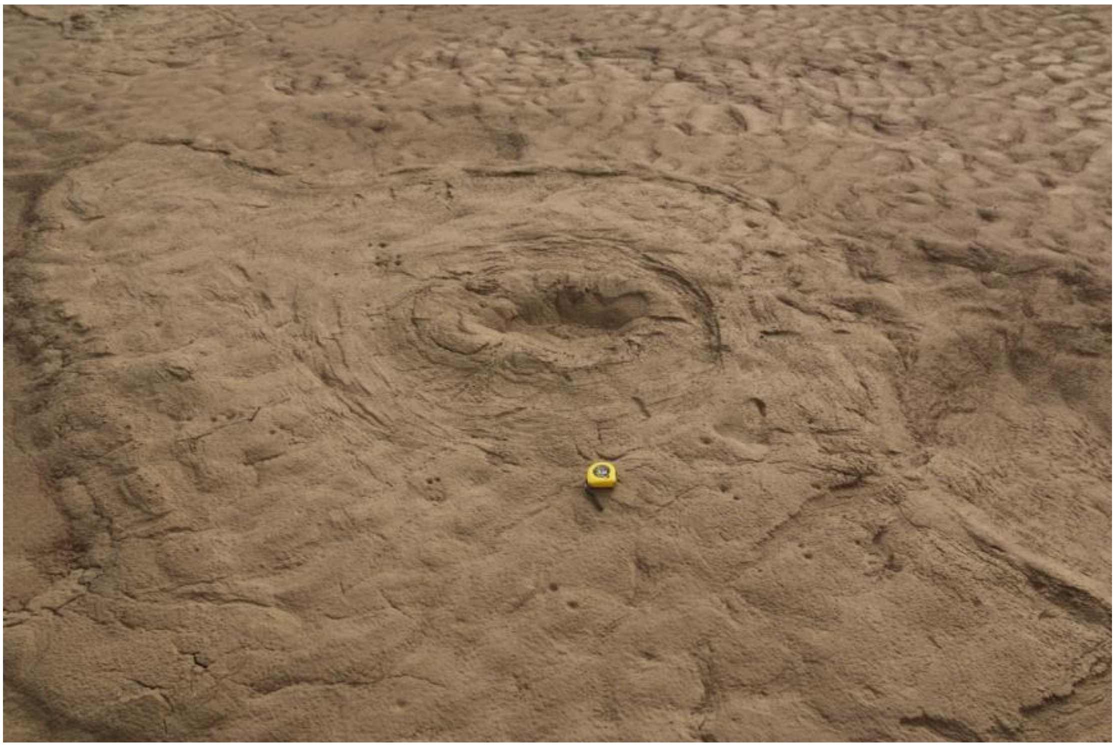

A relatively circular ice melt slump pot like a navel, with a great deal of nearly circular slump fissions. The melt ice block was just in the middle of the pot.

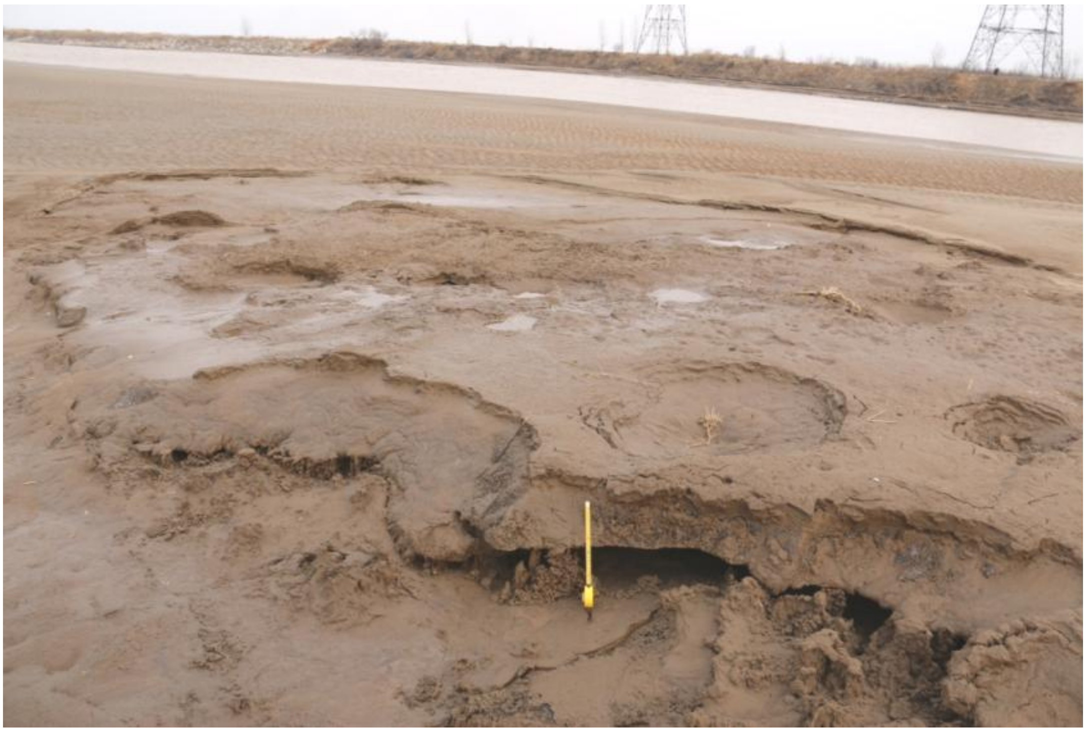

Several ice melt slump pots with 50-80cm diameters and depths of 10-15cm. They are nearly circular in shape, and are formed by collapses resulting from ice melts.

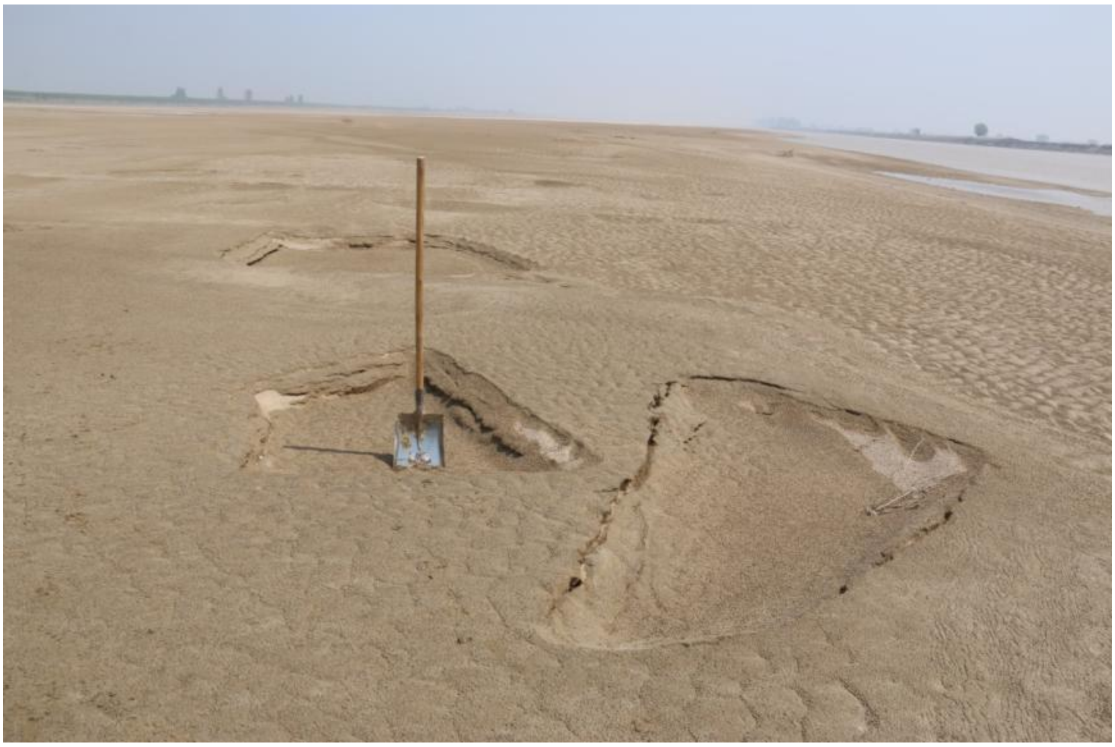

Three polygonal ice-melt slump depressions developed closely. Behind them there are also many other polygonal ice-induced slump depressions（The dark-colored parts）with different shapes and sizes. The standing shovel is about 140cm in length.

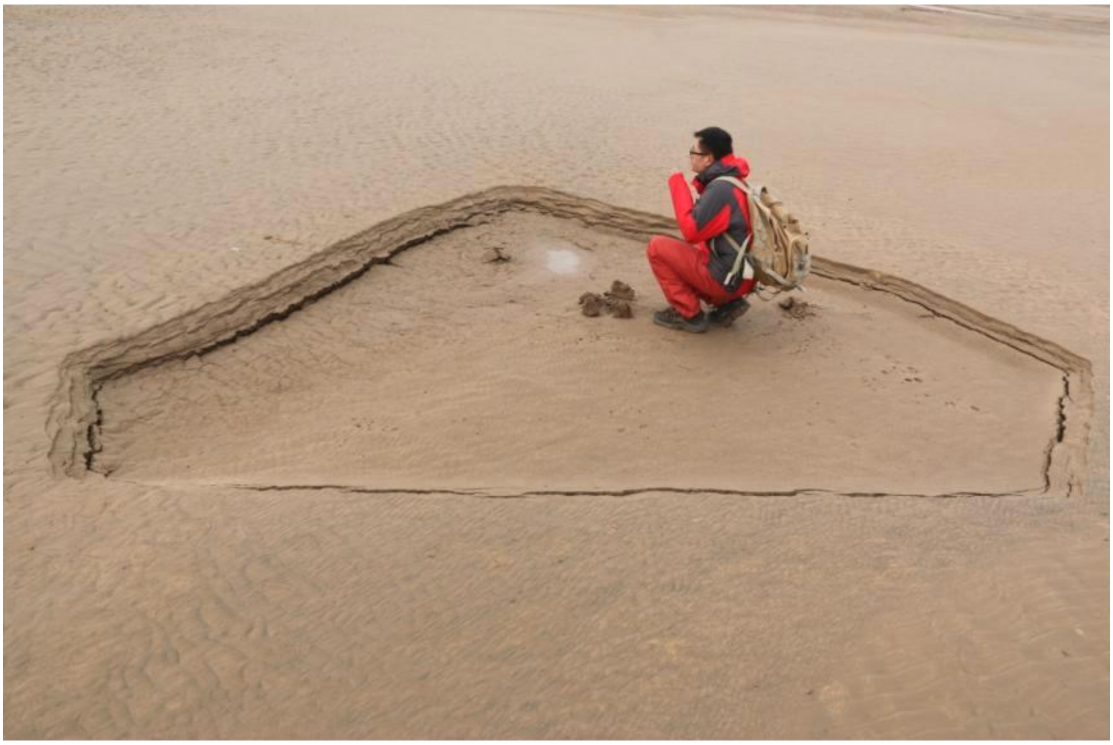

A nearly pentagonal ice-melt slump depression with nearly upright walls on some sides and dip walls forming steps on the left-hand side.

The person appearing in the figure is the co-fauthor Sun Ningliang.

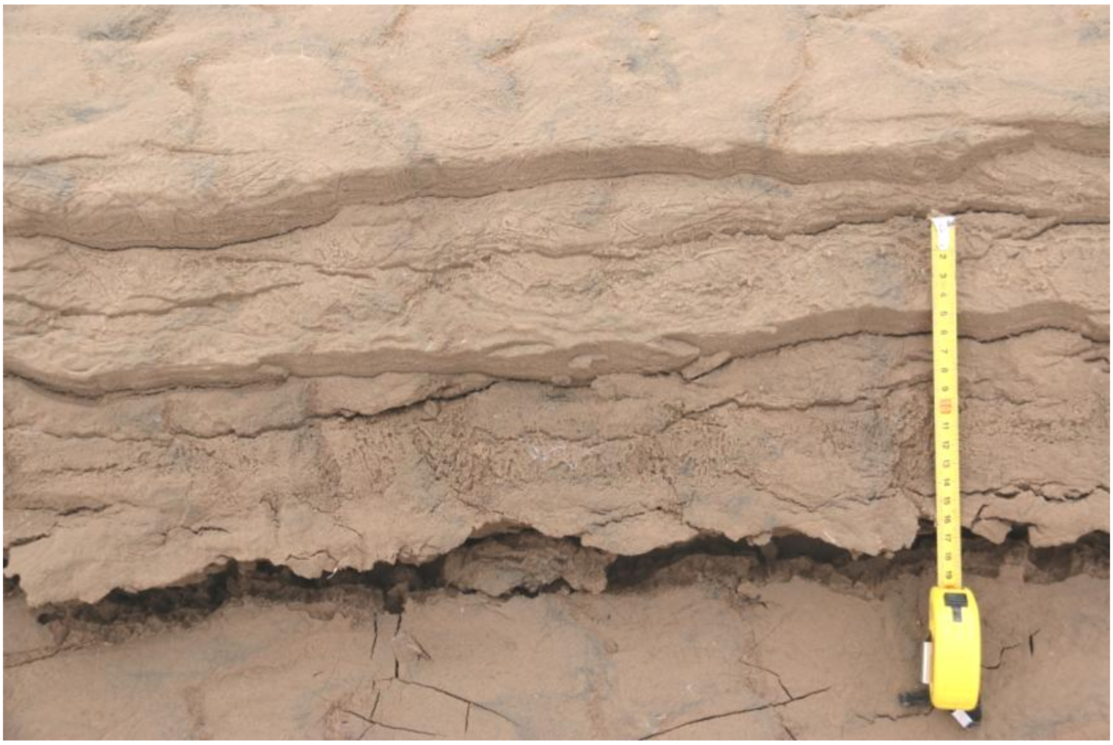

A slump dipping wall of the above depression, formed by two slump steps with a few slump fissions and a great deal of ice crystals. Vertical view of same specimen as above.

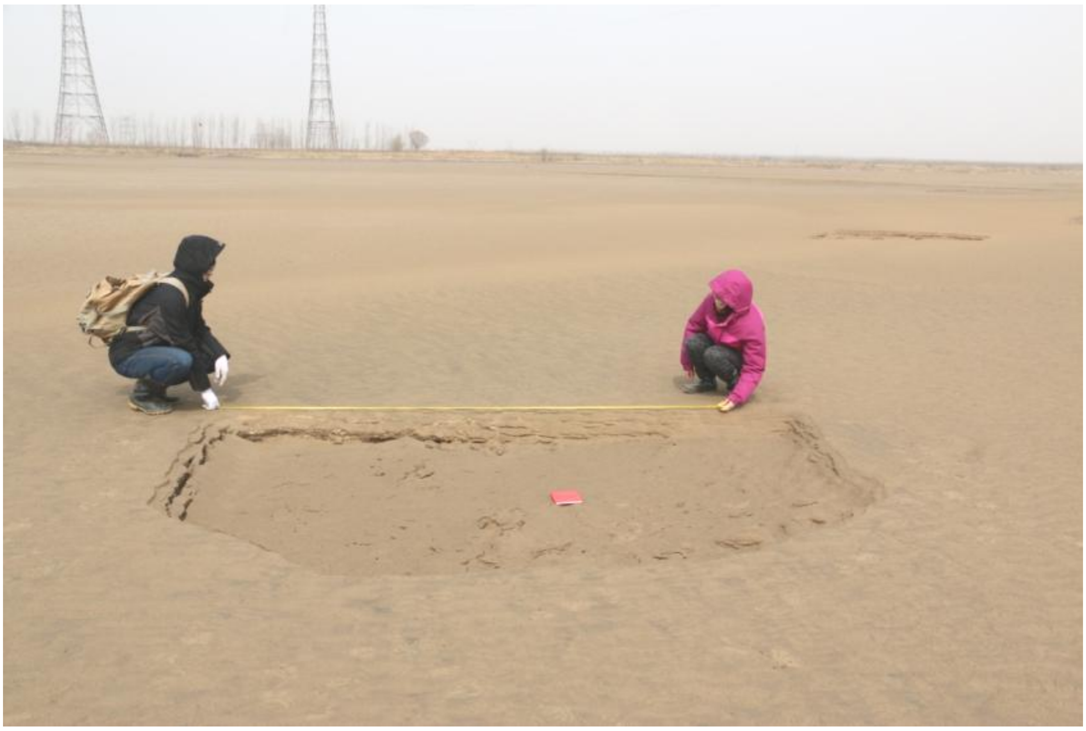

A nearly pentagonal ice-melt slump depression with nearly upright slump walls on some sides, and an inclined wall on the left-hand side.

The person appearing in the figure(left one) is the co- author Liu Chuang.

The person appearing in the figure(right one) is the co-author Cao Mengchun.

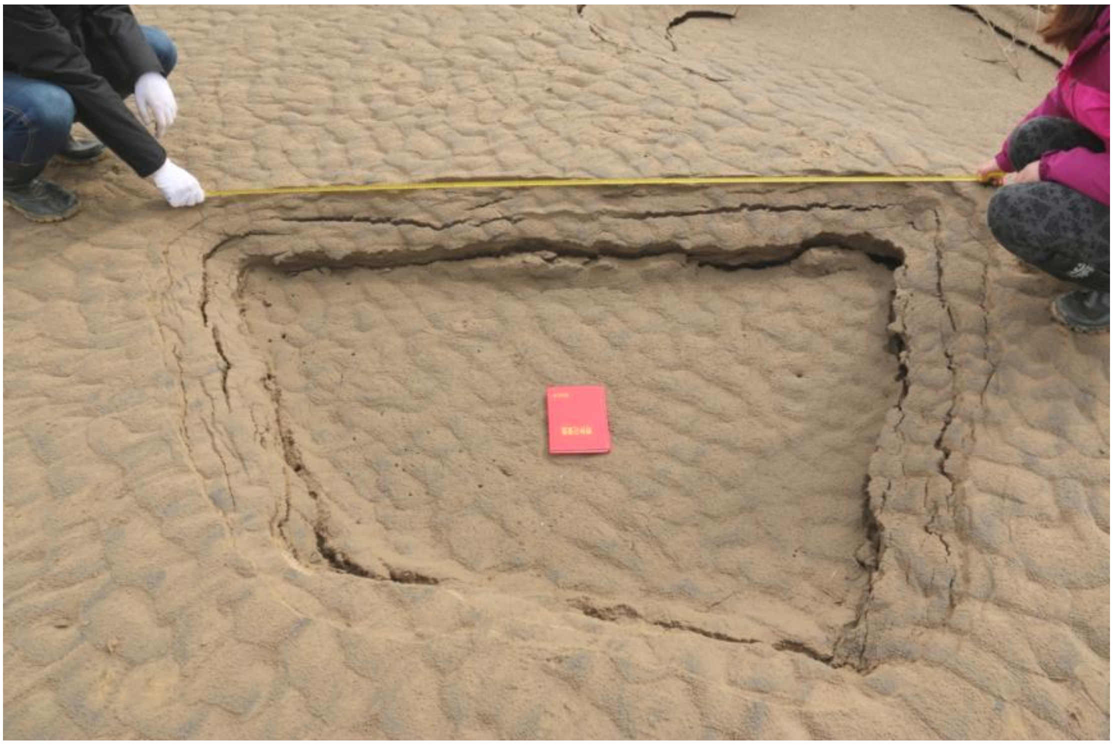

A medium quadrilateral ice-melt slump depression with inclined slump walls and many nearly parallel slump fissures on its wall. There are a lot of same current ripples on the bottom as that of the surrounding. this reveals that the bottom of the depression remained the same height as the surrounding and was acted by the same flow.

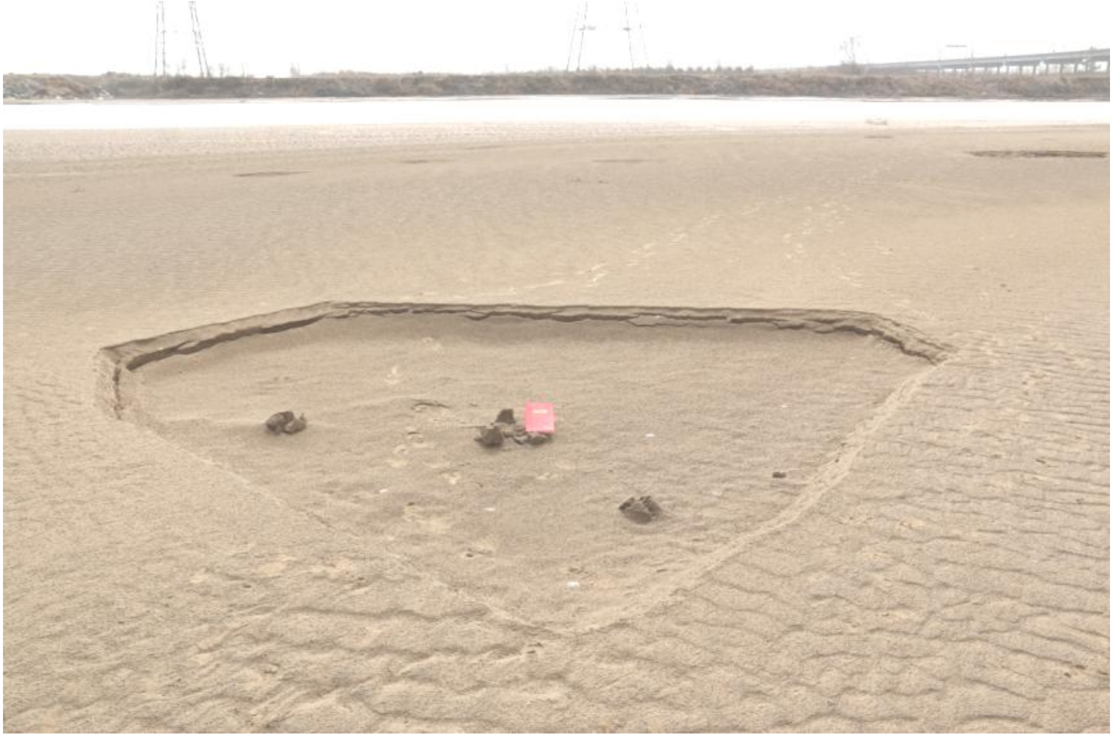

A hexagonal ice-melt slump depression with three ice-induced flower bun-like structures on the bottom. It is surrounded by current ripples.

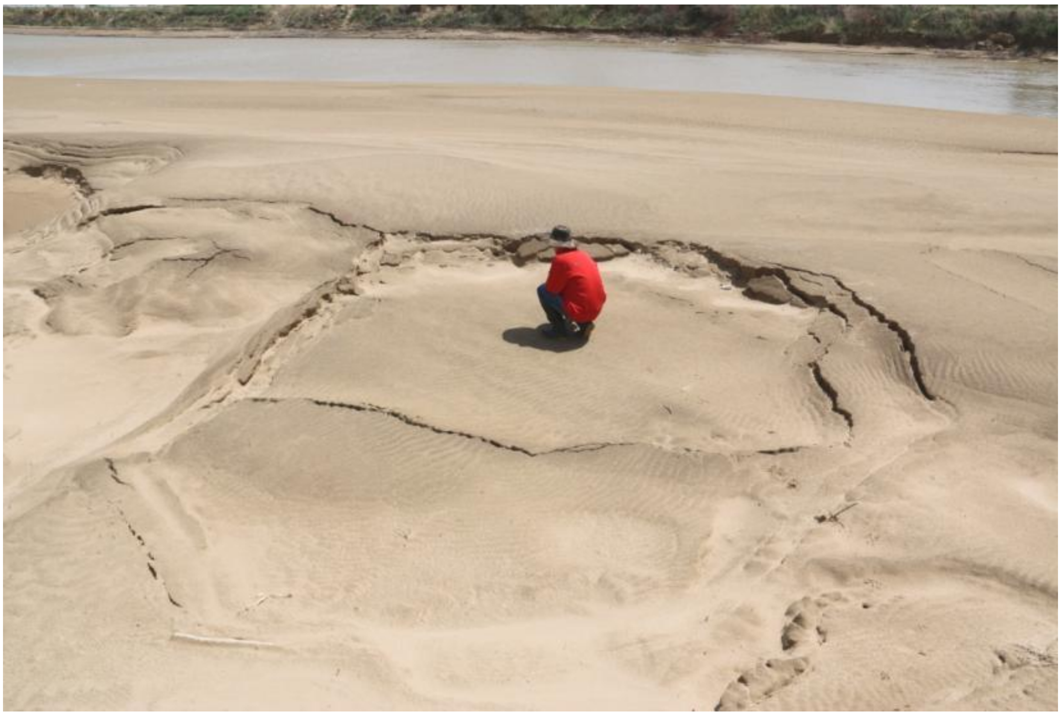

A large irregular hexagonal ice-melt slump depression with an uneven bottom and irregular slump walls.

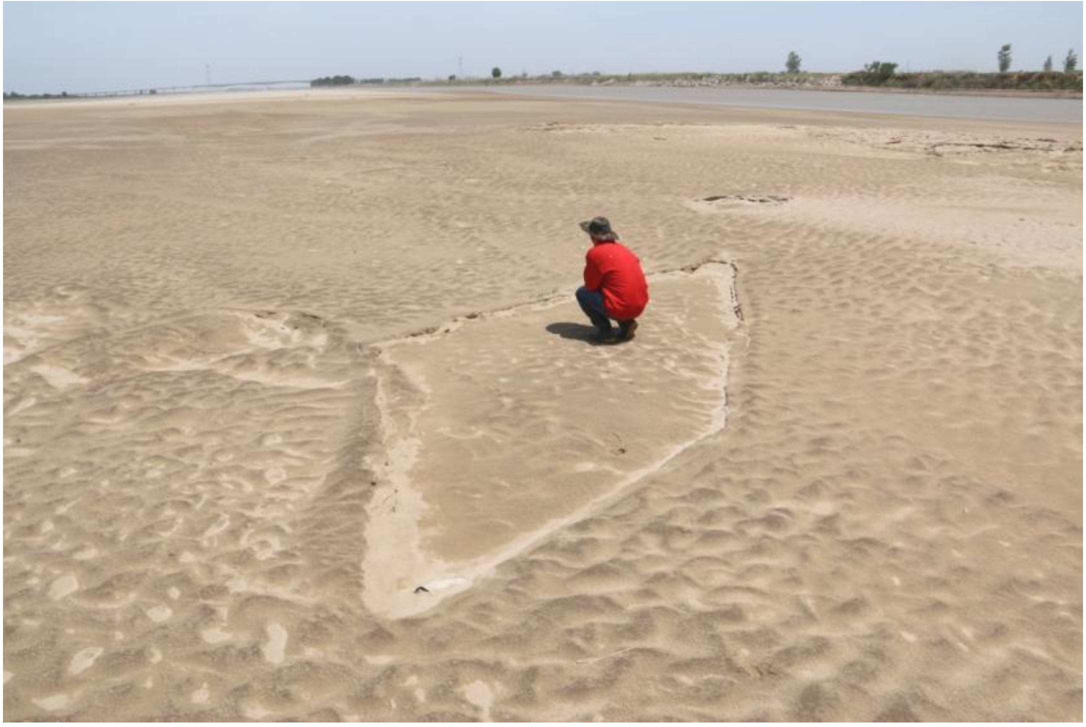

An irregular shallow rhombic ice-melt slump depression.

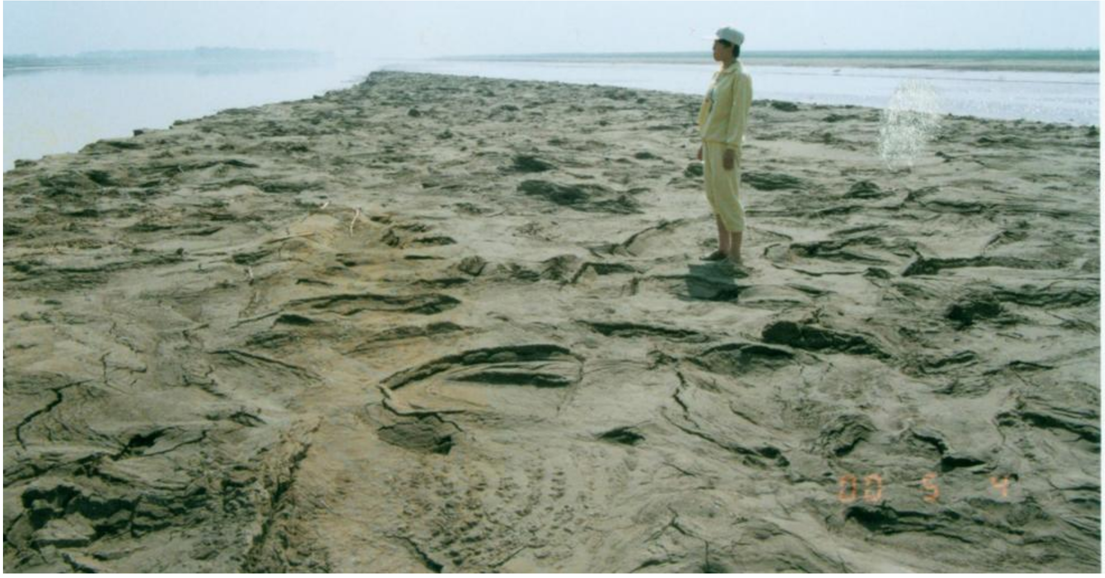

A group of complicated depressions with other ice-induced sedimentary structures developed on the long and narrow channel bar. This made the channel bar being extremely uneven.
The person appearing in the figure is the co-author Cao Mengchun.

11. Frozen sand ridges

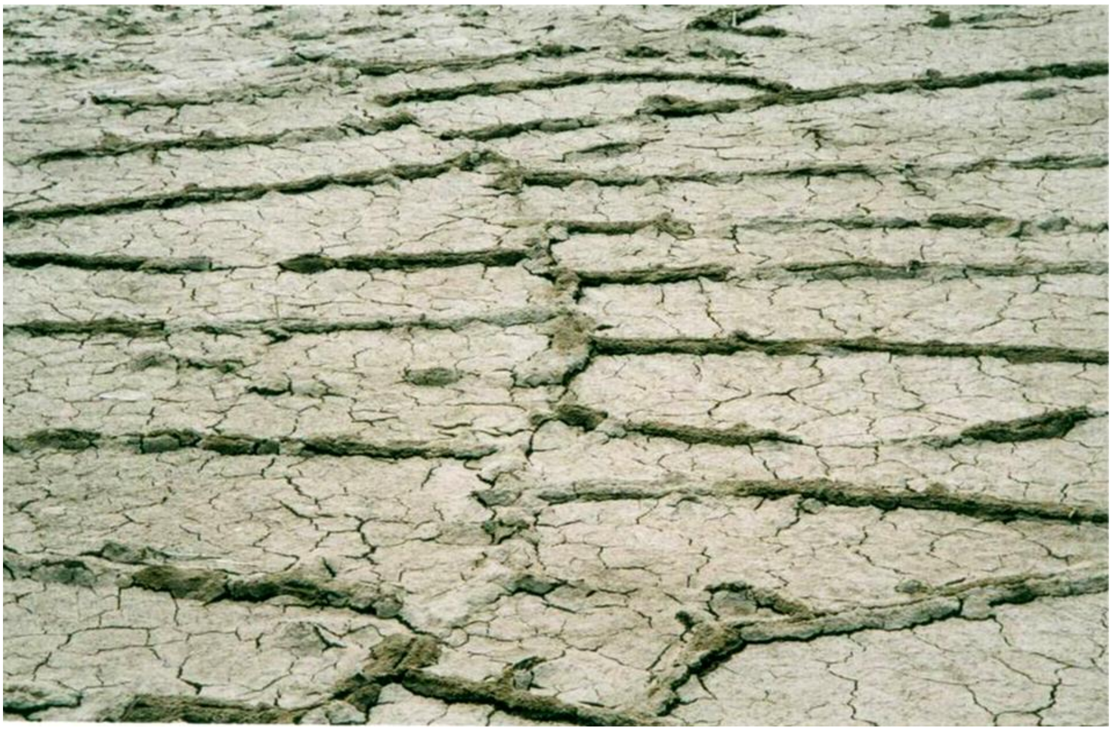

A group of frozen sand ridges with rectangular shapes. They have narrow, convex shapes with widths of 4-6cm and heights of 1-2cm, and fissions in the middle of them, accompanied by a great deal of small cracks in the mud.

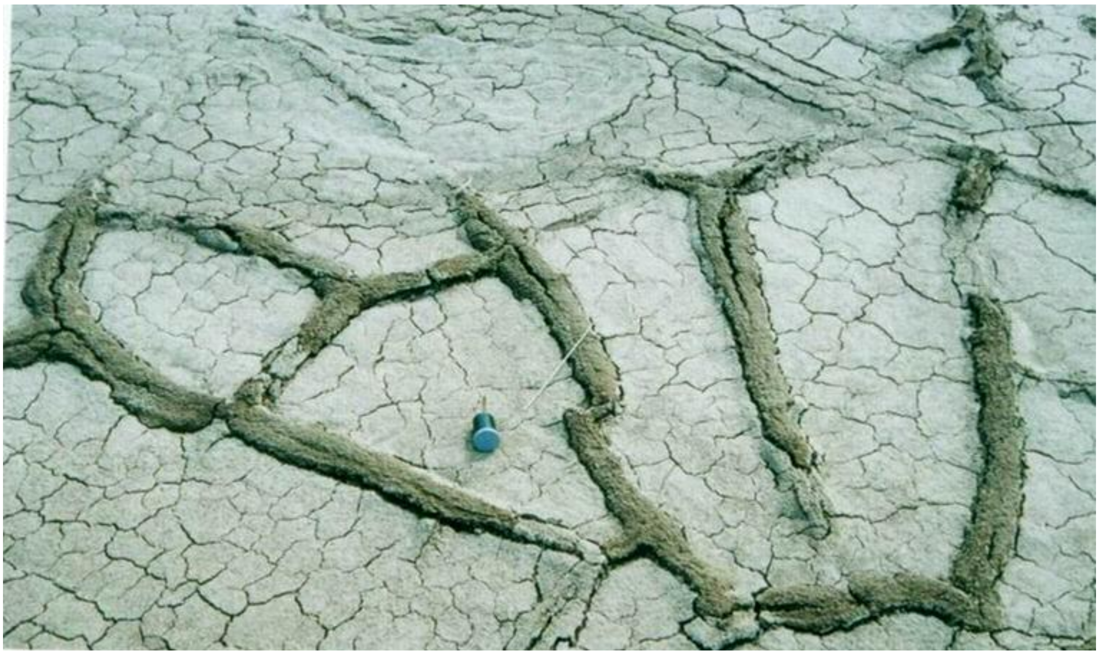

A close-up, nearly top-down view of some frozen sand ridges. They form a vessel net and in the middle there are some clear fissions. In the sand ridge there is a great deal frozen bubbles. The small black box near the lower middle is a film box of about 4.5cm length, and provides a sense of scale.

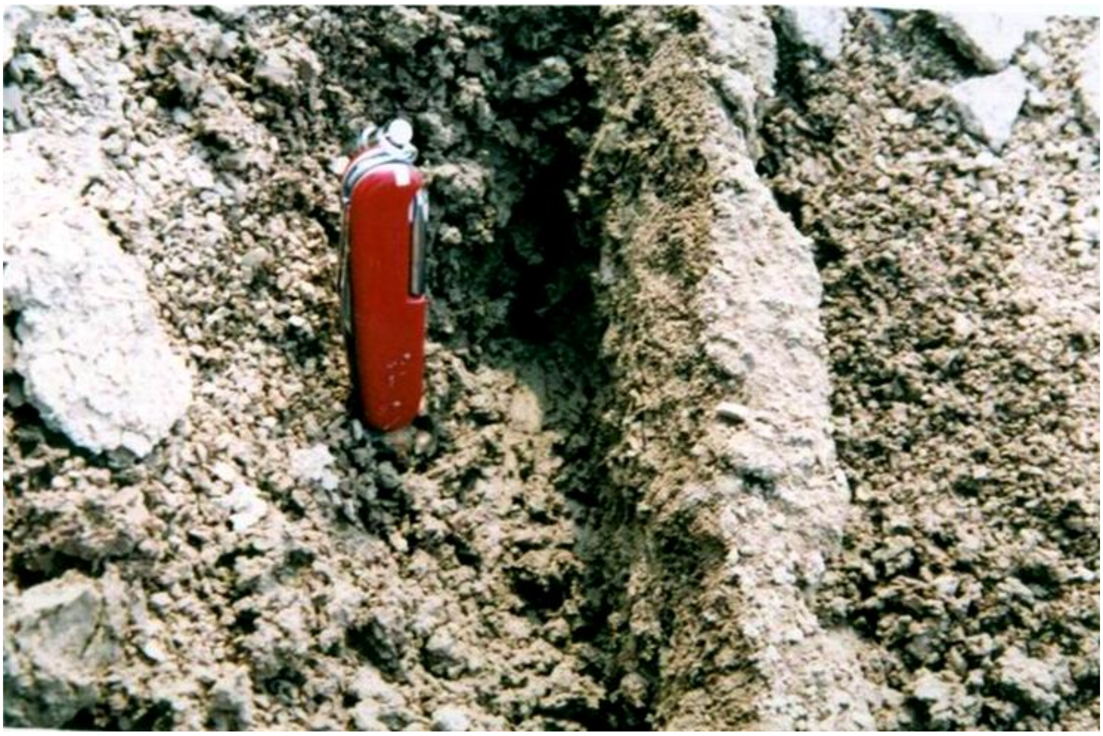

A dug out frozen sand ridge like a upright sand vein in mud. The standing Swiss knife is 6cm long.

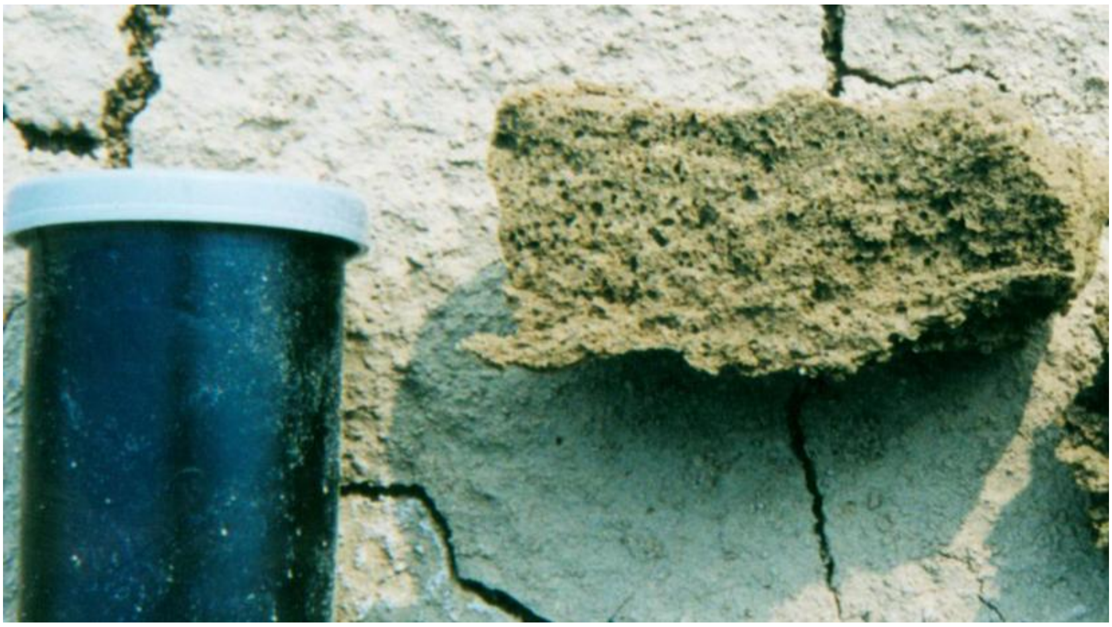

Within the frozen sand ridges there are a great deal of frozen bubbles with spherical shape, from 1-2mm to 4-5mm in diameter, making the sand ridge being expanded and extruded over the deposit surface. The film box is about 4.5cm long.

12. Frozen fissures

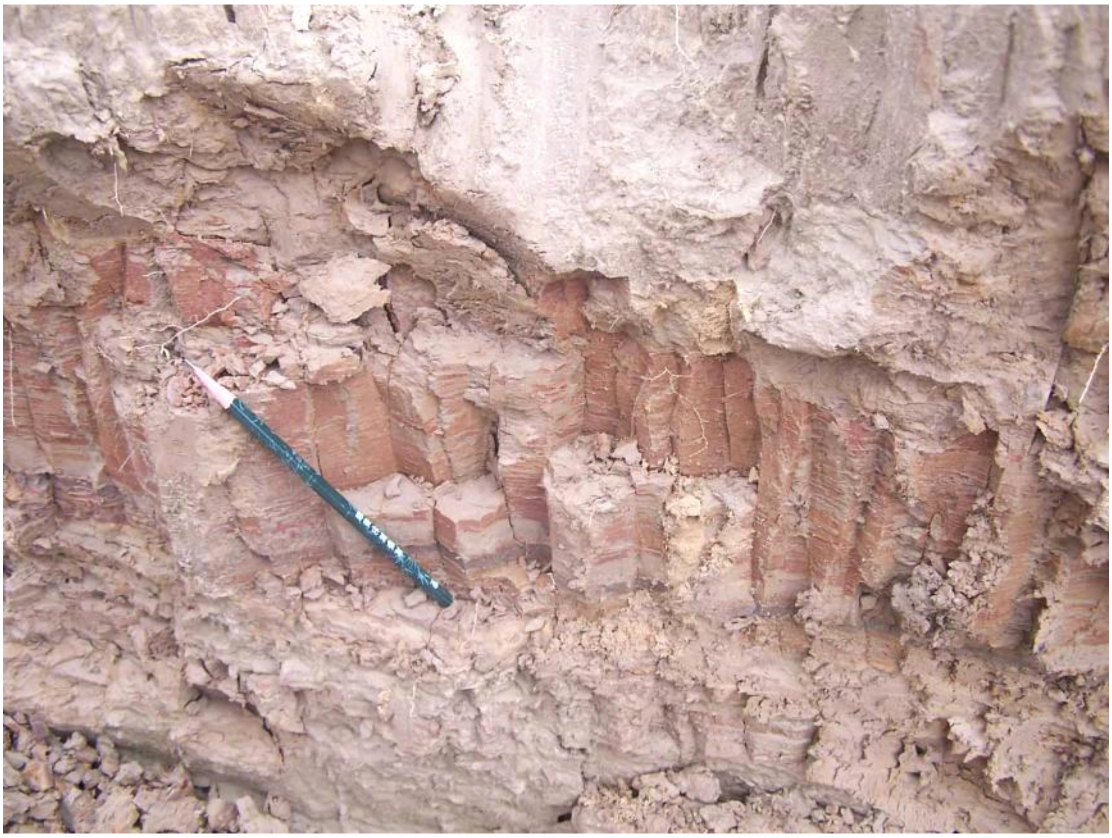

A series of vertical frozen fissures. They developed in wet, reddish-brown mud.

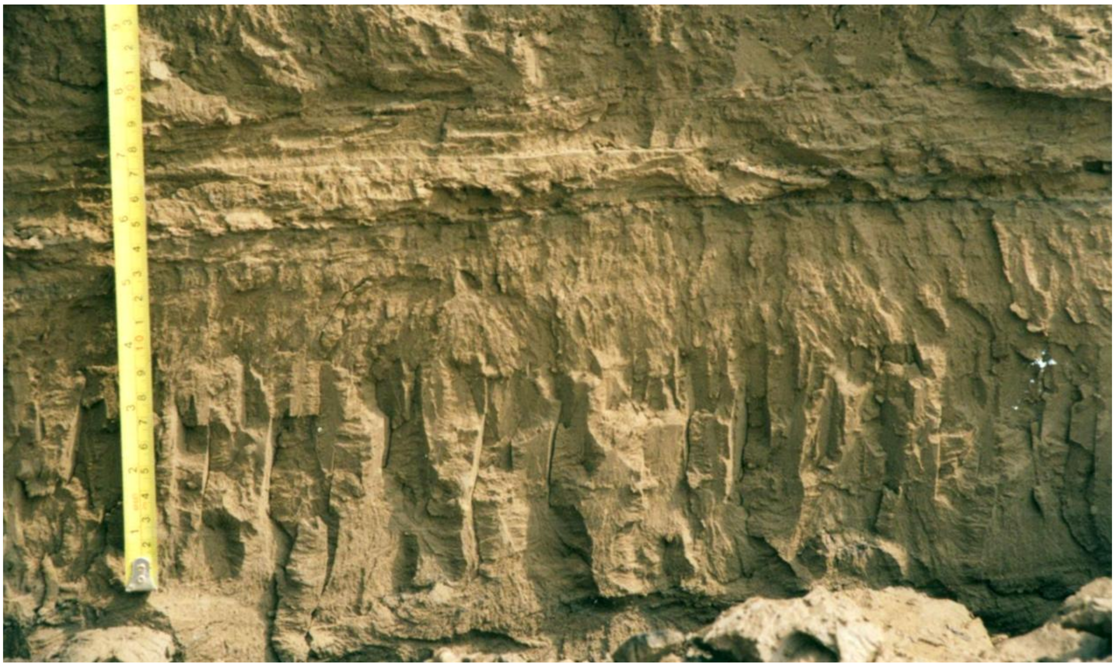

A series of small vertical frozen fissures which developed in wet, earthy-yellow silt layer.

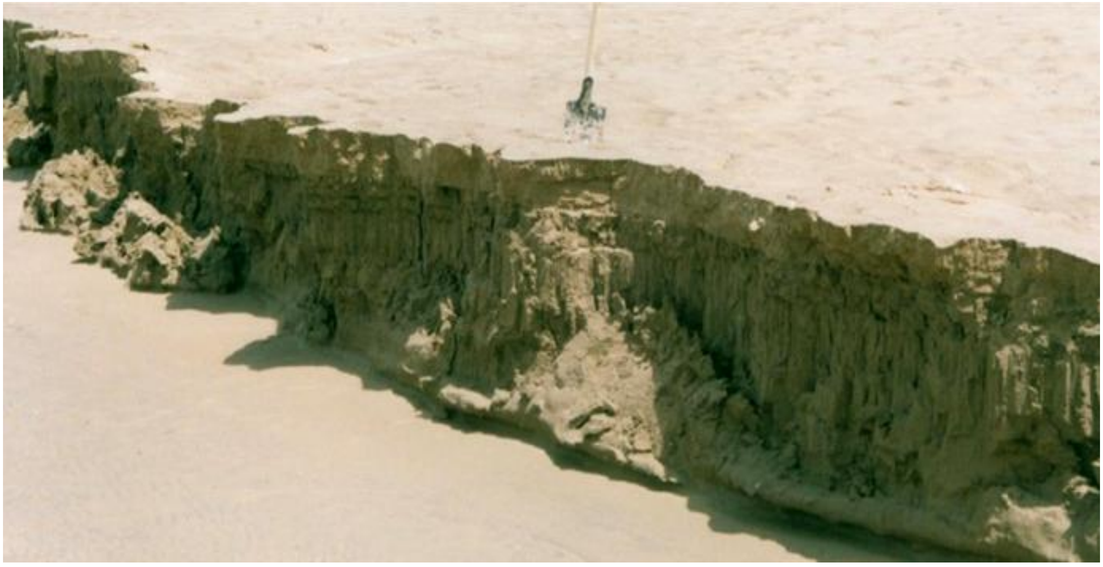

A series of relatively large vertical frozen fissures. They developed in wet, earthy-yellow silt in the vertical channel bar margin which was formed by erosive wave action. The visible part of the standing shovel is about 40cm in length.

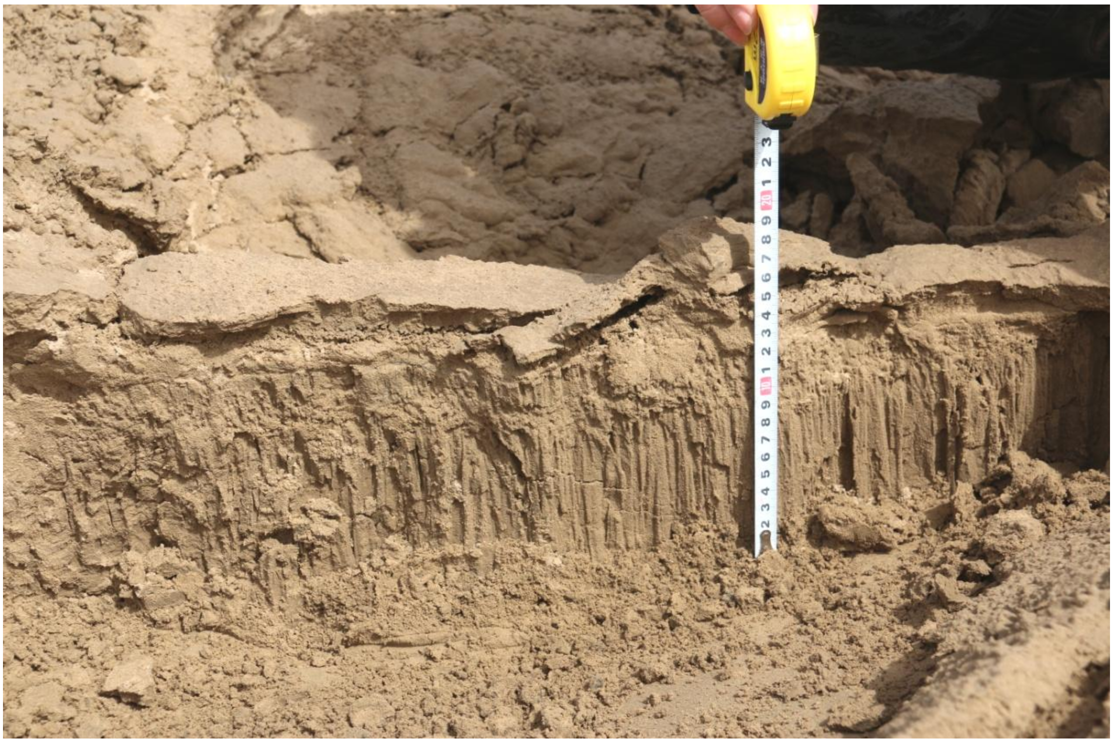

A series of small vertical frozen fissions. They developed in the wet, earthy-yellow silt of the channel bar.

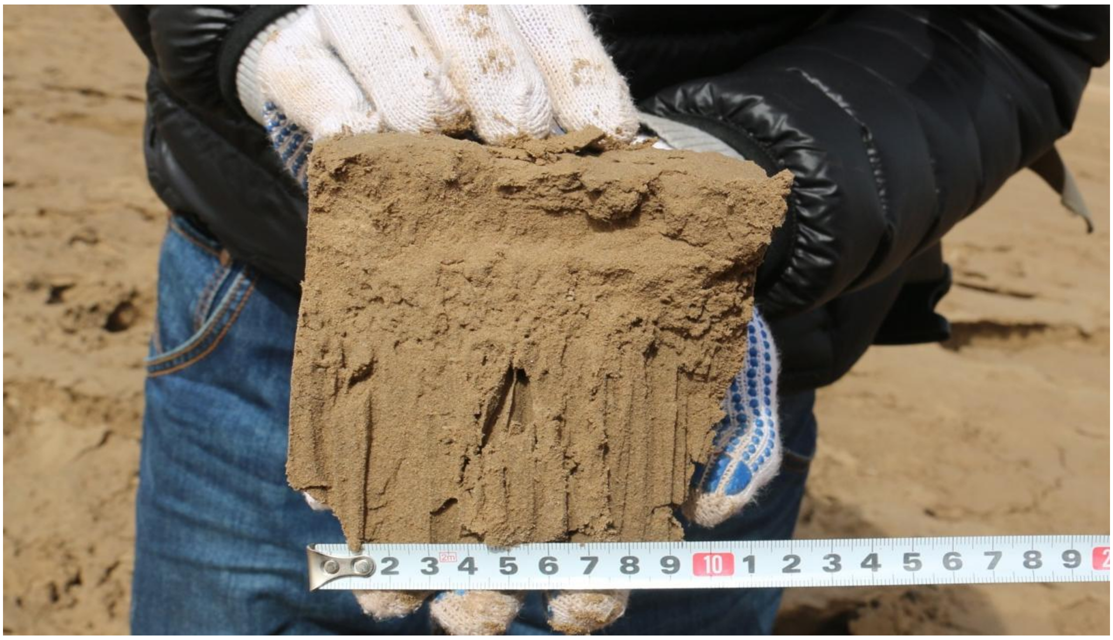

A close-up view of small vertical frozen fissures from the lower section.

13. Ice melt ridges

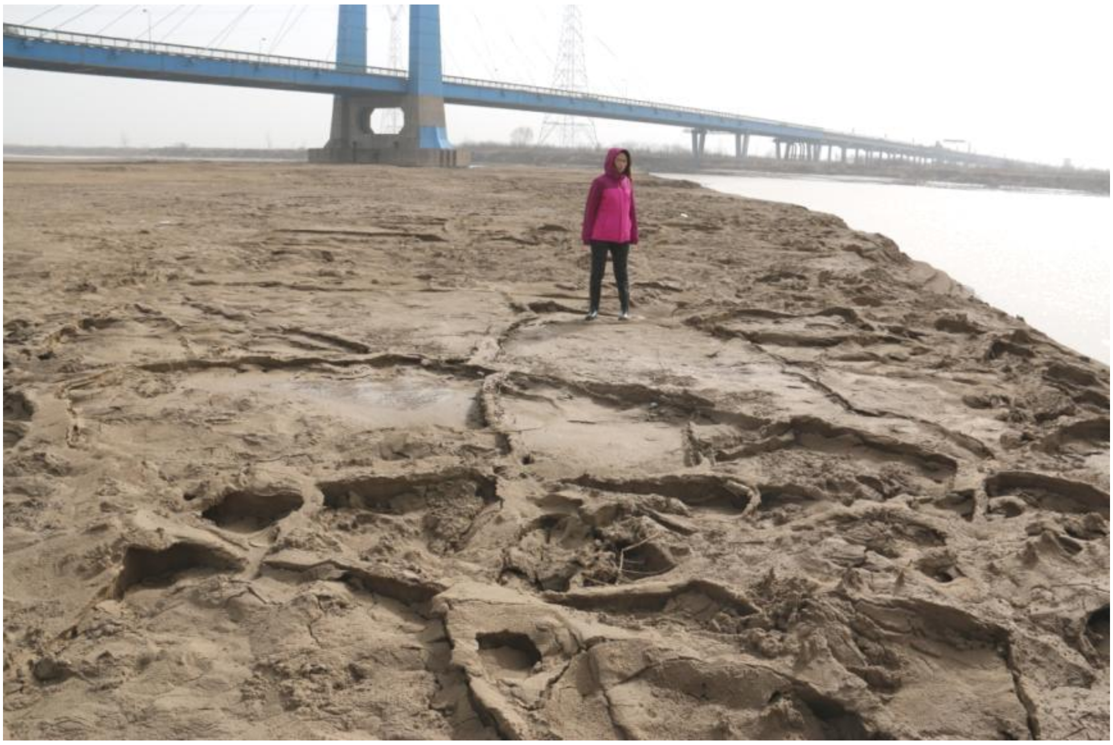

A group of extremely complicated ice melt ridges. They have combined to form a complicated network. The pentagonal depressions between them are formed by the melting of ice blocks. The figure is the seventh author.
The person appearing in the figure is the co- author Cao Mengchun.

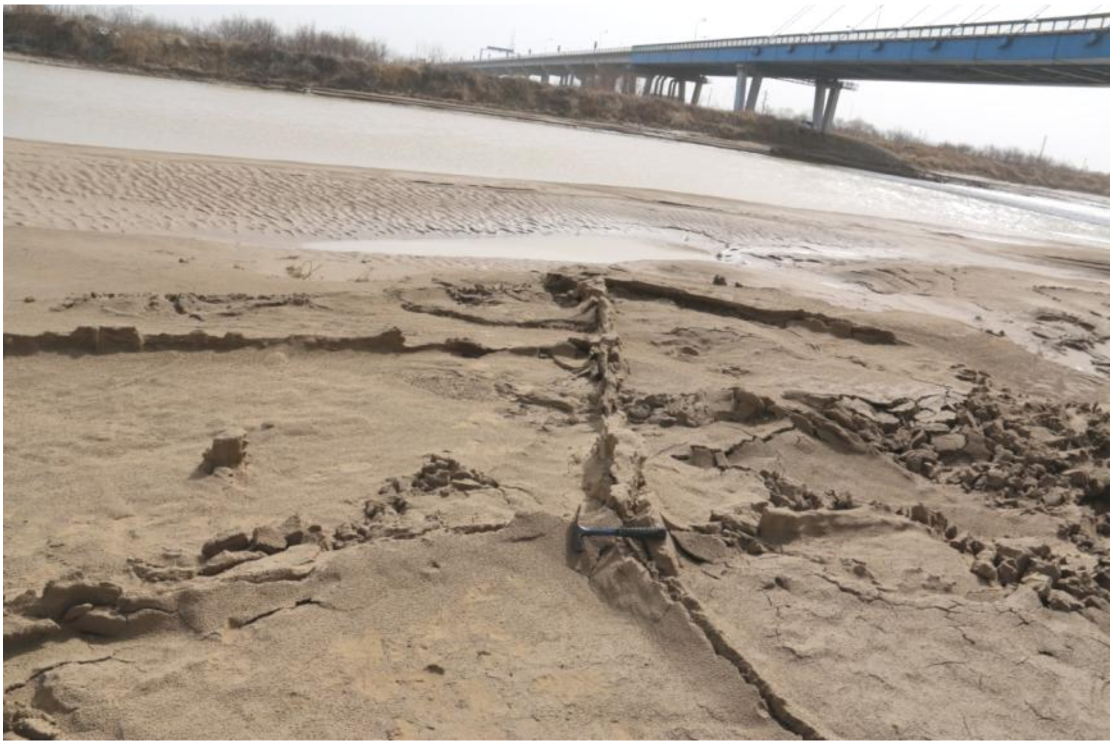

A few of narrow and clear, but extremely irregular ice melt ridges. The hammer is 28cm long.

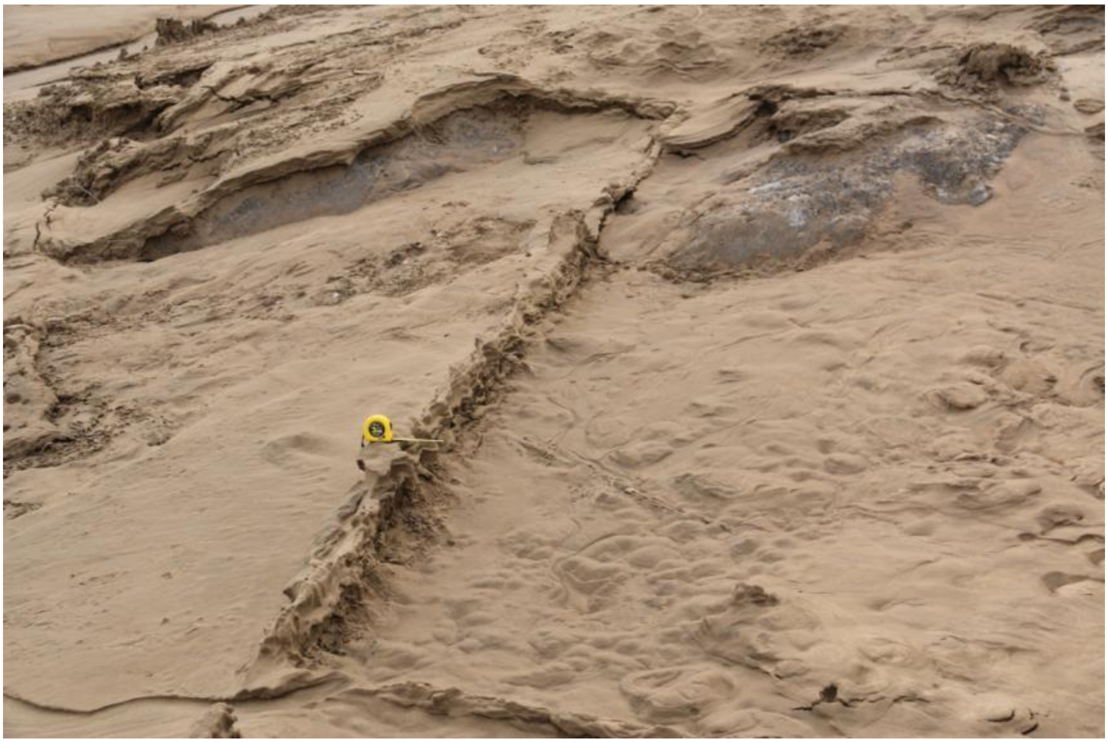

An irregular, upright ice melt ridge. It looks like an extremely irregular, narrow wall and is about 10cm tall and 5cm wide. Its surface is considerably irregular and has a great deal of small holes which were formed by ice water.

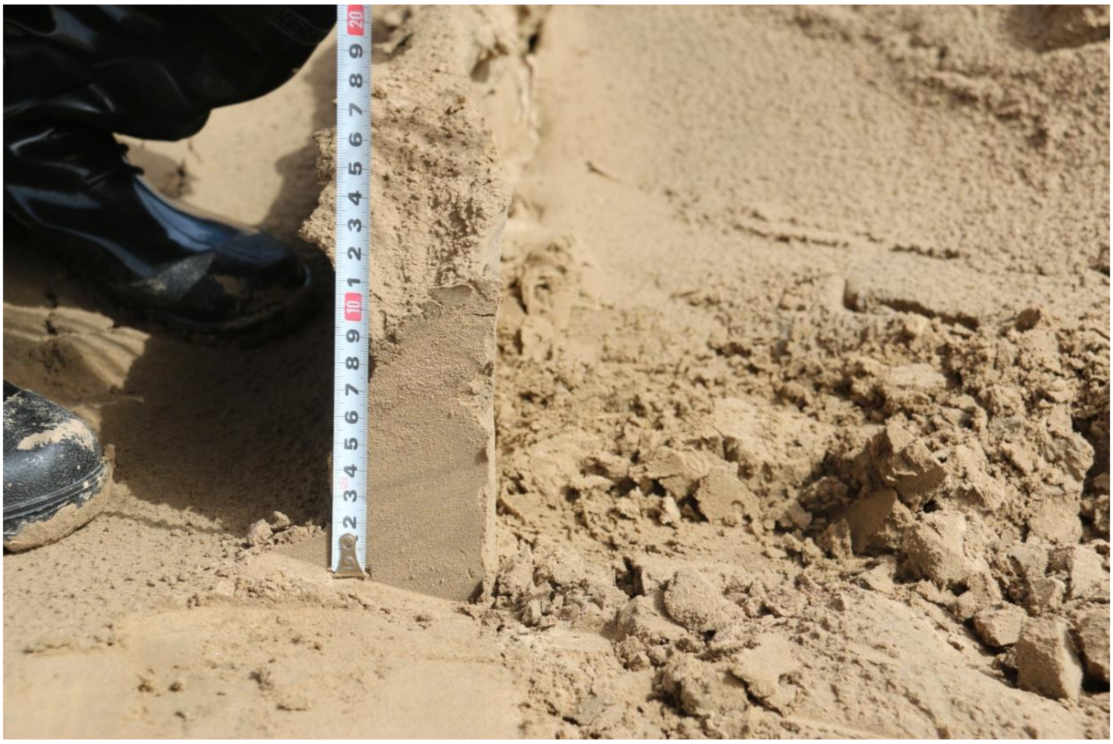

A narrow and upright ice melt ridge. It looks like a narrow wall and is about 10cm tall and 5cm wide. In the lower section, some slightly slanted bedding planes can be seen.

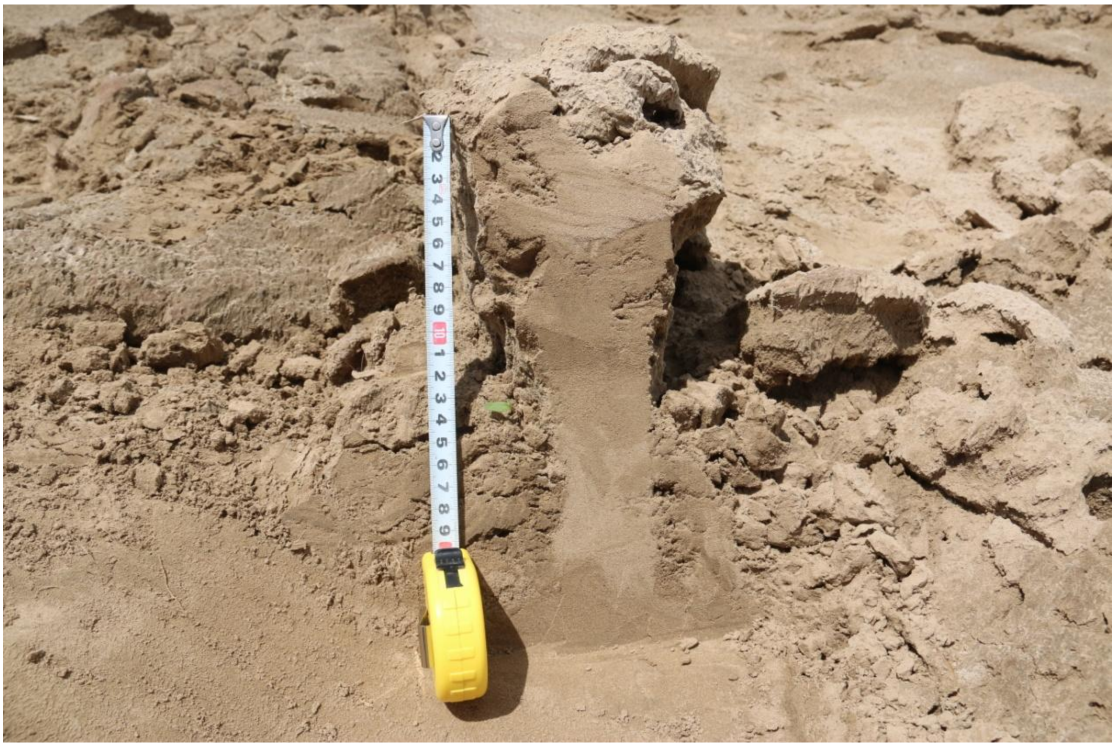

A narrow and upright ice melt ridge with a uneven and inflated top. It looks like a narrow wall and is about 20cm tall and 5-10cm wide. In the middle and lower areas of the upper section there are some slightly slanted bedding planes.

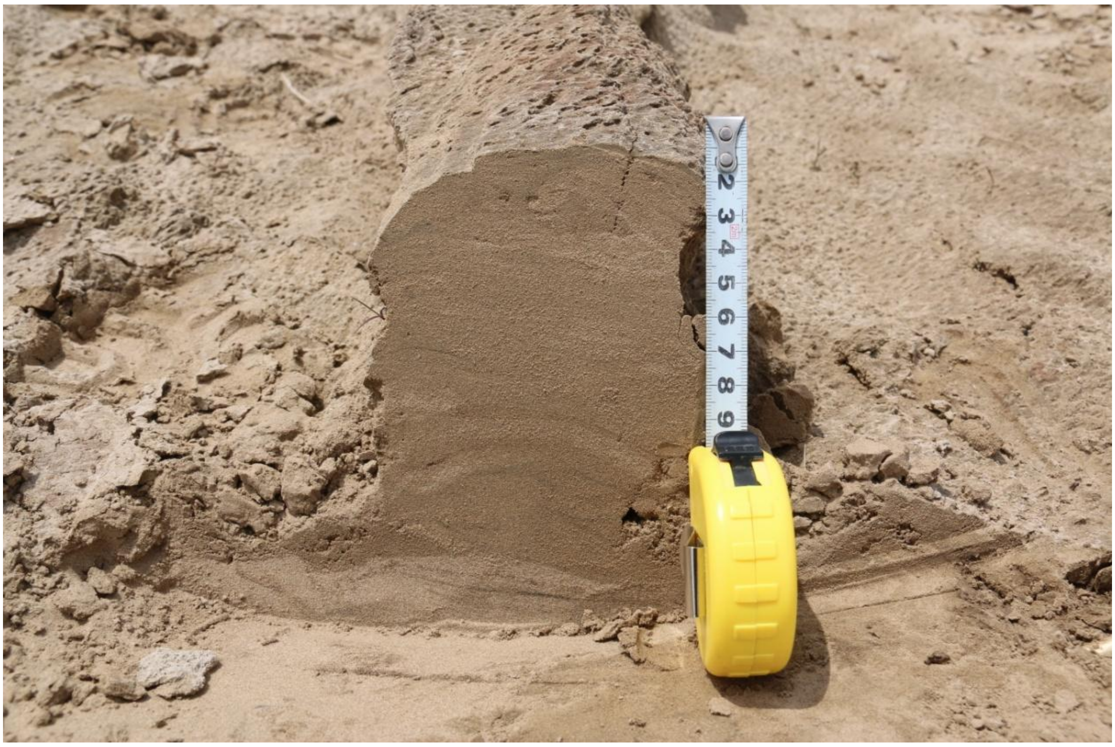

A relatively wide upright ice melt ridge. It looks like a relatively wide wall and is about 10cm tall and 8-9cm wide. There are some slightly convex bedding planes in the lower section.

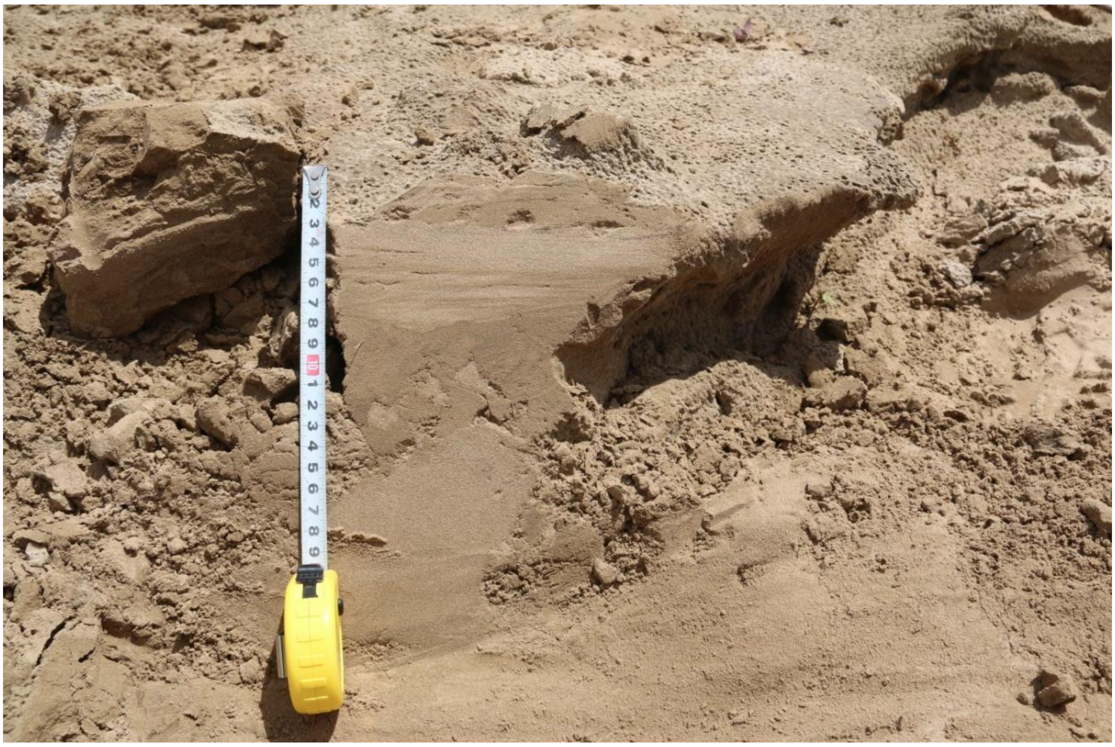

An irregular upright ice melt ridge with an overhanging ledge on upper right side. It is about 20cm in height and 18cm wide at the top, tapering down to just 10cm at the base. In the upper section there are some horizontal bedding planes, and there are some deformed beddings in the lower section.

14. Ice-water-induced root-like structures

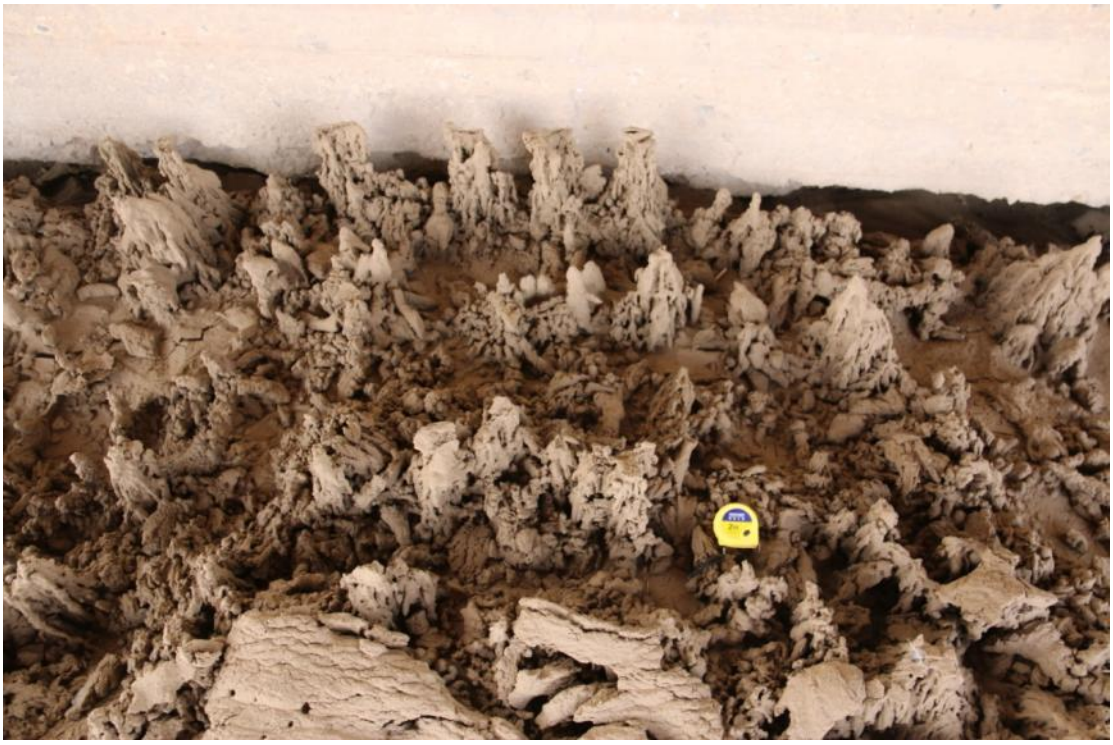

A group of complicated ice-water-induced root-like structures.

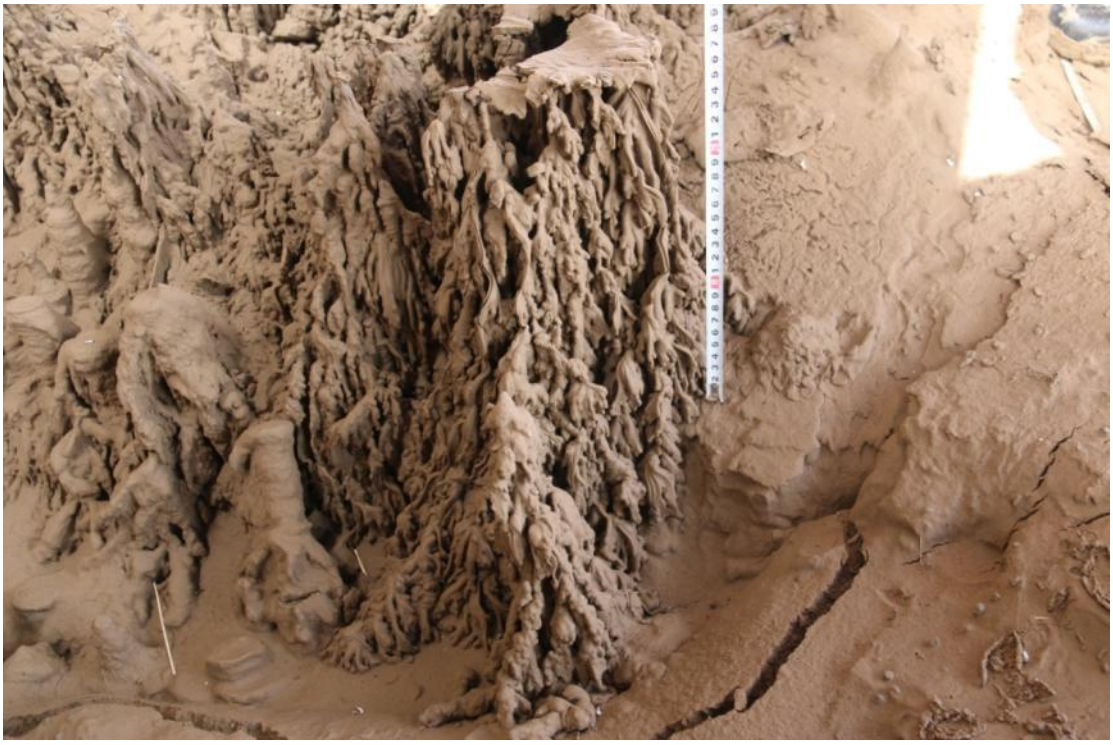

A few clusters of complicated ice-water-induced root-like structures. The structures are comprised of a series of fine or noodle-like shapes, giving the appearance of an irregular network of roots.

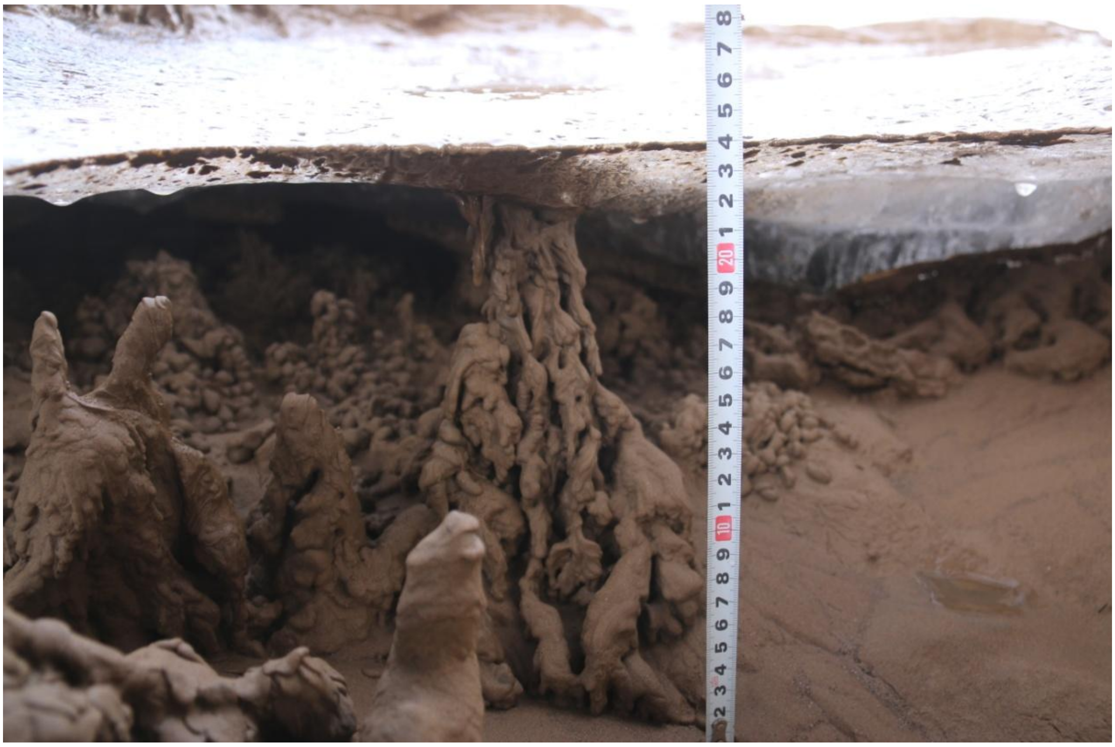

A cluster of ice-water-induced root-like structures still in the forming process. The greyish-white structure which can be seen at the top of the photo is a melting ice layer.

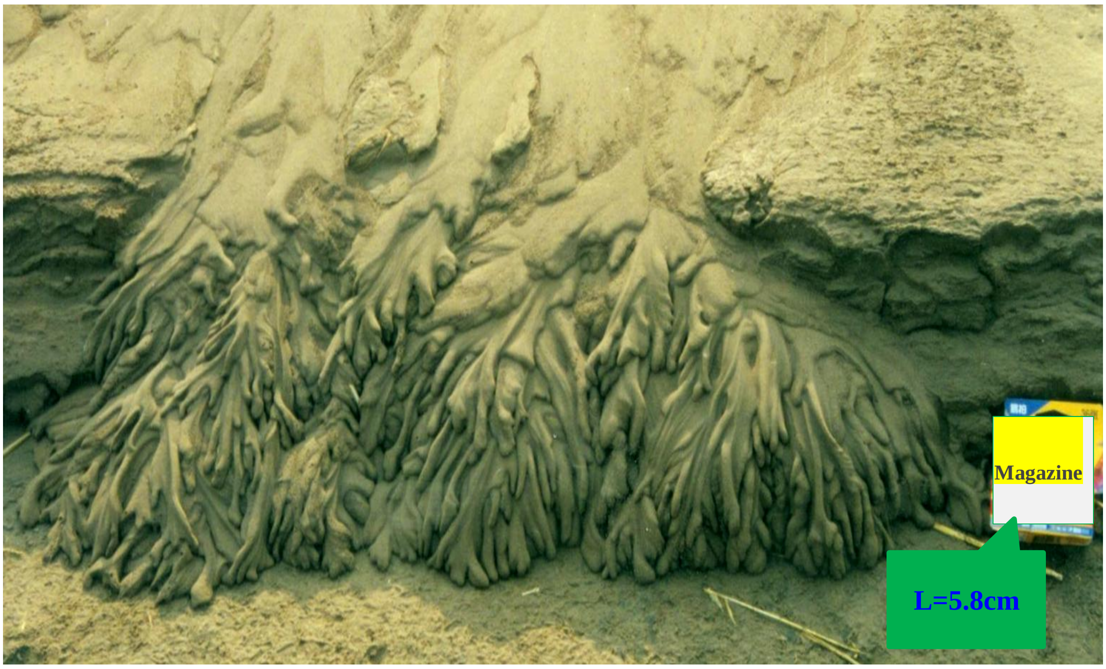

A cluster of well-developed ice-water-induced root-like structures. They developed in the lower upright part of the channel bar margin.

15. Ice-water-induced pillar-like structures

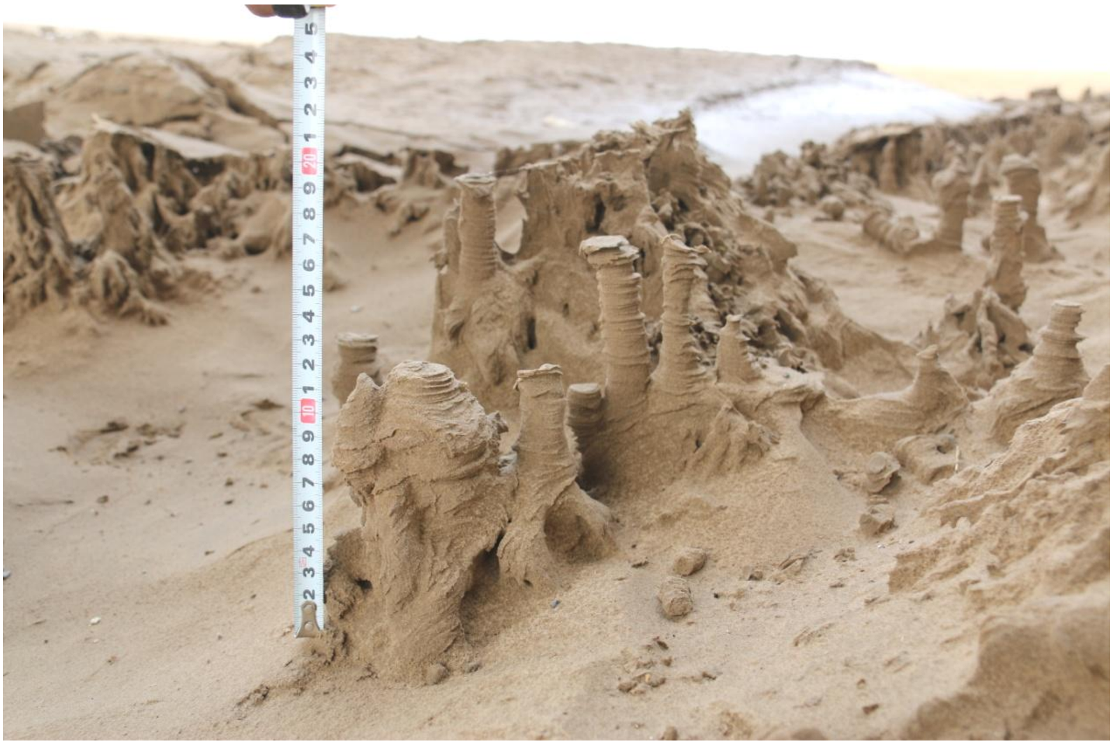

A group of Ice-water-induced pillars. They have laminar structures, which is unusual, they have horizontal bedding planes and are formed by deposition, forming layers one by one from the top to the bottom, and were caused by the melting of the upper silt-bearing ice layer.

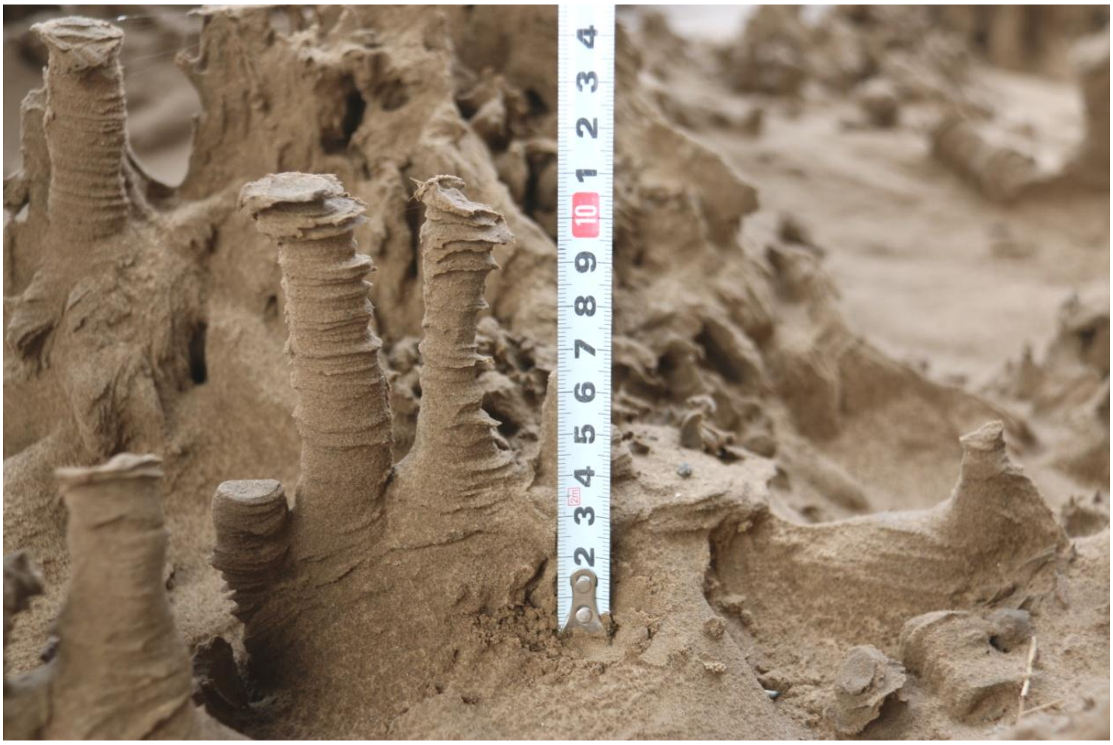

A close-up view of a few ice-water-induced pillar-like structures with laminar structures, which are unusual, and have horizontal bedding planes.

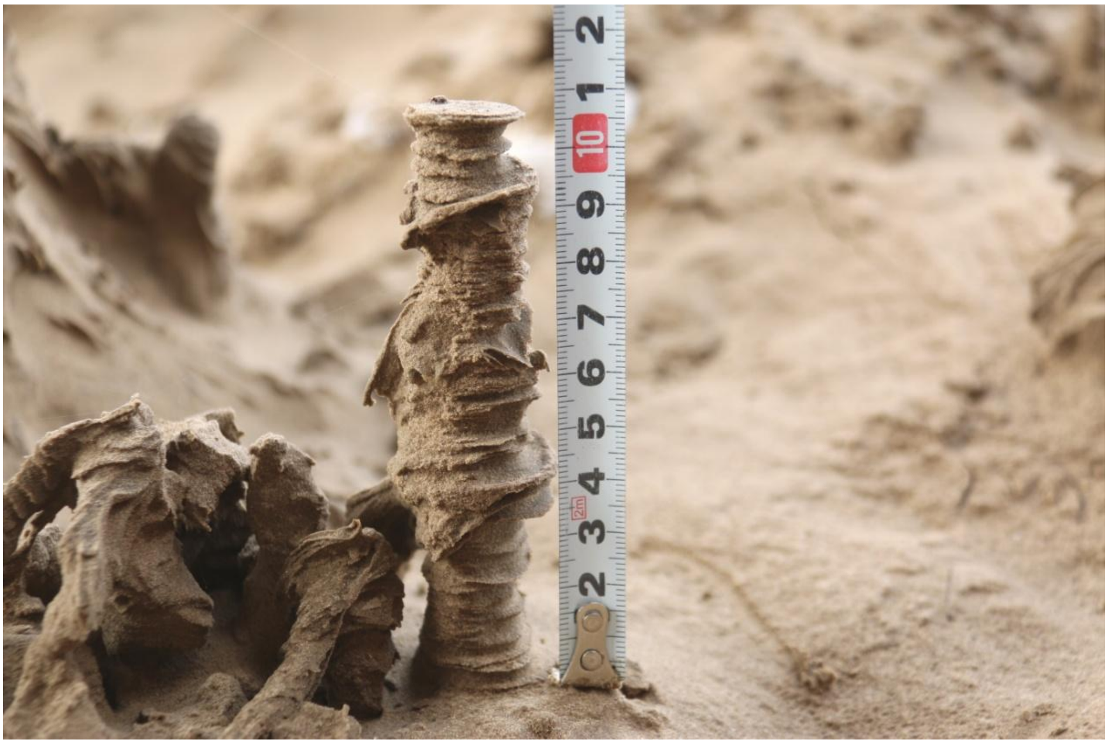

A single ice-water-induced pillar-like with a laminar structure with horizontal or slanted layers. It stands extremely vertically.

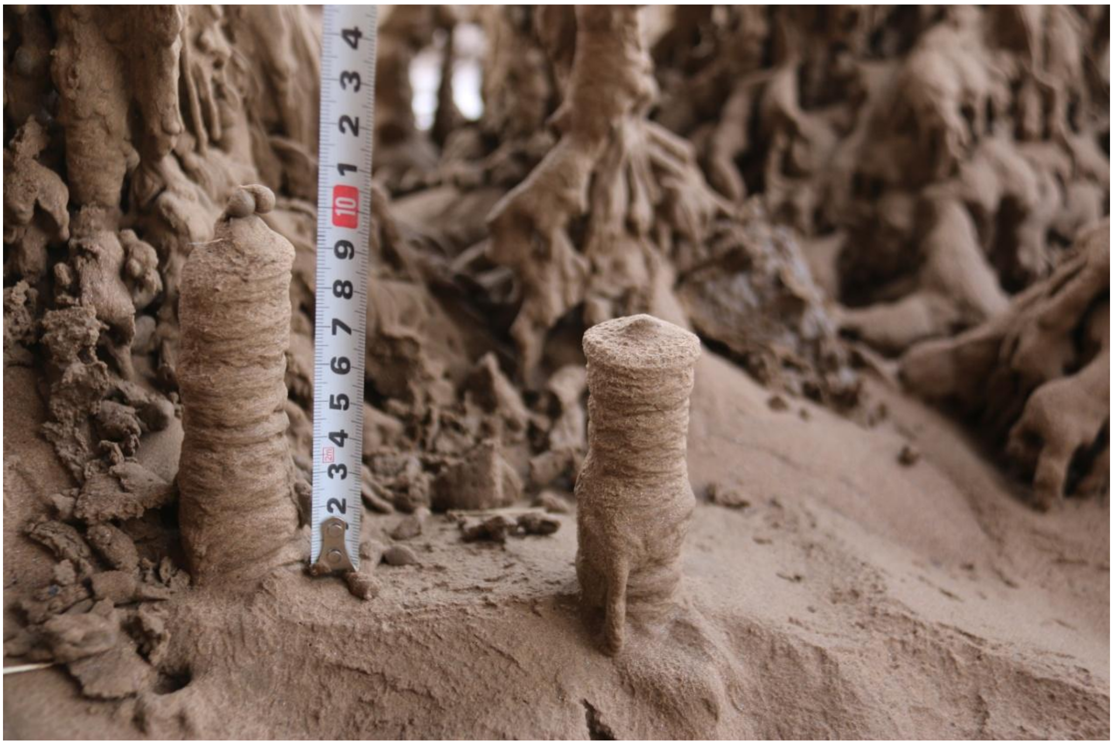

Two typical ice-water-induced pillars. Atop them are nipple-like bulges, which are formed by drips of meltwater bearing a great deal of silt and mud.

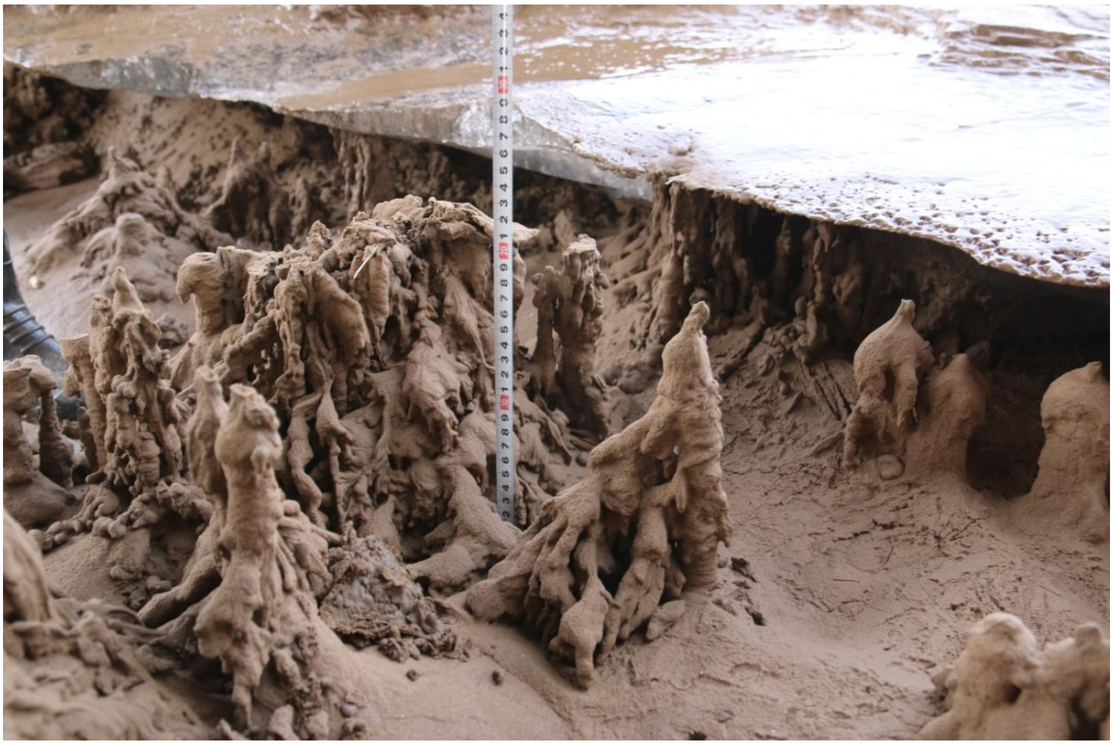

A group of irregular ice-water-induced pillars still in the formation process. The thin transparent ice layer can be seen in the upper section of the photograph. It is melting and carries a small amount of silt and mud. Some of the silt and mud is dripping onto the ice-water-induced pillars.

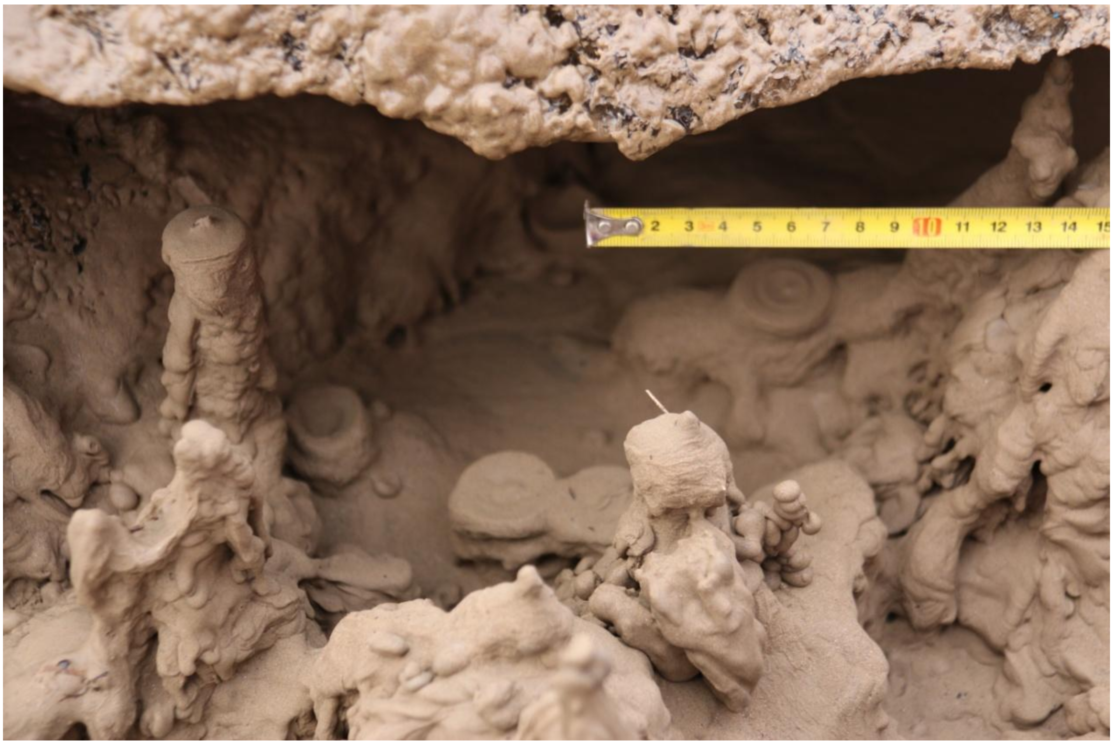

Several prototypical ice-water-induced pillars still in the formation process. It can be observed that the overhanging ice layer bears a great deal of silt and mud, and is melting causing the silt and mud to drop onto the prototypical ice-water-induced pillars.

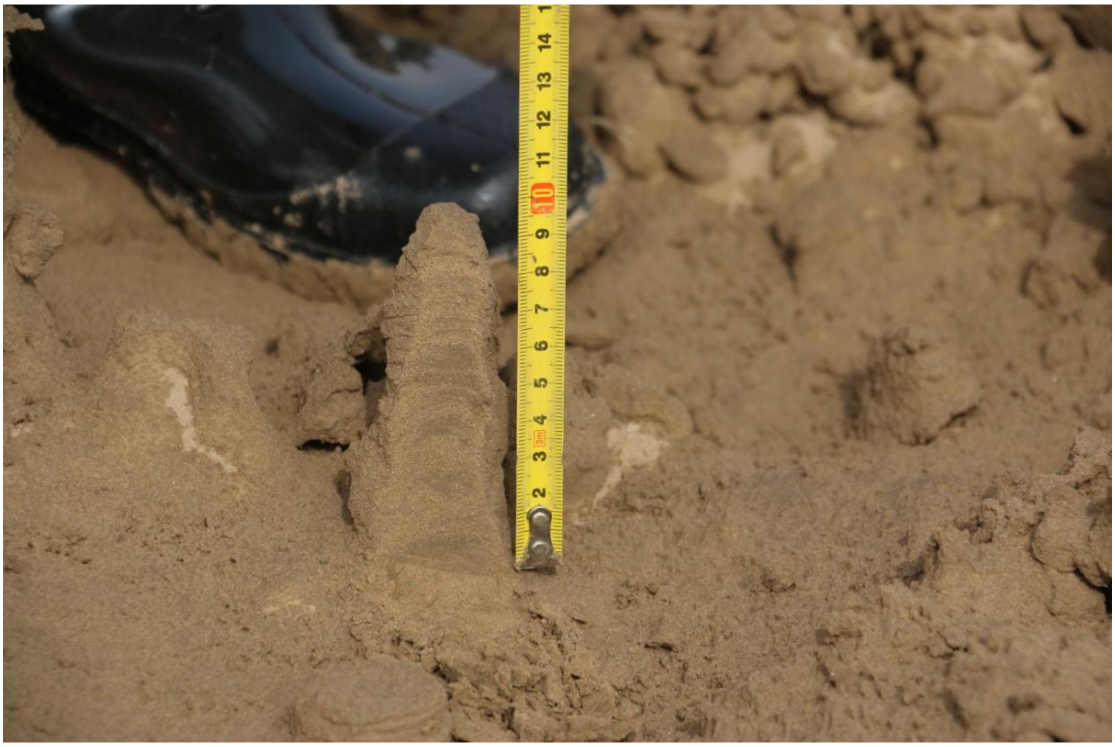

A close-up view of a relatively regular ice-water-induced pillar with some convex beddings.

16. Ice water pea-like structures

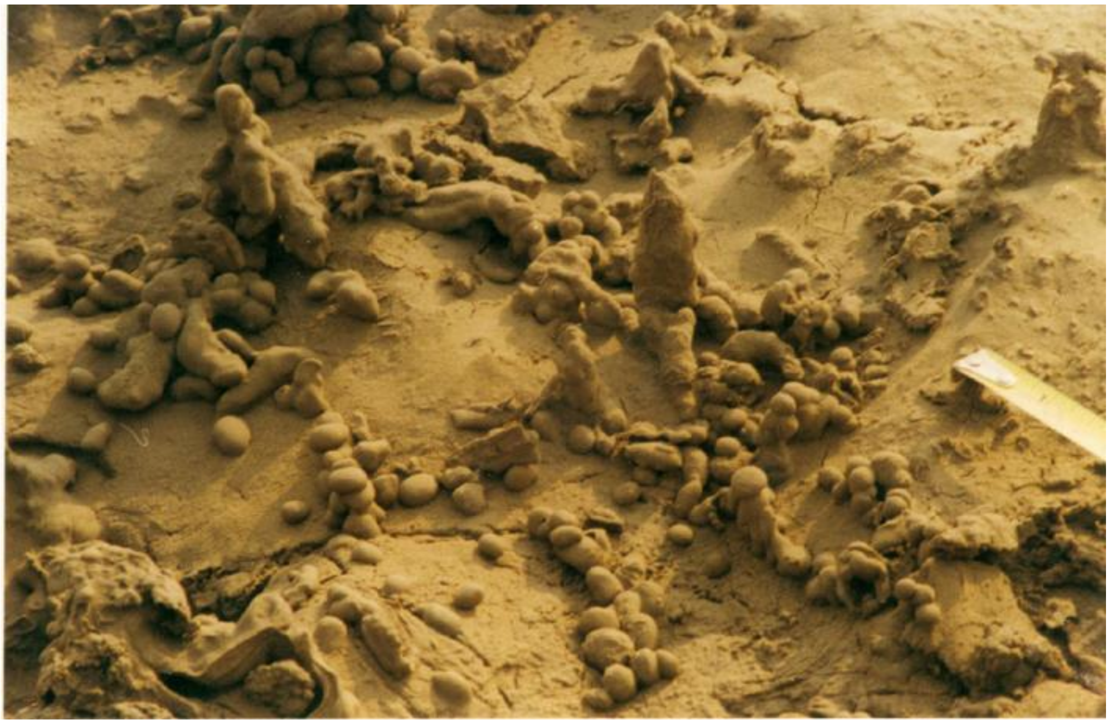

A group of ice water bean-like structures. They share a bean-like shape, with smooth surfaces and measure approximately 10mm in diameter, and are found commonly in groups.

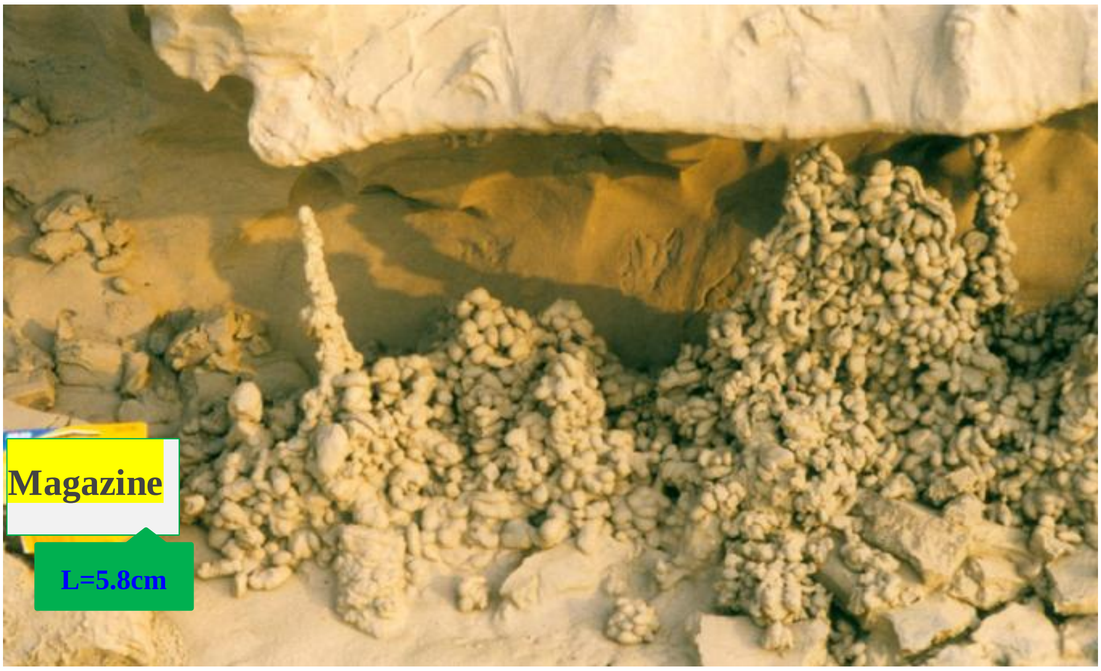

A pile of ice water bean-like structures. They are formed from droplets of meltwater falling from the silt- and mud-saturated ice above.

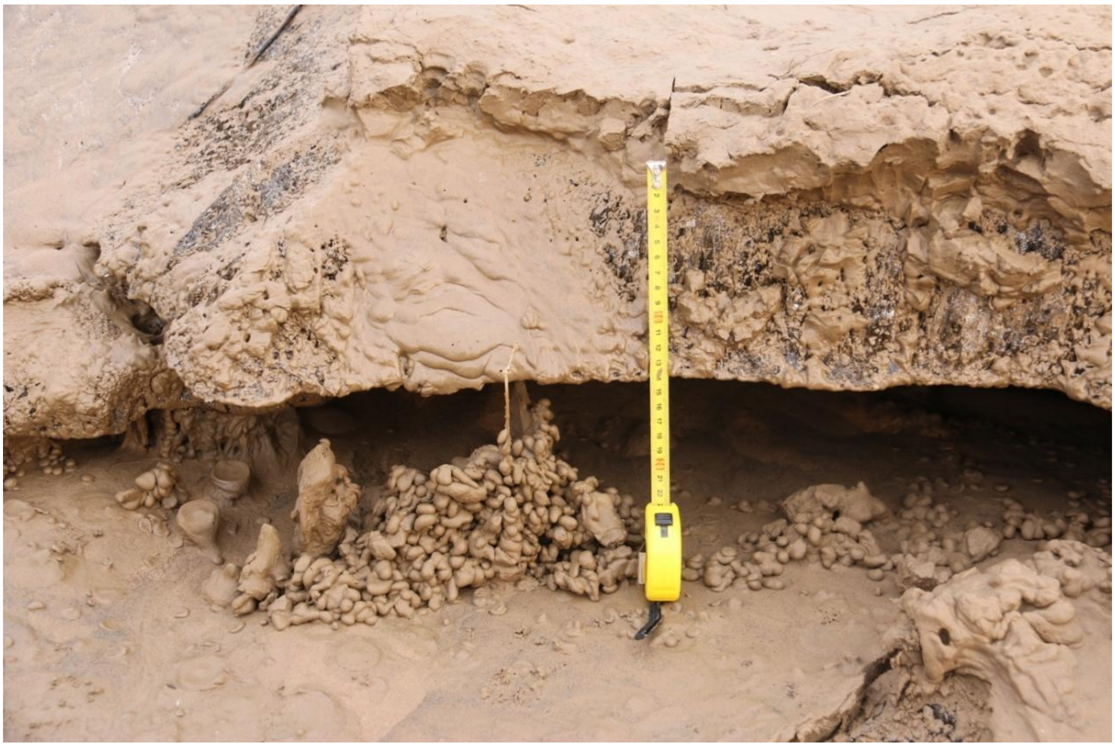

A pile of bean-like structures still in the formation process. They are located just beneath an overhanging ice block, where muddy silt flows from the edge of the ice block, resulting in the formation of the bean-like structures.

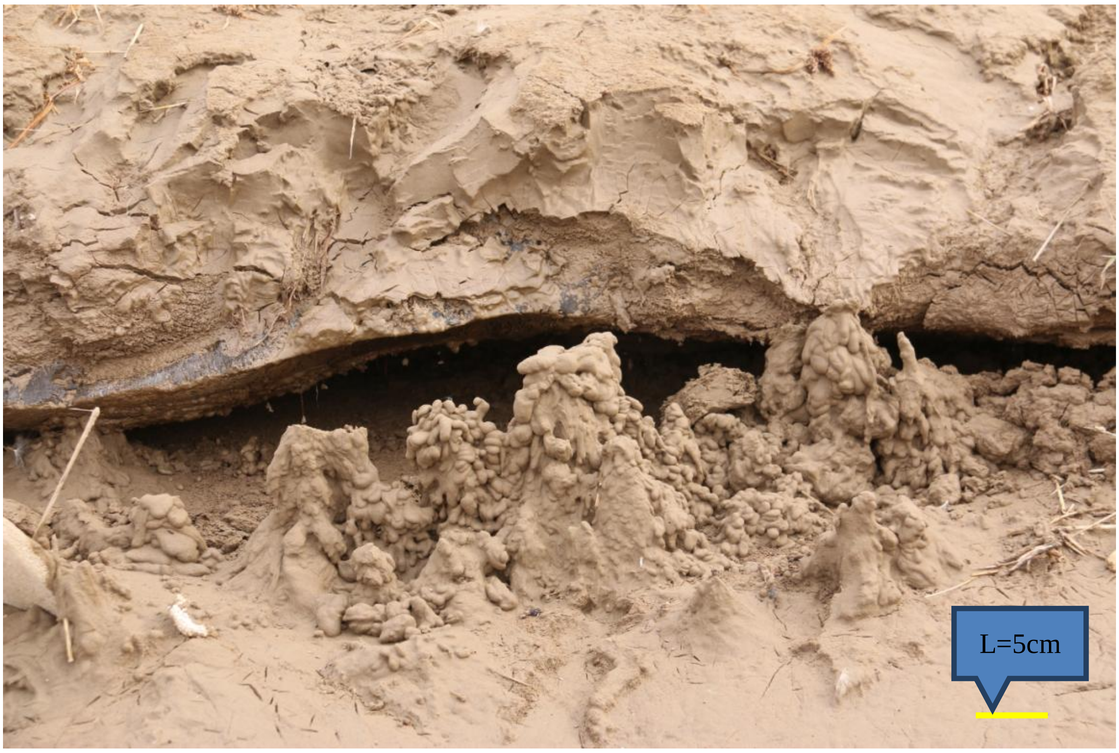

A few piles of bean-like structures still in the formation process. They are located just beneath an overhanging ice block, where muddy silt flows from the edge of the ice block, resulting in the formation of the bean-like structures.

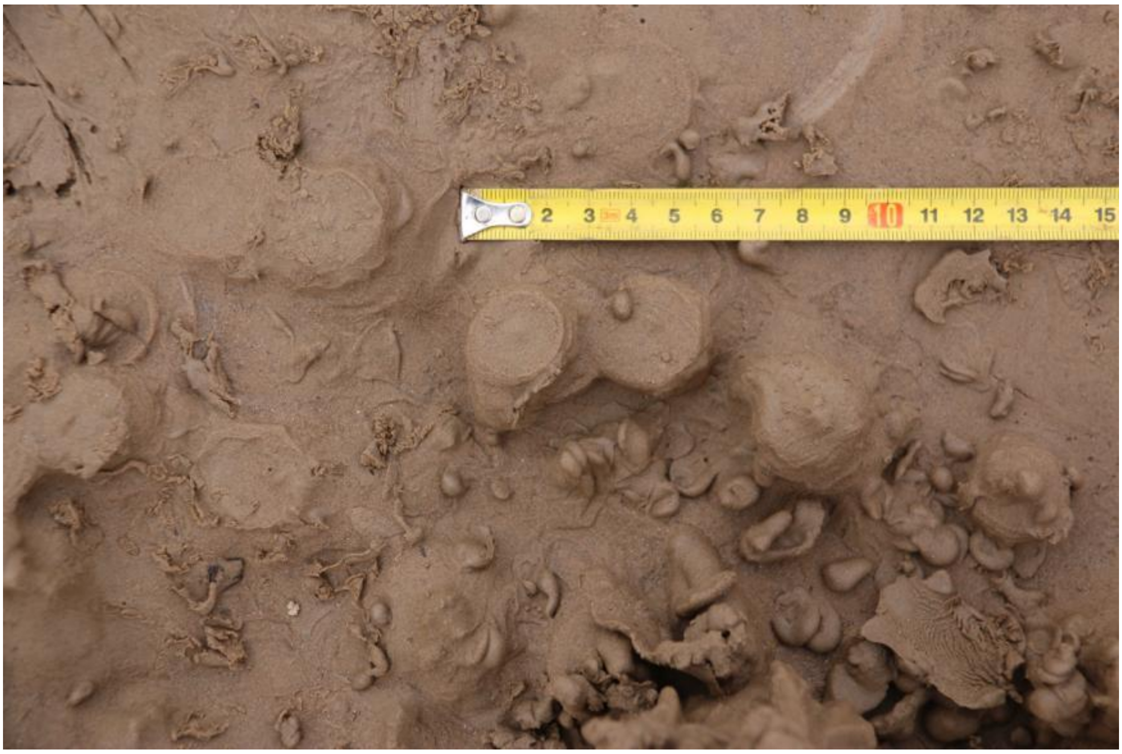

A group of bean-like structures

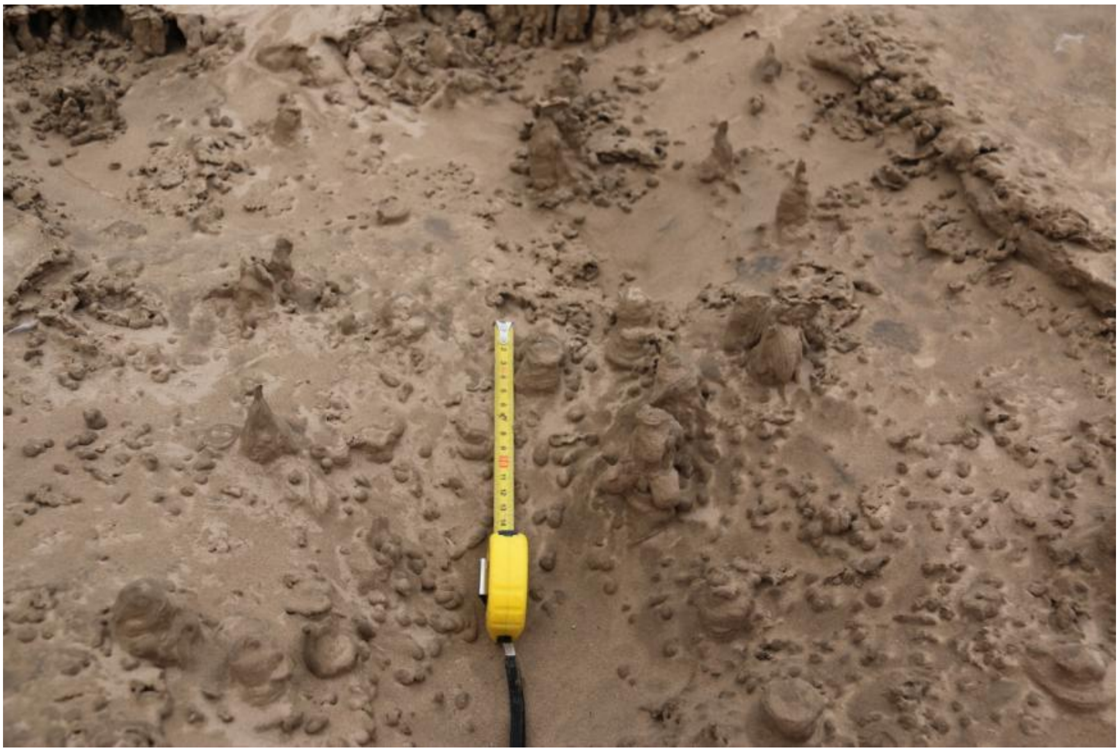

A group of bean-like structures that disperse on the ground.

17. Ice water pseudo-trace structures

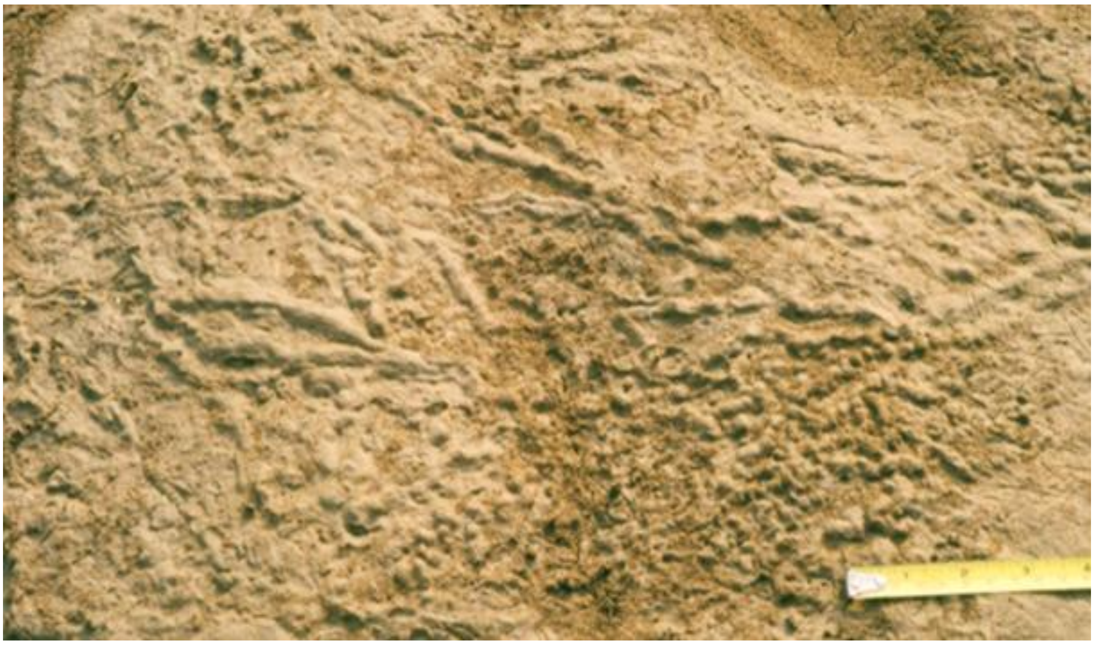

A group of Ice water pseudo-trace structures, so-called because they look so much like trace structures.

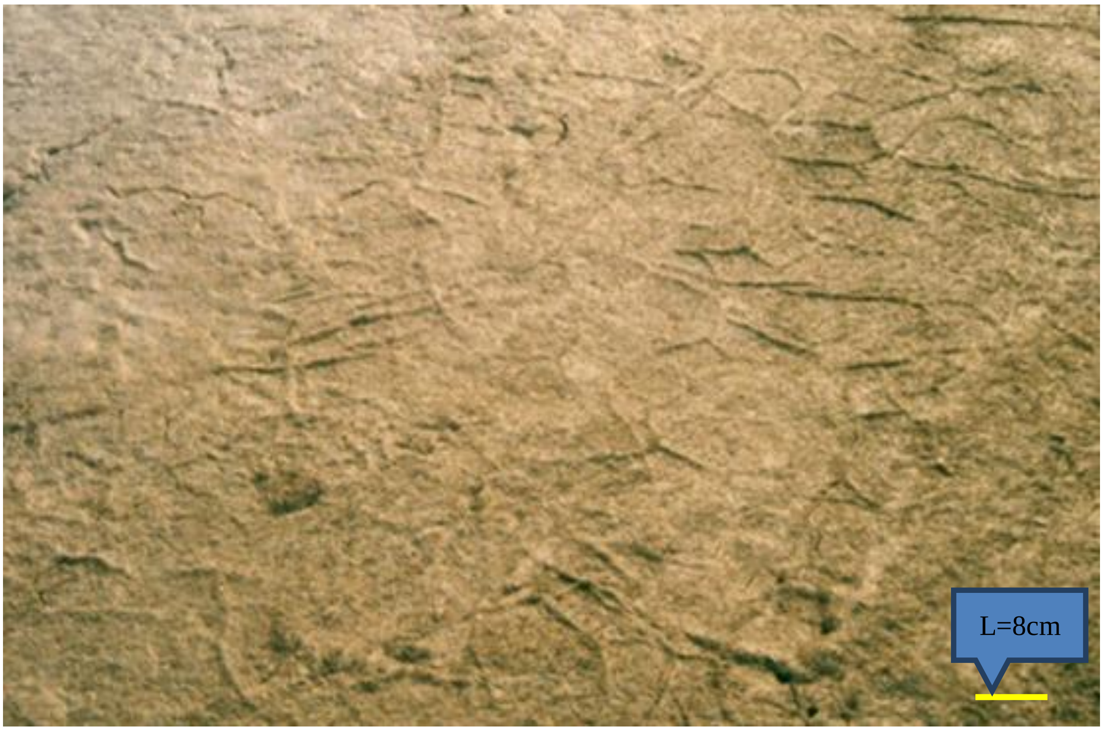

A group of Ice water pseudo-trace structures, they look somewhat like trace structures.

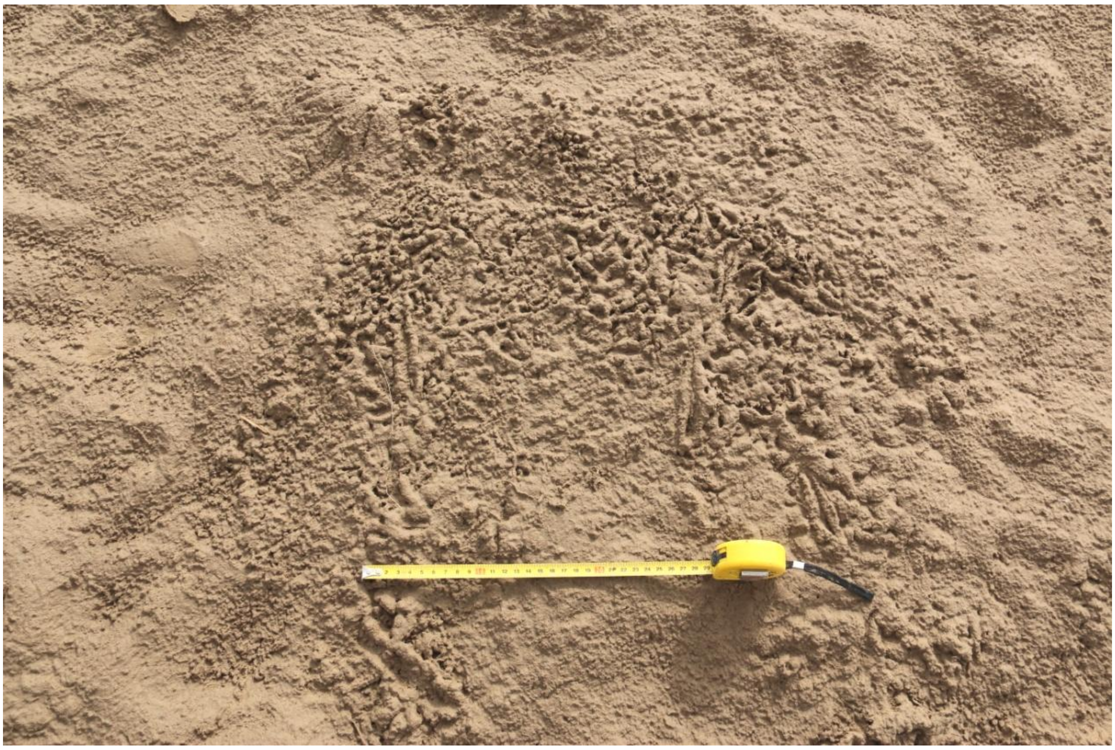

A group of Ice water pseudo-trace structures, which look somewhat similar to trace structures.

18. Frozen ridges

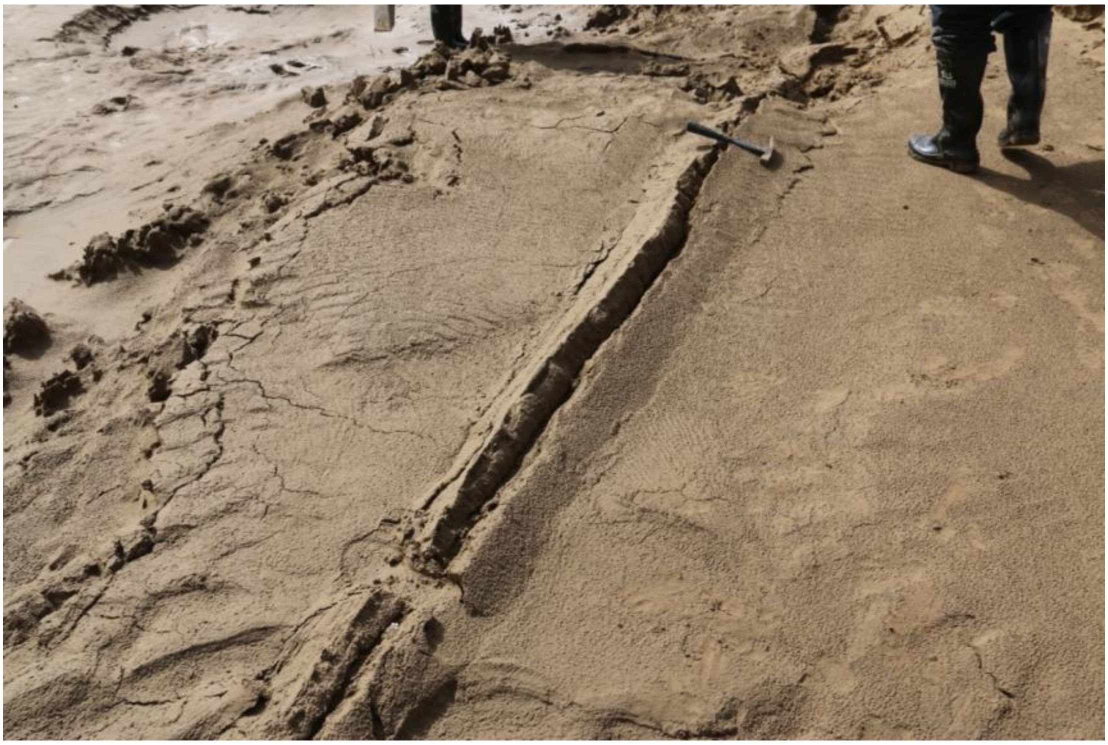

A frozen ridge. It is 555cm long, 32-45cm wide and 4-6cm high. Its most striking feature is the "V"-shaped crack running along its middle. The hammer is 28cm long.

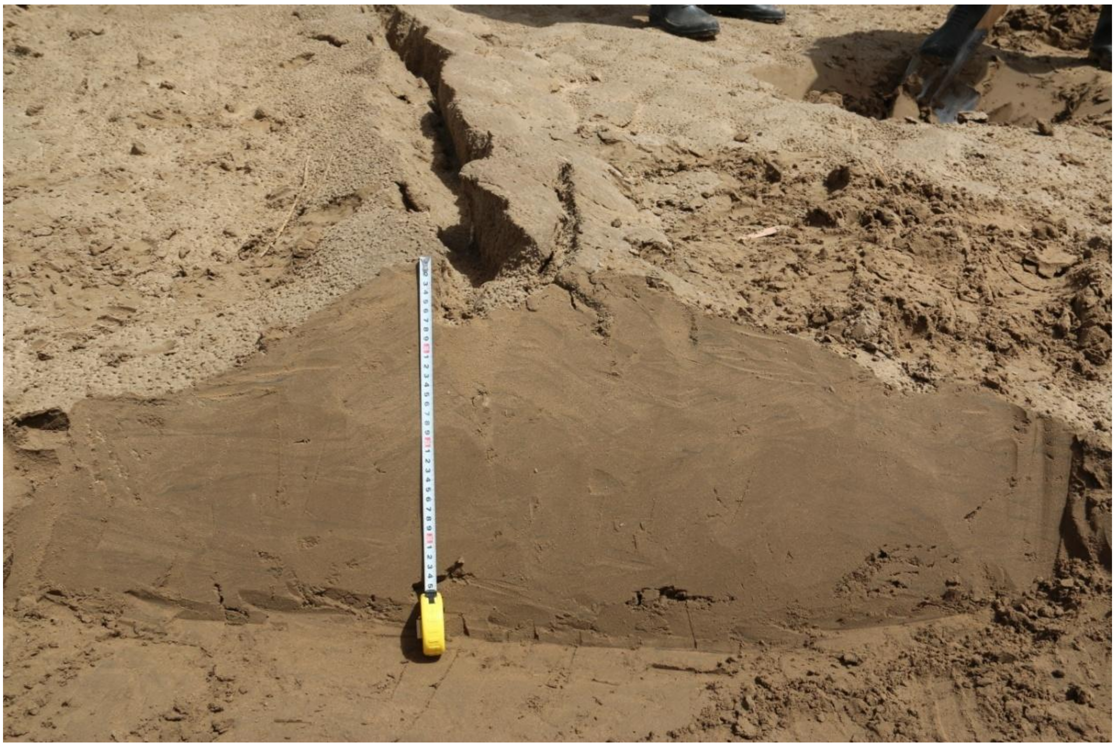

A close-up view of the vertical section of the aforementioned frozen ridge. The lower part of the "V"-shaped crack has been filled with aeolian silt. Its walls are gently sloping. Vertical view of same specimen as above.

19. Ice line mark

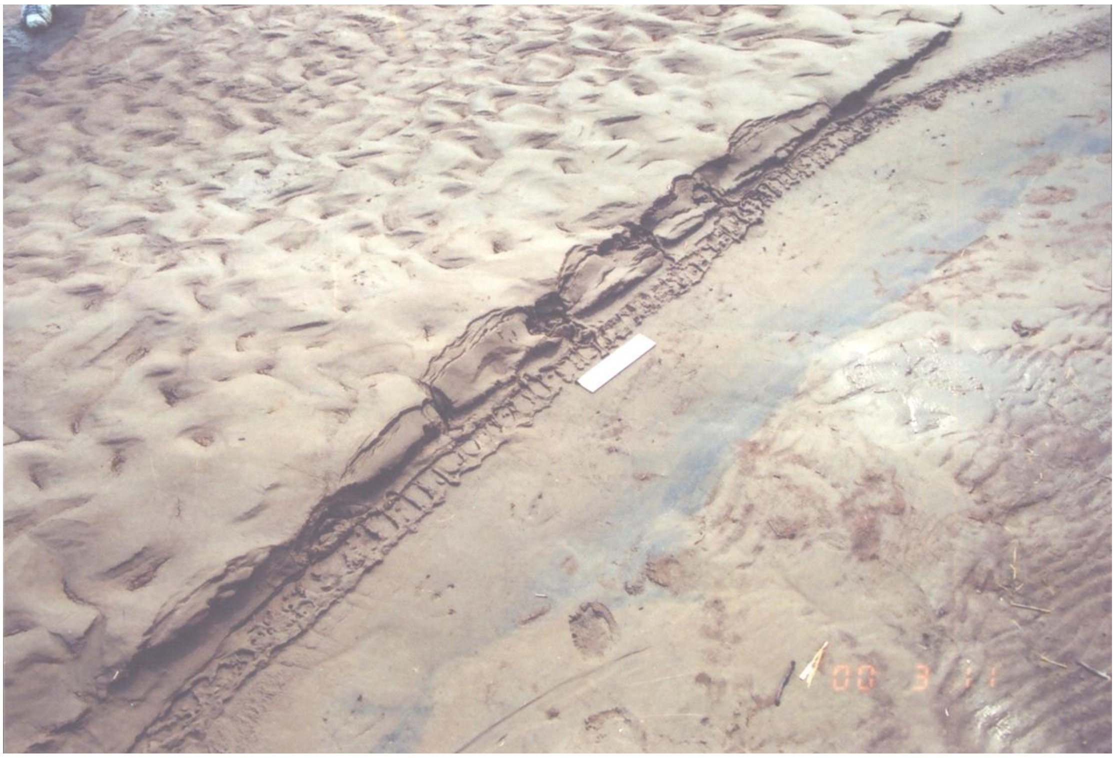

An ice line mark with a unusual, long and narrow belt occurrence, which has a lot of meshes

formed by ice melt deposition, developed near the out margin of a point bar. The length of the ruler is about 24cm.

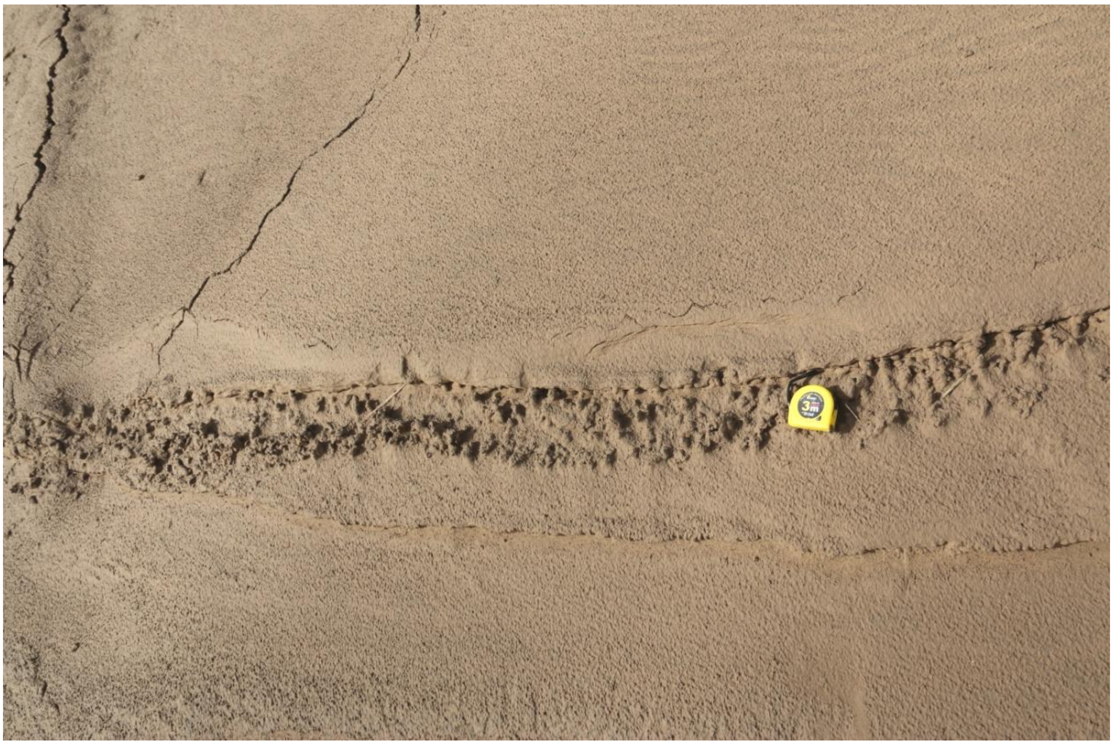

An ice line mark with a unusual, long and narrow belt occurrence, which consists of a lot of bean-like structures formed by ice melt deposition, also developed near the out margin of a point bar.

20. Ice water silt volcanoes

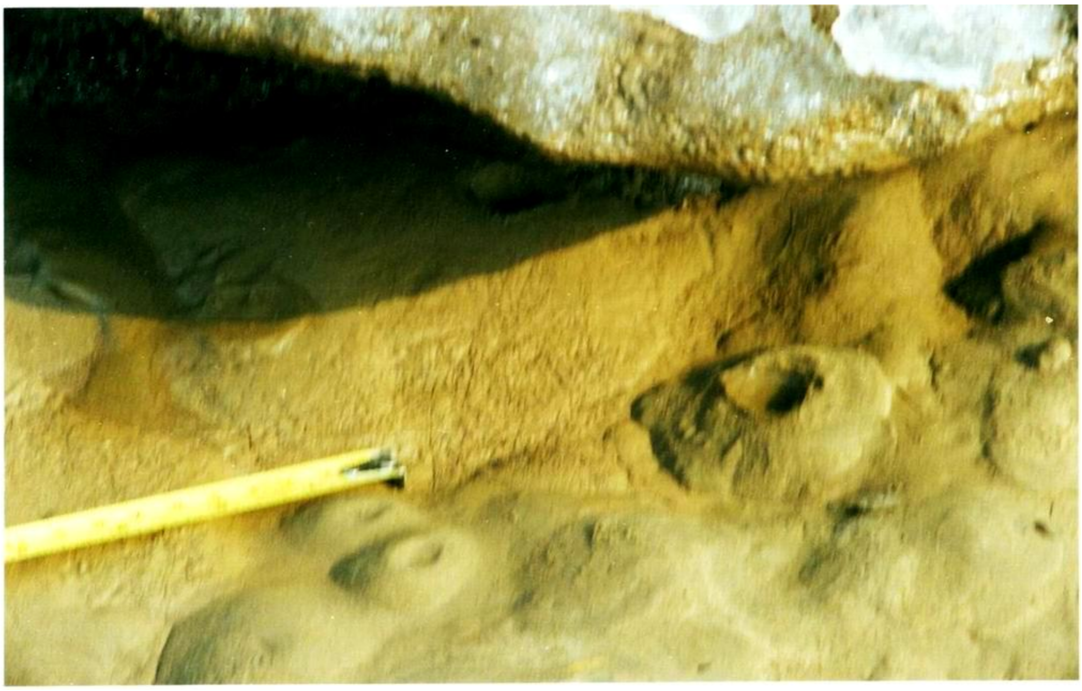

A few small ice water mud and silt volcanoes (Monroes & Dionne, 1972) still in the formation process. Both the mud and sand volcanoes have very similar shapes, though their genesis

mechanisms are not similar. These sedimentary structures were formed by meltwater dripping from the overhanging ice layer, which bears a certain amount of mud and silt.

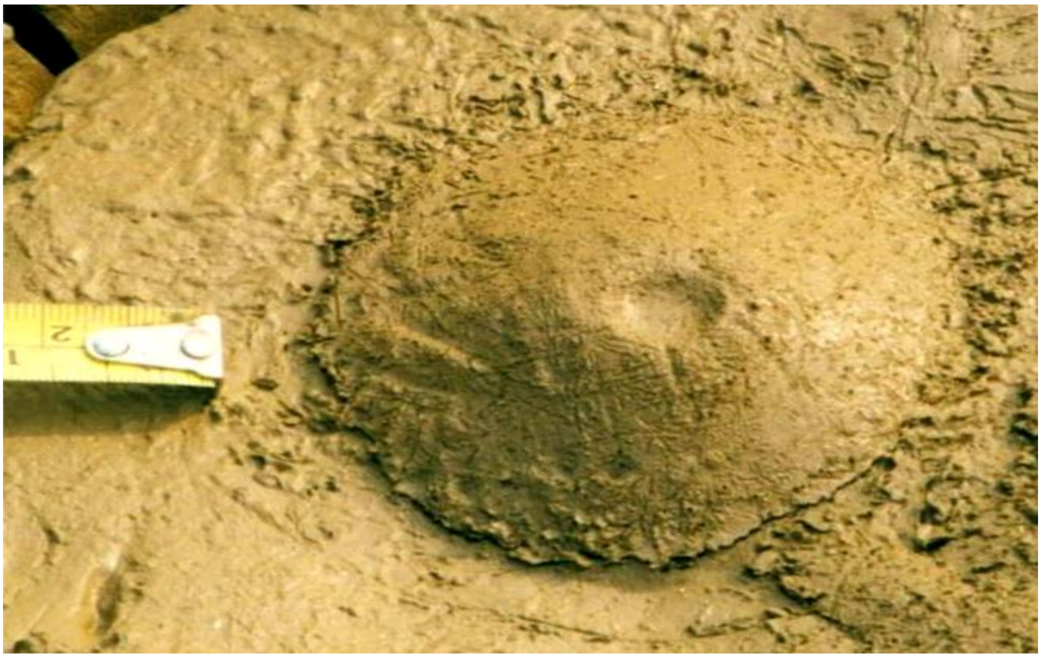

A small ice water mud and silt volcano, it has a crater near the centre, and there is a great deal of ice crystals on its walls and around it. Note: there is a fissure between the volcano and the channel bar surface, suggesting that the volcano could be easily taken away.

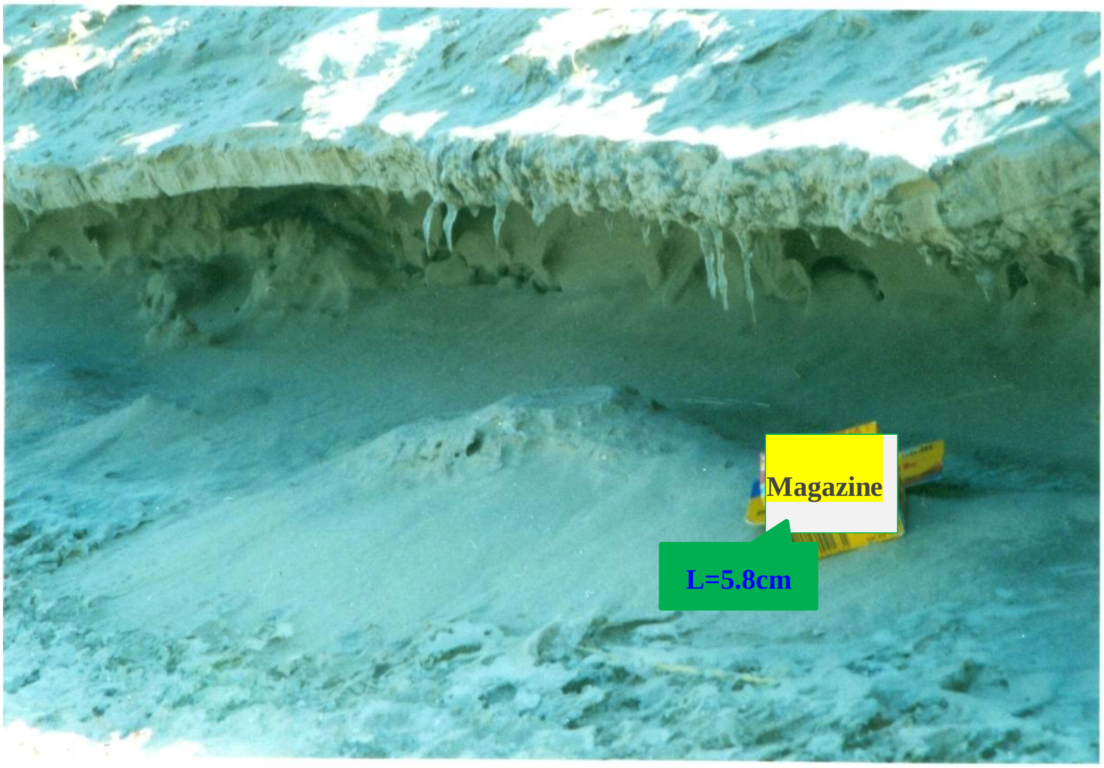

A medium-sized ice water silt volcano still in the formation process, it has a crater in the centre, and there is a great deal of rill marks on its walls, having been by ice water. Note: there is a thin overhanging layer of frozen silt, causing several fine icicles to form over the volcano.

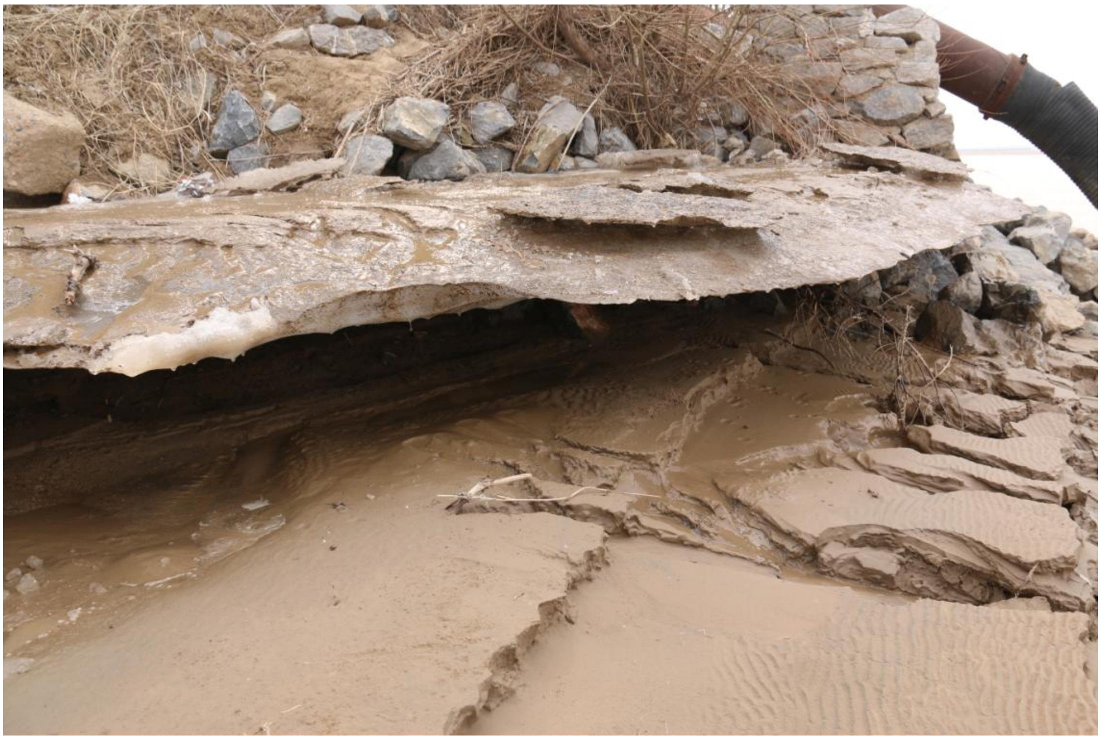

An overhanging ice layer with a little mud and silt and 4-5 small-sized ice water silt volcanoes. They are under construction and very small and extremely flat.

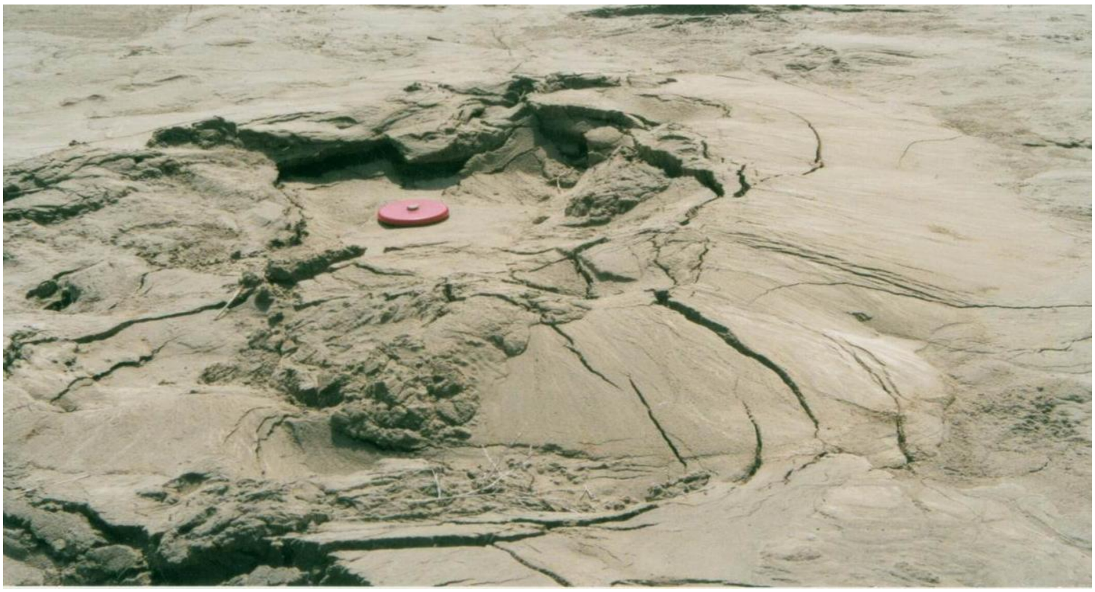

A large ice water silt volcano with a large, irregular crater in the centre. On its walls there is a great deal of rill marks which were formed by ice water.

21. Ice melt collapsed dome

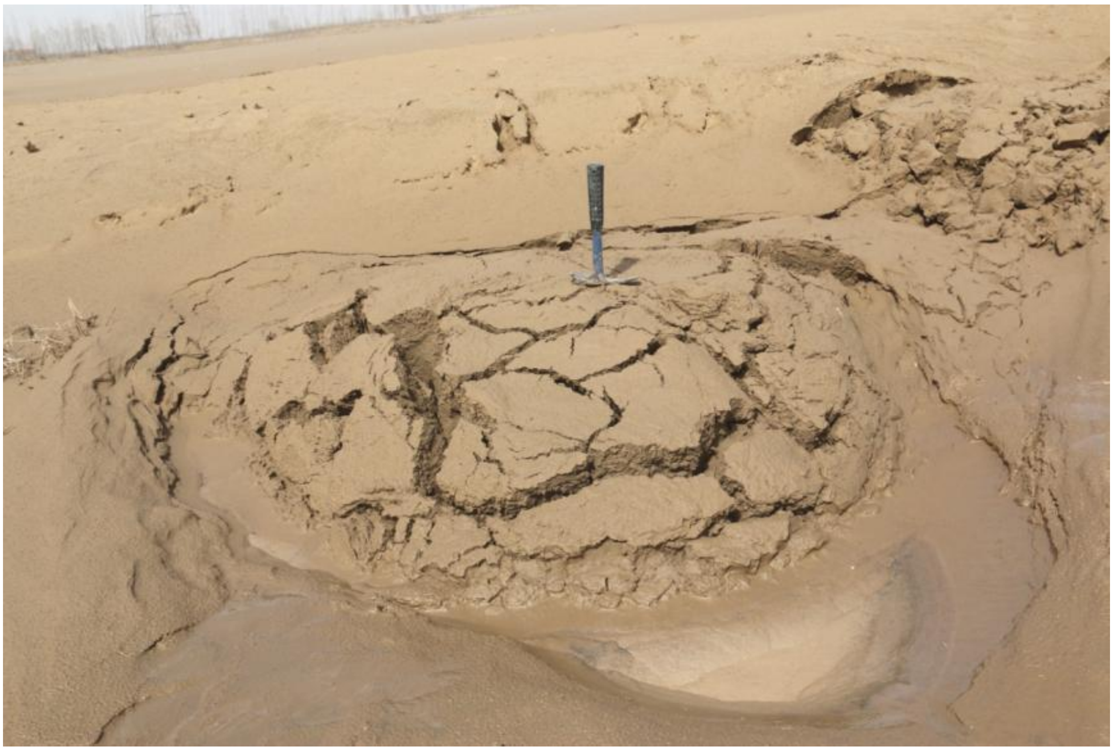

A ice melt collapsed dome with a lot of slump fissions and gently dipping walls in the forming process. Its formation mechanism is as same as a Chinese flower bun-like structure. The hammer is 28cm long.

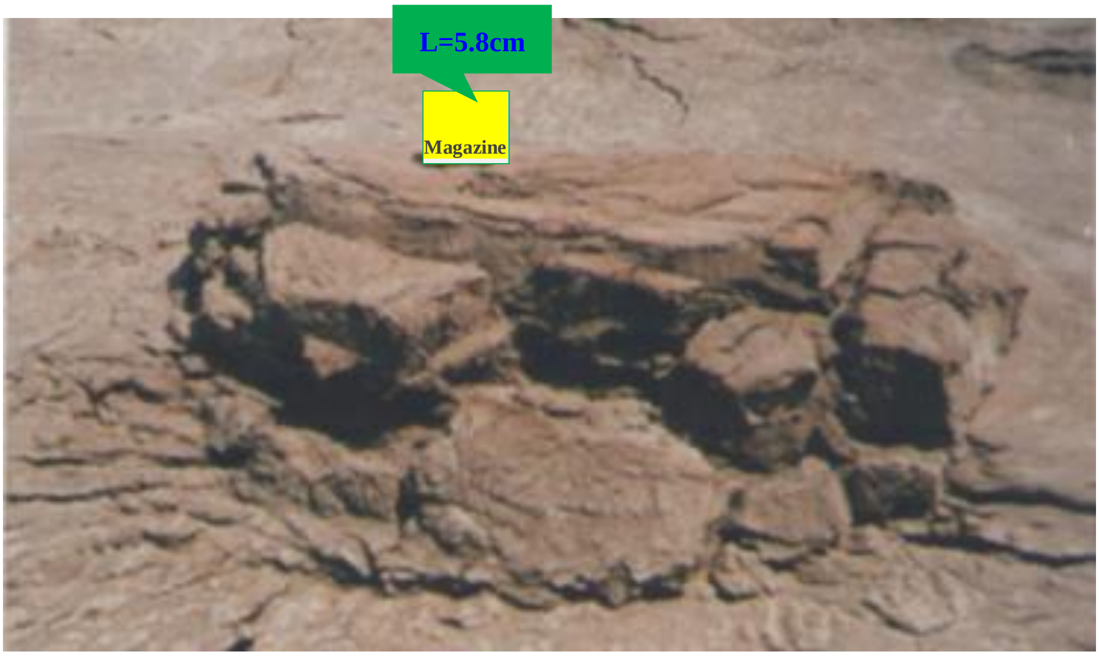

A nearly lateral view of an ice melt collapsed dome with a lot of slump fissions and upright walls. Its formation mechanism is as same as a Chinese flower bun-like structure.

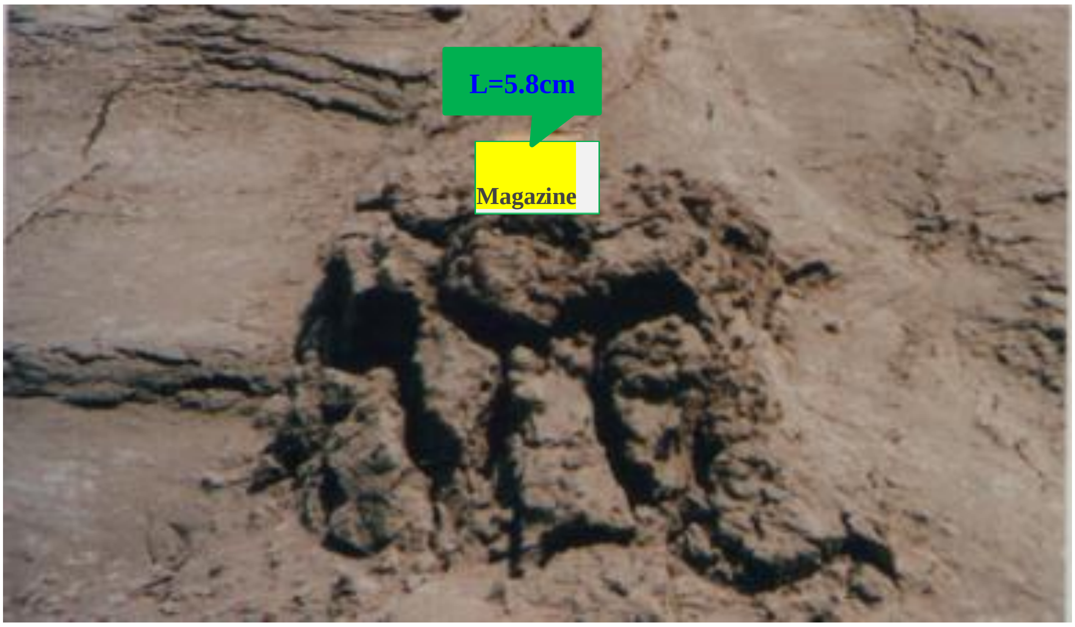

A another nearly lateral view of the aforementioned ice melt collapsed dome, also with a lot of slump fissions and upright walls. Its formation mechanism is as same as a Chinese flower bun-like structure.

22. Frozen slump gullies and steps

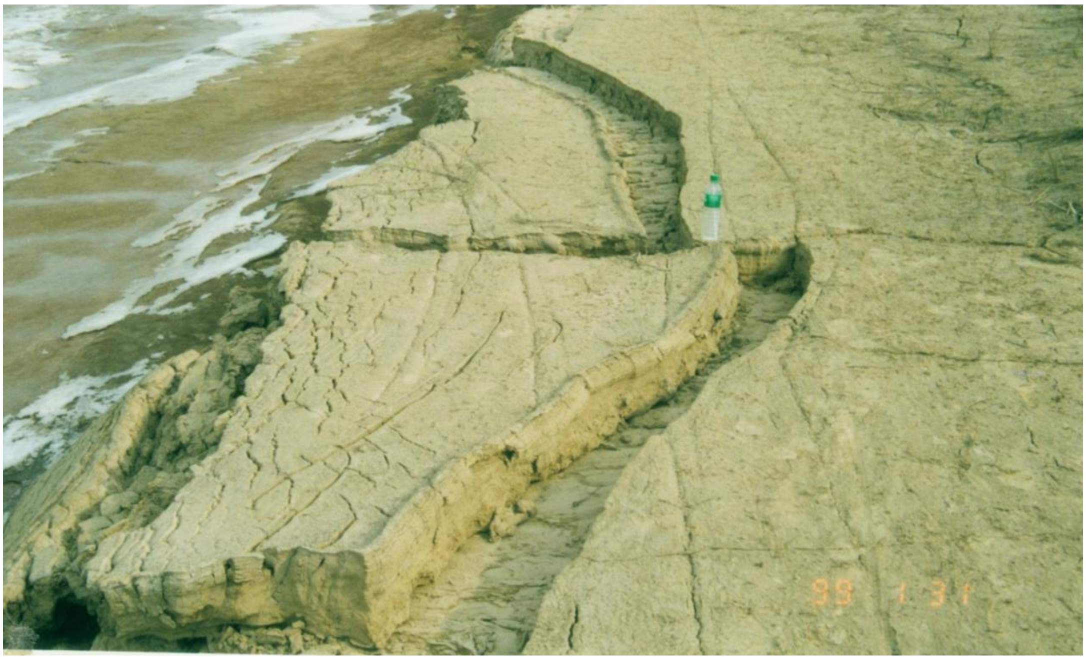

A frozen slump gully with upright slump walls, cut off by a large fissure in its middle. When frozen during the winter, the thin sediment layer near the surface becomes consolidated and may move integrally to form this unusual ice--induced sedimentary structure like a gully. Note a lot of fissions in the same direction as river flow developed on the surface of the sliding thin frozen silt layer. The bottle is about 24cm in height.

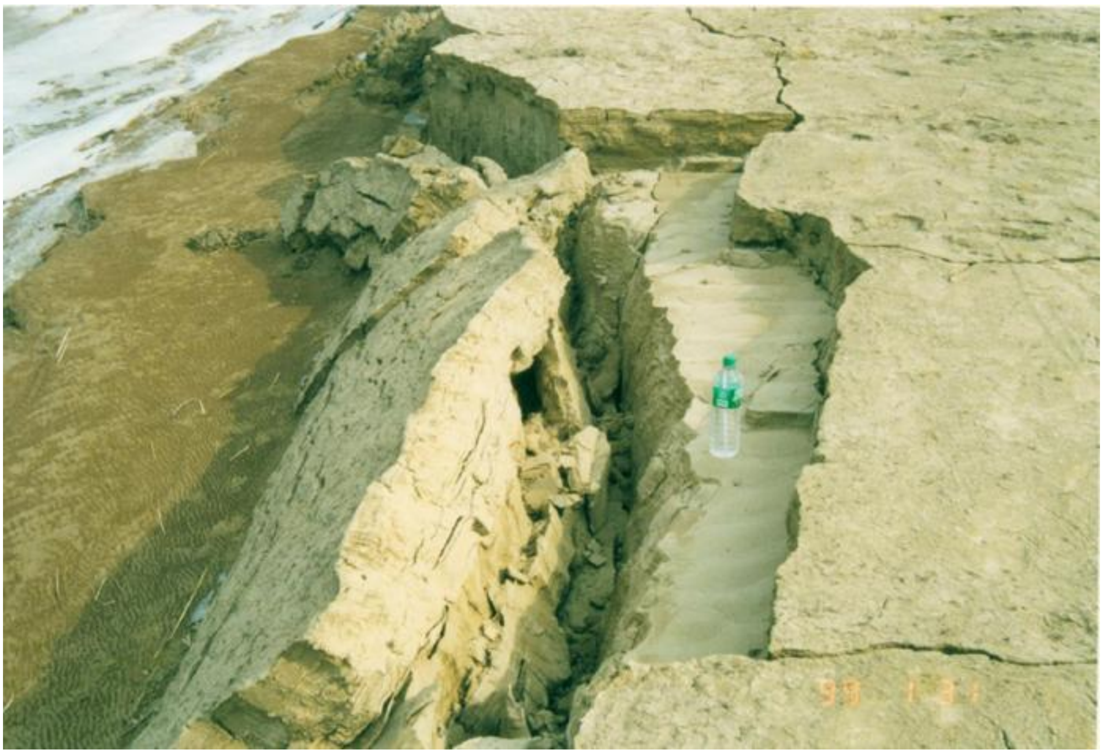

A frozen slump step. It has a steep wall and flat bottom with wave-like ripples. The collapsed block (on the left) piles up just nearly. In the upper left corner of the photograph, a thin layer of white ice can be seen. The bottle is about 24cm in height.

23. Ice water pits or ice water pots

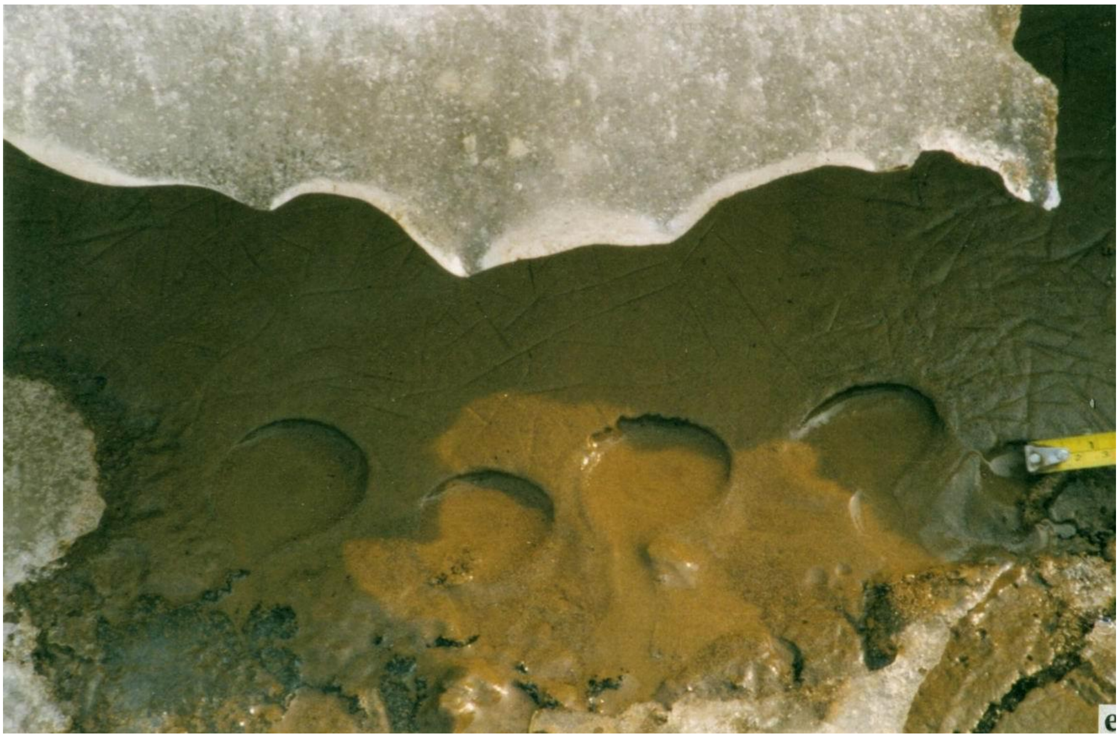

Four small pits which were formed by dripping meltwater. The overhanging ice layer can be seen near the top of the photograph, and the four pits formed just beneath it. Around the pits there are a great deal of ice crystals and melting ice fragments.

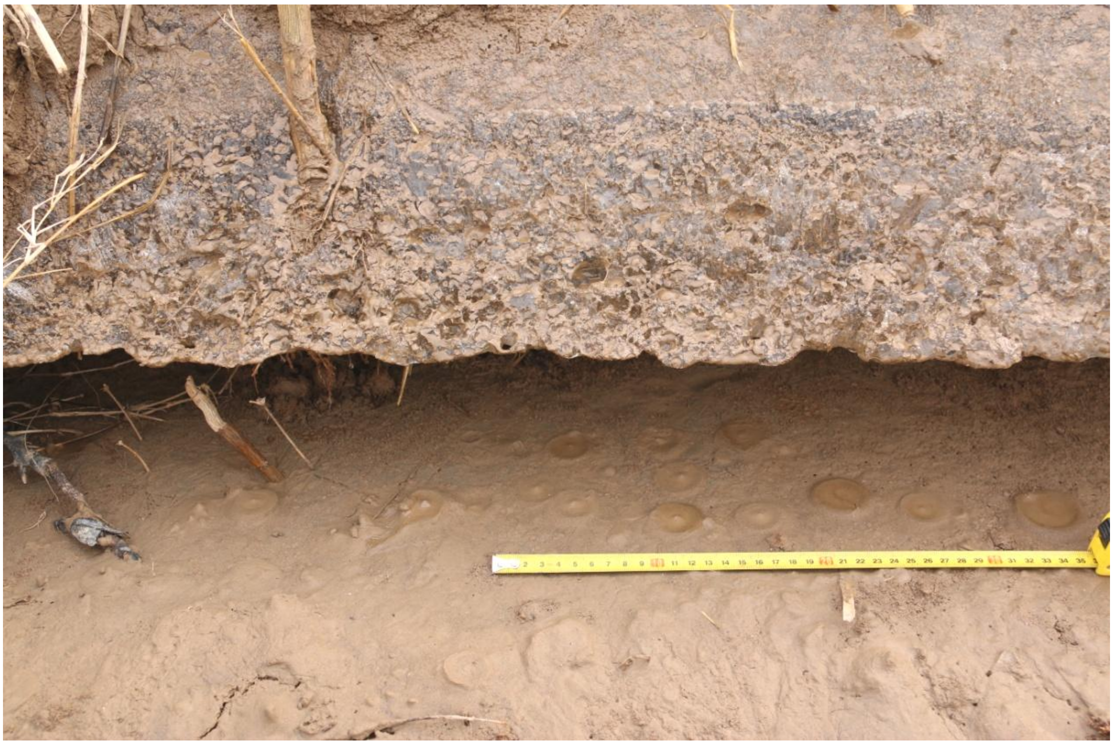

A group of small pits that were formed by dripping meltwater. Above is a thin, porous ice layer mixed with mud and silt, and just below it, about twenty small pits have formed.

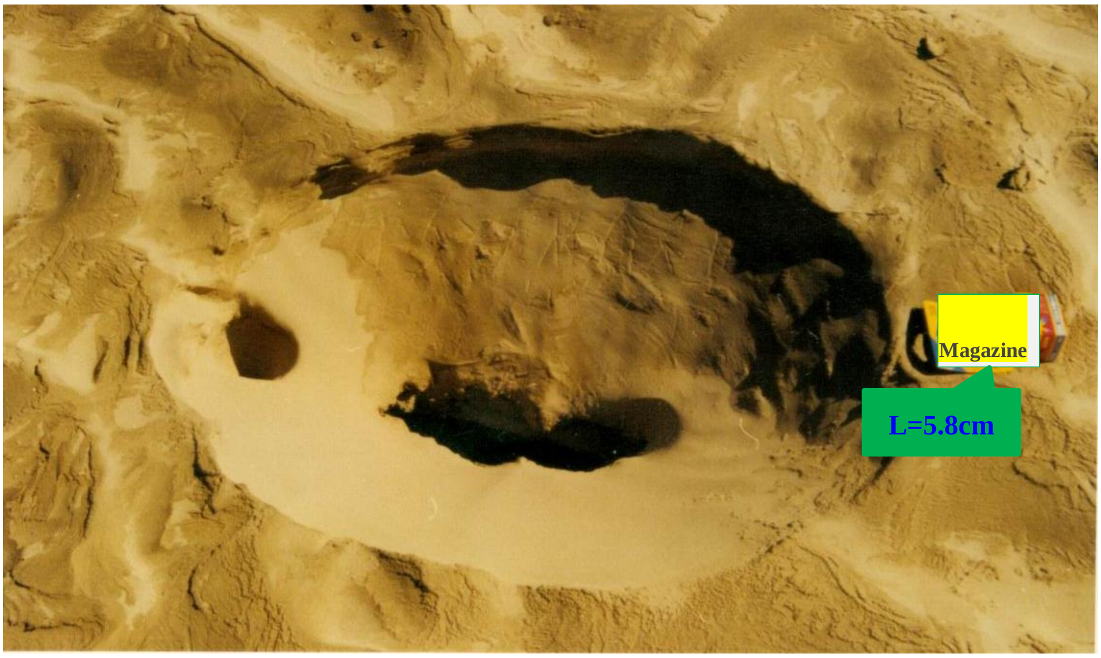

A large scale ice water pot. It has a diameter of about 62cm and a depth of 16cm. There are two centralised holes and another on the left-hand side. Some ice crystals have formed on the pot wall.

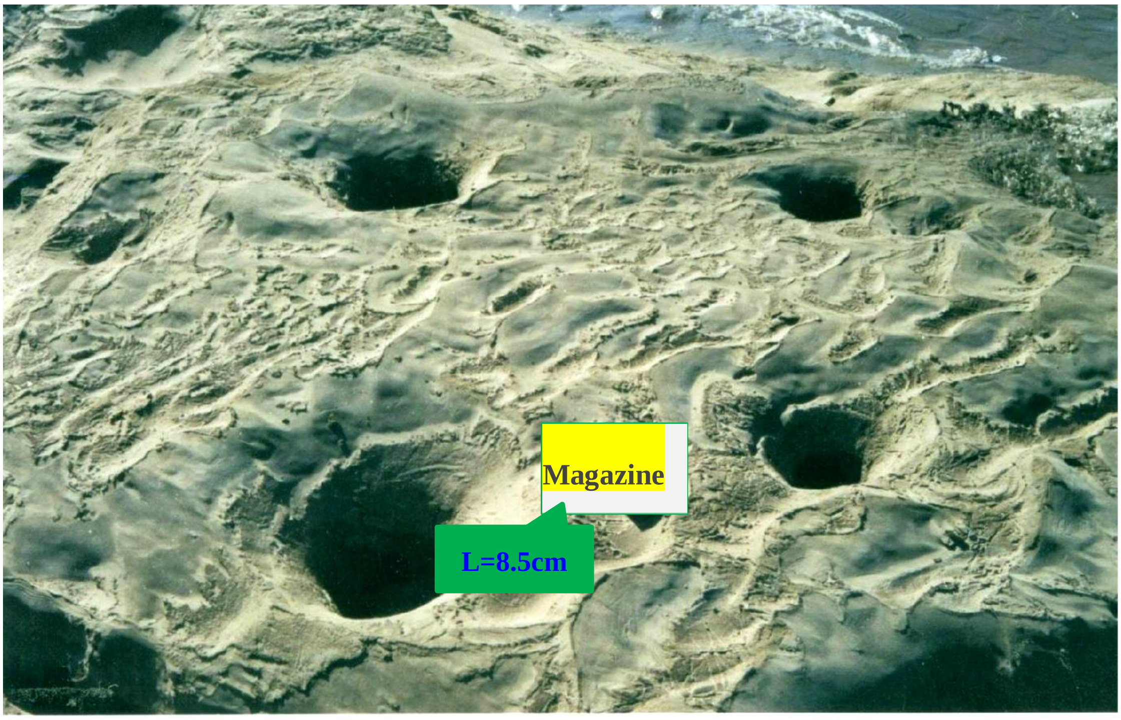

Four large scale ice water pots which have developed on the channel bar. They are 20-45cm in diameter and about 25cm deep.

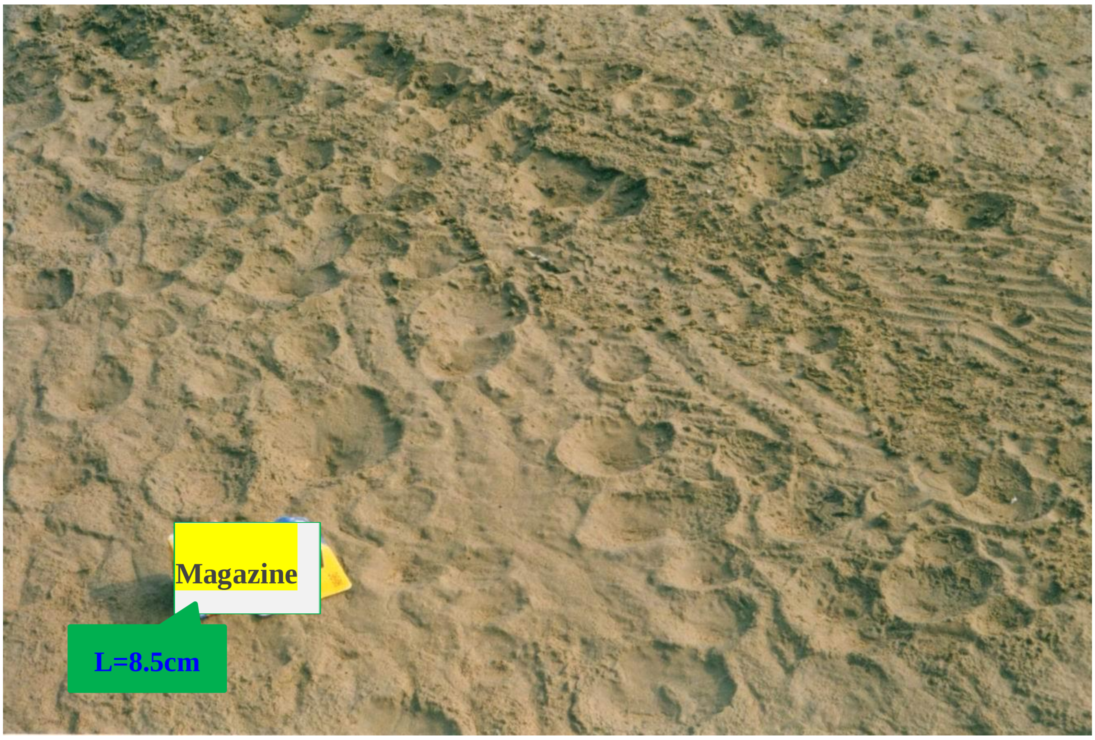

A dense grouping of several small ice water pits which have developed on the lower section of the point bar margin. They have fairly regular conical shapes and narrow ridges.

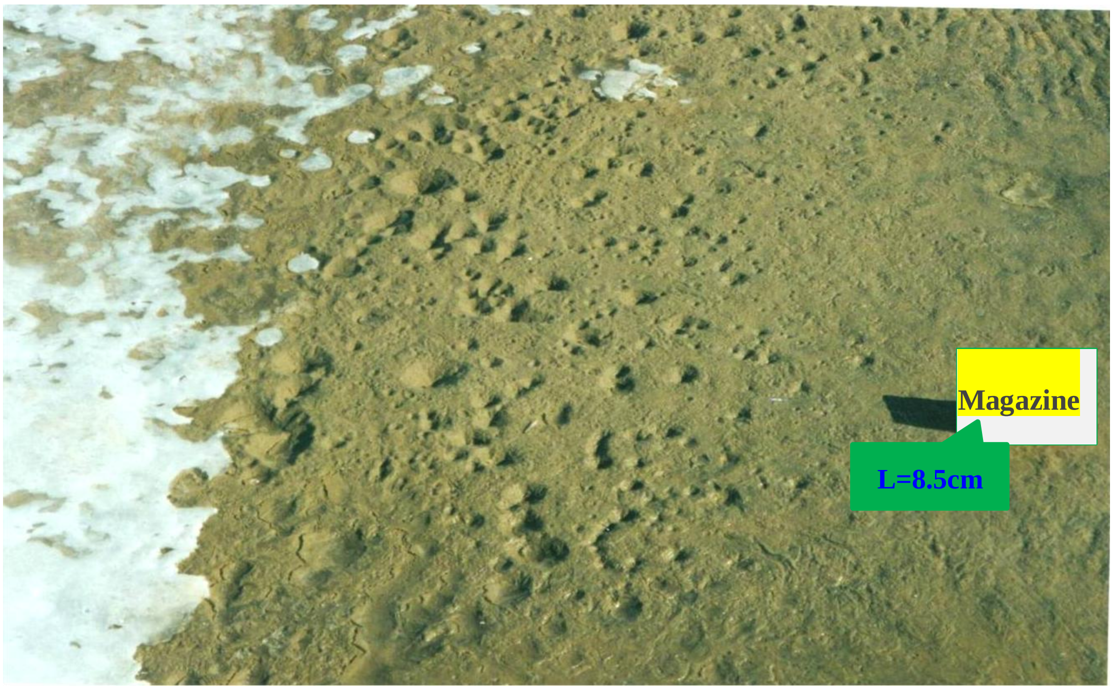

A dense grouping of several small ice water pits which have developed on the lower section of the point bar margin. They have fairly regular conical shapes and are accompanied by some ice water rill marks. On the left is a thin layer of white ice .

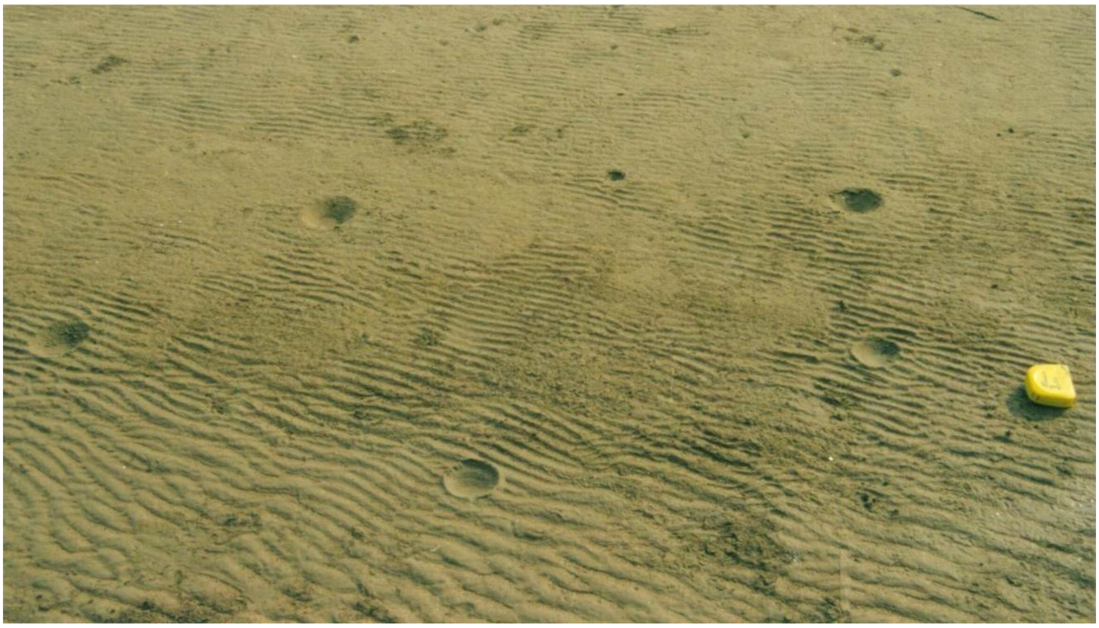

A few small ice water pits are spread sparsely on the channel bar, and are accompanied by small ripple patterns in the sediment.

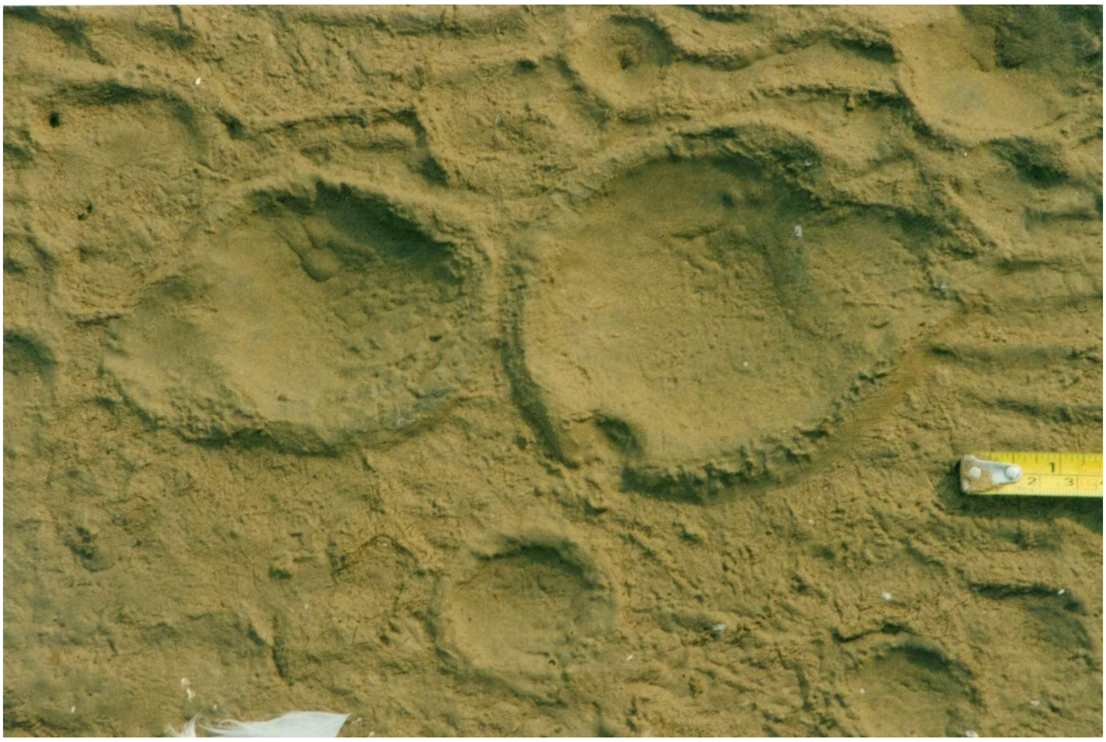

Small ice water pits with narrow ridges. They are shallow and flat and are accompanied by many ice crystals.

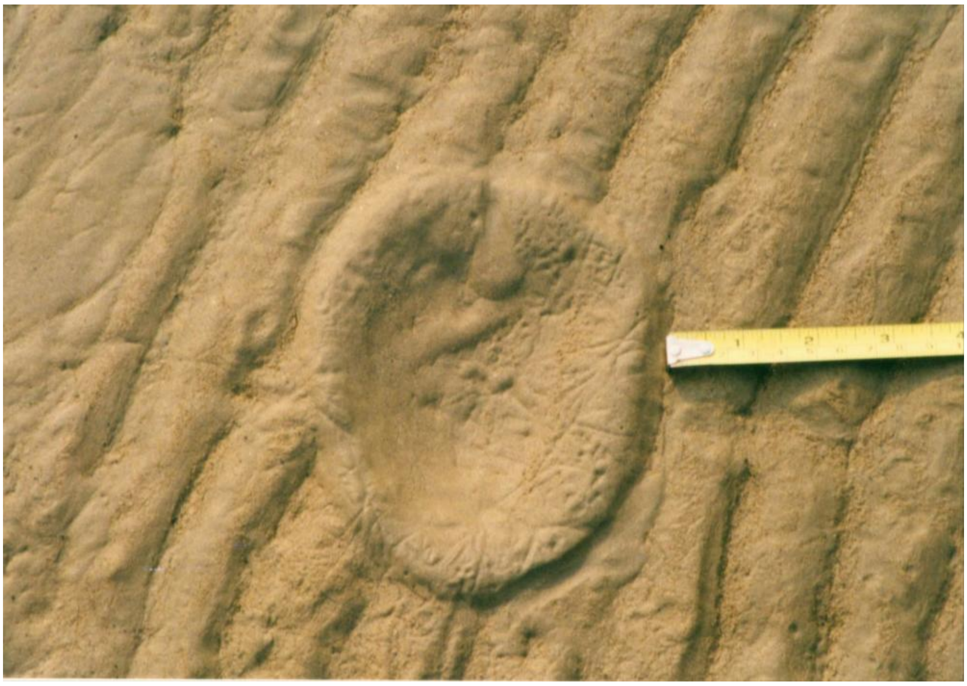

A well-developed small ice water pit with a relatively large ridge. It is shallow and flat and is accompanied by many ice crystals and small ripple patterns in the sediment.

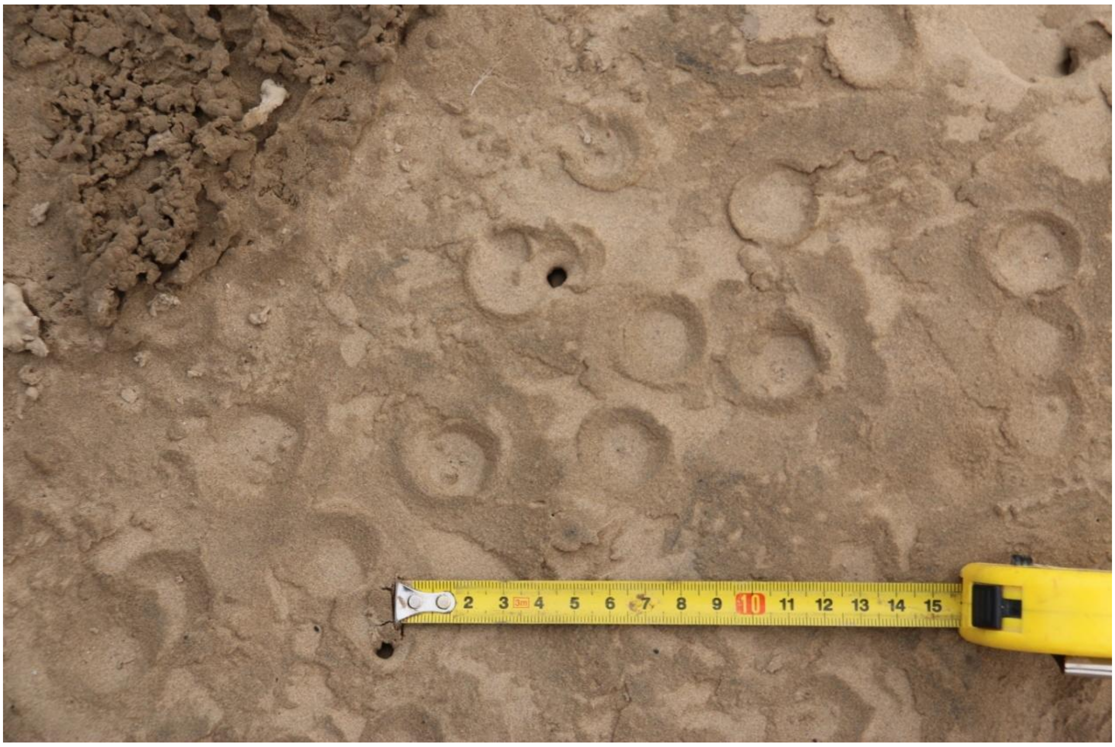

A group of well-developed small ice water pits which have developed on the point bar. They share a fairly regular shallow dish-like shape, and some have extremely narrow ridges. Their bottoms have been covered by alluvial silt.

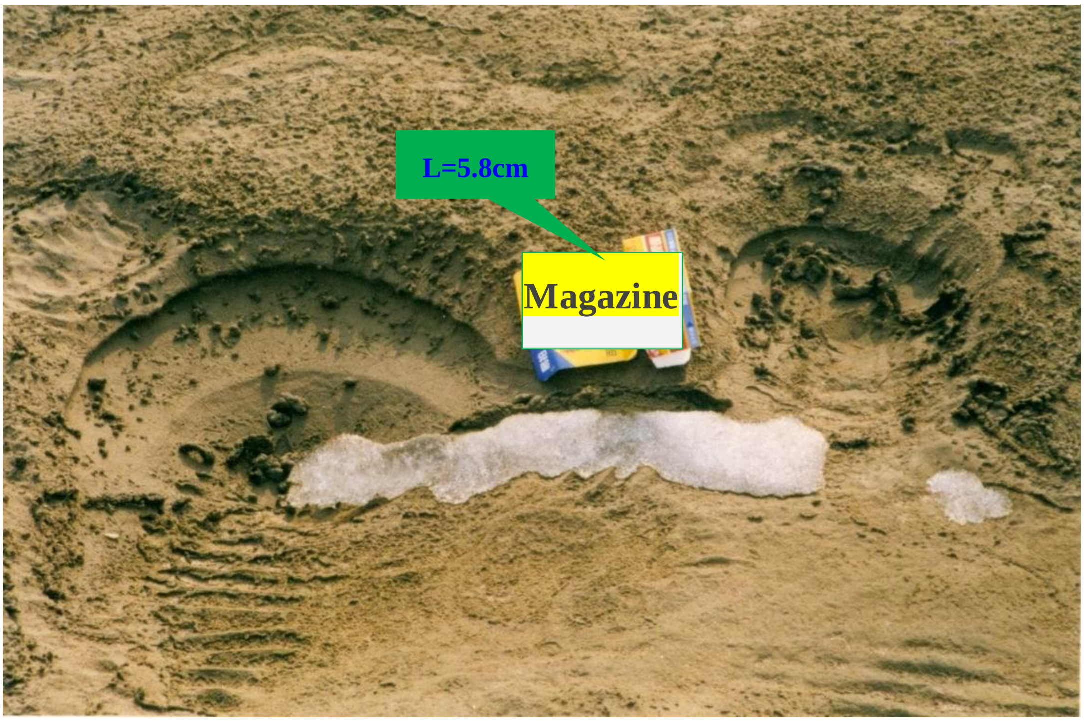

Two half-baked ice water pots. The upper wall is well-developed, whereas the lower wall is missing. They developed at the foot of the point bar.

24. Frozen cracks

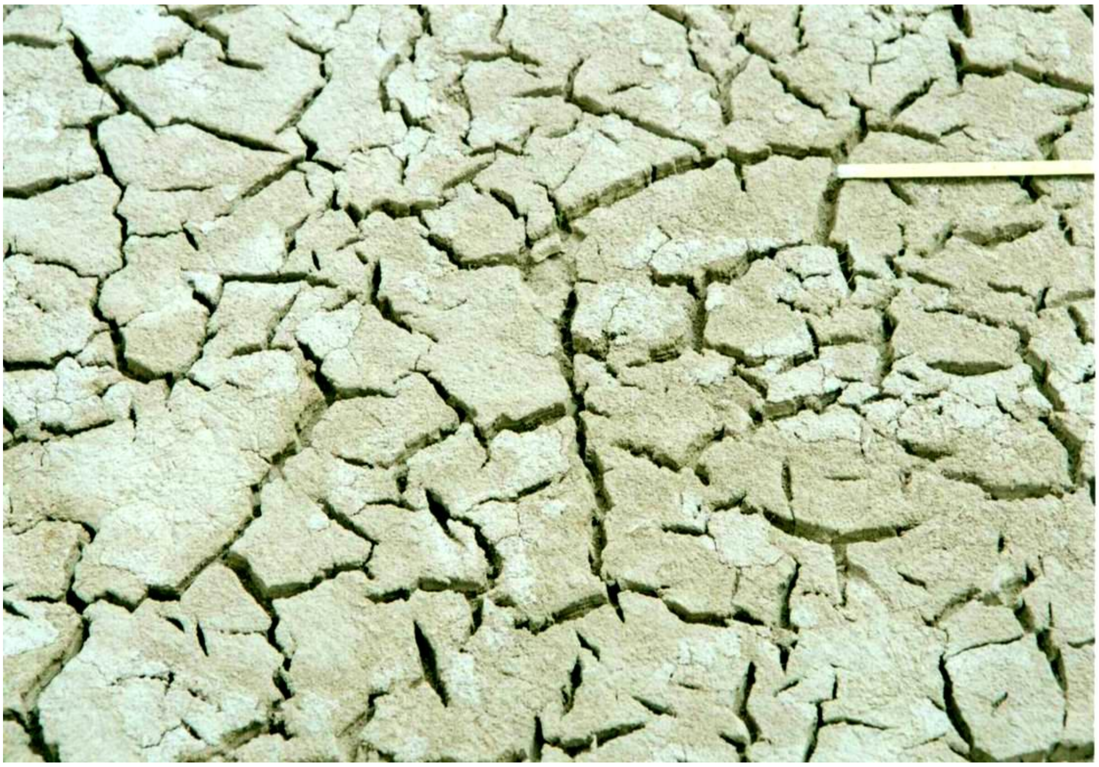

A nearly lateral view of an ice melt collapsed dome with a lot of slump fissions and upright walls. Its formation mechanism is as same as a Chinese flower bun-like structure.

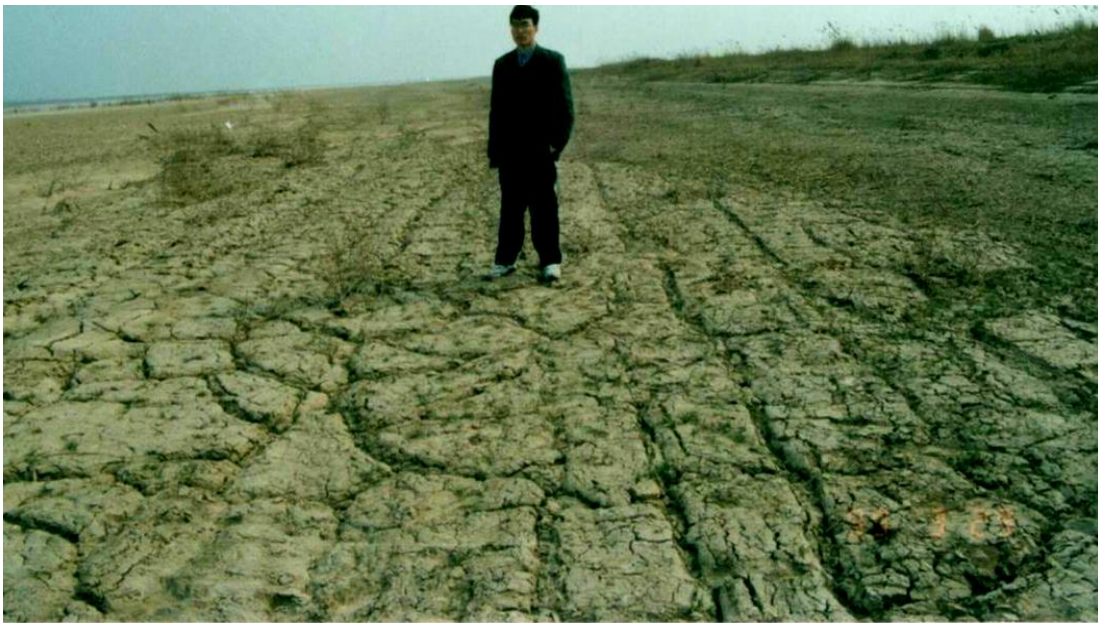

A group of large, longitudinal frozen cracks developed in the same direction as the river flow. Also, the frozen silt layer has a great deal of frozen bubbles, resulting in being like a sponge.
The person appearing in the figure is the co-author Liu Chuang.
